# Polymethoxylated *N*‐Carboranyl Isoquinolinones: A New Scaffold for ABCG2 Inhibitors

**DOI:** 10.1002/cmdc.202500708

**Published:** 2026-02-12

**Authors:** Lydia Kuhnert, Philipp Stockmann, Peter Lönnecke, Mara Anna Wolniewicz, Evamarie Hey‐Hawkins, Walther Honscha

**Affiliations:** ^1^ Institute of Pharmacology Pharmacy and Toxicology Faculty of Veterinary Medicine Universität Leipzig Leipzig Germany; ^2^ Institute of Inorganic Chemistry Faculty of Chemistry and Mineralogy Universität Leipzig Leipzig Germany; ^3^ Centre for Biotechnology and Biomedicine (BBZ) Faculty of Chemistry and Mineralogy Institute of Bioanalytical Chemistry Universität Leipzig Leipzig Germany; ^4^ Faculty of Chemistry and Chemical Engineering Department of Chemistry Babeş‐Bolyai University Cluj‐Napoca Romania

**Keywords:** ABCG2, breast cancer resistance protein, carborane, inhibitors, isoquinolinone

## Abstract

ABCG2‐mediated multidrug resistance (MDR) is a major challenge among chemotherapeutic treatments of colon, pancreatic, and breast cancer, as well as leukemia. Clinical oncology seeks new adjuvant therapeutics to overcome MDR by developing potent but nontoxic ABCG2 inhibitors. Aided by computational docking analyses, based on known substrate and inhibitor structural motifs, a new isoquinolinone framework and several (poly)methoxylated derivatives were designed and synthesized. The novel carborane‐containing *N*‐carboranyl isoquinolinones were evaluated for cytotoxicity, ABCG2 inhibition, and reversal of MDR in combination with mitoxantrone (MXN) in an ABCG2‐expressing Madin–Darby canine kidney II cell model. While the parental compound **IC‐1** showed strong ABCG2 inhibition, its 4‐methoxyphenyl, 3,4‐dimethoxyphenyl, and 3,4,5‐trimethoxyphenyl derivatives (**IC‐4**, **IC‐5,** and **IC‐6**) exhibited improved ABCG2 affinity. Nonsubstituted isoquinolinones **IC‐1** to **IC‐6** displayed higher solubility, lower toxicity, and similar ABCG2 inhibition and reversal of MXN resistance than 6,7‐dimethoxy‐isoquinolinone derivatives **IC‐7** to **IC‐11**. Especially, the 4‐methoxyphenyl‐ and 3,4‐dimethoxyphenyl‐substituted isoquinolinones (**IC‐10**, **IC‐11**) caused the strongest left shift of the MXN IC_50_ value by 8.1‐ and 7.2‐fold, indicating effective resensitization to the chemotherapeutic agent. Therefore, carborane‐containing isoquinolinones featuring additional methoxy groups represent a promising approach for the development of ABCG2 inhibitors to overcome resistance to anticancer drugs.

## Graphical abstract

Combinations of a boron‐based clusters (carborane) and polymethoxy group patterns are crucial to overcome ABCG2‐mediated multidrug resistance in cancer cells. (Created in BioRender. Kuhnert, L. (2025) https://BioRender.com/h4cnh5m).

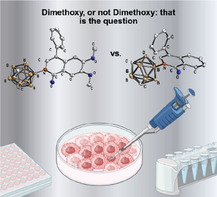

## 1 Introduction

Cancer remains one of the biggest challenges in developed countries, being the second leading cause of death worldwide [1]. Chemotherapy is considered a common anticancer strategy; however, treatment with promising chemotherapeutics is often hampered by multidrug resistance (MDR) [2, 3]. Several mechanisms are associated with MDR. Especially, the increased drug efflux via specific ABC transporter proteins plays a crucial role and may cause therapy failure [4, 5]. This drug efflux leads to an insufficient therapeutic concentration of the applied agents inside cancer cells, resulting in unsuccessful chemotherapy [6]. ABC transporters are ubiquitously expressed in human tissues and have a protective function by eliminating harmful xenobiotics from cells [7, 8]. Particularly, three ABC transporters have been directly connected to MDR‐related drug efflux in cancer cells: the P‐glycoprotein (P‐gp or ABCB1), MDR‐associated protein 1 (MRP1 or ABCC1), and breast cancer resistance protein (BCRP or ABCG2) [9, 10, 11, 12]. Thus, one way of approaching drug resistance in chemotherapy is the development of target‐specific inhibitors of these transporters to improve anticancer treatments.

Ever since ABCG2 was found to be overexpressed in different types of cancer, it has become a topic of interest in the field of medicinal chemistry to investigate novel inhibitors of ABCG2 exhibiting low toxicity, strong target‐specific inhibition, and high reversal potency [13, 14, 15]. Within the last decades, multiple generations of ABCG2 inhibitors have been developed. Until now, none of them reached clinical approval due to high toxicity and critical side effects [16, 17]. Different structural scaffolds and functionalities have been identified to be essential for potent inhibitory activity. Therefore, repeating patterns were used to design these inhibitors, with, for example, quinazoline [18, 19, 20, 21, 22], chalcone [23], and pyrimidine [24] scaffolds. In our previous work, we made use of the quinazoline framework and revealed the concept behind the synthesis and biological evaluation of new ABCG2 inhibitors to successfully reverse MDR, employing an inorganic boron–carbon cluster moiety (*closo*‐dicarbadodecaborane, carbaborane, and carborane) as a pharmacophore [25].

**FIGURE 1:**
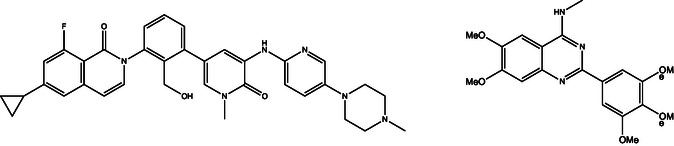
Molecular structures of the BTK inhibitor RN486 [32] (left) and a previously reported polymethoxylated *N*‐carboranyl quinazoline inhibitor of ABCG2 (DMQCd, right) [25].

Within the spectrum of ABCG2 substrates and inhibitors, an embellished benzamide structure is a frequently recurring structural feature. However, we hypothesized that a more rigid form, as a heterocyclic ring system, may be beneficial to improve ABCG2 inhibition. In detail, hetero(bi)cyclic aromatic ring systems are preferable for successful inhibition [16, 26]. Thus, the isoquinolin‐1(2*H*)‐one framework appears as a proficient base for further optimization. Isoquinolinones are found in different natural products, such as alkaloids like oxynitidine [27], and further received attention in drug discovery over the years with being used for developing inhibitors of, for example, hypoxia‐inducible factor 1 (HIF‐1) [28], dihydroorotate dehydrogenase [29], human epidermal growth factor receptor 2 (HER2) [30], or bromodomain and extra‐terminal motif (BET) [31]. Thus far, reversal of MDR by the isoquinolinone scaffold has only been described for the Bruton's tyrosine kinase inhibitor RN486 (Figure 1) mediating a down‐regulation of ABCG2 gene expression. A direct inhibition of the membrane transporter has not been reported [32]. To that extent, we aimed to fuse the isoquinolinone core with a previously successfully introduced carborane moiety and various methoxy substitution patterns. Carboranes are boron–carbon‐based clusters which are able to increase target affinity by means of their advantageous properties, namely high hydrophobicity, steric demand, etc. [33, 34, 35]. As methoxy substituents have been demonstrated to be beneficial functionalities for ABCG2 inhibitors in previous studies, we further observed that a higher number of methoxy substituents resulted in stronger inhibition toward ABCG2 (Figure 1) [25, 36].

Herein, we report the design and synthesis of unsubstituted and (poly)methoxylated *N*‐carboranyl isoquinolinone and *N*‐carboranyl 4‐phenylisoquinolinone derivatives **IC‐1** to **IC‐11**. Further, in vitro studies on the cytotoxicity, the ability to inhibit ABCG2 efflux activity and to reverse mitoxantrone (MXN) resistance in ABCG2‐overexpressing cells were performed to investigate the inhibitory potential of the presented novel isoquinolinone structures.

## 2 Results and Discussion

### 2.1 Structures of Known ABCG2 Inhibitors

**FIGURE 2:**
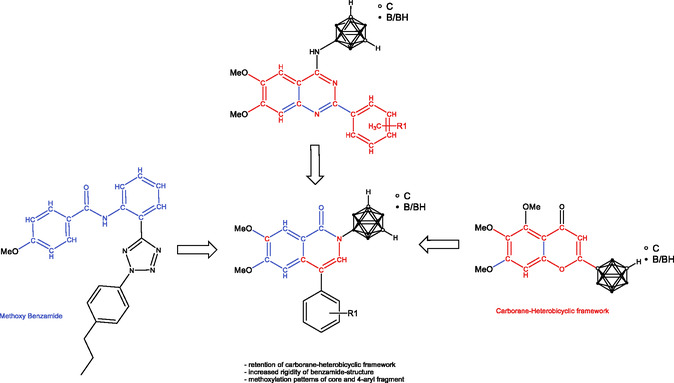
Rational design of polymethoxylated *N*‐carboranyl isoquinolinones. Based on the structural motif of methoxylated *N*‐phenyl benzamide (left) [41] and previous investigated carborane‐heterobicyclic frameworks, like polymethoxylated *N*‐carboranyl quinazoline (mid top) [25] and 5,6,7 trimethoxyborcalein (right) [36, 42], polymethoxylated *N*‐carboranyl isoquinolinones (mid down) were selected.

In recent years, the library of different basic structures of effective ABCG2 inhibitors expanded, ranging from pyrimidine [24], quinazoline [18, 19], chalcone [23], and benzamide‐based tetrazoles [37] to different hybrid structures [38, 39, 40]. However, a benzamide moiety appears to be an often‐occurring structural feature among the known ABCG2 substrates and inhibitors, for example, the phenyltetrazolyl‐phenylamide structure presented by Köhler et al. [41], exhibiting strong inhibitory potential. In this work, we aimed to merge the benzamide scaffold with higher rigidity and the presumably essential motif of a conjugated annelated ring system to form an isoquinolin‐1(2*H*)‐one framework as a foundation for new carborane‐functionalized hybrid ABCG2 inhibitors, illustrated in Figure 2 as an overview of rational design.

### 2.2 Syntheses

**SCHEME 1:**
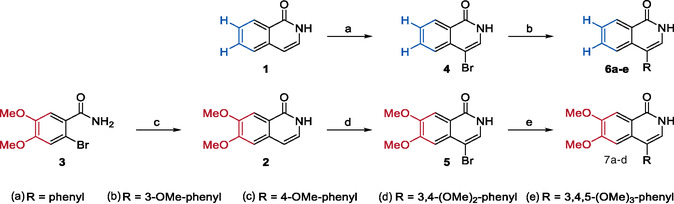
Synthesis of 4‐substituted isoquinoline‐1(2*H*)‐ones **6a–e** and **7a–d**. Reagents and conditions: (a) NBS, DMF, rt, 3 h; (b) boronic acid (1.2 equiv.), Cs_2_CO_3_, dicyclohexyl(2′,6′‐dimethoxy[1,1′‐biphenyl]‐2‐yl)phosphane (SPhos, 10 mol%), [Pd(PPh_3_)_4_] (5 mol%), 1,4‐dioxane/H2O (4:1, *v/v*), reflux, overnight; (c) (i) (E)‐2‐(2‐ethoxyvinyl)‐4,4,5,5‐tetramethyl‐1,3,2‐dioxaborolane (1.5 equiv.), Cs_2_CO_3_, [Pd(PPh_3_)_4_] (5 mol%), 1,4‐dioxane/H_2_O (3:1, *v/v*), 80°C, overnight, (ii) trifluoroacetic acid (20 equiv.), 0°C to rt, 4.5 h; (d) Br_2_, AcOH, rt, 3.5 h.

**SCHEME 2:**
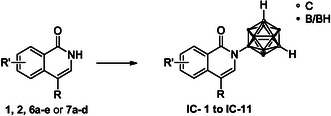
General synthesis of 4‐substituted *N*‐carboranyl isoquinolinones (**IC‐1** to **IC‐11**). Reagents and conditions: *closo*‐9‐bromo‐*meta*‐carborane (1.5 equiv.), KO^t^Bu (2.0 equiv.), SPhos (5 mol%), (methanesulfonato‐*κ*O)[2′‐(methylamino)‐2‐biphenylyl]palladium‐dicyclohexyl(2′,6′‐dimethoxy‐2‐biphenylyl)phosphine (SPhos Pd G4, 5 mol%), 1,4‐dioxane, reflux, overnight.

The *N*‐carboranyl isoquinolinone derivatives were prepared in three steps, starting from an unsubstituted or 6,7‐dimethoxylated isoquinolin‐1(2*H*)‐one (**1** and **2**, respectively; Scheme 1). Compound **2** was synthesized after Schütz et al., originating from 2‐bromo‐4,5‐dimethoxybenzamide (**3**, Scheme 1) [43]. For further modification in the four‐position, the isoquinolinones **1** and **2** were selectively brominated, using *N*‐bromosuccinimide (NBS) in DMF or bromine in acetic acid, respectively [44, 45]. The brominated isoquinolinones **4** and **5** were substituted in a Suzuki–Miyaura cross‐coupling reaction with the appropriate boronic acids, generating the 4‐substituted isoquinolinones with an unsubstituted scaffold (**6a–e**) and a 6,7‐dimethoxy‐substituted scaffold (**7a–d**). In a last step, the desired *N*‐carboranyl target compounds **IC‐1** to **IC‐11** were achieved in a Buchwald–Hartwig‐like cross‐coupling reaction, using SPhos Pd G4 (Scheme 1) and closo‐9‐bromo‐*meta*‐carborane (Scheme 2, Table 1) [46]. For both underlying isoquinolinone scaffolds (unsubstituted **6a–e** and 6,7‐dimethoxy‐substituted **7a–d**), with increasing degrees of methoxy substitution of the phenyl boronic acid, the yields steadily decreased.

**TABLE 1:** Cytotoxicity of carboranyl isoquinolinones, determined in a WST‐1 cell proliferation assay using MDCKII wild‐type (MDCKII‐WT) and human ABCG2‐transfected MDCKII cells (MDCKII‐hABCG2).

Compound	R	R′	IC_50_ ± SEM, µM	
			MDCKII‐WT	MDCKII‐hABCG2
**IC‐1**	H	H	>50	>50
**IC‐2**	Phenyl	H	>25^a^	>25^a^
**IC‐3**	3‐OMe‐phenyl	H	>25^a^	>25^a^
**IC‐4**	4‐OMe‐phenyl	H	>25^a^	>25^a^
**IC‐5**	3,4‐(OMe)_2_‐phenyl	H	>50	>50
**IC‐6**	3,4,5‐(OMe)_3_‐phenyl	H	>50	>50
**IC‐7**	H	6,7‐(OMe)_2_	>10^a^	>10^a^
**IC‐8**	Phenyl	6,7‐(OMe)_2_	>25^a^	>25^a^
**IC‐9**	3‐OMe‐phenyl	6,7‐(OMe)_2_	>5^b^	>5^b^
**IC‐10**	4‐OMe‐phenyl	6,7‐(OMe)_2_	~50	>50
**IC‐11**	3,4‐(OMe)_2_‐phenyl	6,7‐(OMe)_2_	>50	>50

*Note:* Data given as mean ± SEM of three independent experiments (*N* = 3).

a Highest applied concentration 25 µM, due to poor solubility in DMSO.

b Highest applied concentration 5 µM, due to poor solubility in DMSO.

All target compounds (Table 1) were fully characterized, and their structures were elucidated by (2D) NMR spectroscopy (Figures S1–S105) and HR‐MS (Figures S106–S127). Furthermore, X‐ray crystallography studies were conducted on single crystals of **IC‐1**, **IC‐2**, **IC‐4**, **IC‐8,** and **IC‐10** (Figures S128–S132, Table S1). Purity of compounds **IC‐1** to **IC‐11** was confirmed by elemental analysis.

### 2.3 Evaluation of Solubility of IC‐1 to IC‐11

The solubility of the compounds is presented in Table 1. To maintain a low solvent concentration, a maximum of 0.1% DMSO was used in the final dilution. Therefore, it was possible to achieve a stock concentration of 50 mM for **IC‐1**, **IC‐5**, **IC‐6**, **IC‐10,** and **IC‐11,** which were further diluted into a 50 µM working solution. **IC‐9** demonstrated the lowest solubility in DMSO with 5 mM. All other compounds reached a solubility of 25 mM in the stock concentration. While a single *meta*‐methoxy substitution on the phenyl ring (**IC‐9**) appears to decrease the solubility, a single *para*‐methoxy substitution (**IC‐10**) or 6,7‐dimethoxy substitution (**IC‐11**) did not.

### 2.4 Evaluation of Cytotoxicity in Madin–Darby Canine Kidney II (MDCKII‐hABCG2 and MDCKII‐Wild‐Type (WT) Cells

The cytotoxic activity of **IC‐1** to **IC‐11** on parental WT and human ABCG2‐expressing (hABCG2) MDCKII cells was determined by water‐soluble tetrazolium 1 (WST‐1) cell proliferation assay. The obtained IC_50_ values (Table 1) demonstrate a decreased cell viability of 50%, referenced to Triton X‐100 and solvent (0.1% DMSO) as positive and negative controls, respectively. Calculation of substance‐specific IC_50_ values was not possible, as complete concentration‐effect curves were not available due to low solubility of the compounds above 50 µM. The complete concentration‐effect curves are shown in Figures S133 and S134 (Supporting Information). The investigated compounds **IC‐2** to **IC‐5** (Figure S133B–E), **IC‐8** (Figure S134B), and **IC‐9** (Figure S134C) did not influence the cell viability at any tested concentrations. There were no significant differences observed between ABCG2‐expressing and WT MDCKII cells. Compounds **IC‐1**, **IC‐6**, **IC‐7,** and **IC‐10** significantly decreased the cell viability by more than 40% (Figures S133A,F and S134A,D). As shown in the graphs (Figures S133–S134), this effect was more pronounced in MDCKII‐hABCG2 cells than in the parental WT cells, which potentially suggests a higher accumulation of ABCG2‐inhibiting compounds.

### 2.5 Hoechst 33342‐Based Investigation of ABCG2 Inhibition

**FIGURE 3:**
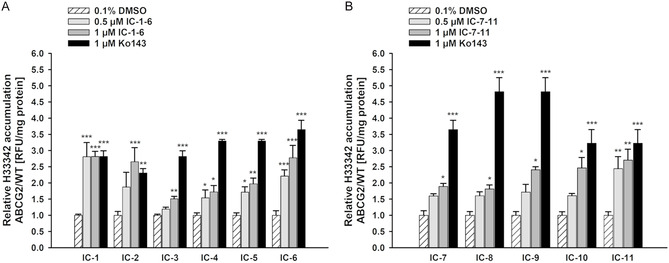
Relative intracellular Hoechst 33342 accumulation in MDCKII‐hABCG2 cells in relation to MDCKII‐WT cells. Cells were treated with 0.5 and 1.0 µM of (A) unsubstituted *N*‐carboranyl isoquinolinones **IC‐1** to **IC‐6**, and (B) *N*‐carboranyl 6,7‐dimethoxyisoquinolinones **IC‐7** to **IC‐11**, the positive control Ko143 (1.0 µM) or solvent control (0.1% DMSO) for 4 h. Afterward, intracellular Hoechst 33342 accumulation was determined which represents an ABCG2 interaction of the investigated compounds. Data were normalized to solvent control and are presented as mean ± SEM (*N* = 5, one‐way ANOVA with Holm–Šidák post hoc test, * indicating significant difference in comparison to the solvent control, ***p ≤ 0.001, **p ≤ 0.01, *p ≤ 0.05).

The inhibitory activity of the investigated compounds **IC‐1** to **IC‐11** were assessed in the Hoechst 33342 accumulation assay in an MDCKII cell model, based on a recently published protocol [25, 36]. The fluorescent dye Hoechst 33342 is a known substrate of the ABCG2 transporter. By actively inhibiting the ABCG2 protein through the examined compounds, the intracellular Hoechst 33342 concentration is increased. This accumulation is reflected by an enhanced intracellular fluorescence and thus, the degree of fluorescence indicates the degree of inhibition. MDCKII cells were treated with the investigated compounds in two concentrations (0.5 µM; 1.0 µM). Ko143, a commercially available ABCG2 inhibitor, was used as reference (1.0 µM) and 0.1% DMSO as solvent control. The relative intracellular Hoechst accumulation was compared to solvent control. As presented in Figure 3, all investigated compounds led to a significant increase of intracellular Hoechst accumulation at least at 1.0 µM. Due to the Hoechst 33342 accumulation assay not being quantifiable, a higher ABCG2 affinity is represented by a significant increase of the intracellular fluorescence in lower concentrations of the investigated inhibitors. Compounds **IC‐1, IC‐2, IC‐4**, **IC‐5, IC‐6** (Figure 3A), and **IC‐11** (Figure 3B) were able to inhibit the target protein in nanomolar ranges. While the unsubstituted compound **IC‐1** exhibited a strong ABCG2 inhibition at 0.5 and 1.0 µM, the addition of a phenyl ring at position four (**IC‐2**) decreased the activity. However, with an increasing degree of substitution of methoxy groups on the 4‐phenyl substituent, the ABCG2 inhibition is re‐enhanced. This is shown by a stronger inhibition of double and threefold methoxy substituted derivatives **IC‐5** and **IC‐6** in comparison to **IC‐2, IC‐3,** and **IC‐4** (Figure 3A). A similar effect of methoxy group patterns was already described in the literature [47] and was also shown in our previous projects [25, 36]. 6,7‐Dimethoxy‐substituted *N*‐carboranyl isoquinolinone derivatives (**IC‐7** to **IC‐11**) were modified with an increasing degree of methoxy substitution of the phenyl ring, similar to **IC‐1** to **IC‐6**. In comparison to the unsubstituted compound **IC‐1**, the 6,7‐dimethoxy‐substituted derivative **IC‐7** exhibited a lower solubility, a higher toxicity and a lower ABCG2 inhibition. A similar correlation is observed for the phenyl‐substituted compound **IC‐2** compared to **IC‐8**. The 3‐methoxy‐substituted compounds **IC‐3** and **IC‐9** possess analogous ABCG2‐inhibiting properties, with **IC‐9** exhibiting lower solubility in DMSO (**IC‐3**: 25 mM; **IC‐9**: 5 mM). The 4‐methoxy‐substituted *N*‐carboranyl isoquinolinone **IC‐4,** compared to its 6,7‐dimethoxy‐substituted analog **IC‐10,** could also enable an inhibition at 0.5 µM, while **IC‐10** only inhibited in 1.0 µM concentration. The 3,4‐methoxylated derivatives **IC‐5** and **IC‐11,** as well as the threefold methoxylated compound **IC‐6,** showed the strongest ABCG2 inhibition in both applied concentrations. In addition to the parental compound **IC‐1**, these results suggest a greater improvement of the ABCG2 inhibition by substitution of a phenyl ring with three methoxy groups to the unsubstituted *N*‐carboranyl isoquinolinone than the *N*‐carboranyl 6,7‐dimethoxy‐substituted isoquinolinone.

### 2.6 Absence of Autofluorescence

Bicyclic aromatic frameworks potentially feature intrinsic fluorescence, influencing the Hoechst 33342 accumulation assay [25, 35]. In order to exclude autofluorescence, MDCKII cells were treated with compounds **IC‐1** to **IC‐11** in order to determine intracellular fluorescence in the absence of the substrate dye. As shown in Figures S135 and S136 (Supporting Information), no autofluorescence, which may lead to disturbance of the Hoechst 33342 accumulation assay, was detectable in MDCKII‐hABCG2 (Figures S135A and S136A) or MDCKII‐WT cells under the reported condititions (Figures S135B and S136B).

### 2.7 Reversal of ABCG2‐Mediated Mitoxantrone Resistance

Tumor cells with ABCG2‐mediated drug resistance are able to eliminate drugs out of the cells, resulting in their excretion to subtherapeutic levels and potential chemotherapy failure [48]. MXN, an approved anticancer agent, is used to treat different types of cancer, for example, metastatic breast cancer, and is a well‐studied ABCG2 substrate. Therefore, compounds **IC‐1** to **IC‐11** were selected to investigate their potential to reverse ABCG2‐mediated MXN resistance in hABCG2‐overexpressing MDCKII cells. Based on a WST cell proliferation assay, following the procedure described previously [25], the effective inhibitory concentration (IC_50_) of the chemotherapeutic MXN was evaluated in coadministration with the investigated compounds (1.0 µM). With successful inhibition of the ABCG2 transporter, the intracellular effective concentration of the cytotoxic drug, here MXN, is higher and leads to a reduced cell viability, being reflected by a left shift of the concentration–effect curve of MXN (Figures S137 and S138). As the parental MDCKII‐WT cells do not express the hABCG2 transporter, a higher toxicity of MXN compared to ABCG2‐transfected MDCKII‐hABCG2 cells is assumed. As reported previously [36], the IC_50_ value of MXN in MDCKII cells and MDCKII‐hABCG2 cells was determined to be 0.519 ± 0.042 and 2.649 ± 0.594 µM, respectively. Table 2 shows the IC_50_ values of MXN and MXN in cotreatment with the inhibitors **IC‐1** to **IC‐11** in MDCKII‐hABCG2 cells. Corresponding concentration–effect curves are given in the Supporting Information (Figures S137 and S138). All investigated compounds were able to fully reverse the ABCG2‐induced MDR at 1.0 µM.

**TABLE 2:** Left‐shift factor calculated as IC_50_ MXN/IC_50_ MXN in combination with *N*‐carboranyl isoquinazoline derivatives **IC‐1** to **IC‐11** determined in MDCKII‐hABCG2 cells using WST‐1 assay.

Treatment of MDCKII‐hABCG2	IC_50_, µM	Left‐shift factor	Comparison to MXN
MXN	2.572 ± 0.600		
MXN + 1.0 µM **IC‐1**	0.420 ± 0.044	4.3‐fold	###
MXN + 1.0 µM **IC‐2**	0.396 ± 0.075	4.2‐fold	###
MXN + 1.0 µM **IC‐3**	0.362 ± 0.044	4.9‐fold	###
MXN + 1.0 µM **IC‐4**	0.245 ± 0.031	7.1‐fold	###
MXN + 1.0 µM **IC‐5**	0.296 ± 0.025	6.1‐fold	###
MXN + 1.0 µM **IC‐6**	0.250 ± 0.032	7.0‐fold	###
MXN + 1.0 µM **IC‐7**	0.722 ± 0.072	2.5‐fold	#
MXN + 1.0 µM **IC‐8**	0.668 ± 0.033	2.8‐fold	##
MXN + 1.0 µM **IC‐9**	0.373 ± 0.049	4.7‐fold	###
MXN + 1.0 µM **IC‐10**	0.218 ± 0.025	8.1‐fold	###
MXN + 1.0 µM **IC‐11**	0.241 ± 0.034	7.2‐fold	###

*Note:* IC_50_ values are given as mean ± SEM (*N* = 3, two‐way ANOVA, # represents significant difference in comparison to single MXN treatment, ###*p* ≤ 0.001, ##*p* ≤ 0.01, #*p* ≤ 0.05).

In particular, the tested compounds with no substituent or the unsubstituted phenyl ring in the four‐position (**IC‐1**, **IC‐2**, **IC‐3**, **IC‐7**, **IC‐8**, and **IC‐9**) yielded the lowest sensitization of the cells toward MXN for both scaffolds (*N*‐carboranyl isoquinolinone and *N*‐carboranyl 6,7‐dimethoxyisoquinolinone). Furthermore, it was evident that 4‐methoxy functionalization of the phenyl substituent (**IC‐4** and **IC‐10**) resulted in enhanced efficacy over the derivatives with 3‐methoxy substitution (**IC‐3** and **IC‐9**). The 3,4‐dimethoxy‐substituted compounds **IC‐5** and **IC‐11** caused a significant left shift of the IC_50_ value of about 6.1‐ and 7.2‐fold, respectively. Moreover, the 3,4,5‐methoxylated compound **IC‐6** induced a strong 7.0‐fold left shift in MDCKII‐hABCG2 cells. Altogether, all investigated compounds showing an ABCG2 inhibition in the Hoechst 33342 accumulation assay were able to significantly reverse MXN resistance in the MDCKII cell model. Especially 4‐methoxyphenyl (**IC‐4**, **IC‐10**), 3,4‐dimethoxyphenyl (**IC‐5**, **IC‐11**), and 3,4,5‐dimethoxyphenyl (**IC‐6**) substitution led to the strongest reversal of MXN resistance. In line with the Hoechst study, the data suggest a stronger MXN left shift caused by unsubstituted *N*‐carboranyl isoquinoline derivatives (**IC‐1** to **IC‐3**) compared to their *N*‐carboranyl 6,7‐dimethoxyisoquinolinone analogs (**IC‐7** to **IC‐9**). In addition, a higher number of methoxy groups (**IC‐4** to **IC‐6**; **IC‐9** to **IC‐11**) enhanced the reversal of MXN resistance.

### 2.8 Molecular Docking Simulations and Investigation of Mode of Inhibition

The benzamide moiety appears to be a recurring motif in different reported ABCG2 inhibitor and substrate structures [16, 26]. In order to investigate whether the increase of the molecule's rigidity by transforming the benzamide into an isoquinolinone framework is beneficial for ABCG2 inhibition, we employed docking simulations on the tested compounds **IC‐1** to **IC‐11** in comparison with the flexible structure *N*‐(*meta*‐carboran‐9‐yl)benzamide. As a protein, the cryo‐EM structure of the hABCG2 (PDB code 5NJ3) was employed, and nondynamic docking studies were performed [49]. Docking of Hoechst 33342 and MXN was used to evaluate the carboranyl derivatives’ binding behavior. Similar ligand–protein interactions within the binding site might suggest competitive inhibition between inhibitor and substrate. The obtained binding free energy values (Table S2) and putative binding modes suggest stronger interactions between the isoquinolinones and the protein compared to its flexible equivalent, *N*‐(*meta*‐carboran‐9‐yl)benzamide. Consequently, the top‐ranked poses of MXN in the alleged binding cavity of ABCG2 overlaid with the most prevalent predicted docking modes of all investigated structures (Figure S139). This observation is in agreement with the literature as well as the cocrystallized structure of MXN coupled to ABCG2 (PDB code 6VXI) [25, 50]. The observed results suggest a competitive interaction between inhibitor and substrate (Hoechst 33342 and MXN) to be the reason for the transporter protein's inhibition.

However, among the examined compounds no significant differences in protein–substrate interactions and structural orientation within the binding sites were obtained to support the biological findings. Thus, further details on the assessed molecular docking simulations are reported in the Supporting Information (Supplementary Text S1, Figure S139, Table S2).

## 3 Conclusion

The synthesis, characterization, and biological evaluation of a small library of eleven *N*‐carboranyl isoquinolinones were presented, combining an isoquinolinone structure with the 3D inorganic carborane cluster moiety. The results of the biological studies on cytotoxicity, ABCG2 inhibition and the ability to reverse ABCG2‐mediated MDR were discussed. Computational methods were added to ascertain an explanation of affinity differences.

Among the biologically examined structures, low solubility was observed for several compounds, and thus leading to a decreased concentration range of the cytotoxicity assessment in the WST‐1 cell proliferation assay. The lowest solubility was observed for **IC‐9** (5 mM). With the exception of **IC‐7** and **IC‐9**, no IC_50_ values below 25 µM in MDCKII‐hABCG2 and their parental MDCKII cells were obtained. **IC‐7** and **IC‐9** were less soluble than their unsubstituted analogous (**IC‐1** and **IC‐3**). While **IC‐1** and **IC‐2** exhibited stronger ABCG2 inhibition and reversal of MXN resistance, all other unsubstituted *N*‐carboranyl isoquinolinones (**IC‐2** to **IC‐6**) exhibit similar properties in ABCG2 inhibition and MXN reversal at 1.0 µM concentration in comparison to their 6,7‐dimethoxy‐substituted analogs (**IC‐7** to **IC‐11**). Furthermore, a higher number of methyl groups on the phenyl ring in position four of the isoquinolinone scaffold increased ABCG2 inhibition and improved the sensitivity of MDCKII cells toward MXN. The in silico binding studies mostly exhibited affinities of the tested molecules to the inner (S1) cavity, with mainly π–π stacking and strong hydrophobic interactions with hydrophobic amino acid residues of the protein. Compared to the investigated chemotherapeutic agent MXN, the inhibitors **IC‐1** to **IC‐11**, apart from **IC‐3**, showed overlaying binding modes, suggesting a competitive inhibition mechanism, but no significant differences were ascertained to explain biological findings. Thus, the *N*‐carboranyl isoquinolinone motif seems to be an interesting novel scaffold for further investigations on reversing ABCG2‐mediated MDR.

## 4 Experimental Section

### 4.1 Chemistry: Materials

All solvents were degassed, dried, and purified with the solvent purification system SPS‐800 by MBraun and stored over activated 4 Å molecular sieves. All reactions were carried out under a nitrogen or argon atmosphere using standard Schlenk techniques and anhydrous, degassed solvents, unless otherwise stated. Deuterated solvents (CDCl_3_ and DMSO‐*d*_6_) were dried over P_2_O_5_, vacuum‐transferred and degassed by freeze–pump–thaw cycling. Closo‐9‐bromo‐*meta*‐carborane [46] and (methanesulfonato‐*κ*O)[2′‐(methylamino)‐2‐biphenylyl]palladium‐dicyclohexyl(2′,6′‐dimethoxy‐2‐biphenylyl)phosphine (SPhos Pd G4) were prepared according to previously published protocols [51]. All other starting materials, reagents, and metal salts are commercially available and were used without further purification. NMR spectra were recorded on a BRUKER Avance III HD 400 MHz NMR spectrometer at 25°C (^1^H, ^11^B, ^13^C, 2D (^1^H–^1^H COSY, ^1^H–^13^C HSQC, and ^1^H–^13^C HMBC)). The chemical shifts *δ* are reported in parts per million (ppm). Tetramethylsilane (TMS) or solvent residual peaks were used as the internal reference in ^1^H and ^13^C NMR spectra, all other nuclei spectra were referenced to TMS using the Ξ scale [52]. The numbering scheme for ^1^H and ^13^C NMR signals is presented for each compound in the Supporting Information (Figures S1–S105). Electrospray ionization mass spectrometry was carried out with an ESI‐qTOF Impact II by Bruker Daltonics GmbH in positive mode. IR spectra were obtained with an FTIR spectrometer Nicolet iS5 (ATR, transmission, Thermo Scientific) scanning between 4000–400 cm^−1^ with a KBr beam splitter (only selected frequencies are given). Elemental analyses (C, H, and N) were performed with a Heraeus VARIO EL microanalyzer. The melting points were determined in glass capillaries using a Gallenkamp apparatus and are uncorrected. Column chromatography was performed using a Biotage Isolera ONE and KP‐SIL columns. All NMR and mass spectra are shown in the Supporting Information (Figures S1–S127).

Data for X‐ray structures for compounds **IC‐1**, **IC‐2**, **IC‐4**, **IC‐8,** and **IC‐10** were collected on a Gemini diffractometer (Rigaku Oxford Diffraction) [53] using Mo‐Kα radiation and ω‐scan rotation. Data reduction was performed with CrysAlisPro [53], including the program SCALE3 ABSPACK for empirical absorption correction. All structures were solved by dual space methods with SHELXT [54], and the refinement was performed with SHELXL [55]. All hydrogen atoms were located on difference Fourier maps were calculated at the final stage of the structure refinement, and carborane carbon atoms could be localized for all structures with bond length and displacement parameter analysis. Structure figures were generated with DIAMOND‐4 [56]. Deposition numbers CCDC 2468442 (**IC‐1**), 2468443 (**IC‐2**), 2468444 (**IC‐4**), 2468445 (**IC‐8**), and 2468446 (**IC‐10**) contain the supplementary crystallographic data for this publication. These data are provided free of charge by the joint Cambridge Crystallographic Data Centre and Fachinformationszentrum Karlsruhe Access Structures service and can be found under https://summary.ccdc.cam.ac.uk/structure‐summary‐form. Deposition Number(s) https://www.ccdc.cam.ac.uk/services/structures?id=doi:10.1002/cmdc.202500708 2468442 (for IC‐1), 2468443 (for IC‐2), 2468444 (for IC‐4), 2468445 (for IC‐8), 2468446 (for IC‐10) contain(s) the supplementary crystallographic data for this paper. These data are provided free of charge by the joint Cambridge Crystallographic Data Centre and Fachinformationszentrum Karlsruhe http://www.ccdc.cam.ac.uk/structures Access Structures service). These data can be obtained free of charge via https://summary.ccdc.cam.ac.uk/structure‐summary‐form (or from the Cambridge Crystallographic Data Centre, 12 Union Road, Cambridge CB2 1EZ, UK; fax: (+44)1223‐336‐033; or deposit@ccdc.cam.uk).

#### 4.1.1 Synthesis of 6,7‐Dimethoxyisoquinolin‐1(2*H*)‐One (2) [57]

In a Schlenk flask under an argon atmosphere, 0.321 g (1.23 mmol, 1.00 equiv.) of 2‐bromo‐6,7‐dimethoxybenzamide (commercially available) (**3**), 0.071 g (0.061 mmol, 5 mol%) of [Pd(PPh_3_)_4_], and 0.370 g (1.87 mmol, 1.50 equiv.) of boronic acid ester were placed. The solids were dissolved in dry 1,4‐dioxane (10 mL) and stirred for 15 min at rt. To the stirred mixture, 1.25 g (3.82 mmol, 3.00 equiv.) of Cs_2_CO_3_ in 2.5 mL of degassed water was added and stirred at 75°C for 19 h. At 0°C, 2.0 mL TFA were added, and the reaction solution was stirred for 4.5 h at rt. A saturated NH_4_Cl solution (15 mL) was added, and the mixture was extracted with EtOAc (3 × 30 mL). The combined organic phases were washed with brine (30 mL), dried over MgSO_4_ and concentrated. The resulting yellow–orange solid was washed several times with a small amount of DCM to give compound 2 (238 mg, 95%) as a colorless solid. *R*_f_ = 0.39 (DCM/MeOH, 20:1, *v/v*). ^1^H NMR (400 MHz, DMSO‐*d*_6_): *δ* [ppm] = 11.07 (s, 1H, NH), 7.55 (s, 1H, H8), 7.14 (s, 1H, H5), 7.05 (dd, ^3^ *J*_HH_ = 7.3 Hz, ^3^ *J*_HH_ = 3.4 Hz, 1H, H3), 6.46 (d, ^3^ *J*_HH_ = 7.1 Hz, 1H, H4), 3.87 (s, 3H, OCH_3_), 3.85 (s, 3H, OCH_3_). HR‐MS (ESI(+), acetonitrile): *m/z* calc. [C_11_H_11_NO_3_] ([M + H]^+^): 206.0817, found: 206.0827.

#### 4.1.2 Synthesis of 4‐Bromoisoquinolin‐1(2*H*)‐One (4) [44]

NBS (1.23 g, 6.86 mmol) was added to isoquinolin‐1(2*H*)‐one (1.00 g, 6.87 mmol) in DMF (30 mL). The reaction mixture was stirred for 3 h at rt, poured into water (100 mL), and the resulting precipitate was filtered off. The crude product was washed with cold water and dried overnight in a desiccator under vacuum to obtain 4 as an off‐white solid (1.26 g, 82%). ^1^H NMR (400 MHz, DMSO‐*d*_6_): *δ* [ppm] = 11.57 (s, 1H, NH), 8.23 (dd, ^3^ *J*_HH_ = 7.9 Hz, ^4^ *J*_HH_ = 1.4 Hz, 1H, H5), 7.86 (ddd, ^3^ *J*_HH_ = 8.3, 7.1 Hz, ^4^ *J*_HH_ = 1.4 Hz, 1H, H7), 7.76 (d, ^3^ *J*_HH_ = 8.1 Hz, 1H, H6), 7.60 (ddd, ^3^ *J*_HH_ = 8.2, 7.1 Hz, ^4^ *J*_HH_ = 1.2 Hz, 1H, H8), 7.55 (s, 1H, H3). HR‐MS (ESI(+), acetonitrile): *m/z* calc. [C_9_H_7_NOBr] ([M + H]^+^): 225.9691, found: 225.9701.

#### 4.1.3 Synthesis of 4‐Bromo‐6,7‐Dimethoxyisoquinolin‐1(2*H*)‐One (5) [45]

Isoquinolinone 2 (0.410 g, 2.00 mmol, 1.00 equiv.) was dissolved in glacial acetic acid (3.4 mL); then bromine (51.0 μL) in glacial acetic acid (1.5 mL) was added dropwise with vigorous stirring. After 1 h, 46.6 μL of bromine, dissolved in 1.5 mL of glacial acetic acid, was added. The mixture was stirred for 3.5 h at rt, ice water (50 mL) was added, and the mixture was extracted with DCM (3 × 100 mL). The combined organic phases were dried over anhydr. Na_2_SO_4_, and then the solvent was removed under reduced pressure. Compound 5 (0.379 g, 76%) was obtained as a red–brown solid. *R*_f_ (EtOAc:*n*‐hexane 8:2) = 0.41. ^1^H NMR (400 MHz, DMSO‐*d*_6_): *δ* [ppm] = 11.44 (s, 1H, NH), 7.62 (s, 1H, H8), 7.44 (s, 1H, H3), 7.12 (s, 1H, H5), 3.94 (s, 3H, OCH_3_), 3.89 (s, 3H, OCH_3_). HR‐MS (ESI(+), acetonitrile): *m/z* calc. [C_11_H_10_NO_3_Br] ([M + H]^+^): 283.9922, found: 283.9940.

#### 4.1.4 General Procedure for the Preparation of 4‐Arylisoquinolin‐1(2*H*)‐Ones 6a–e and 7a–d

4‐Bromoisoquinolin‐1(2*H*)‐one (0.250 g, 1.12 mmol, 1.0 equiv.), [Pd(PPh_3_)_4_] (0.127 g, 0.11 mmol, 0.1 equiv.), Cs_2_CO_3_ (1.45 g, 4.46 mmol, 4.0 equiv.), and the appropriate boronic acid (1.5 equiv.) were placed in an Ar‐flushed Schlenk flask. Degassed H_2_O (10 mL) and dry DME (30 mL) were added, and the mixture was heated to reflux overnight. The mixture was cooled to rt, water (50 mL) was added, and the reaction mixture was extracted with EtOAc (3 × 70 mL). The organic phase was washed with brine, dried over MgSO_4_ and filtered through a plug of Celite:silica (1:1, 100% EtOAc). Solvents were removed, and the crude product was washed with minimum amounts of cold diethyl ether or purified on silica gel (eluent: *n*‐hexane/EtOAc) to obtain the product as a colorless to red‐brownish solid.

4‐Phenylisoquinolin‐1(2*H*)‐one (6a) was obtained from 4‐bromoisoquinolin‐1(2*H*)‐one and phenyl boronic acid as a colorless solid in 86% yield (213 mg) according to the general procedure described above. ^1^H NMR (400 MHz, DMSO‐*d*_6_): *δ* [ppm] = 11.46 (s, 1H, NH), 8.30 (dd, ^3^ *J*_HH_ = 8.0 Hz, ^4^ *J*_HH_ = 1.4 Hz, 1H, H5), 7.70 (ddd, ^3^ *J*_HH_ = 8.4, 7.1 Hz, ^4^ *J*_HH_ = 1.5 Hz, 1H, H7), 7.57–7.47 (m, 4H, H_arom_), 7.45–7.41 (m, 3H, H_arom_), 7.10 (d, ^3^ *J*_HH_ = 4.4 Hz, 1H, H3). ^13^C{^1^H} NMR (101 MHz, DMSO‐*d*_6_): *δ* [ppm] = 161.3 (1C, C1), 136.6 (1C, C_arom_), 136.2 (1C, C_arom_), 132.4 (1C, C7), 129.7 (2C, C_arom_), 128.7 (2C, C_arom_), 127.8 (1C, C3), 127.4 (1C, C_arom_), 127.2 (1C, C5), 126.5 (1C, C6), 125.9 (1C, C_arom_), 124.2 (1C, C_arom_), 117.4 (1C, C_arom_). HR‐MS (ESI(+), acetonitrile): *m/z* calc. [C_15_H_11_NO] ([M + H]^+^): 222.0919, found: 222.0938. IR (KBr): $\tilde{v}$ [cm^−1^] = 3035 (m), 2922 (w), 2603 (m), 2359 (w), 1650 (s), 1619 (s), 1599 (s), 1505 (s), 1458 (s), 1272 (s), 1240 (s), 1178 (s), 1154 (s), 1113 (s), 1069 (s), 1026 (s), 1005 (s), 874 (s), 771 (s), 761 (s), 697 (s), 591 (s), 542 (s), 521 (s), 487 (s), 474 (s), 466 (s), 458 (s), 424 (s).

4‐(3‐Methoxyphenyl)isoquinolin‐1(2*H*)‐one (6b) was obtained from 4‐bromoisoquinolin‐1(2*H*)‐one and 3‐methoxyphenyl boronic acid as a colorless to off‐white solid in 73% yield (205 mg) according to the general procedure described above. ^1^H NMR (400 MHz, DMSO‐*d*_6_): *δ* [ppm] = 11.46 (d, ^3^ *J*_HH_ = 5.0 Hz, 1H, NH), 8.32–8.28 (m, 1H, H5), 7.71 (ddd, ^3^ *J*_HH_ = 8.3, 6.9 Hz, ^4^ *J*_HH_ = 1.5 Hz, 1H, H7), 7.57–7.51 (m, 2H, H6, H8), 7.41 (t, ^3^ *J*_HH_ = 7.9 Hz, 1H, H5′), 7.12 (d, ^3^ *J*_HH_ = 5.5 Hz, 1H, H3), 7.01 (d, ^4^ *J*_HH_ = 2.1 Hz, 1H, H2′), 6.98 (td, ^3^ *J*_HH_ = 3.5, 1.6 Hz, 2H, H4′, H6′), 3.80 (s, 3H, OCH_3_). ^13^C{^1^H} NMR (101 MHz, DMSO‐*d*_6_): *δ* [ppm] = 161.3 (1C, C1), 159.4 (1C, C3′), 137.6 (1C, C1′), 136.6 (1C, C8a), 132.4 (1C, C7), 129.6 (1C, C5′), 127.8 (1C, C3), 127.2 (1C, C5), 126.5 (1C, C6), 125.9 (1C, C5a), 124.3 (1C, C8), 122.0 (1C, C2′), 117.3 (1C, C1), 115.2 (1C, C_arom_), 113.0 (1C, C_arom_), 55.1 (1C, OCH_3_). HR‐MS (ESI(+), acetonitrile): *m/z* calc. [C_16_H_13_NO_2_] ([M + H]^+^): 252.1025, found: 252.1026. IR (KBr): $\tilde{v}$ [cm^−1^] = 2832 (m), 1634 (s), 1606 (s), 1504 (s), 1464 (s), 1439 (s), 1251 (s), 1215 (s), 1179 (s), 1134 (s), 1027 (s), 855 (s), 816 (s), 785 (s), 764 (s), 743 (s), 707 (s), 690 (s), 597 (s), 569 (s), 519 (s), 464 (s).

4‐(4‐Methoxyphenyl)isoquinolin‐1(2*H*)‐one (6c) was obtained from 4‐bromoisoquinolin‐1(2*H*)‐one and 4‐methoxyphenyl boronic acid as a light‐yellow solid in 89% yield (281 mg) according to the general procedure described above. ^1^H NMR (400 MHz, DMSO‐*d*_6_): *δ* [ppm] = 11.40 (s, 1H, NH), 8.29 (dd, ^3^ *J*_HH_ = 8.0 Hz, ^4^ *J*_HH_ = 1.5 Hz, 1H, H5), 7.69 (ddd, ^3^ *J*_HH_ = 8.4, 7.1 Hz, ^4^ *J*_HH_ = 1.6 Hz, 1H, H7), 7.55–7.47 (m, 2H, H6, H8), 7.36–7.32 (m, 2H, H2′), 7.05 (d, ^3^ *J*_HH_ = 7.8 Hz, 3H, H3, H3′), 3.81 (s, 3H, OCH_3_). ^13^C{^1^H} NMR (101 MHz, DMSO‐*d*_6_): *δ* [ppm] = 161.2 (1C, C1), 158.6 (1C, C4′), 137.0 (1C, C8a), 132.4 (1C, C7), 130.9 (2C, C2′), 128.3 (1C, C1′), 127.4 (1C, C5), 127.2 (1C, C3), 126.4 (1C, C6), 125.9 (1C, C5a), 124.3 (1C, C8), 117.0 (1C, C4), 114.1 (2C, C3′), 55.2 (1C, OCH_3_). HR‐MS (ESI(+), acetonitrile): *m/z* calc. [C_16_H_13_NO_2_] ([M + H]^+^): 252.1025, found: 252.1018. IR (KBr): $\tilde{v}$ [cm^−1^] = 2833 (m), 1655 (s), 1636 (s), 1604 (s), 1515 (s), 1499 (s), 1472 (s), 1238 (s), 1219 (s), 1172 (s), 1134 (s), 1103 (s), 1023 (s), 923 (s), 884 (s), 839 (s), 820 (s), 802 (s), 786 (s), 767 (s), 685 (s), 670 (s), 570 (s), 540 (s), 522 (s), 498 (s).

4‐(3,4‐Dimethoxyphenyl)isoquinolin‐1(2*H*)‐one (6d) was obtained from 4‐bromoisoquinolin‐1(2*H*)‐one and 3,4‐dimethoxyphenyl boronic acid as a light‐yellow solid in 78% yield (245 mg) according to the general procedure described above. ^1^H NMR (400 MHz, DMSO‐*d*_6_): *δ* [ppm] = 11.41 (d, ^3^ *J*_HH_ = 5.3 Hz, 1H, NH), 8.29 (dd, ^3^ *J*_HH_ = 8.0 Hz, ^4^ *J*_HH_ = 1.4 Hz, 1H, H5), 7.73–7.67 (m, 1H, H7), 7.58–7.50 (m, 2H, H6, H8), 7.09–7.04 (m, 2H, H2′, H6′), 6.98 (d, ^3^ *J*_HH_ = 2.0 Hz, 1H, H3), 6.93 (dd, ^3^ *J*_HH_ = 8.2 Hz, ^4^ *J*_HH_ = 2.0 Hz, 1H, H5′), 3.81 (s, 3H, OCH_3_), 3.78 (s, 3H, OCH_3_). ^13^C{^1^H} NMR (101 MHz, DMSO‐*d*_6_): *δ* [ppm] = 161.2 (1C, C1), 148.7 (1C, C4′), 148.2 (1C, C3′), 137.0 (1C, C8a), 132.4 (1C, C7), 128.6 (1C, C1′), 127.5 (1C, C6′), 127.2 (1C, C5), 126.4 (1C, C6), 125.9 (1C, C5a), 124.5 (1C, C8), 121.9 (1C, C5′), 117.3 (1C, C4), 113.6 (1C, C3), 111.9 (1C, C2′), 55.6 (1C, OCH_3_), 55.5 (1C, OCH_3_). HR‐MS (ESI(+), acetonitrile): *m/z* calc. [C_17_H_15_NO_3_] ([M + H]^+^): 282.1130, found: 282.1136. IR (KBr): $\tilde{v}$ [cm^−1^] = 2835 (m), 1661 (s), 1636 (s), 1603 (s), 1517 (s), 1499 (s), 1471 (s), 1444 (s), 1330 (s), 1251 (s), 1220 (s), 1174 (s), 1139 (s), 1022 (s), 876 (s), 825 (s), 787 (s), 776 (s), 739 (s), 691 (s), 597 (s), 572 (s), 523 (s), 482 (s), 466 (s).

4‐(3,4,5‐Trimethoxyphenyl)isoquinolin‐1(2*H*)‐one (6e) was obtained from 4‐bromoisoquinolin‐1(2*H*)‐one and 3,4,5‐trimethoxyphenyl boronic acid as an off‐white solid in 71% yield (247 mg) according to the general procedure described above. ^1^H NMR (400 MHz, DMSO‐*d*_6_): *δ* [ppm] = 11.45 (s, 1H, NH), 8.29 (d, ^3^ *J*_HH_ = 7.9 Hz, 1H, H5), 7.74–7.68 (m, 1H, H7), 7.62 (d, ^3^ *J*_HH_ = 8.1 Hz, 1H, H8), 7.53 (t, ^3^ *J*_HH_ = 7.5 Hz, 1H, H6), 7.13 (s, 1H, H3), 6.70 (s, 2H, H2′), 3.80 (s, 6H, OCH_3_), 3.72 (s, 3H, OCH_3_). ^13^C{^1^H} NMR (101 MHz, DMSO‐*d*_6_): *δ* [ppm] = 161.3 (1C, C1), 152.9 (2C, C3′), 136.8 (1C, C4′), 136.7 (1C, C8a), 132.5 (1C, C7), 131.8 (1C, C1′), 127.7 (1C, C3), 127.2 (1C, C5), 126.4 (1C, C6), 125.8 (1C, C5a), 124.5 (1C, C8), 117.5 (1C, C4), 107.2 (1C, C2′), 60.0 (1C, OCH_3_), 56.0 (2C, OCH_3_). MS (ESI(+), acetonitrile): *m/z* calc. [C_18_H_17_NO_4_] ([M + H]^+^): 312.1236, found: 312.1242. IR (KBr): $\tilde{v}$ [cm^−1^] = 3160 (w), 2834 (m), 2362 (w), 1634 (s), 1604 (s), 1577 (s), 1546 (s), 1497 (s), 1464 (s), 1440 (s), 1412 (s), 1375 (s), 1332 (s), 1235 (s), 1216 (s), 1178 (s), 1111 (s), 1073 (s), 1028 (s), 1000 (s), 900 (s), 854 (s), 830 (s), 786 (s), 771 (s), 741 (s), 707 (s), 689 (s), 669 (s), 625 (s), 596 (s), 573 (s), 520 (s), 500 (s), 482 (s), 464 (s).

6,7‐Dimethoxy‐4‐phenylisoquinolin‐1(2*H*)‐one (7a) was obtained from 4‐bromo‐6,7‐dimethoxyisoquinolin‐1(2*H*)‐one and phenyl boronic acid as a light‐yellow solid in 59% yield (185 mg) according to the general procedure described above. ^1^H NMR (400 MHz, DMSO‐*d*_6_): *δ* [ppm] = 11.33 (d, ^3^ *J*_HH_ = 5.4 Hz, 1H, NH), 7.68 (s, 1H, H5), 7.53–7.44 (m, 5H, H_arom_), 6.98 (d, ^3^ *J*_HH_ = 5.4 Hz, 1H, H3), 6.94 (s, 1H, H8), 3.89 (s, 3H, OCH_3_), 3.71 (s, 3H, OCH_3_). ^13^C{^1^H} NMR (101 MHz, DMSO‐*d*_6_): *δ* [ppm] = 160.6 (1C, C1), 152.9 (1C, C6), 148.5 (1C, C7), 136.6 (1C, C1′), 134.5 (1C, C8a), 131.6 (1C, C_arom_), 129.5 (1C, C_arom_), 128.7 (1C, C_arom_), 128.3 (1C, C_arom_), 127.3 (1C, C_arom_), 126.3 (1C, C3), 119.7 (1C, C4), 117.1 (1C, C5a), 107.4 (1C, C5), 104.9 (1C, C8), 55.5 (1C, OCH_3_), 55.3 (1C, OCH_3_). HR‐MS (ESI(+), acetonitrile): *m/z* calc. [C_17_H_15_NO_3_] ([M + H]^+^): 282.1130, found: 282.1140. IR (KBr): $\tilde{v}$ [cm^−1^] = 2832 (m), 1635 (s), 1608 (s), 1505 (s), 1464 (s), 1439 (s), 1389 (m), 1328 (s), 1253 (s), 1215 (s), 1180 (s), 1134 (s), 1110 (m), 1071 (m), 1027 (s), 992 (m), 908 (m), 883 (s), 856 (s), 848 (s), 818 (m), 785 (m), 764 (m), 743 (m), 707 (m), 690 (s), 597 (m), 568 (s), 521 (s), 462 (m).

6,7‐Dimethoxy‐4‐(3‐methoxyphenyl)isoquinolin‐1(2*H*)‐one (7b) was obtained from 4‐bromo‐isoquinolin‐1(2*H*)‐one and 3‐methoxyphenyl boronic acid as an off‐white solid in 53% yield (184 mg) according to the general procedure described above. *R*_f_ = 0.32 (*n*‐hexane/EtOAc, 1:1, *v/v*). ^1^H NMR (400 MHz, DMSO‐*d*_6_): *δ* [ppm] = 11.33 (s, 1H, NH), 7.68 (s, 1H, H5), 7.39 (t, ^3^ *J*_HH_ = 7.9 Hz, 1H, H5′), 7.05–6.95 (m, 5H, H_arom_), 3.88 (s, 3H, OCH_3_), 3.80 (s, 3H, OCH_3_), 3.72 (s, 3H, OCH_3_). ^13^C{^1^H} NMR (101 MHz, DMSO‐*d*_6_): *δ* [ppm] = 160.6 (1C, C1), 159.5 (1C, C3′), 152.9 (1C, C_q_), 148.6 (1C, C_q_), 138.0 (1C, C_q_), 131.5 (1C, C_q_), 129.7 (1C, C5′), 126.4 (1C, C_arom_H), 121.7 (1C, C_arom_H), 119.7 (1C, C_q_), 117.0 (1C, C_q_), 114.7 (1C, C_arom_H), 113.2 (1C, C_arom_H), 107.4 (1C, C5), 104.9 (1C, CH), 55.6 (1C, OCH_3_), 55.4 (1C, OCH_3_), 55.1 (1C, OCH_3_). HR‐MS (ESI(+), acetonitrile): *m/z* calc. [C_18_H_17_NO_4_] ([M + H]^+^): 312.1236, found: 312.1234. IR (KBr): $\tilde{v}$ [cm^−1^] = 2902 (m), 2362 (w), 1635 (s), 1607 (s), 1507 (s), 1464 (s), 1438 (s), 1386 (s), 1329 (s), 1270 (s), 1248 (s), 1213 (s), 1178 (s), 1134 (s), 1074 (s), 1025 (s), 997 (s), 906 (s), 854 (s), 818 (s), 786 (s), 772 (s), 742 (s), 707 (s), 690 (s), 596 (s), 572 (s), 520 (s), 499 (s), 463 (s).

6,7‐Dimethoxy‐4‐(4‐methoxyphenyl)isoquinolin‐1(2*H*)‐one (7c) was obtained from 4‐bromo‐isoquinolin‐1(2*H*)‐one and 4‐methoxyphenyl boronic acid as a light‐yellow solid in 59% yield (205 mg) according to the general procedure described above. *R*_f_ = 0.29 (*n*‐hexane/EtOAc, 1:1, *v/v*). ^1^H NMR (400 MHz, DMSO‐*d*_6_): *δ* [ppm] = 11.27 (s, 1H, NH), 7.67 (s, 1H, H5), 7.38 (d, ^3^ *J*_HH_ = 8.1 Hz, 2H, H2′), 7.04 (d, ^3^ *J*_HH_ = 8.1 Hz, 2H, H3′), 6.92 (s, 2H, H3, H8), 3.88 (s, 3H, OCH_3_), 3.81 (s, 3H, OCH_3_), 3.72 (s, 3H, OCH_3_). ^13^C{^1^H} NMR (101 MHz, DMSO‐*d*_6_): *δ* [ppm] = 160.6 (1C, C1), 158.5 (1C, C4′), 152.8 (1C, C6), 148.5 (1C, C7), 132.0 (1C, C1′), 130.6 (2C, C2′), 128.7 (1C, C8a), 125.9 (1C, C3), 119.7 (1C, C5a), 116.8 (1C, C4), 114.1 (2C, C3′), 107.4 (1C, C5), 105.0 (1C, C8), 55.5 (1C, OCH_3_), 55.4 (1C, OCH_3_), 55.1 (1C, OCH_3_). MS (ESI(+), acetonitrile): *m/z* calc. [C_18_H_17_NO_4_] ([M + H]^+^): 312.1236, found: 312.1241. IR (KBr): $\tilde{v}$ [cm^−1^] = 2902 (m), 2362 (w), 1635 (s), 1607 (s), 1507 (s), 1464 (s), 1438 (s), 1386 (s), 1329 (s), 1270 (s), 1248 (s), 1213 (s), 1178 (s), 1134 (s), 1074 (s), 1025 (s), 997 (s), 906 (s), 854 (s), 818 (s), 786 (s), 772 (s), 742 (s), 707 (s), 690 (s), 596 (s), 572 (s), 520 (s), 499 (s), 463 (s).

6,7‐Dimethoxy‐4‐(3,4‐dimethoxyphenyl)isoquinolin‐1(2*H*)‐one (7d) was obtained from 4‐bromo‐isoquinolin‐1(2*H*)‐one and 3,4‐dimethoxyphenyl boronic acid as a red–orange solid in 34% yield (130 mg) according to the general procedure described above. *R*_f_ = 0.21 (*n*‐hexane/EtOAc, 1:1, *v/v*). ^1^H NMR (400 MHz, DMSO‐*d*_6_): *δ* [ppm] = 11.28 (d, ^3^ *J*_HH_ = 5.6 Hz, 1H, NH), 7.67 (s, 1H, H5), 7.07–7.01 (m, 3H, H8, H2′, H5′), 7.00–6.95 (m, 2H, H3, H6′), 3.88 (s, 3H, OCH_3_), 3.80 (s, 3H, OCH_3_), 3.79 (s, 3H, OCH_3_), 3.73 (s, 3H, OCH_3_). ^13^C{^1^H} NMR (101 MHz, DMSO‐*d*_6_): *δ* [ppm] = 160.6 (1C, C1), 152.8 (1C, C4′), 148.7 (1C, C3′), 148.5 (1C, C6), 148.1 (1C, C7), 131.9 (1C, C1′), 129.0 (1C, C8a), 126.0 (1C, C3), 121.5 (1C, C6′), 119.6 (1C, C4), 117.0 (1C, C5a), 113.2 (1C, C2′), 112.0 (1C, C5′), 107.4 (1C, C5), 105.1 (1C, C8), 55.6 (2C, OCH_3_), 55.5 (1C, OCH_3_), 55.4 (1C, OCH_3_). HR‐MS (ESI(+), acetonitrile): *m/z* calc. [C_19_H_19_NO_5_] ([M + H]^+^): 342.1341, found: 342.1341. IR (KBr): $\tilde{v}$ [cm^−1^] = 3137 (w), 2893 (m), 2831 (m), 2359 (w), 1636 (s), 1610 (s), 1505 (s), 1481 (s), 1462 (s), 1437 (s), 1388 (s), 1266 (s), 1245 (s), 1225 (s), 1212 (s), 1173 (s), 1135 (s), 1114 (s), 1073 (s), 1029 (s), 1009 (s), 906 (s), 873 (s), 850 (s), 814 (s), 786 (s), 765 (s), 744 (s), 681 (s), 624 (s), 584 (s), 547 (s), 486 (s).

#### 4.1.5 General S version synthesis for *N*‐Carboranyl 4‐Isoquinolin‐1(2*H*)‐Ones IC‐1 to IC‐11

An oven‐dried Schlenk flask was charged with the appropriate isoquinolinone (1.00 mmol), *closo*‐9‐bromo‐1,7‐dicarbadodecaborane (12) (1.50 mmol), SPhos (5 mol%), SPhos Pd G4 (5 mol%), and potassium tert‐butoxide (2.00 mmol), and the flask was evacuated three times and backfilled with argon. 1,4‐Dioxane (2 mL) was added, and the mixture was heated to reflux overnight. After cooling to rt, ethyl acetate was added, and the suspension was filtered over a plug of Celite and silica. After removal of solvent, the crude product was purified by column chromatography on silica gel (CHCl_3_ or *n*‐hexane/EtOAc).

*N*‐(*closo*‐1,7‐dicarbadodecaboran(12)‐9‐yl)isoquinolin‐1(2*H*)‐one (**IC‐1**) was obtained from isoquinolin‐1(2*H*)‐one as a beige solid in 71% yield according to the general procedure described above. Single crystals were obtained by slow evaporation of a saturated acetone solution of **IC‐1**. *R*_f_ = 0.61 (CHCl_3_). Mp. = 174–176°C (*n*‐pentane). ^1^H NMR (400 MHz, CDCl_3_): *δ* [ppm] = 8.44–8.40 (m, 1H, H5), 7.59 (ddd, ^3^ *J*_HH_ = 8.1, 6.9 Hz, ^4^ *J*_HH_ = 1.3 Hz, 1H, H7), 7.47–7.42 (m, 2H, H6, H8), 7.40 (q, ^3^ *J*_HH_ = 5.5 Hz, 1H, H3), 6.43 (d, ^3^ *J*_HH_ = 7.5 Hz, 1H, H4), 3.70–1.28 (br, 9H, cluster‐BH), 2.98 (s, 2H, cluster‐CH). ^13^C{^1^H} NMR (101 MHz, CDCl_3_): *δ* [ppm] = 165.8 (1C, C1), 137.5 (1C, C8a), 134.2 (1C, C3), 132.4 (1C, C7), 128.4 (1C, C5), 126.8 (1C, C6), 125.6 (1C, C8), 106.5 (1C, C4), 52.6 (2C, cluster‐C). ^11^B{^1^H} NMR (128 MHz, CDCl_3_): *δ* [ppm] = 1.4 (s, 1B, BN), −6.7 (s, 2B, BH), −11.2 (s, 1B, BH), −13.4 (s, 2B, BH), −15.2 (s, 2B, BH), −18.3 (s, 1B, BH), −20.7 (s, 1B, BH). ^11^B‐NMR (128 MHz, CDCl_3_): *δ* [ppm] = 1.4 (s, 1B, BN), −6.7 (d, 2B, BH), −11.2 (d, 1B, BH), −12.4 to −16.6 (m, 4B, BH), −18.3 (d, 1B, BH), −20.7 (d, 1B, BH). HR‐MS (ESI(+), acetonitrile): *m/z* calc. [C_11_H_17_B_10_NO] ([M + H]^+^): 288.2392, found: 288.2408. IR (KBr): $\tilde{v}$ [cm^−1^] = 3055 (w), 3025 (m), 2922 (w), 2615 (s), 2359 (w), 1645 (s), 1617 (s), 1595 (s), 1557 (m), 1507 (m), 1489 (m), 1456 (m), 1330 (m), 1302 (s), 1275 (s), 1252 (s), 1224 (s), 1177 (s), 1149 (s), 1122 (s), 1065 (s), 1024 (s), 1004 (s), 980 (s), 894 (m), 834 (s), 775 (s), 733 (s), 692 (s), 614 (m), 577 (s), 558 (s), 508 (m), 466 (m). Elemental analysis (C_11_H_17_B_10_NO) calc.(%): C 45.97, H 5.96, N 4.87, found(%): C 45.88, H 6.07, N 4.80.

*N*‐(*closo*‐1,7‐dicarbadodecaboran(12)‐9‐yl)‐4‐phenylisoquinolin‐1(2*H*)‐one (**IC‐2**) was obtained from 4‐phenylisoquinolin‐1(2*H*)‐one as a beige solid in 74% yield according to the general procedure described above. Single crystals were obtained by slow evaporation of a saturated acetone solution of **IC‐2**. *R*_f_ = 0.15 (CHCl_3_). Mp. = 201–203°C (*n*‐pentane). ^1^H NMR (400 MHz, CDCl_3_): *δ* [ppm] = 8.52 (dd, ^3^ *J*_HH_ = 8.0 Hz, ^4^ *J*_HH_ = 1.5 Hz, 1H, H5), 7.57 (ddd, ^3^ *J*_HH_ = 8.3, 6.8 Hz, ^4^ *J*_HH_ = 1.5 Hz, 1H, H7), 7.52–7.40 (m, 7H, H6, H8, H2′, H3′, H4′), 7.36 (q, ^3^ *J*_HH_ = 2.3 Hz, 1H, H3), 3.50–1.60 (br, 9H, cluster‐BH), 2.98 (s, 2H, cluster‐CH). ^13^C{^1^H} NMR (101 MHz, CDCl_3_): *δ* [ppm] = 165.3 (1C, C1), 137.3 (1C, C_arom_), 136.8 (1C, C_arom_), 133.3 (1C, C3), 132.3 (1C, C7), 130.2 (2C, C_arom_), 128.8 (2C, C5, C_arom_), 127.6 (1C, C_arom_), 126.9 (1C, C_arom_), 124.4 (1C, C8), 52.6 (2C, cluster‐C). ^11^B{^1^H} NMR (128 MHz, CDCl_3_): *δ* [ppm] = 1.4 (s, 1B, BN), −6.6 (s, 2B, BH), −11.1 (s, 1B, BH), −13.4 (s, 2B, BH), −15.1 (s, 2B, BH), −18.3 (s, 1B, BH), −20.7 (s, 1B, BH). ^11^B‐NMR (128 MHz, CDCl_3_): *δ* [ppm] = 1.4 (s, 1B, BN), −6.0 (d, 2B, BH), −9.8 to −16.8 (m, 5B, BH), −18.3 (d, 1B, BH), −20.6 (d, 1B, BH). HR‐MS (ESI(+), acetonitrile): *m/z* calc. [C_17_H_21_B_10_NO] ([M + H]^+^): 364.2705, found: 364.2705. IR (KBr): $\tilde{v}$ [cm^−1^] = 3050 (w), 3031 (m), 2612 (s), 2357 (w), 1739 (w), 1645 (s), 1618 (s), 1597 (s), 1554 (s), 1480 (s), 1447 (s), 1338 (s), 1326 (s), 1267 (s), 1234 (s), 1180 (s), 1152 (s), 1139 (s), 1065 (s), 1029 (s), 1004 (s), 977 (s), 952 (s), 913 (m), 882 (m), 771 (s), 749 (s), 699 (s), 625 (s), 606 (s), 595 (s), 549 (s), 525 (s), 492 (s), 468 (s), 433 (s). Elemental analysis (C_17_H_21_B_10_NO) calc.(%): C 56.18, H 5.82, N 3.85, found(%): C 56.30, H 5.88, N 3.81.

*N*‐(*closo*‐1,7‐dicarbadodecaboran(12)‐9‐yl)‐4‐(3‐methoxyphenyl)isoquinolin‐1(2*H*)‐one (**IC‐3**) was obtained from 4‐(3‐methoxyphenyl)isoquinolin‐1(2*H*)‐one as a colorless solid in 79% yield according to the general procedure described above. *R*_f_ = 0.22 (*n*‐hexane/EtOAc, 85:15, *v/v*). Mp. = 206–208°C (*n*‐pentane). ^1^H NMR (400 MHz, CDCl_3_): *δ* [ppm] = 8.51 (d, ^3^ *J*_HH_ = 7.9 Hz, 1H, H7), 7.55 (td, ^3^ *J*_HH_ = 8.5, 4.0 Hz, 2H, H7, H8), 7.47 (ddd, ^3^ *J*_HH_ = 8.2, 6.4 Hz, ^4^ *J*_HH_ = 1.8 Hz, 1H, H6), 7.37 (d, ^3^ *J*_HH_ = 8.0 Hz, 2H, H3′, H6′), 7.03 (d, ^3^ *J*_HH_ = 7.6 Hz, 1H, H2′), 6.99–6.94 (m, 2H, H3, H4′), 3.86 (s, 3H, OCH_3_), 3.50–1.60 (br, 9H, cluster‐BH), 2.98 (s, 2H, cluster‐CH). ^13^C{^1^H} NMR (101 MHz, CDCl_3_): *δ* [ppm] = 165.3 (1C, C1), 159.9 (1C, C5′), 138.6 (1C, C1′), 136.7 (1C, C8a), 133.3 (1C, C6′), 132.3 (1C, C7), 129.7 (1C, C3′), 128.7 (1C, C5), 126.9 (1C, C6), 126.5 (1C, C5a), 124.4 (1C, C8), 122.6 (1C, C2′), 119.7 (1C, C4), 115.9 (1C, C3), 112.9 (1C, C4′), 55.5 (1C, OCH_3_), 52.6 (2C, cluster‐C). ^11^B{^1^H} NMR (128 MHz, CDCl_3_): *δ* [ppm] = 1.4 (s, 1B, BN), −6.7 (s, 2B, BH), −11.2 (s, 1B, BH), −13.4 (s, 2B, BH), −15.2 (s, 2B, BH), −18.3 (s, 1B, BH), −20.7 (s, 1B, BH). ^11^B‐NMR (128 MHz, CDCl_3_): *δ* [ppm] = 1.4 (s, 1B, BN), −6.7 (d, 2B, BH), −10.0 to −16.8 (m, 5B, BH), −18.3 (d, 1B, BH), −20.7 (d, 1B, BH). HR‐MS (ESI(+), acetonitrile): *m/z* calc. [C_18_H_23_B_10_NO_2_] ([M + H]^+^): 394.2810, found: 394.2818. IR (KBr): $\tilde{v}$ [cm^−1^] = 3040 (m), 2611 (s), 2359 (w), 1644 (s), 1617 (s), 1492 (s), 1466 (s), 1425 (s), 1282 (s), 1270 (s), 1229 (s), 1180 (s), 1152 (s), 1140 (s), 1063 (s), 1027 (s), 1007 (s), 784 (s), 768 (s), 738 (s), 702 (s), 690 (s), 605 (s), 546 (s), 534 (s), 493 (s), 465 (s). Elemental analysis (C_18_H_23_B_10_NO_2_) calc.(%): C 54.94, H 5.98, N 3.56, found(%): C 54.81, H 6.09, N 3.50.

*N*‐(*closo*‐1,7‐dicarbadodecaboran(12)‐9‐yl)‐4‐(4‐methoxyphenyl)isoquinolin‐1(2*H*)‐one (**IC‐4**) was obtained from 4‐(4‐methoxyphenyl)isoquinolin‐1(2*H*)‐one as an off‐white solid in 73% yield according to the general procedure described above. Single crystals were obtained by slow evaporation of a saturated CDCl_3_ solution of **IC‐4**. *R*_f_ = 0.24 (*n*‐hexane/EtOAc, 4:1, *v/v*). Mp. = 226–228°C (*n*‐pentane). ^1^H NMR (400 MHz, CDCl_3_): *δ* [ppm] = 8.51 (dd, ^3^ *J*_HH_ = 8.0 Hz, ^4^ *J*_HH_ = 1.4 Hz, 1H, H5), 7.56 (ddd, ^3^ *J*_HH_ = 8.3, 6.9 Hz, ^4^ *J*_HH_ = 1.5 Hz, 1H, H7), 7.50–7.45 (m, 2H, H6, H8), 7.35 (d, ^3^ *J*_HH_ = 8.6 Hz, 2H, H2′), 7.32 (d, ^3^ *J*_HH_ = 3.7 Hz, 1H, H3), 7.03–6.99 (m, 2H, H3′), 3.88 (s, 3H, OCH_3_), 3.50–1.60 (br, 9H, cluster‐BH), 2.98 (s, 2H, cluster‐CH). ^13^C{^1^H} NMR (101 MHz, CDCl_3_): *δ* [ppm] = 165.3 (1C, C1), 159.2 (1C, C4′), 137.1 (1C, C8a), 133.1 (1C, C3), 132.2 (1C, C7), 131.2 (2C, C2′), 129.5 (1C, C5), 128.7 (1C, C5a), 126.8 (1C, C6 or C8), 126.5 (1C, C1′), 124.5 (1C, C6 or C8), 119.4 (1C, C4), 114.2 (2C, C3′), 55.5 (1C, OCH_3_), 52.5 (2C, cluster‐C). ^11^B{^1^H} NMR (128 MHz, CDCl_3_): *δ* [ppm] = 1.4 (s, 1B, BN), −6.8 (s, 2B, BH), −11.2 (s, 1B, BH), −13.5 (s, 2B, BH), −15.2 (s, 2B, BH), −18.4 (s, 1B, BH), −20.8 (s, 1B, BH). ^11^B‐NMR (128 MHz, CDCl_3_): *δ* [ppm] = 1.4 (s, 1B, BN), −6.8 (d, 2B, BH), −10.0 to −16.8 (m, 5B, BH), −19.5 (m, 2B, BH). HR‐MS (ESI(+), acetonitrile): *m/z* calc. [C_18_H_23_B_10_NO_2_] ([M + H]^+^): 394.2810, found: 394.2821. IR (KBr): $\tilde{v}$ [cm^−1^] = 3055 (m), 3035 (m), 2932 (w), 2827 (w), 2605 (s), 2556 (m), 2360 (w), 1647 (s), 1617 (s), 1599 (s), 1580 (s), 1507 (s), 1449 (s), 1328 (s), 1269 (s),1238 (s), 1178 (s), 1121 (s), 1034 (s), 1006 (s), 980 (s), 834 (s), 770 (s),753 (s), 699 (s), 545 (s). Elemental analysis (C_18_H_23_B_10_NO_2_) calc.(%): C 54.94, H 5.98, N 3.56, found(%): C 54.99, H 6.07, N 3.50.

*N*‐(*closo*‐1,7‐dicarbadodecaboran(12)‐9‐yl)‐4‐(3,4‐dimethoxyphenyl)isoquinolin‐1(2*H*)‐one (**IC‐5**) was obtained from 4‐(3,4‐dimethoxyphenyl)isoquinolin‐1(2*H*)‐one as a colorless solid in 67% yield according to the general procedure described above. *R*_f_ = 0.27 (*n*‐hexane/EtOAc, 7:3, *v/v*). Mp. = 208–210°C (*n*‐pentane). ^1^H NMR (400 MHz, CDCl_3_): *δ* [ppm] = 8.51 (dd, ^3^ *J*_HH_ = 8.0 Hz, ^4^ *J*_HH_ = 1.4 Hz, 1H, H5), 7.57 (ddd, ^3^ *J*_HH_ = 8.2, 6.8 Hz, ^4^ *J*_HH_ = 1.5 Hz, 1H, H7), 7.53–7.45 (m, 2H, H6, H8), 7.34 (d, ^3^ *J*_HH_ = 4.5 Hz, 1H, H3), 6.96 (d, ^3^ *J*_HH_ = 10.0 Hz, 3H, H2′, H3′, H6′), 3.95 (s, 3H, OCH_3_), 3.91 (s, 3H, OCH_3_), 3.60–1.70 (br, 9H, cluster‐BH), 2.98 (s, 2H, cluster‐CH). ^13^C{^1^H} NMR (101 MHz, CDCl_3_): *δ* [ppm] = 165.3 (1C, C1), 149.0 (1C, C5′), 148.7 (1C, C4′), 137.1 (1C, C8a), 133.1 (1C, C3), 132.3 (1C, C7), 129.9 (1C, C5a), 128.7 (1C, C5), 126.9 (1C, C6), 126.5 (1C, C1′), 124.5 (1C, C8), 122.4 (1C, C2′), 119.6 (1C, C4), 113.5 (2C, C3′), 111.5 (1C, C6′), 56.2 (1C, OCH_3_), 56.1 (1C, OCH_3_), 52.5 (2C, cluster‐C). ^11^B{^1^H} NMR (128 MHz, CDCl_3_): *δ* [ppm] = 1.4 (s, 1B, BN), −6.7 (s, 2B, BH), −11.2 (s, 1B, BH), −13.5 (s, 2B, BH), −15.2 (s, 2B, BH), −18.4 (s, 1B, BH), −20.8 (s, 1B, BH). ^11^B‐NMR (128 MHz, CDCl_3_): *δ* [ppm] = 1.4 (s, 1B, BN), −6.7 (d, 2B, BH), −10.1 to −16.9 (m, 5B, BH), −17.2 to −22.1 (m, 2B, BH). HR‐MS (ESI(+), acetonitrile): *m/z* calc. [C_19_H_25_B_10_NO_3_] ([M + H]^+^): 424.2916, found: 424.2930. IR (KBr): $\tilde{v}$ [cm^−1^] = 3040 (m), 2611 (s), 2359 (w), 1646 (s), 1617 (s), 1594 (s), 1492 (s), 1480 (s), 1466 (s), 1448 (s), 1425 (s), 1282 (s), 1270 (s), 1220 (s), 1180 (s), 1152 (s), 1115 (s), 1064 (s), 1027 (s), 1007 (s), 971 (s), 909 (s), 889 (s), 784 (s), 738 (s), 768 (s), 702 (s), 690 (s), 605 (s), 547 (s), 532 (s), 502 (s), 493 (s), 465 (s), 436 (s), 428 (s). Elemental analysis (C_19_H_25_B_10_NO_3_) calc.(%): C 53.88, H 5.95, N 3.31, found(%): C 53.96, H 6.08, N 3.30.

*N*‐(*closo*‐1,7‐dicarbadodecaboran(12)‐9‐yl)‐4‐(3,4,5‐trimethoxyphenyl)isoquinolin‐1(2*H*)‐one (**IC‐6**) was obtained from 4‐(3,4,5‐trimethoxyphenyl)isoquinolin‐1(2*H*)‐one as a colorless solid in 61% yield according to the general procedure described above. *R*_f_ = 0.31 (*n*‐hexane/EtOAc, 3:2, *v/v*). Mp. = 208–210°C (*n*‐pentane). ^1^H NMR (400 MHz, CDCl_3_): *δ* [ppm] = 8.51 (dd, ^3^ *J*_HH_ = 8.0 Hz, ^4^ *J*_HH_ = 1.4 Hz, 1H, H5), 7.59 (ddd, ^3^ *J*_HH_ = 8.2, 6.7 Hz, ^4^ *J*_HH_ = 1.4 Hz, 1H, H7), 7.53 (d, ^3^ *J*_HH_ = 7.9 Hz, 1H, H8), 7.50–7.46 (m, 1H, H6), 7.36 (d, ^3^ *J*_HH_ = 4.3 Hz, 1H, H3), 6.63 (s, 2H, H2′), 3.93 (s, 3H, OCH_3_), 3.89 (s, 6H, OCH_3_), 3.60–1.50 (br, 9H, cluster‐BH), 2.99 (s, 2H, cluster‐CH). ^13^C{^1^H} NMR (101 MHz, CDCl_3_): *δ* [ppm] = 165.6 (1C, C1), 153.4 (2C, C3′), 137.6 (1C, C8a), 136.9 (1C, C4′), 133.1 (1C, C3), 132.8 (1C, C1′), 132.4 (1C, C7), 128.8 (1C, C5), 126.9 (1C, C6), 126.5 (1C, C5a), 124.4 (1C, C8), 119.9 (1C, C4), 107.4 (2C, C2′), 61.1 (1C, OCH_3_), 56.4 (2C, OCH_3_), 52.6 (2C, cluster‐C). ^11^B{^1^H} NMR (128 MHz, CDCl_3_): *δ* [ppm] = 1.3 (s, 1B, BN), −6.7 (s, 2B, BH), −11.2 (s, 1B, BH), −13.5 (s, 2B, BH), −15.2 (s, 2B, BH), −18.3 (s, 1B, BH), −20.8 (s, 1B, BH). ^11^B‐NMR (128 MHz, CDCl_3_): *δ* [ppm] = 1.3 (s, 1B, BN), −6.7 (d, 2B, BH), −9.7 to −16.8 (m, 5B, BH), −19.5 (m, 2B, BH). HR‐MS (ESI(+), acetonitrile): *m/z* calc. [C_20_H_29_B_10_NO_4_] ([M + H]^+^): 454.3022, found: 454.3043. IR (KBr): $\tilde{v}$ [cm^−1^] = 3054 (m), 2933 (w), 2608 (m), 2588 (m), 2555 (m), 1649 (s), 1616 (s), 1579 (s), 1506 (m), 1484 (m), 1448 (m), 1414 (m), 1380 (m), 1327 (s), 1306 (s), 1269 (s), 1238 (s), 1177 (s), 1123 (s), 1067 (m), 1033 (m), 1008 (s), 979 (m), 869 (m), 834 (m), 773 (s), 752 (s), 724 (s), 700 (s), 666 (s), 645 (s), 633 (s), 600 (m), 545 (m), 528 (m). Elemental analysis (C_20_H_27_B_10_NO_4_) calc.(%): C 52.96, H 6.00, N 3.09, found(%): C 52.85, H 6.07, N 3.03.

*N*‐(*closo*‐1,7‐dicarbadodecaboran(12)‐9‐yl)‐6,7‐dimethoxyisoquinolin‐1(2*H*)‐one (**IC‐7**) was obtained from 6,7‐dimethoxyisoquinolin‐1(2*H*)‐one as a beige solid in 22% yield according to the general procedure described above. *R*_f_ = 0.15 (*n*‐hexane/EtOAc, 2:1, *v/v*). Mp. = 217–219°C (*n*‐pentane). ^1^H NMR (400 MHz, CDCl_3_): *δ* [ppm] = 7.83 (s, 1H, H5), 7.32 (t, ^3^ *J*_HH_ = 6.2 Hz, 1H, H3), 6.82 (s, 1H, H8), 6.36 (d, ^3^ *J*_HH_ = 7.4 Hz, 1H, H4), 3.97 (s, 6H, OCH_3_), 3.50–1.60 (br, 9H, cluster‐BH), 2.96 (s, 2H, cluster‐CH). ^13^C{^1^H} NMR (101 MHz, CDCl_3_): *δ* [ppm] = 165.1 (1C, C1), 153.5 (1C, C6), 149.2 (1C, C7), 132.9 (1C, C3), 120.6, 108.4 (1C, C5), 106.1 (1C, C4), 105.8 (1C, C8), 56.3 (2C, OCH_3_), 52.5 (2C, cluster‐C). ^11^B{^1^H} NMR (128 MHz, CDCl_3_): *δ* [ppm] = 1.5 (s, 1B, BN), −6.6 (s, 2B, BH), −11.3 (s, 1B, BH), −13.4 (s, 2B, BH), −15.2 (s, 2B, BH), −18.3 (s, 1B, BH), −20.8 (s, 1B, BH). ^11^B‐NMR (128 MHz, CDCl_3_): *δ* [ppm] = 1.5 (s, 1B, BN), −6.7 (d, 2B, BH), −10.2 to −16.7 (m, 5B, BH), −19.5 (m, 2B, BH). HR‐MS (ESI(+), acetonitrile): *m/z* calc. [C_13_H_21_B_10_NO_3_] ([M + H]^+^): 348.2603, found: 348.2620. IR (KBr): $\tilde{v}$ [cm^−1^] = 3040 (m), 2611 (s), 2359 (w), 1740 (w), 1643 (s), 1617 (s), 1594 (s), 1497 (s), 1469 (s), 1427 (s), 1269 (s), 1217 (s), 1180 (s), 1105 (s), 1064 (s), 1027 (s), 1009 (s), 973 (s), 861 (s), 784 (s), 768 (s), 736 (s), 702 (s), 690 (s), 657 (s), 534 (s), 464 (s). Elemental analysis (C_13_H_21_B_10_NO_3_) calc.(%): C 44.94, H 6.09, N 4.03, found(%): C 45.04, H 6.16, N 4.00.

*N*‐(*closo*‐1,7‐dicarbadodecaboran(12)‐9‐yl)‐6,7‐dimethoxy‐4‐phenylisoquinolin‐1(2*H*)‐one (**IC‐8**) was obtained from 6,7‐dimethoxy‐4‐phenylisoquinolin‐1(2*H*)‐one as a beige solid in 74% yield according to the general procedure described above. Single crystals were obtained by slow evaporation of a saturated acetone solution of **IC‐8**. *R*_f_ = 0.35 (*n*‐hexane/EtOAc, 2:1, *v/v*). Mp. = 231–233°C (*n*‐pentane). ^1^H NMR (400 MHz, CDCl_3_): *δ* [ppm] = 7.87 (s, 1H, H5), 7.41–7.37 (m, 5H, H2′, H3′, H4′), 7.21 (d, ^3^ *J*_HH_ = 4.5 Hz, 1H, H3), 6.83 (s, 1H, H8), 3.93 (s, 3H, OCH_3_), 3.74 (s, 3H, OCH_3_), 3.40–1.50 (br, 9H, cluster‐BH), 2.91 (s, 2H, cluster‐CH). ^13^C{^1^H} NMR (101 MHz, CDCl_3_): *δ* [ppm] = 164.7 (1C, C1), 153.3 (1C, C6), 149.1 (1C, C7), 137.5 (1C, C1′), 132.2 (1C, C8a), 132.1 (1C, C3), 129.9 (2C, C2′ or C3′), 128.8 (2C, C2′ or C3′), 127.6 (1C, C4′), 120.4 (1C, C4), 119.6 (1C, C5a), 108.8 (1C, C5), 105.0 (1C, C8), 56.2 (1C, OCH_3_), 56.1 (1C, OCH_3_), 52.5 (2C, cluster‐C). ^11^B{^1^H} NMR (128 MHz, CDCl_3_): *δ* [ppm] = 1.5 (s, 1B, BN), −6.6 (s, 2B, BH), −11.1 (s, 1B, BH), −13.3 (s, 2B, BH), −15.1 (s, 2B, BH), −18.3 (s, 1B, BH), −20.6 (s, 1B, BH). ^11^B‐NMR (128 MHz, CDCl_3_): *δ* [ppm] = 1.5 (s, 1B, BN), −6.6 (d, 2B, BH), −10.0 to −16.4 (m, 5B, BH), −17.0 to −21.9 (m, 2B, BH). HR‐MS (ESI(+), acetonitrile): *m/z* calc. [C_19_H_25_B_10_NO_3_] ([M + H]^+^): 424.2916, found: 424.2936. IR (KBr): $\tilde{v}$ [cm^−1^] = 3036 (m), 2923 (m), 2852 (m), 2602 (m), 2359 (w), 1734 (w), 1643 (s), 1593 (s), 1504 (s), 1464 (s), 1439 (s), 1422 (s), 1394 (s), 1283 (s), 1258 (s), 1211 (s), 1190 (s), 1146 (s), 1114 (s), 1060 (s), 1033 (s), 1015 (s), 891 (s), 875 (s), 853 (s), 817 (s), 788 (s), 765 (s), 738 (s), 705 (s), 691 (s), 603 (s), 538 (s), 466 (m). Elemental analysis (C_19_H_25_B_10_NO_3_) calc.(%): C 53.88, H 5.95, N 3.31, found(%): C 53.80, H 5.99, N 3.28.

*N*‐(*closo*‐1,7‐dicarbadodecaboran(12)‐9‐yl)‐4‐(3‐methoxyphenyl)isoquinolin‐1(2*H*)‐one (**IC‐9**) was obtained from 6,7‐dimethoxy‐4‐(3‐methoxyphenyl)isoquinolin‐1(2*H*)‐one as a beige solid in 79% yield according to the general procedure described above. *R*_f_ = 0.21 (*n*‐hexane/EtOAc, 2:1, *v/v*). Mp. = 262–264°C (*n*‐pentane). ^1^H NMR (400 MHz, CDCl_3_): *δ* [ppm] = 7.94 (s, 1H, H5), 7.39 (t, ^3^ *J*_HH_ = 7.8 Hz, 1H, H3′), 7.29 (d, ^3^ *J*_HH_ = 4.4 Hz, 1H, H3), 7.04 (d, ^3^ *J*_HH_ = 7.6 Hz, 1H, H4′), 6.99 (t, ^4^ *J*_HH_ = 2.1 Hz, 1H, H6′), 6.98–6.93 (m, 2H, H8, H2′), 4.00 (s, 3H, OCH_3_), 3.86 (s, 3H, OCH_3_), 3.83 (s, 3H, OCH_3_), 3.50–1.70 (br, 9H, cluster‐BH), 2.98 (s, 2H, cluster‐CH). ^13^C{^1^H} NMR (101 MHz, CDCl_3_): *δ* [ppm] = 164.6 (1C, C1), 160.0 (1C, C5′), 153.3 (1C, C6), 149.1 (1C, C7), 139.0 (1C, C1′), 132.1 (1C, C8a), 132.0 (1C, C3), 129.8 (1C, C3′), 122.4 (1C, C4′), 120.4 (1C, C4), 119.4 (1C, C5a), 115.6 (1C, C6′), 113.0 (1C, C2′), 108.8 (1C, C5), 105.0 (1C, C8), 56.2 (1C, OCH_3_), 56.1 (1C, OCH_3_), 55.5 (1C, OCH_3_), 52.5 (2C, cluster‐C). ^11^B{^1^H} NMR (128 MHz, CDCl_3_): *δ* [ppm] = 1.4 (s, 1B, BN), −6.7 (s, 2B, BH), −11.3 (s, 1B, BH), −13.5 (s, 2B, BH), −15.2 (s, 2B, BH), −18.4 (s, 1B, BH), −20.8 (s, 1B, BH). ^11^B‐NMR (128 MHz, CDCl_3_): *δ* [ppm] = 1.4 (s, 1B, BN), −6.7 (d, 2B, BH), −9.9 to −16.7 (m, 5B, BH), −17.2 to −22.0 (m, 2B, BH). HR‐MS (ESI(+), acetonitrile): *m/z* calc. [C_20_H_29_B_10_NO_4_] ([M + H]^+^): 454.3022, found: 454.3037. IR (KBr): $\tilde{v}$ [cm^−1^] = 3040 (m), 2929 (w), 2828 (w), 2600 (m), 2361 (w), 1734 (w), 1643 (s), 1591 (s), 1503 (s), 1464 (s), 1392 (s), 1207 (s), 1259 (s), 1208 (s), 1190 (s), 1114 (s), 1034 (s), 1009 (s), 849 (s), 786 (s), 745 (s), 713 (s), 691 (s). Elemental analysis (C_20_H_27_B_10_NO_4_) calc.(%): C 52.96, H 6.00, N 3.09, found(%): C 53.03, H 6.05, N 3.08.

*N*‐(*closo*‐1,7‐dicarbadodecaboran(12)‐9‐yl)‐6,7‐dimethoxy‐4‐(4‐methoxyphenyl)isoquinolin‐1(2*H*)‐one (**IC‐10**) was obtained from 6,7‐dimethoxy‐4‐(4‐methoxyphenyl)isoquinolin‐1(2*H*)‐one as an off‐white solid in 40% yield according to the general procedure described above. Single crystals were obtained by slow evaporation of a saturated acetone solution of **IC‐10**. *R*_f_ = 0.23 (*n*‐hexane/EtOAc, 2:1, *v/v*). M.p. = 211–213°C (*n*‐pentane). ^1^H NMR (400 MHz, CDCl_3_): *δ* [ppm] = 7.94 (s, 1H, H5), 7.38–7.34 (m, 2H, H2′), 7.24 (d, ^3^ *J*_HH_ = 4.6 Hz, 1H, H3), 7.03–6.99 (m, 2H, H3′), 6.87 (s, 1H, H8), 3.99 (s, 3H, OCH_3_), 3.88 (s, 3H, OCH_3_), 3.82 (s, 3H, OCH_3_), 3.50–1.70 (br, 9H, cluster‐BH), 2.97 (s, 2H, cluster‐CH). ^13^C{^1^H} NMR (101 MHz, CDCl_3_): *δ* [ppm] = 164.7 (1C, C1), 159.2 (1C, C4′), 153.3 (1C, C6), 149.1 (1C, C7), 132.5 (1C, C1′), 131.9 (1C, C3), 131.0 (2C, C2′), 129.8 (1C, C8a), 120.4 (1C, C4), 119.2 (1C, C5a), 114.2 (2C, C3′), 108.8 (1C, C5), 105.0 (1C, C8), 56.2 (1C, OCH_3_), 56.1 (1C, OCH_3_), 55.5 (1C, OCH_3_), 52.5 (2C, cluster‐C). ^11^B{^1^H} NMR (128 MHz, CDCl_3_): *δ* [ppm] = 1.4 (s, 1B, BN), −6.7 (s, 2B, BH), −11.3 (s, 1B, BH), −13.5 (s, 2B, BH), −15.2 (s, 2B, BH), −18.4 (s, 1B, BH), −20.8 (s, 1B, BH). ^11^B‐NMR (128 MHz, CDCl_3_): *δ* [ppm] = 1.4 (s, 1B, BN), −6.7 (d, 2B, BH), −9.9 to −16.8 (m, 5B, BH), −17.2 to −22.0 (m, 2B, BH). HR‐MS (ESI(+), acetonitrile): *m/z* calc. [C_20_H_29_B_10_NO_4_] ([M + H]^+^): 454.3022, found: 454.3032. IR (KBr): $\tilde{v}$ [cm^−1^] = 3054 (m), 2933 (w), 2826 (w), 2605 (m), 2555 (m), 2539 (w), 1647 (s), 1616 (s), 1598 (s), 1580 (s), 1502 (s), 1484 (s), 1449 (s), 1415 (s), 1275 (s), 1230 (s), 1213 (s), 1177 (s), 1120 (s), 1033 (s), 1009 (s), 980 (s), 833 (s), 736 (s), 723 (s), 700 (s), 645 (s), 633 (s), 545 (s), 528 (s). Elemental analysis (C_20_H_27_B_10_NO_4_) calc.(%): C 52.96, H 6.00, N 3.09, found(%): C 52.87, H 6.10, N 3.07.

*N* *‐*(*closo*‐1,7‐dicarbadodecaboran(12)‐9‐yl)‐4‐(3,4‐dimethoxyphenyl)‐6,7‐dimethoxyisoquinolin‐1(2*H*)‐one (**IC‐11**) was obtained from 4‐(3,4‐dimethoxyphenyl)‐6,7‐dimethoxyisoquinolin‐1(2*H*)‐one as a colorless solid in 29% yield according to the general procedure described above. *R*_f_ = 0.24 (*n*‐hexane/EtOAc, 3:2, *v/v*). Mp. = 206–208°C (*n*‐pentane). ^1^H NMR (400 MHz, CDCl_3_): *δ* [ppm] = 7.94 (s, 1H, H5), 7.27 (s, 1H, H3), 6.97 (d, 3H, H2′, H3′, H6′), 6.90 (s, 1H, H8), 3.99 (s, 3H, OCH_3_), 3.95 (s, 3H, OCH_3_), 3.90 (s, 3H, OCH_3_), 3.81 (s, 3H, OCH_3_), 3.60–1.70 (br, 9H, cluster‐BH), 2.98 (s, 2H, cluster‐CH). ^13^C{^1^H} NMR (101 MHz, CDCl_3_): *δ* [ppm] = 164.6 (1C, C1), 153.3 (1C, C5′), 149.1 (1C, C6), 149.0 (1C, C4′), 148.6 (1C, C7), 132.4 (1C, C1′), 131.9 (1C, C3), 130.1 (1C, C8a), 122.2 (1C, C2′), 120.4 (1C, C4), 119.3 (1C, C5a), 113.2 (1C, C6′), 111.5 (1C, C3′), 108.8 (1C, C5), 105.0 (1C, C8), 56.2 (1C, OCH_3_), 56.2 (1C, OCH_3_), 56.1 (2C, OCH_3_), 52.5 (2C, cluster‐C). ^11^B{^1^H} NMR (128 MHz, CDCl_3_): *δ* [ppm] = 1.5 (s, 1B, BN), −6.6 (s, 2B, BH), −11.2 (s, 1B, BH), −13.4 (s, 2B, BH), −15.1 (s, 2B, BH), −18.3 (s, 1B, BH), −20.7 (s, 1B, BH). ^11^B‐NMR (128 MHz, CDCl_3_): *δ* [ppm] = 1.5 (s, 1B, BN), −6.6 (d, 2B, BH), −9.8 to −16.7 (m, 5B, BH), −17.2 to −21.6 (m, 2B, BH). HR‐MS (ESI(+), acetonitrile): *m/z* calc. [C_21_H_29_B_10_NO_5_] ([M + H]^+^): 484.3127, found: 484.3144. IR (KBr): $\tilde{v}$ [cm^−1^] = 3053 (w), 3022 (m), 2923 (w), 2631 (m), 2588 (m), 1641 (s), 1595 (s), 1504 (s), 1467 (s), 1443 (s), 1392 (s), 1285 (s), 1271 (s), 1252 (s), 1206 (s), 1191 (s), 1171 (s), 1137 (s), 1114 (s), 1026 (s), 1014 (s), 1002 (s), 848 (s), 816 (s), 786 (s), 764 (s), 747 (s), 689 (s), 589 (s), 502 (s). Elemental analysis (C_21_H_29_B_10_NO_5_) calc.(%): C 52.16, H 6.04, N 2.90, found(%): C 52.22, H 6.11, N 2.89.

### 4.2 Biological Studies

#### 4.2.1 Cultivation of Cells

Madin–Darby canine kidney cells (MDCKII) WT and their related cells stably transfected with hABCG2 (MDCKII‐hABCG2) were purchased from Alfred Schinkel (Het Nederlands Kanker Instituut, Amsterdam, Netherlands). Minimum essential medium (MEM) with Earle's Salts (2.2 g/L NaHCO_3_, stable glutamine; Biowest, Nuaillé, France) supplemented with 10% (*v/v*) fetal calf serum (Life Technology, Karlsruhe, Germany), 1% (*v/v*) nonessential amino acids (Biowest, Nuaillé, France), 100 U/mL penicillin, and 100 µg/mL streptomycin (Biowest, Nuaillé, France) was used for cultivation. Cells were grown in a humidified atmosphere (37°C, 5% CO_2_) and were subcultured every 3–4 days using 0.05% trypsin/0.02% EDTA (Biowest, Nuaillé, France), up to a total of 14 passages.

#### 4.2.2 Detection of Cell Viability by WST‐1 Proliferation Assay

MDCKII‐hABCG2 cells and their parental MDCKII‐WT cells were seeded in 96‐well plates (TPP, Trasadingen, Switzerland) in a density of 2 × 10^4^ and 3 × 10^4^ cells/mL, respectively. The cells were treated with increasing concentrations up to 50 µM of compounds **IC‐1** to **IC‐11** (Table 1), with 0.1% Triton X‐100 as a positive control and solvent (0.1% DMSO) as a negative control for 48 h. The substance‐specific cytotoxicity was subsequently determined by WST‐1 (4‐[3‐(4‐iodophenyl)‐2‐(4‐nitro‐phenyl)‐2*H*‐5‐tetrazolio]‐1,3‐benzene sulfonate; WST‐1) assay. Afterward, the cells were washed twice with 200 µL/well of prewarmed PBS, and then 100 µL/well of WST‐1 reagent (5% *v/v*) was added. The cell viability is reflected by an increase of formazan formation and was determined by a microplate reader at 450 nm (Tecan Sunrise, Crailsheim, Germany) as described previously [25, 35, 36].

#### 4.2.3 Determination of ABCG2 Inhibition by Hoechst 33342 Accumulation Assay

An interaction of an investigated compound with the ABCG2 transporter causes an increase of the intracellular Hoechst 33342 fluorescent dye. Hence, MDCKII‐WT (3.5 × 10^4^ cells/mL) and MDCKII‐hABCG2 cells (2.5 × 10^4^ cells/mL) were seeded in 96‐well plates and cultured for 72 h. Afterward, the cells were treated with 0.5 and 1.0 µM of the examined compounds, its respective solvent control (0.1% DMSO) or with the positive control Ko143 for 4 h. Then, the Hoechst 33342 accumulation assay was performed as described previously [25, 35, 36]. The intracellular Hoechst 33342 amount was detected by spectrofluorometry (360 nm excitation/465 nm emission wavelengths, Tecan Infinite F200 Pro, Crailsheim, Germany) and was correlated to the protein amount quantified by bicinchoninic acid assay (BCA) (Thermo Scientific, Rockford, USA) following the manufacturer's instructions.

#### 4.2.4 Evaluation of Autofluorescence

MDCKII and MDCKII‐hABCG2 cells were seeded and treated as described for Hoechst 33342 accumulation assay. Subsequently, intracellular autofluorescence was detected by spectrofluorometer (360 nm excitation/465 nm emission wavelengths, Tecan Infinite F200 Pro, Crailsheim, Germany). The total intracellular fluorescence was correlated to the protein amount quantified by BCA (Thermo Scientific, Rockford, USA) following the manufacturer's instructions. Autofluorescence was defined as a significant increase of total intracellular fluorescence unit (RFU) in comparison to solvent‐treated control. A more detailed description was published previously [25, 35, 36].

#### 4.2.5 Determination of the Reversal of Mitoxantrone Resistance

The MDCKII cells were seeded as described in the WST‐1 assay section and treated with increasing up to 50 µM of MXN alone or in combination with selected compounds **IC‐1** to **IC‐11** (1.0 µM) for 48 h. An incubation with 0.1% Triton X‐100 served as a positive control, and untreated MDCKII cells were used as a negative control. The treatment was renewed once a day. Afterward, the WST‐1 assay was performed as described previously [25, 35, 36]. The left‐shift factor was calculated from IC_50_ minus standard error of mean (SEM) from the MXN‐treated cells by IC_50_ plus SEM obtained from combined treatment of MXN and investigated compounds.

#### 4.2.6 Statistical Analysis

Data from WST‐1 cell viability assays (*N* = 3), Hoechst 33342 accumulation assay (*N* = 5), autofluorescence assay (*N* = 3), and MXN reversal assay (*N* = 3) were tested for normality with Shapiro–Wilk test. All data were analyzed by one‐way ANOVA with Holm–Šidák post hoc test using SigmaPlot 14.5 (Systec Software, San Jose, CA, USA). IC_50_ values were defined as 50% reduced cell viability and calculated with SigmaPlot 14.5 by nonlinear regression. In order to detect significant differences between groups treated with MXN, two‐way ANOVA with Holm–Šidák post hoc test was additionally performed by using SigmaPlot 14.5. With the exception of the autofluorescence data, all obtained values were normalized against vehicle control (0.1% DMSO), which was set as 1, and were expressed as mean ± SEM, calculated from at least three independent experiments.

## Supporting Information

Additional supporting information can be found online in the Supporting Information section. **Supporting Fig. S1**: ^1^H NMR spectrum of **2** in DMSO‐*d*_6_. **Supporting Fig. S2**: ^1^H NMR spectrum of **4** in DMSO‐*d*_6_. **Supporting Fig. S3**: ^1^H NMR spectrum of **5** in DMSO‐*d*_6_. **Supporting Fig. S4**: ^1^H NMR spectrum of **6a** in DMSO‐*d*_6_. **Supporting Fig. S5**: ^13^C{^1^H} NMR spectrum of **6a** in DMSO‐*d*_6_. **Supporting Fig. S6**: ^1^H‐COSY NMR spectrum of **6a** in DMSO‐*d*_6_. **Supporting Fig. S7**: HSQC NMR spectrum of **6a** in DMSO *d*_6_. **Supporting Fig. S8**: ^1^H NMR spectrum of **6b** in DMSO‐*d*_6_. **Supporting Fig. S9**: ^13^C{^1^H} NMR spectrum of **6b** in DMSO‐*d*_6_. **Supporting Fig. S10**: ^1^H‐COSY NMR spectrum of **6b** in DMSO‐*d*_6_. **Supporting Fig. S11**: HSQC NMR spectrum of **6b** in DMSO‐*d*_6_. **Supporting Fig. S12**: ^1^H NMR spectrum of **6c** in DMSO‐*d*_6_. **Supporting Fig. S13**: ^13^C{^1^H} NMR spectrum of **6c** in DMSO‐*d*_6_. **Supporting Fig. S14**: ^1^H‐COSY NMR spectrum of **6c** in DMSO‐*d*_6_. **Supporting Fig. S15**: HSQC NMR spectrum of **6c** in DMSO‐*d*_6_. **Supporting Fig. S16**: ^1^H NMR spectrum of **6d** in DMSO‐*d*_6_. **Supporting Fig. S17**: ^13^C{^1^H} NMR spectrum of **6d** in DMSO‐*d*_6_. **Supporting Fig. S18**: ^1^H‐COSY NMR spectrum of **6d** in DMSO‐*d*_6_. **Supporting Fig. S19**: HSQC NMR spectrum of **6d** in DMSO‐*d*_6_. **Supporting Fig. S20**: ^1^H NMR spectrum of **6e** in DMSO‐*d*_6_. **Supporting Fig. S21**: ^13^C{^1^H} NMR spectrum of **6e** in DMSO‐*d*_6_. **Supporting Fig. S22**: ^1^H‐COSY NMR spectrum of **6e** in DMSO‐*d*_6_. **Supporting Fig. S23**: HSQC NMR spectrum of **6e** in DMSO‐*d*_6_. **Supporting Fig. S24**: ^1^H NMR spectrum of **7a** in DMSO‐*d*6. **Supporting Fig. S25**: ^13^C{^1^H} NMR spectrum of **7a** in DMSO‐*d*_6_. **Supporting Fig. S26**: ^1^H‐COSY NMR spectrum of **7a** in DMSO‐*d*_6_. **Supporting Fig. S27**: HSQC NMR spectrum of **7a** in DMSO‐*d*6. **Supporting Fig. S28**: ^1^H NMR spectrum of **7b** in DMSO‐*d*_6_. **Supporting Fig. S29**: ^13^C{^1^H} NMR spectrum of **7b** in DMSO‐*d*_6_. **Supporting Fig. S30**: ^1^H‐COSY NMR spectrum of **7b** in DMSO‐*d*_6_. **Supporting Fig. S31**: HSQC NMR spectrum of **7b** in DMSO‐*d*_6_. **Supporting Fig. S32**: ^1^H NMR spectrum of **7c** in DMSO‐*d*_6_. **Supporting Fig. S33**: ^13^C{^1^H} NMR spectrum of **7c** in DMSO‐*d*_6_. **Supporting Fig. S34**: ^1^H‐COSY NMR spectrum of **7c** in DMSO‐*d*_6_. **Supporting Fig. S35**: HSQC NMR spectrum of **7c** in DMSO‐*d*_6_. **Supporting Fig. S36**: ^1^H NMR spectrum of **7d** in DMSO‐*d*_6_. **Supporting Fig. S37**: ^13^C{^1^H} NMR spectrum of **7d** in DMSO‐*d*_6_. **Supporting Fig. S38**: ^1^H‐COSY NMR spectrum of **7d** in DMSO‐*d*_6_. **Supporting Fig. S39**: HSQC NMR spectrum of **7d** in DMSO‐*d*_6_. **Supporting Fig. S40**: ^1^H NMR spectrum of **IC‐1** in CDCl_3_. **Supporting Fig. S41**: ^13^C{^1^H} NMR spectrum of **IC‐1** in CDCl_3_. **Supporting Fig. S42**: ^1^H‐COSY NMR spectrum of **IC‐1** in CDCl_3_. **Supporting Fig. S43**: HSQC NMR spectrum of **IC‐1** in CDCl_3_. **Supporting Fig. S44**: ^11^B{^1^H} NMR spectrum of **IC‐1** in CDCl_3_. **Supporting Fig. S45**: ^11^B NMR spectrum of **IC‐1** in CDCl_3_. **Supporting Fig. S46**: ^1^H NMR spectrum of **IC‐2** in CDCl_3_. **Supporting Fig. S47**: ^13^C{^1^H} NMR spectrum of **IC‐2** in CDCl_3_. **Supporting Fig. S48**: ^1^H‐COSY NMR spectrum of **IC‐2** in CDCl_3_. **Supporting Fig. S49**: HSQC NMR spectrum of **IC‐2** in CDCl_3_. **Supporting Fig. S50**: ^11^B{^1^H} NMR spectrum of **IC‐2** in CDCl_3_. **Supporting Fig. S51**: ^11^B NMR spectrum of **IC‐2** in CDCl_3_. **Supporting Fig. S52**: ^1^H NMR spectrum of **IC‐3** in CDCl_3_. **Supporting Fig. S53**: ^13^C{^1^H} NMR spectrum of **IC‐3** in CDCl_3_. **Supporting Fig. S54**: ^1^H‐COSY NMR spectrum of **IC‐3** in CDCl_3_. **Supporting Fig. S55**: HSQC NMR spectrum of **IC‐3** in CDCl_3_. **Supporting Fig. S56**: ^11^B{^1^H} NMR spectrum of **IC‐3** in CDCl_3_. **Supporting Fig. S57**: ^11^B NMR spectrum of **IC‐3** in CDCl_3_. **Supporting Fig. S58**: ^1^H NMR spectrum of **IC‐4** in CDCl_3_. **Supporting Fig. S59**: ^13^C{^1^H} NMR spectrum of **IC‐4** in CDCl_3_. **Supporting Fig. S60**: ^1^H‐COSY NMR spectrum of **IC‐4** in CDCl_3_. **Supporting Fig. S61**: HSQC NMR spectrum of **IC‐4** in CDCl_3_. **Supporting Fig. S62**: ^11^B{^1^H} NMR spectrum of **IC‐4** in CDCl_3_. **Supporting Fig. S63**: ^11^B NMR spectrum of **IC‐4** in CDCl_3_. **Supporting Fig. S64**: ^1^H NMR spectrum of **IC‐5** in CDCl_3_. **Supporting Fig. S65**: ^13^C{^1^H} NMR spectrum of **IC‐5** in CDCl_3_. **Supporting Fig. S66**: ^1^H‐COSY NMR spectrum of **IC‐5** in CDCl_3_. **Supporting Fig. S67**: HSQC NMR spectrum of **IC‐5** in CDCl_3_. **Supporting Fig. S68**: ^11^B{^1^H} NMR spectrum of **IC‐5** in CDCl_3_. **Supporting Fig. S69**: ^11^B NMR spectrum of **IC‐5** in CDCl_3_. **Supporting Fig. S70**: ^1^H NMR spectrum of **IC‐6** in CDCl_3_. **Supporting Fig. S71**: ^13^C{^1^H} NMR spectrum of **IC‐6** in CDCl_3_. **Supporting Fig. S72**: ^1^H‐COSY NMR spectrum of **IC‐6** in CDCl_3_. **Supporting Fig. S73**: HSQC NMR spectrum of **IC‐6** in CDCl_3_. **Supporting Fig. S74**: ^11^B{^1^H} NMR spectrum of **IC‐6** in CDCl_3_. **Supporting Fig. S75**: ^11^B NMR spectrum of **IC‐6** in CDCl_3_. **Supporting Fig. S76**: ^1^H NMR spectrum of **IC‐7** in CDCl_3_. **Supporting Fig. S77**: ^13^C{^1^H} NMR spectrum of **IC‐7** in CDCl_3_. **Supporting Fig. S78**: ^1^H‐COSY NMR spectrum of **IC‐7** in CDCl_3_. **Supporting Fig. S79**: HSQC NMR spectrum of **IC‐7** in CDCl_3_. **Supporting Fig. S80**: ^11^B{^1^H} NMR spectrum of **IC‐7** in CDCl_3_. **Supporting Fig. S81**: ^11^B NMR spectrum of **IC‐7** in CDCl_3_. **Supporting Fig. S82**: ^1^H NMR spectrum of **IC‐8** in CDCl_3_. **Supporting Fig. S83**: ^13^C{^1^H} NMR spectrum of **IC‐8** in CDCl_3_. **Supporting Fig. S84**: ^1^H‐COSY NMR spectrum of **IC‐8** in CDCl_3_. **Supporting Fig. S85**: HSQC NMR spectrum of **IC‐8** in CDCl_3_. **Supporting Fig. S86**: ^11^B{^1^H} NMR spectrum of **IC‐8** in CDCl_3_. **Supporting Fig. S87**: ^11^B NMR spectrum of **IC‐8** in CDCl_3_. **Supporting Fig. S88**: ^1^H NMR spectrum of **IC‐9** in CDCl_3_. **Supporting Fig. S89**: ^13^C{^1^H} NMR spectrum of **IC‐9** in CDCl_3_. **Supporting Fig. S90**: ^1^H‐COSY NMR spectrum of **IC‐9** in CDCl_3_. **Supporting Fig. S91**: HSQC NMR spectrum of **IC‐9** in CDCl_3_. **Supporting Fig. S92**: ^11^B{^1^H} NMR spectrum of **IC‐9** in CDCl_3_. **Supporting Fig. S93**: ^11^B NMR spectrum of **IC‐9** in CDCl_3_. **Supporting Fig. S94**: ^1^H NMR spectrum of **IC‐10** in CDCl_3_. **Supporting Fig. S95**: ^13^C{^1^H} NMR spectrum of **IC‐10** in CDCl_3_. **Supporting Fig. S96**: ^1^H‐COSY NMR spectrum of **IC‐10** in CDCl_3_. **Supporting Fig. S97**: HSQC NMR spectrum of **IC‐10** in CDCl_3_. **Supporting Fig. S98**: ^11^B{^1^H} NMR spectrum of **IC‐10** in CDCl_3_. **Supporting Fig. S99**: ^11^B NMR spectrum of **IC‐10** in CDCl_3_. **Supporting Fig. S100**: ^1^H NMR spectrum of **IC‐11** in CDCl_3_. **Supporting Fig. S101**: ^13^C{^1^H} NMR spectrum of **IC‐11** in CDCl_3_. **Supporting Fig. S102**: ^1^H‐COSY NMR spectrum of **IC‐11** in CDCl_3_. **Supporting Fig. S103**: HSQC NMR spectrum of **IC‐11** in CDCl_3_. **Supporting Fig. S104**: ^11^B{^1^H} NMR spectrum of **IC‐11** in CDCl_3_. **Supporting Fig. S105**: ^11^B NMR spectrum of **IC‐11** in CDCl_3_. **Supporting Fig. S106**: Mass (HR‐ESI+) spectrum of **4** in CH_3_CN. **Supporting Fig. S107**: Mass (HR‐ESI+) spectrum of **5** in CH_3_CN. **Supporting Fig. S108**: Mass (HR‐ESI+) spectrum of **6a** in CH_3_CN. **Supporting Fig. S109**: Mass (HR‐ESI+) spectrum of **6b** in CH_3_CN. **Supporting Fig. S110**: Mass (HR‐ESI+) spectrum of **6c** in CH_3_CN. **Supporting Fig. S111**: Mass (HR‐ESI+) spectrum of **6d** in CH_3_CN. **Supporting Fig. S112**: Mass (HR‐ESI+) spectrum of **6e** in CH_3_CN. **Supporting Fig. S113**: Mass (HR‐ESI+) spectrum of **7a** in CH_3_CN. **Supporting Fig. S114**: Mass (HR‐ESI+) spectrum of **7b** in CH_3_CN. **Supporting Fig. S115**: Mass (HR‐ESI+) spectrum of **7c** in CH_3_CN. **Supporting Fig. S116**: Mass (HR‐ESI+) spectrum of **7d** in CH_3_CN. **Supporting Fig. S117**: Mass (HR‐ESI+) spectrum of **IC‐1** in CH_3_CN. **Supporting Fig. S118**: Mass (HR‐ESI+) spectrum of **IC‐2** in CH_3_CN. **Supporting Fig. S119**: Mass (HR‐ESI+) spectrum of **IC‐3** in CH_3_CN. **Supporting Fig. S120**: Mass (HR‐ESI+) spectrum of **IC‐4** in CH_3_CN. **Supporting Fig. S121**: Mass (HR‐ESI+) spectrum of **IC‐5** in CH_3_CN. **Supporting Fig. S122**: Mass (HR‐ESI+) spectrum of **IC‐6** in CH_3_CN. **Supporting Fig. S123**: Mass (HR‐ESI+) spectrum of **IC‐7** in CH_3_CN. **Supporting Fig. S124**: Mass (HR‐ESI+) spectrum of **IC‐8** in CH_3_CN. **Supporting Fig. S125**: Mass (HR‐ESI+) spectrum of **IC‐9** in CH_3_CN. **Supporting Fig. S126**: Mass (HR‐ESI+) spectrum of **IC‐10** in CH_3_CN. **Supporting Fig. S127**: Mass (HR‐ESI+) spectrum of **IC‐11** in CH_3_CN. **Supporting Fig. S128**: Molecular structure and labeling scheme of **IC‐1**. Hydrogen atoms were omitted for clarity. Displacement ellipsoids are drawn at the 50% probability level. **Supporting Fig. S129**: Molecular structure and labeling scheme of **IC‐2**. Hydrogen atoms were omitted for clarity. Displacement ellipsoids are drawn at the 50% probability level. **Supporting Fig. S130**: Molecular structure and labeling scheme of **IC‐4**. Hydrogen atoms were omitted for clarity. Displacement ellipsoids are drawn at the 50% probability level. **Supporting Fig. S131.** Molecular structure and labeling scheme of **IC‐8**. Hydrogen atoms were omitted for clarity. Displacement ellipsoids are drawn at the 50% probability level. **Supporting Fig. S132**: Molecular structure and labeling scheme of **IC‐10**. Hydrogen atoms were omitted for clarity. Displacement ellipsoids are drawn at the 50% probability level. **Supporting Fig. S133**: Cytotoxicity of non‐substituted *N*‐carboranyl isoquinolinones derivatives (**A**) **IC‐1**, (**B**) **IC‐2**, (**C**) **IC‐3**, (**D**) **IC‐4**, (**E**) **IC‐5** and (**F**) **IC‐6** toward MDCKII‐hABCG2 (black) and MDCKII‐WT cells (blue). MDCKII cells were treated with increasing concentrations of each examined compound in the mentioned concentrations for 48 h. Afterwards, cell viability was assessed by water‐soluble tetrazolium‐1 (WST‐1) assay. Data were normalized to solvent control (0.1% DMSO), which was set as 100% (mean ± SEM, N = 3, one‐way ANOVA with Holm‐Šidák post‐hoc test, * indicating significant difference in comparison to the solvent control, *** *p* ≤ 0.001, ** *p* ≤ 0.01, * *p* ≤ 0.05). **Supporting Fig. S134**: Cytotoxicity of *N*‐carboranyl 6,7‐dimethoxyisoquinolinones derivatives (**A**) **IC‐7**, (**B**) **IC‐8**, (**C**) **IC‐ 9**, (**D**) **IC‐10**, and (**E**) **IC‐11** toward MDCKII‐hABCG2 (black) and MDCKII‐WT cells (blue). MDCKII cells were treated with increasing concentrations of each investigational compound in the mentioned concentrations for 48 h. Afterwards, cell viability was assessed by water‐soluble tetrazolium‐1 (WST‐1) assay. Data were normalized to solvent control (0.1% DMSO), which was set as 100% (mean ± SEM, N = 3, one‐way ANOVA with Holm‐Šidák post‐hoc test, * indicating significant difference in comparison to the solvent control, *** *p* ≤ 0.001, ** *p* ≤ 0.01, * *p* ≤ 0.05). **Supporting Fig. S135**: Autofluorescence of unsubstituted *N*‐carboranyl isoquinolinones **IC‐1 – IC‐6** in (**A**) MDCKII‐ hABCG2 and (**B**) MDCKII‐WT cells. Cells were incubated with 0.5 µM and 1.0 µM of the **IC‐1 – IC‐6** for 4 h. Thereafter, cells were lysed and intracellular fluorescence was detected as described. Data are given as mean ± SEM (N = 3, one‐way ANOVA with Holm‐Šidák post‐hoc test, * significant difference in comparison to the solvent control: *p* ≤ 0.05). **Supporting Fig. S136**: Autofluorescence of *N*‐carboranyl 6,7‐dimethoxy isoquinolinones **IC‐7 – IC‐11** in (**A**) MDCKII‐ hABCG2 and (**B**) MDCKII‐WT cells. Cells were incubated with 0.5 µM and 1.0 µM of the **IC‐7 – IC‐11** for 4 h. Thereafter, cells were lysed and intracellular fluorescence was detected as described. Data are given as mean ± SEM (N = 3, one‐way ANOVA with Holm‐Šidák post‐hoc test, * significant difference in comparison to the solvent control: *p* ≤ 0.05). **Supporting Fig. S137**: Reversal of ABCG2‐mediated mitoxantrone resistance of unsubstituted *N*‐carboranyl isoquinolinones **IC‐1 – IC‐6** in MDCKII‐hABCG2 cells. Cells were treated with increasing concentration of MXN alone or in combination with 1.0 µM of (**A**) **IC‐1**, (**B**) **IC‐2**, (**C**) **IC‐3**, (**D**) **IC‐4**, (**E**) **IC‐5** and (**F**) **IC‐6** for 48 h. Afterwards, cell viability was determined by WST‐1 assay. Data were normalized to solvent control (0.1% DMSO) and were shown as mean ± SEM (N = 4, one‐way ANOVA with Holm‐Šidák post‐hoc test, # significant different to MXN alone, ### *p* ≤ 0.001, ## *p* ≤ 0.01, # *p* ≤ 0.05). **Supporting Fig. S138**: Reversal of ABCG2‐mediated mitoxantrone resistance of *N*‐carboranyl 6,7‐dimethoxy isoquinolinones **IC‐7 – IC‐11** in MDCKII‐hABCG2 cells. Cells were treated with increasing concentration of MXN alone or in combination with 1.0 µM of (**A**) **IC‐7**, (**B**) **IC‐8**, (**C**) **IC‐9**, (**D**) **IC‐10** and (**E**) **IC‐11** for 48 h. Afterwards, cell viability was determined by WST‐1 assay. Data were normalized to solvent control (0.1% DMSO) and were shown as mean ± SEM (N = 4, one‐way ANOVA with Holm‐Šidák post‐hoc test, # significant different to MXN alone, ### *p* ≤ 0.001, ## *p* ≤ 0.01). **Supporting Fig. S139**: (**A**) Cartoon representation of ABCG2, with monomers depicted in rose and blue. Top‐ranked binding poses of **IC‐3** (green) and **IC‐4** (yellow) within the lateral cavity S2 and the inner cavity S1, respectively. (**B**) Comparison of the highest scoring simulated poses of **IC‐4** (yellow), **IC‐5** (blue) and **IC‐6** (olive); structures shown as stick model; hydrogen atoms omitted for clarity. (**C**) 2D interaction diagram of the top‐score poses of **IC‐10**; blue hexagon represents the carboranyl moiety. (**D**) Docking of **IC‐4** (yellow), **IC‐5** (blue), **IC‐6** (olive), **IC‐ 10** and **IC‐11** (violet) into the crystal structure of ABCG2 (5NJ3) in comparison with mitoxantrone (red). **Supporting Table S1**: Fundamental structure parameters. **Supporting Table S2**: Free binding energies of compounds **IC‐1** to **IC‐11** towards human ABCG2 transporter in rigid ABCG2 protein (PDB code 5NJ3).

## Funding

This work was supported by Deutsche Forschungsgemeinschaft (He 1376/54‐1), European Chiropractors' Union (PNRR‐III‐C9‐2023‐I8‐CF76), and Graduate School Leipzig School of Natural Sciences – Building with Molecules and Nano‐objects (BuildMoNa).

## Conflicts of Interest

The authors declare no conflicts of interest.

## Supporting information

Supplementary Material
