# Supplementary material for: Polymethoxylated N‐Carboranyl Isoquinolinones: A New Scaffold for ABCG2 Inhibitors
Source: ChemMedChem. 2026 Feb 12;21(3):e202500708. doi: 10.1002/cmdc.202500708 (PMC12900506; doi:10.1002/cmdc.202500708)
Supplement: Supplementary file 1 — Supplementary Material [file CMDC-21-e202500708-s001.pdf]

Supporting Information:

Polymethoxylated *N*-Carboranyl Isoquinolinones – A New Scaffold for ABCG2 Inhibitors.

Lydia Kuhnert,*[a], Philipp Stockmann,[b] Peter Lönnecke,[b] Mara Anna Wolniewicz,[b] Evamarie Hey-Hawkins*[c,d] and Walther Honscha*[a]

[a] Dr. L. Kuhnert (LK, https://orcid.org/0000-0002-9779-2440)*, Prof. Dr. W. Honscha (WH, https://orcid.org/0000-0001-5954-0659)*
Institute of Pharmacology, Pharmacy and Toxicology, Faculty of Veterinary Medicine, Universität Leipzig, An den Tierkliniken 15, 04103 Leipzig, Germany
Phone: (+49)341-9738142
E-mail: lydia.kuhnert@vetmed.uni-leipzig.de, honscha@vetmed.uni-leipzig.de

[b] Dr. P. Stockmann (PS, http://orcid.org/0000-0002-4174-8539), Dr. P. Lönnecke (PL, https://orcid.org/0000-0003-1335-0897), M. A. Wolniewicz (MAW)
Institute of Inorganic Chemistry, Faculty of Chemistry and Mineralogy, Universität Leipzig, Johannisallee 29, 04103 Leipzig, Germany
Phone: (+49)341-9736151

[c,d] Prof. Dr. E. Hey-Hawkins (EHH, http://orcid.org/0000-0003-4267-0603)
Centre for Biotechnology and Biomedicine (BBZ), Faculty of Chemistry and Mineralogy, Institute of Bioanalytical Chemistry, Universität Leipzig, Deutscher Platz 5, 04103 Leipzig, Germany
Faculty of Chemistry and Chemical Engineering, Department of Chemistry, Babeș-Bolyai University, Str. Arany Janos Nr. 11, RO-400028 Cluj-Napoca, Romania
E-mail: evamarie.hey@ubbcluj.ro

This PDF file includes

1. **NMR spectra of compounds 2, 4, 5, 6a-e, 7a-d, IC-1 to IC-12**
   (Figures S1 to S105)
2. **MS spectra**
   (Figures S106 to S127)
3. **X-ray Crystallography: Molecular structures of IC-1, IC-2, IC-4, IC-8 and IC-10**
   (Figures S128 to S132; Table S1)
4. **Biological Data**
   (Figures S133 to S138)
5. **Molecular Docking**
   (Supplementary Text S1, Figure S139, Table S2)
6. **References**

1. **NMR spectra of compounds 2, 4, 5, 6a-e, 7a-d, IC-1 to IC-11**

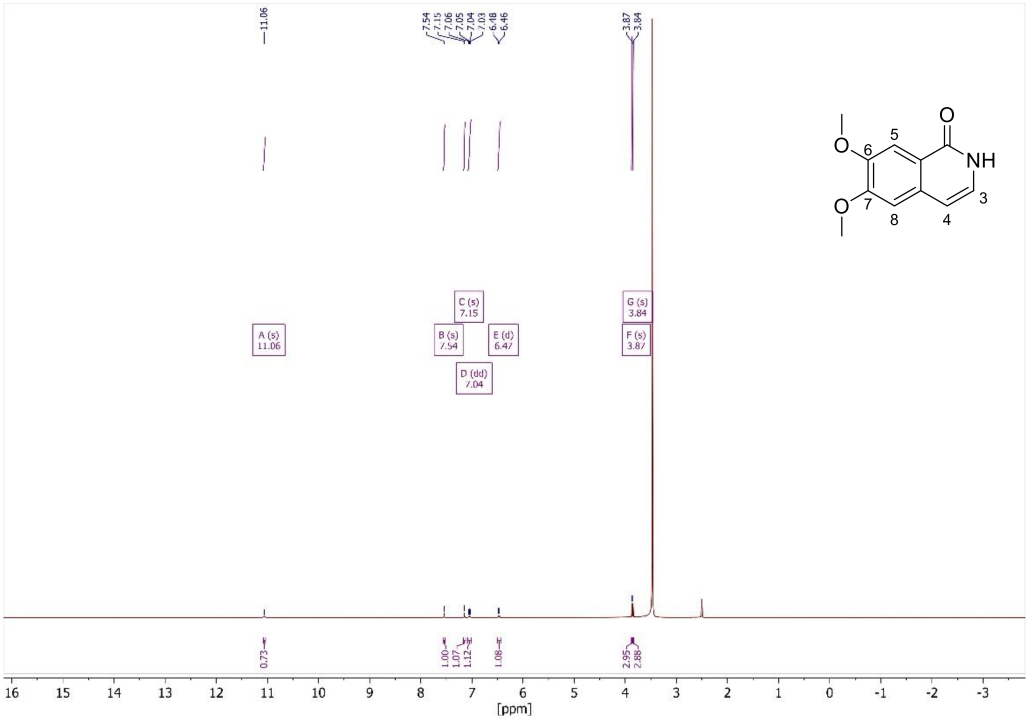

Figure S1. ^{1}H NMR spectrum of **2** in DMSO-d_6.

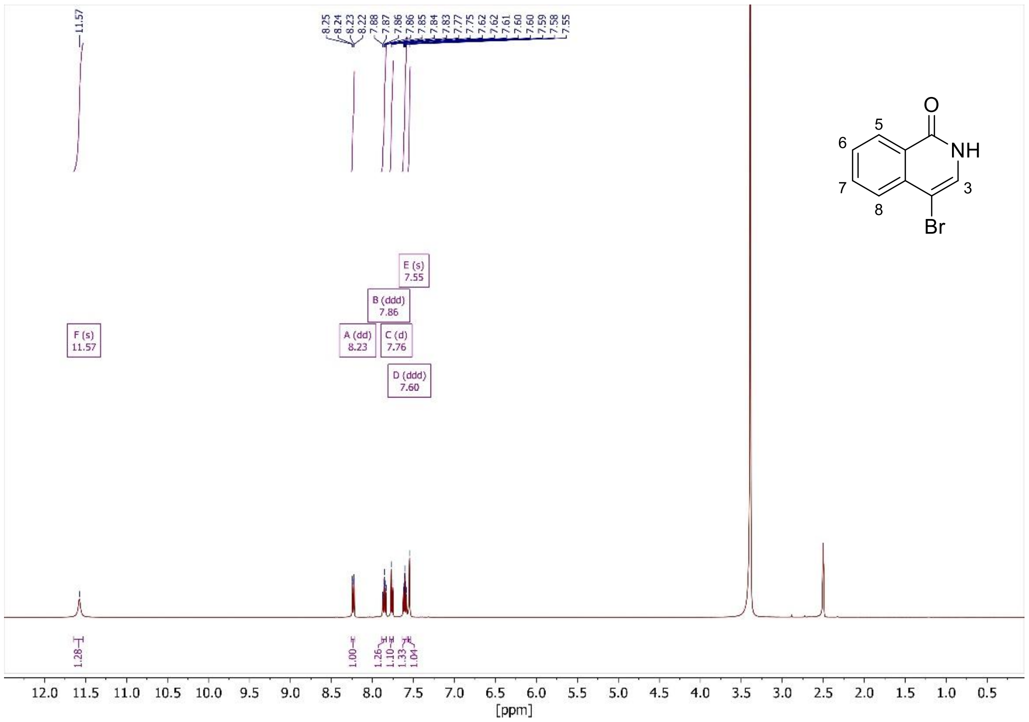

Figure S2. ^{1}H NMR spectrum of **4** in DMSO-d_6.

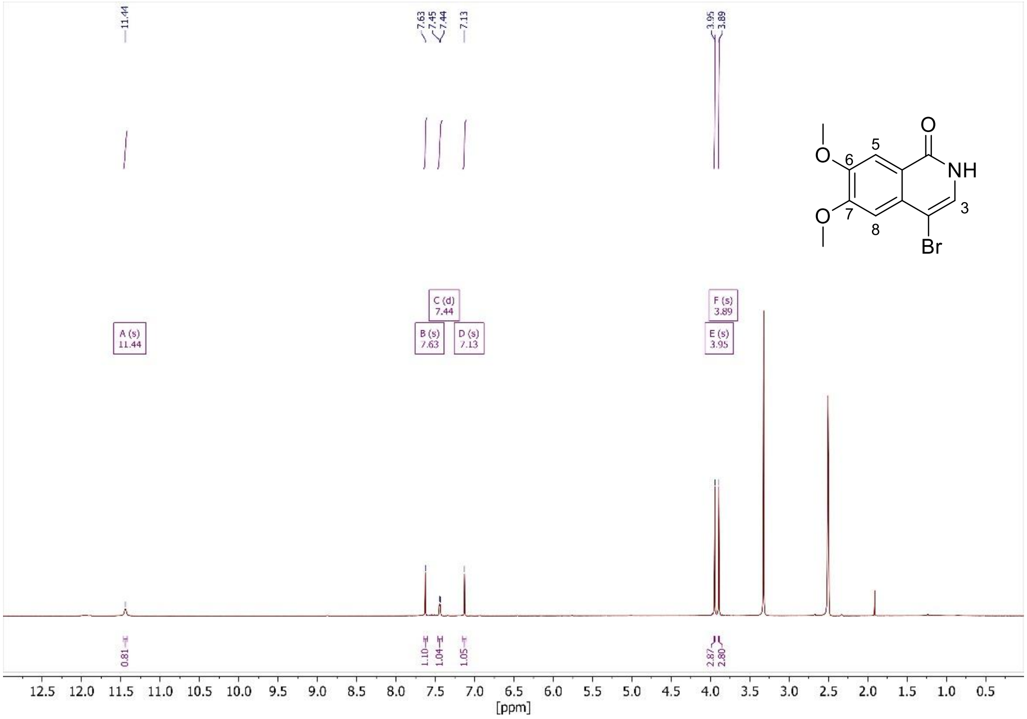

Figure S3. ^{1}H NMR spectrum of **5** in DMSO-d_6.

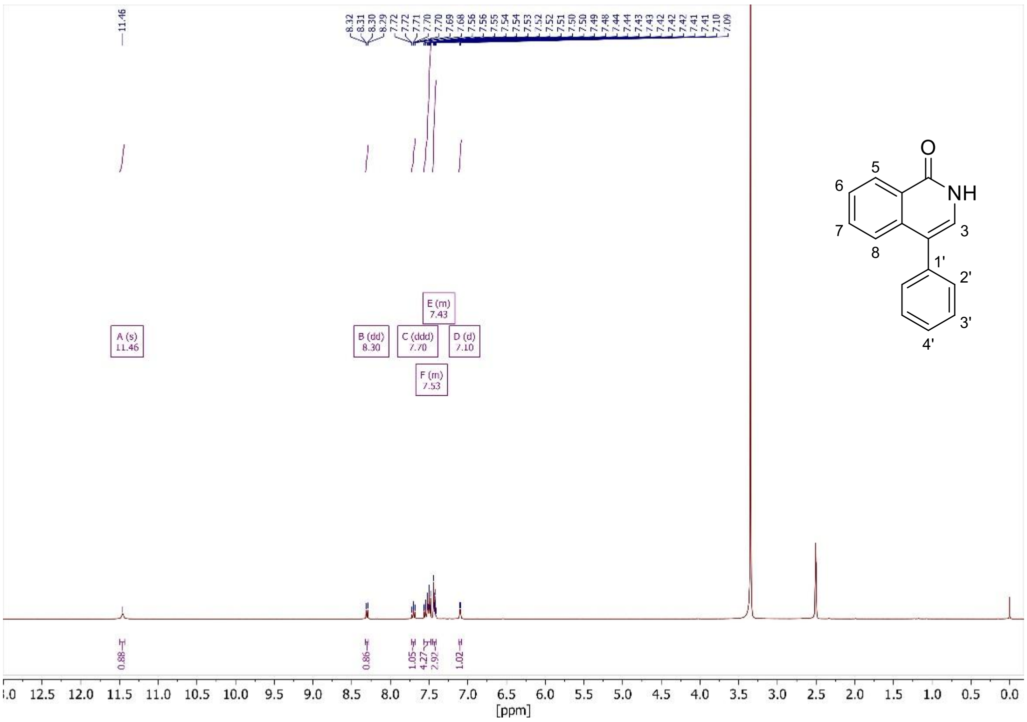

Figure S4. ^{1}H NMR spectrum of **6a** in DMSO-d_6.

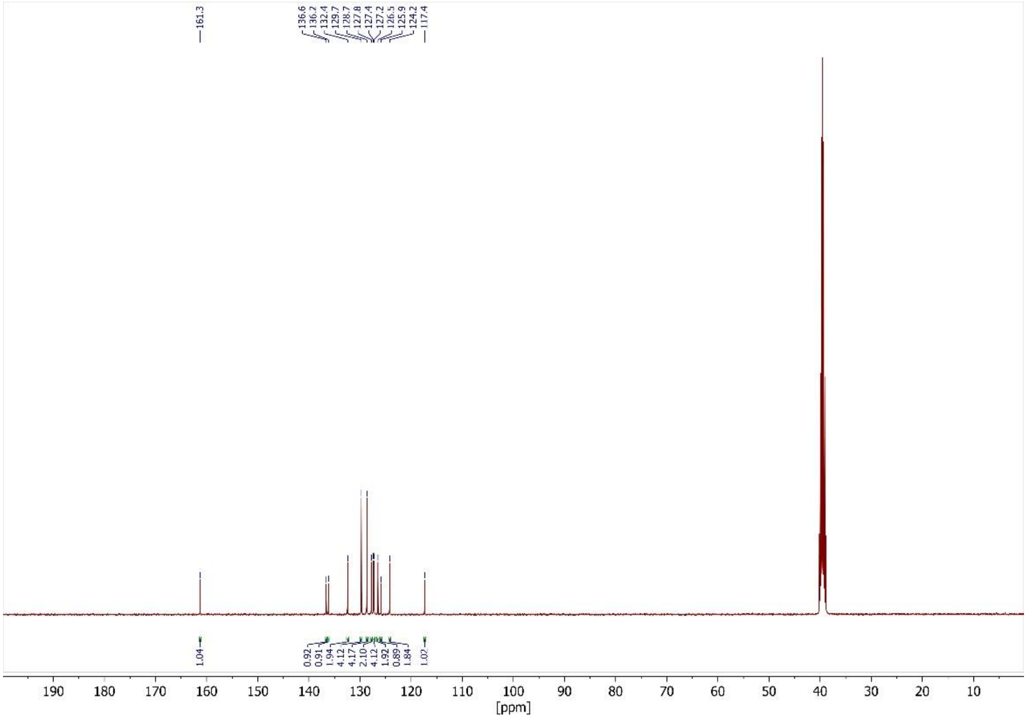

Figure S5. $^{13}C\{^{1}H\}$ NMR spectrum of **6a** in DMSO-d_6.

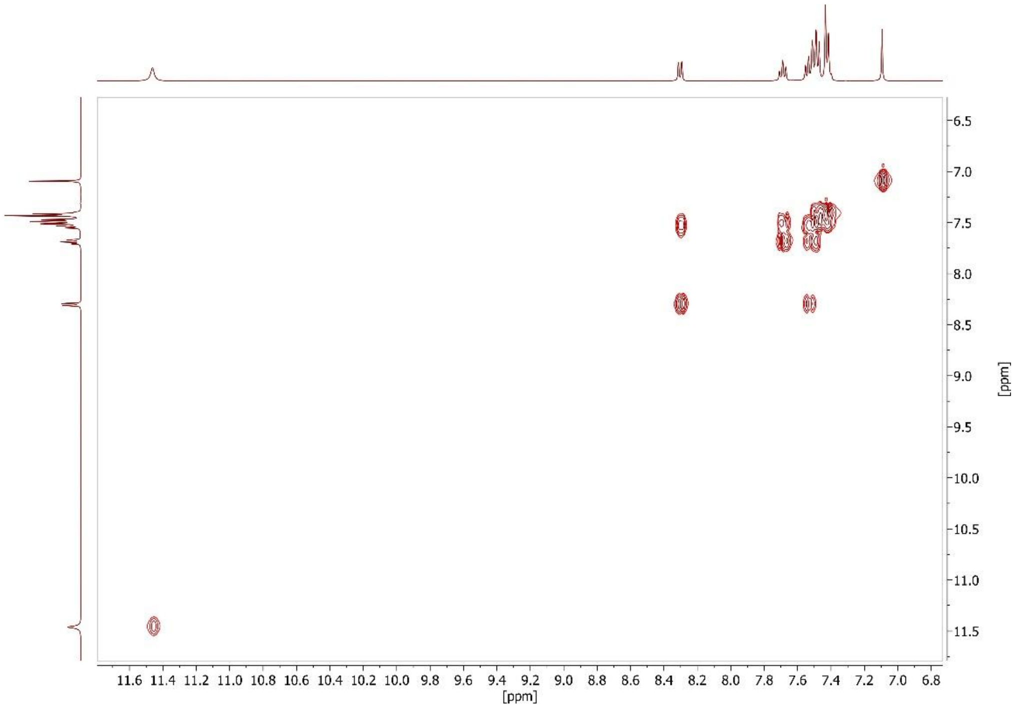

Figure S6. ^{1}H-COSY NMR spectrum of **6a** in DMSO-d_6.

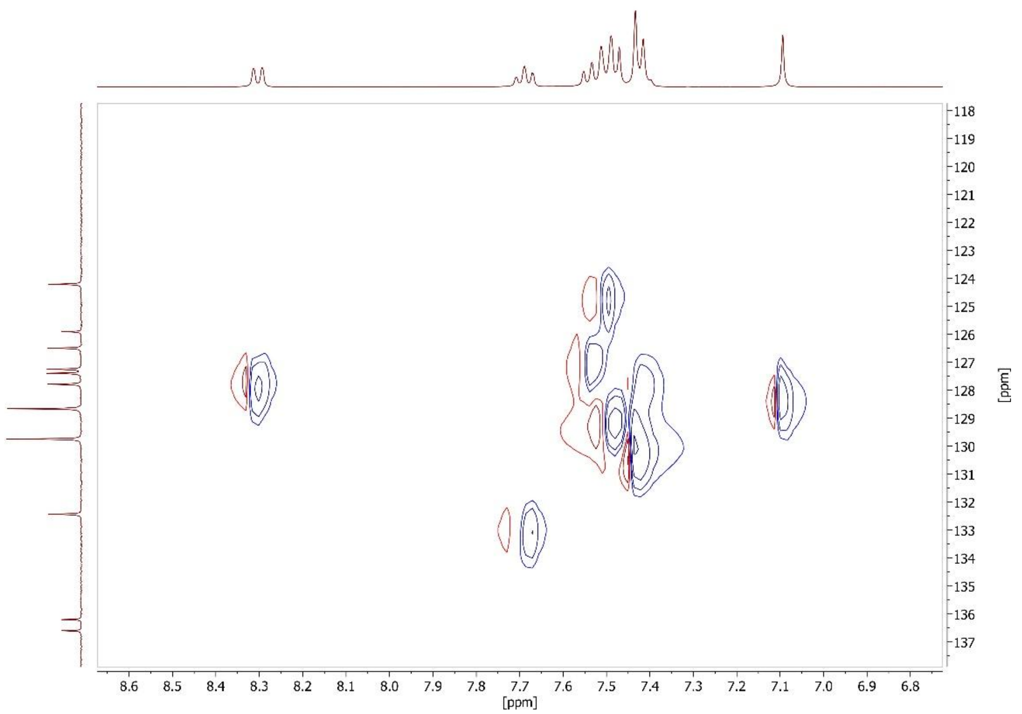

Figure S7. HSQC NMR spectrum of **6a** in DMSO d_6.

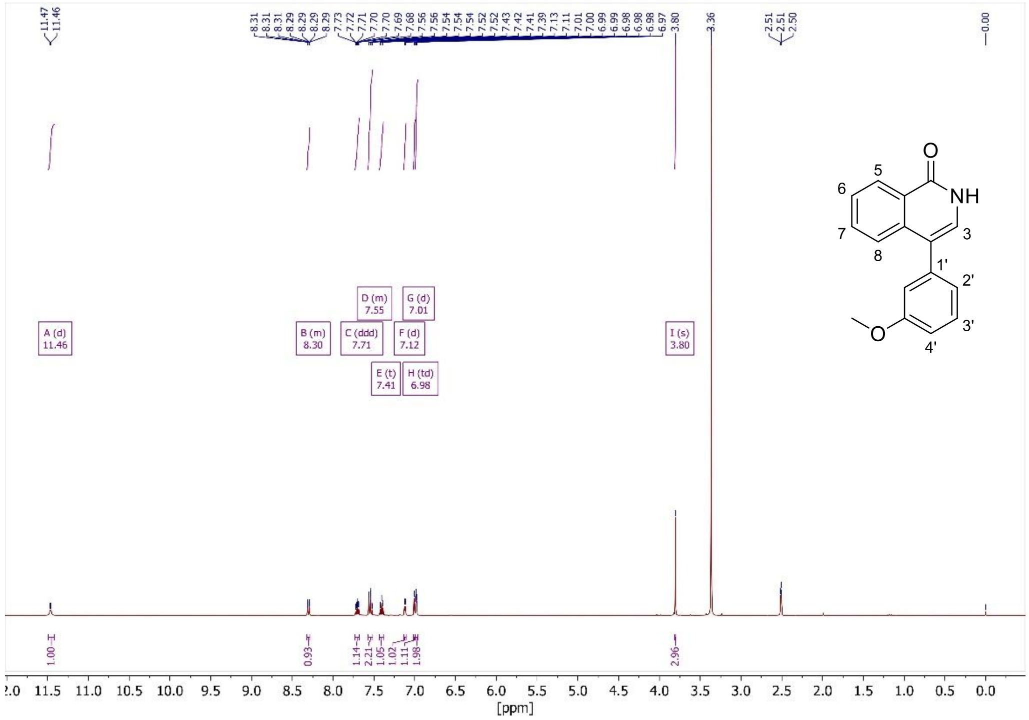

Figure S8. ^{1}H NMR spectrum of **6b** in DMSO-d_6.

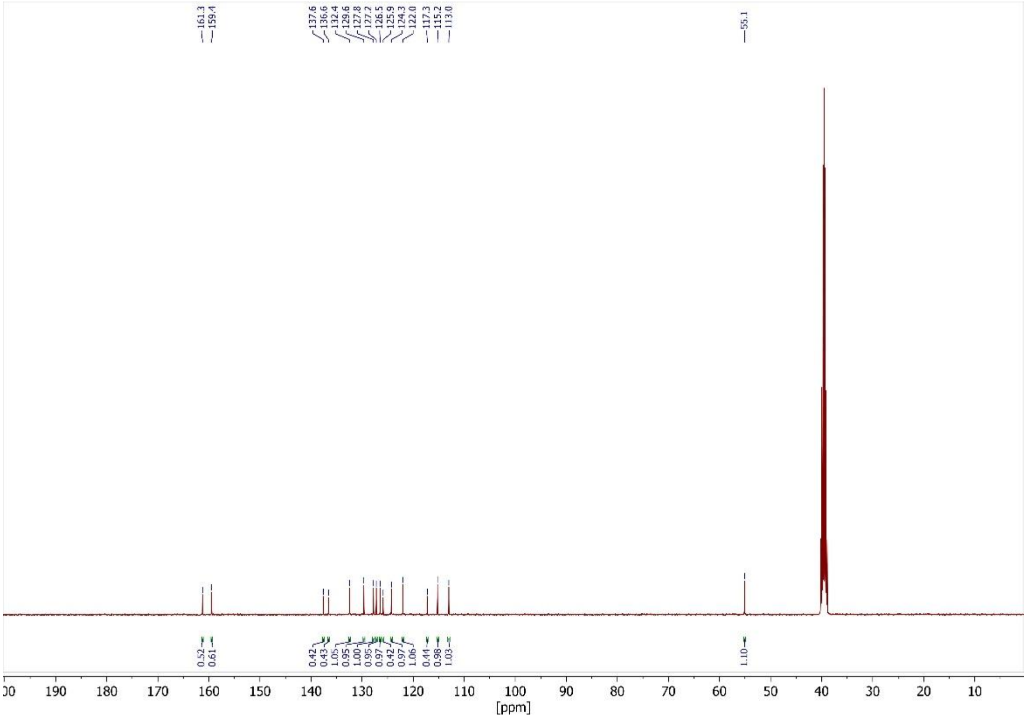

Figure S9. $^{13}C\{^{1}H\}$ NMR spectrum of **6b** in DMSO-d_6.

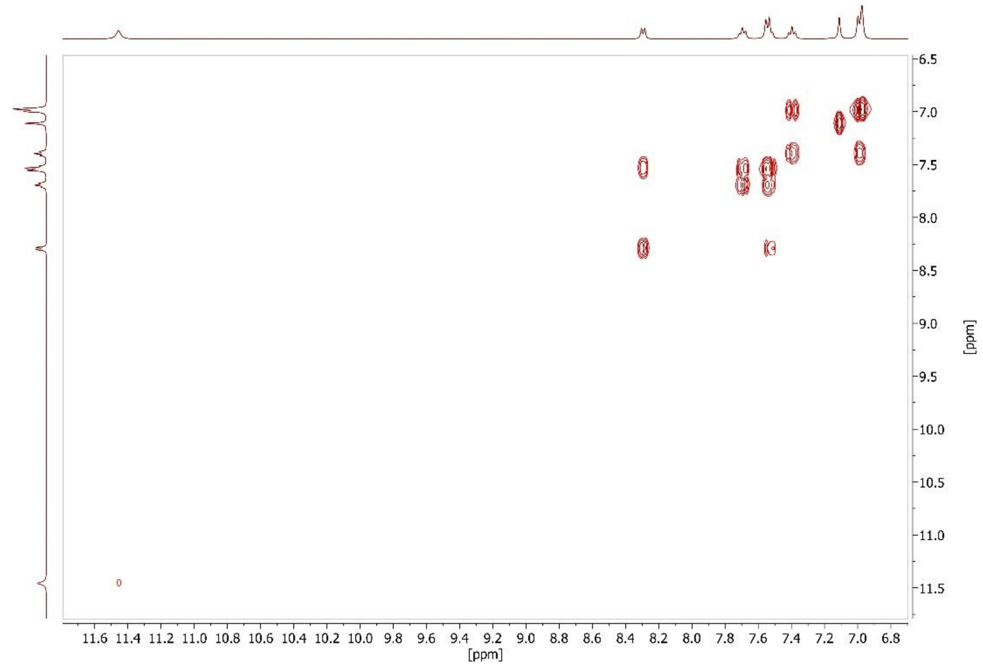

Figure S10. ^{1}H-COSY NMR spectrum of **6b** in DMSO-d_6.

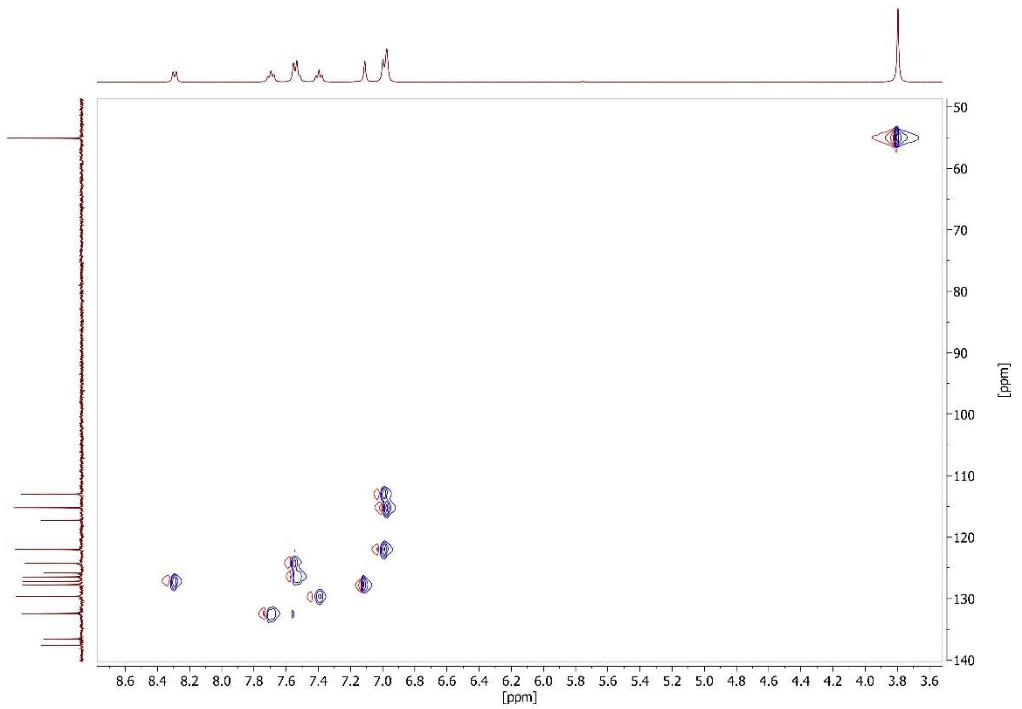

Figure S11. HSQC NMR spectrum of **6b** in DMSO-d_6.

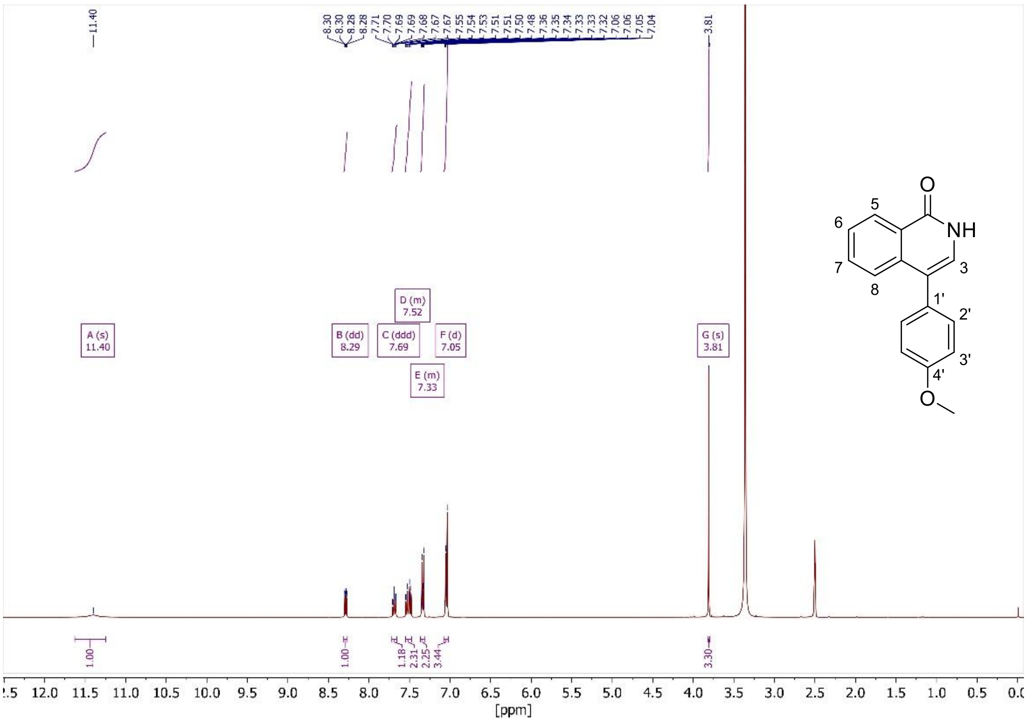

Figure S12. ^{1}H NMR spectrum of **6c** in DMSO-d_6.

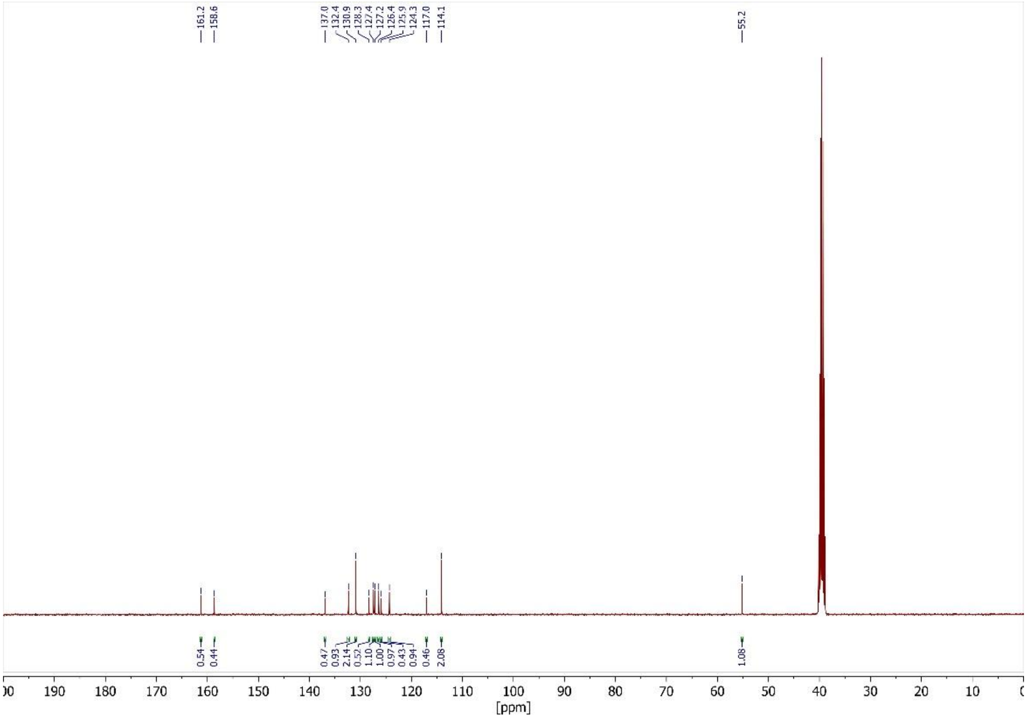

Figure S13. $^{13}C\{^{1}H\}$ NMR spectrum of **6c** in DMSO-d_6.

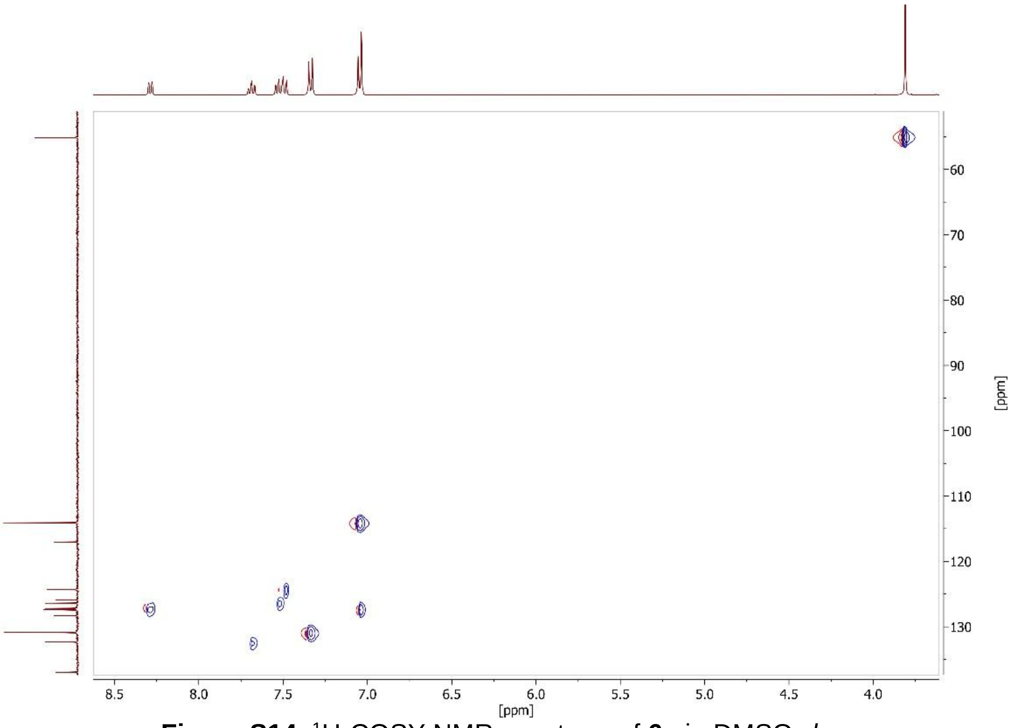

Figure S14. ^{1}H-COSY NMR spectrum of **6c** in DMSO-d_6.

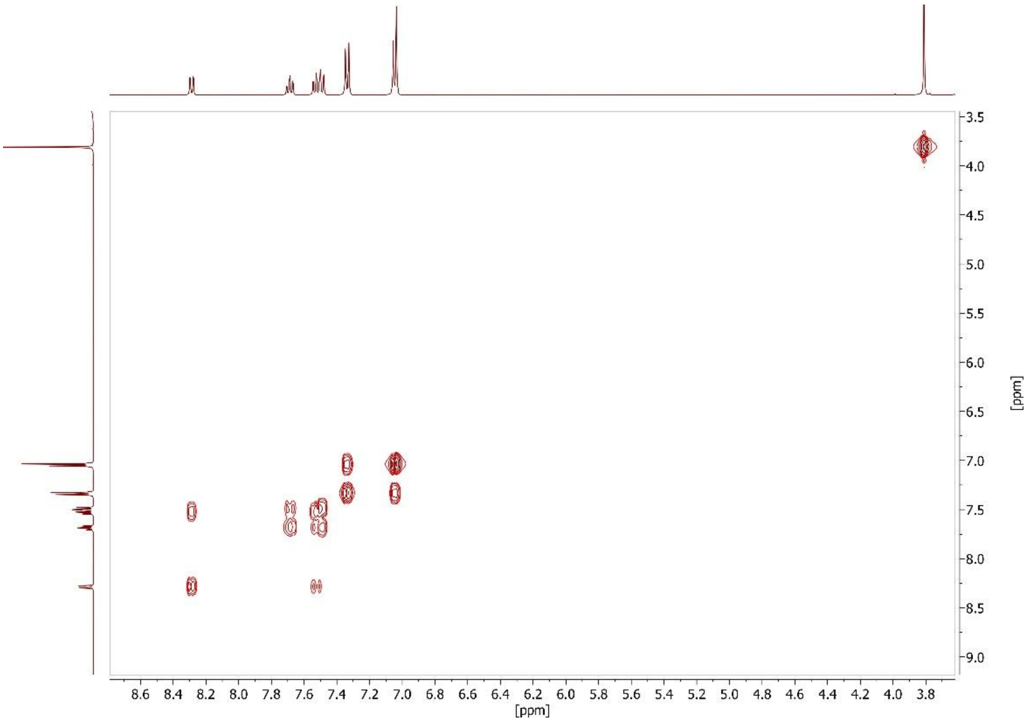

Figure S15. HSQC NMR spectrum of **6c** in DMSO-d_6.

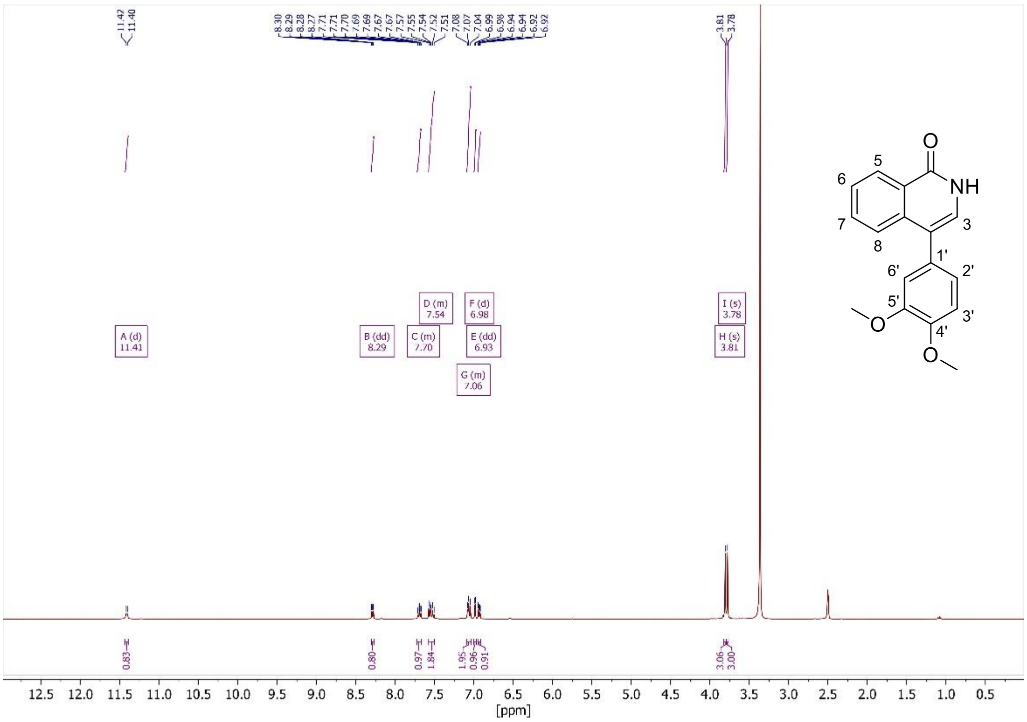

Figure S16. ^{1}H NMR spectrum of **6d** in DMSO-d_6.

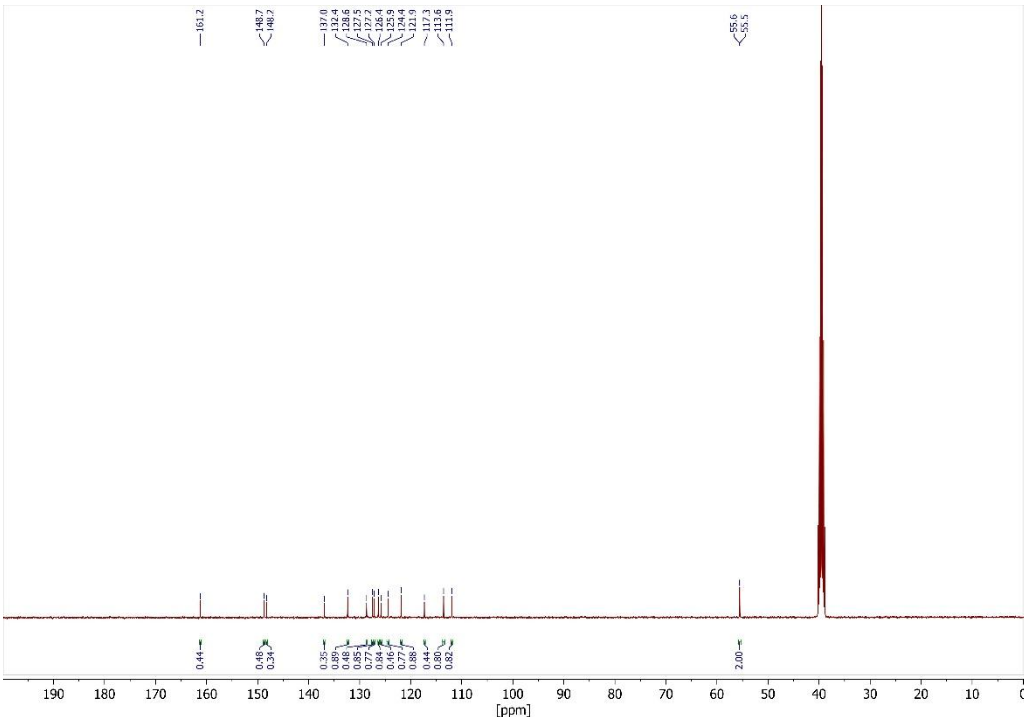

Figure S17. $^{13}C\{^{1}H\}$ NMR spectrum of **6d** in DMSO-*d*$_6$.

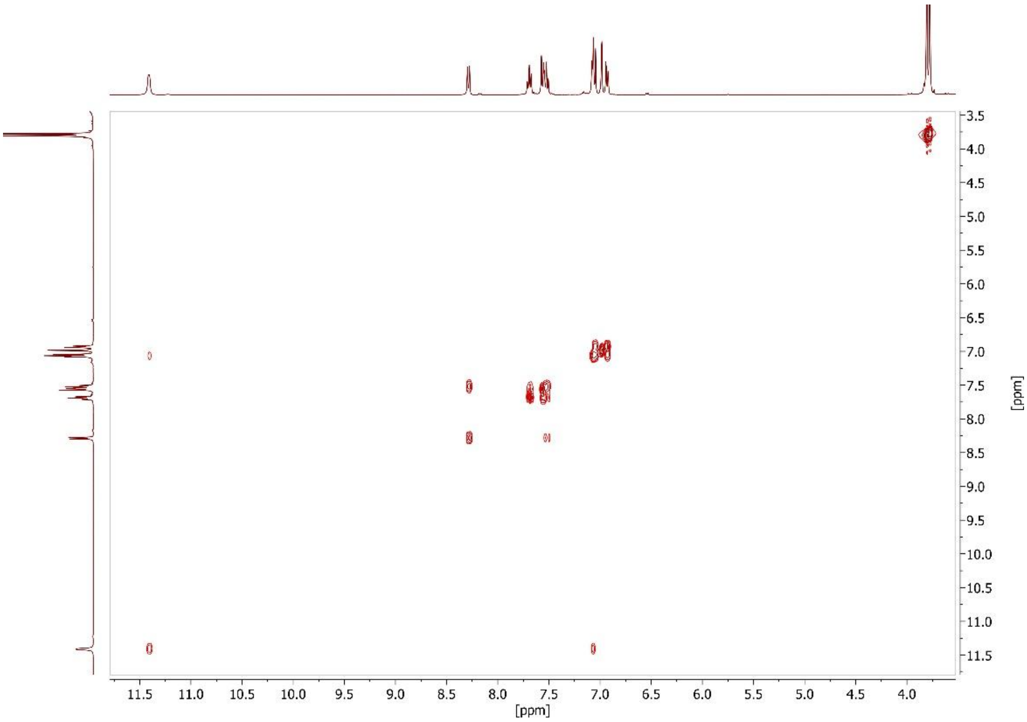

Figure S18. ^{1}H-COSY NMR spectrum of **6d** in DMSO-*d*$_6$.

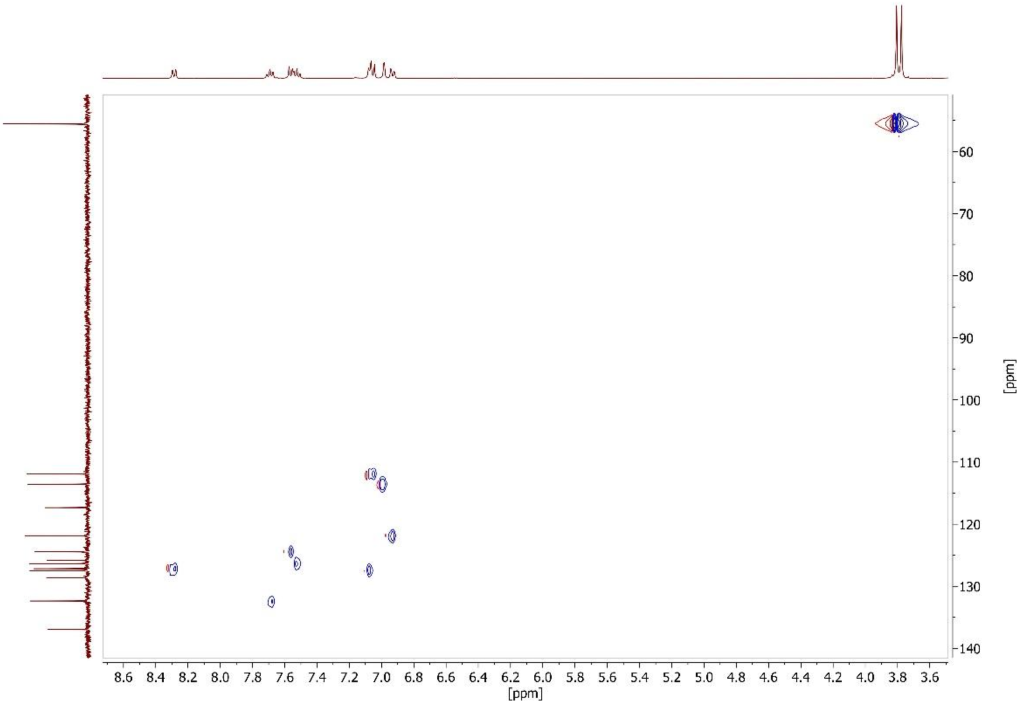

Figure S19. HSQC NMR spectrum of **6d** in DMSO-d_6.

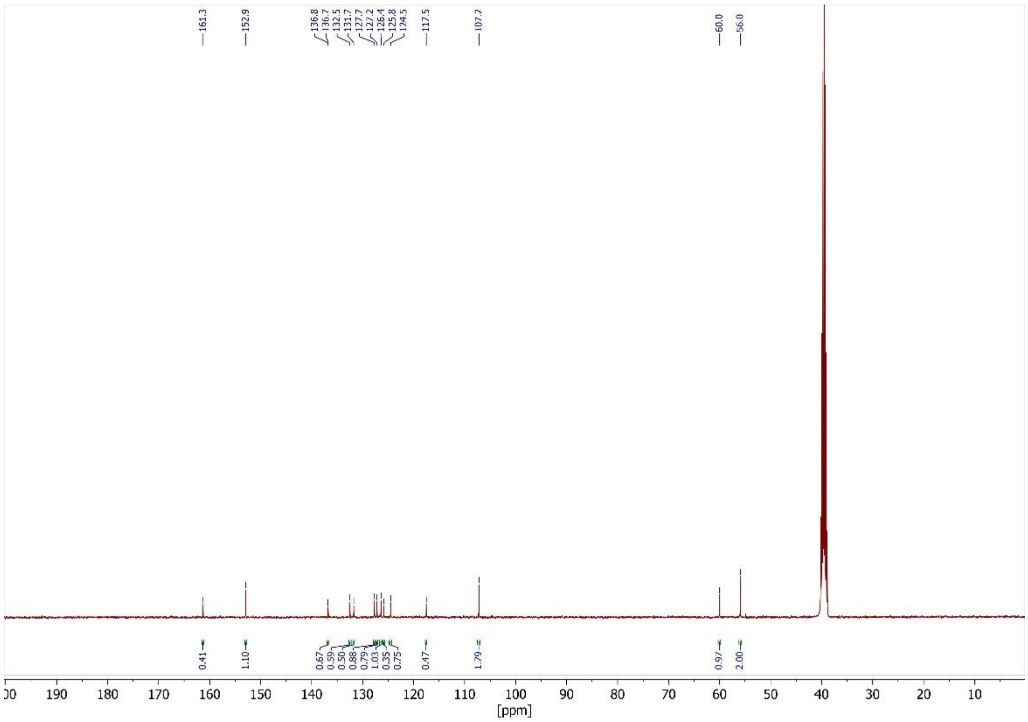

Figure S20. ^{1}H NMR spectrum of **6e** in DMSO-d_6.

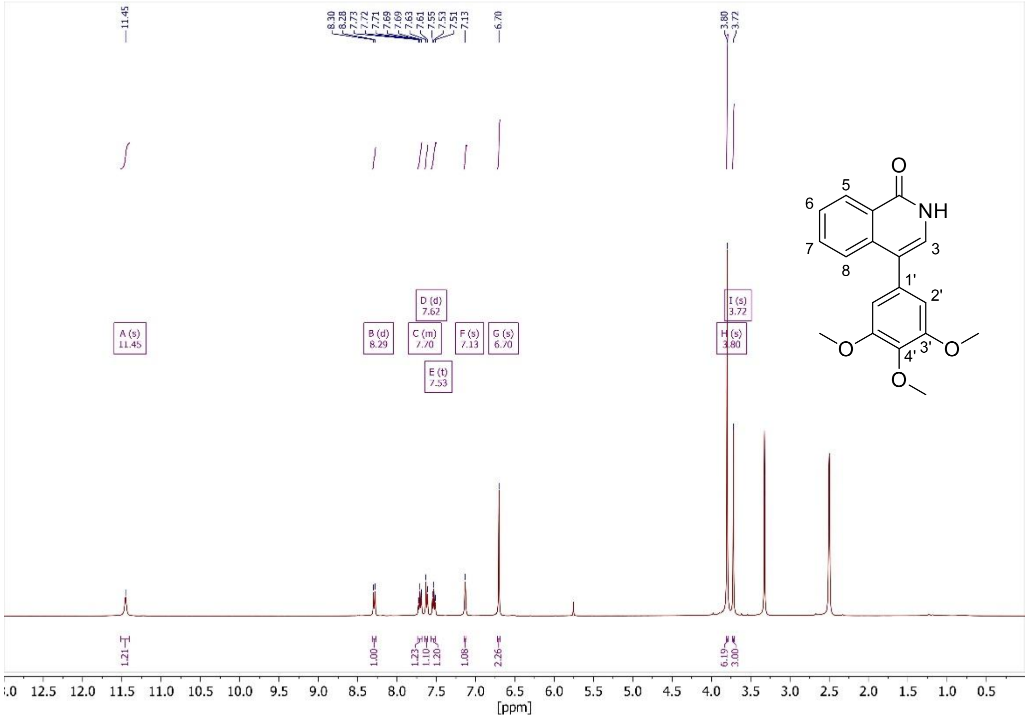

Figure S21. $^{13}C\{^1H\}$ NMR spectrum of **6e** in DMSO-d_6.

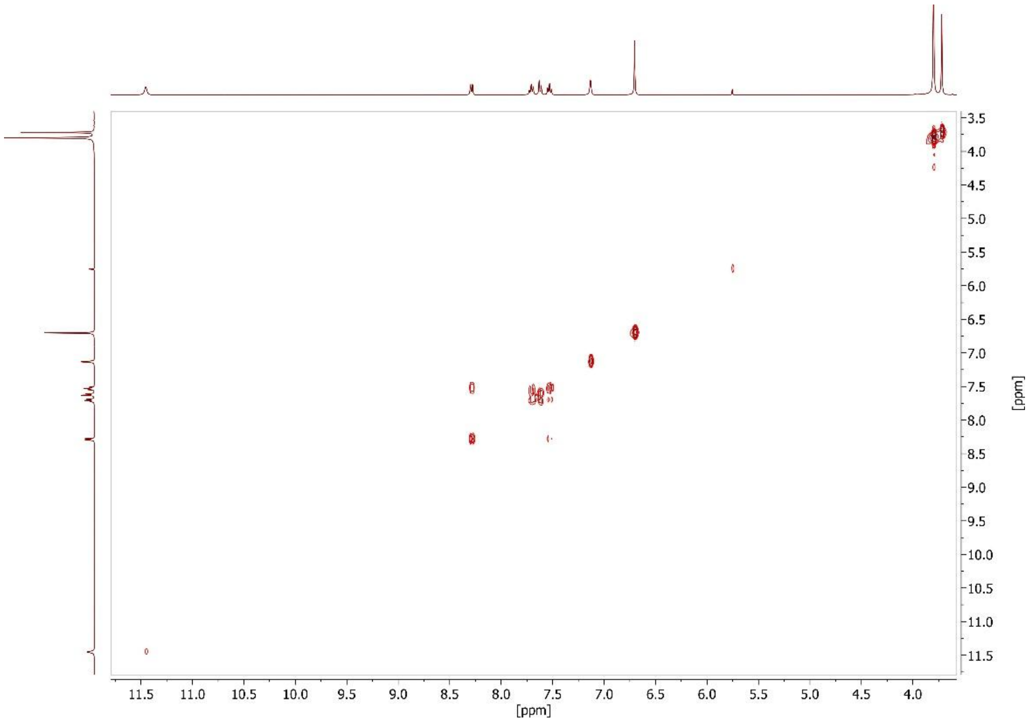

Figure S22. ^1H-COSY NMR spectrum of **6e** in DMSO-d_6.

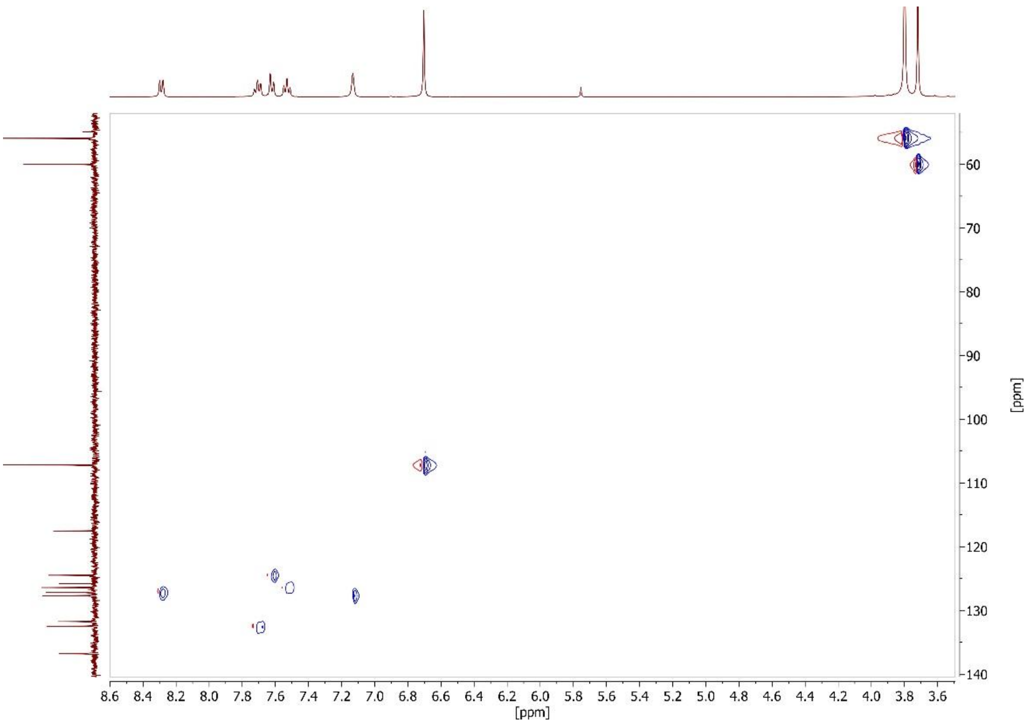

Figure S23. HSQC NMR spectrum of **6e** in DMSO-d_6.

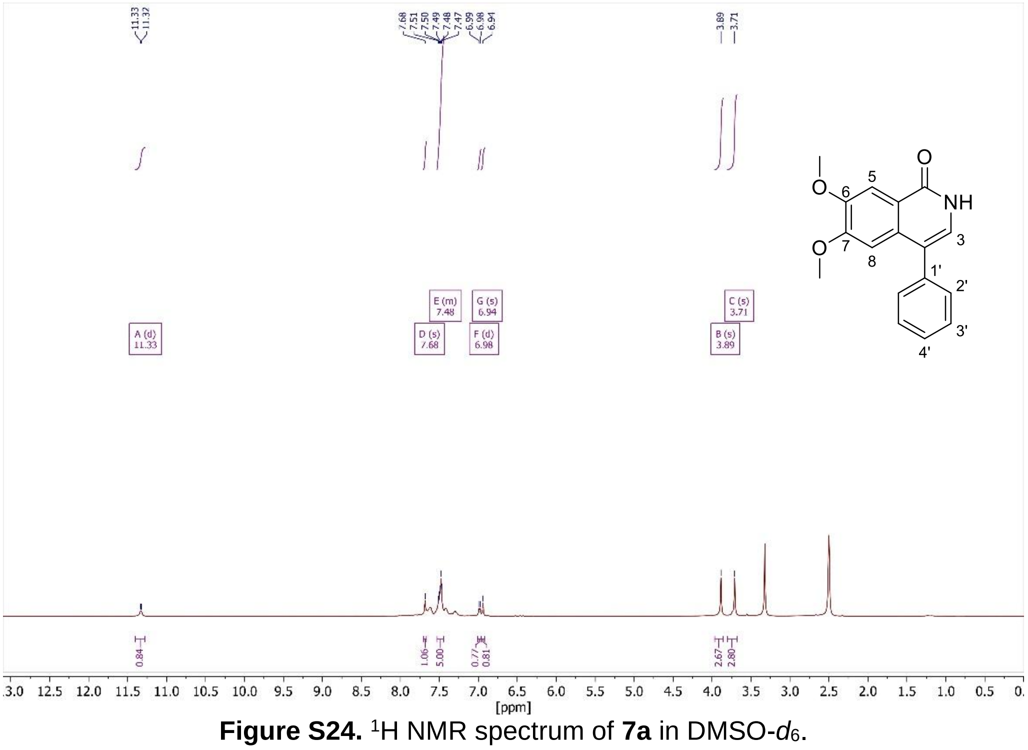

Figure S24. ^{1}H NMR spectrum of **7a** in DMSO-d_6.

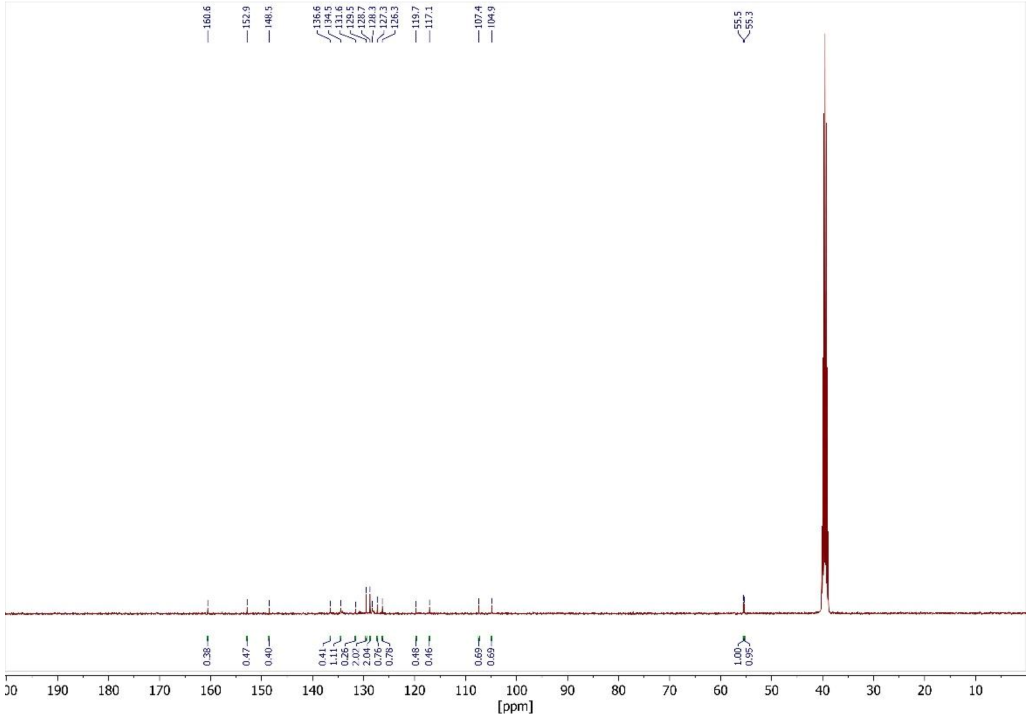

Figure S25. $^{13}C\{^{1}H\}$ NMR spectrum of **7a** in DMSO-d_6.

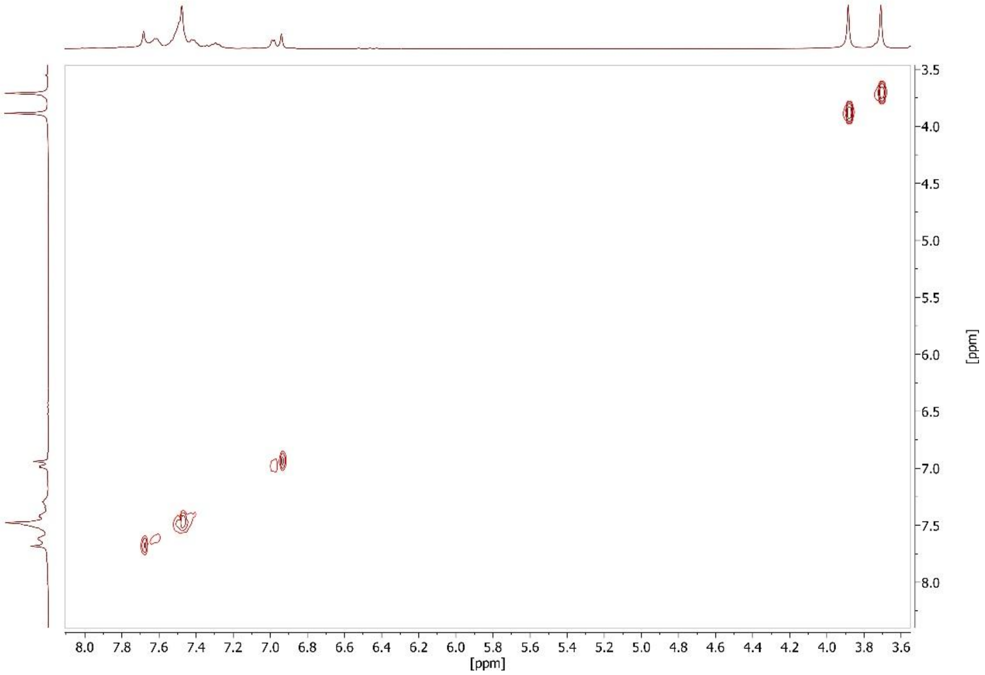

Figure S26. ^{1}H-COSY NMR spectrum of **7a** in DMSO-d_6.

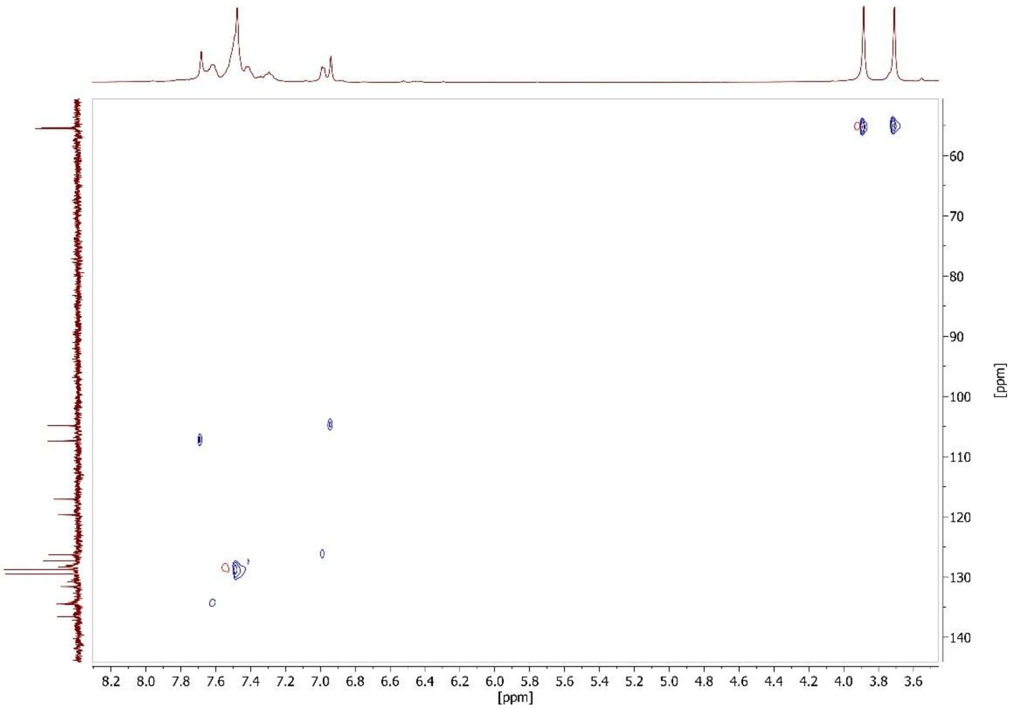

Figure S27. HSQC NMR spectrum of **7a** in DMSO-d_6.

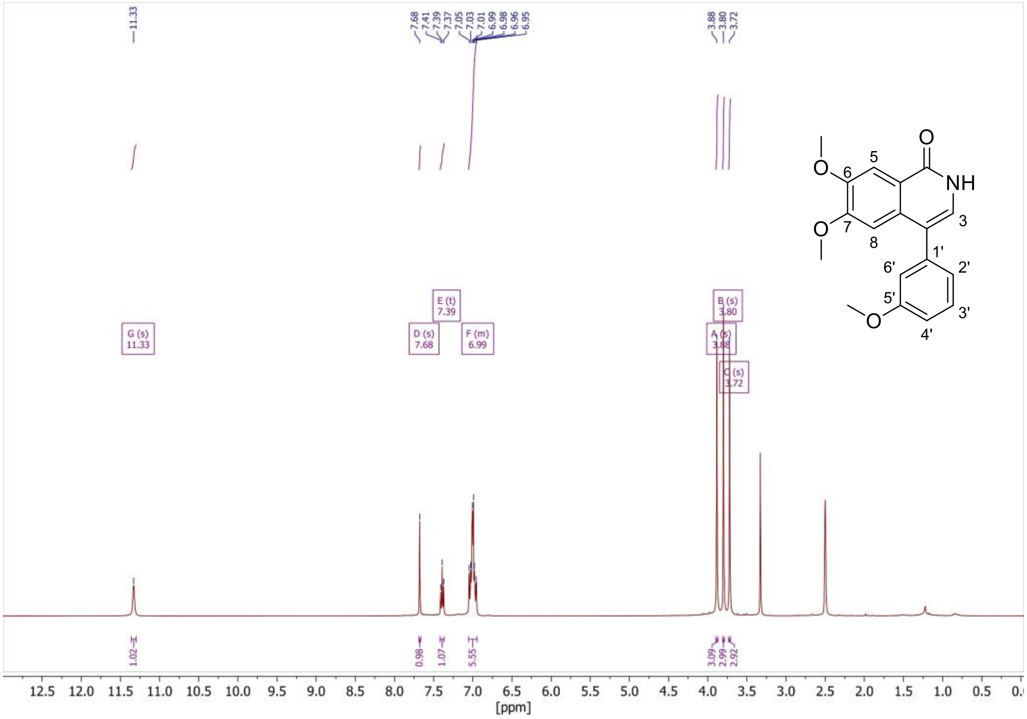

Figure S28. ^{1}H NMR spectrum of **7b** in DMSO-d_6.

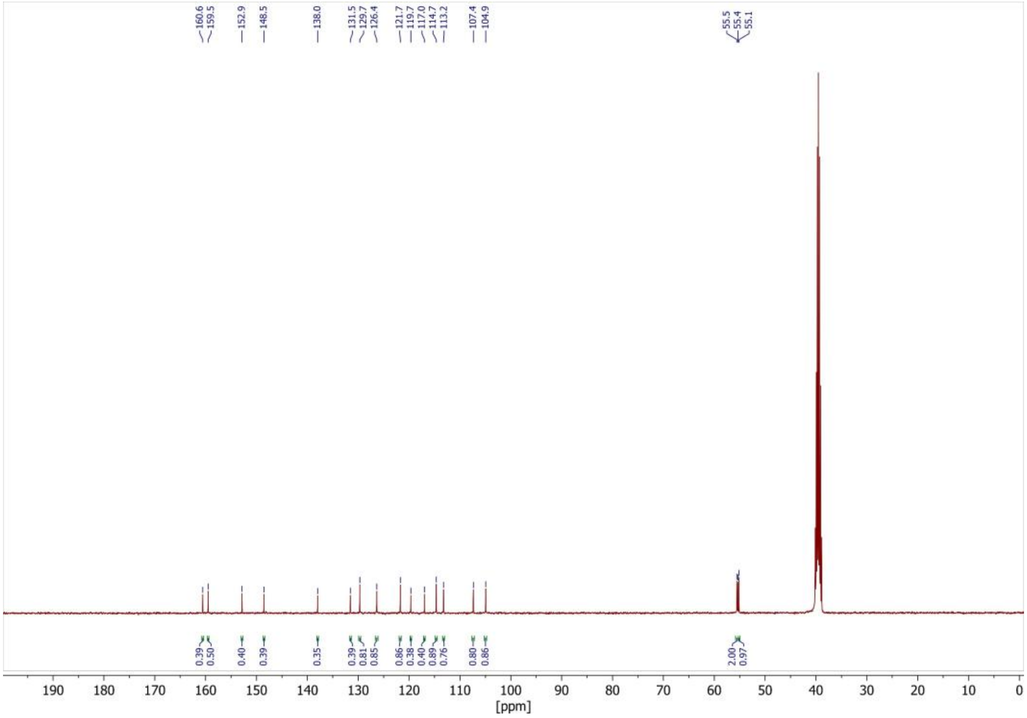

Figure S29. $^{13}C\{^{1}H\}$ NMR spectrum of **7b** in DMSO-d_6.

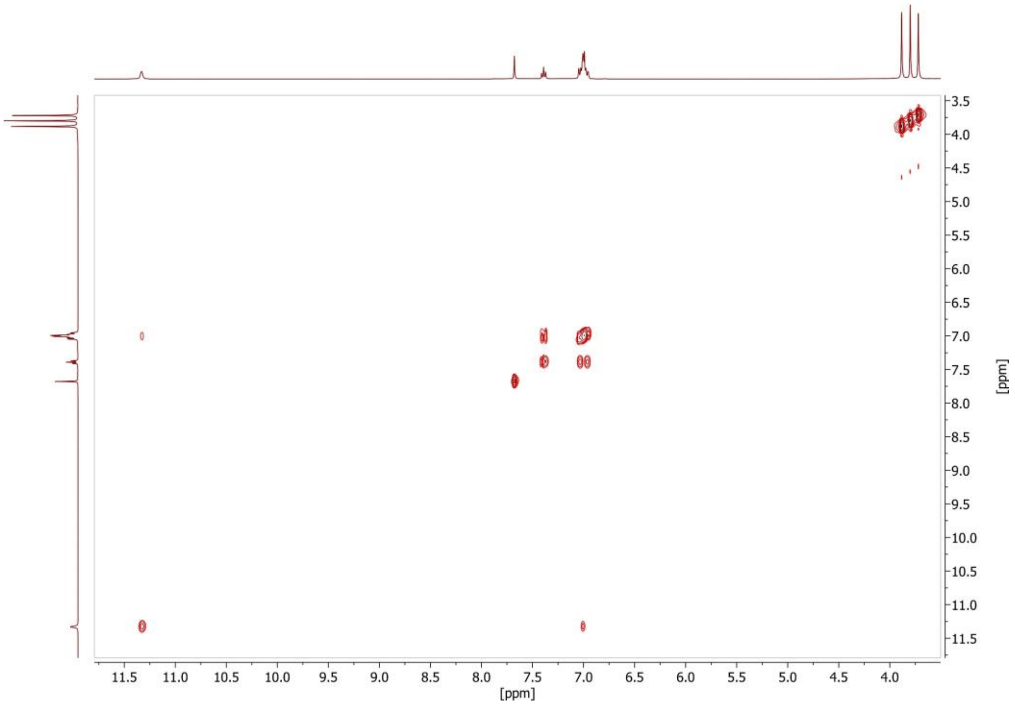

Figure S30. ^{1}H-COSY NMR spectrum of **7b** in DMSO-d_6.

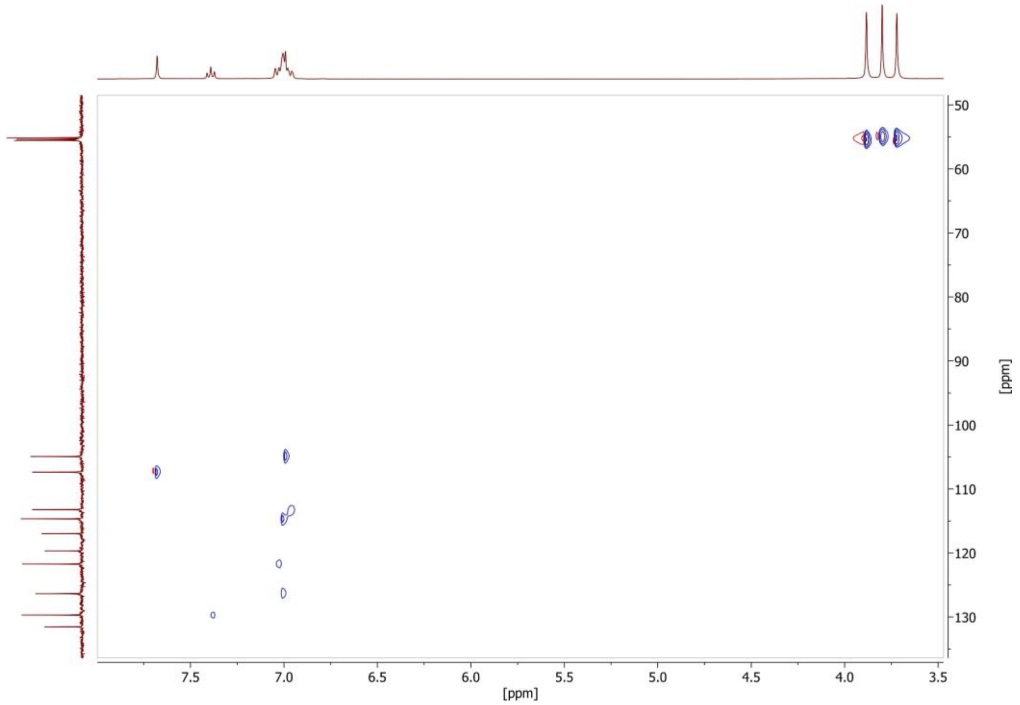

Figure S31. HSQC NMR spectrum of **7b** in DMSO-d_6.

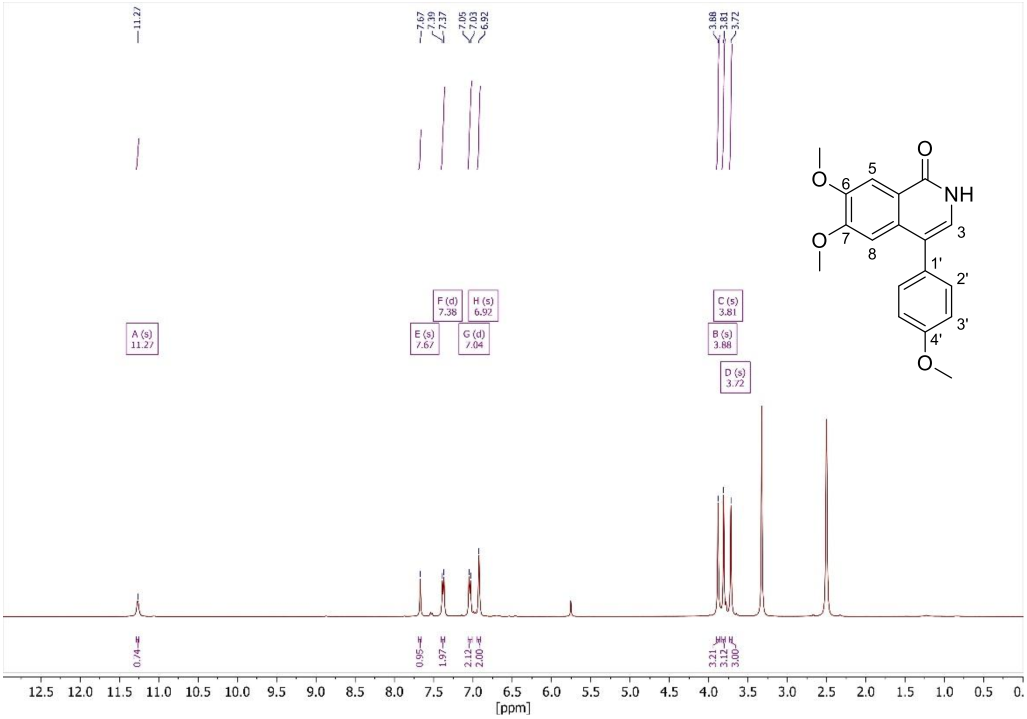

Figure S32. ^{1}H NMR spectrum of **7c** in DMSO-d_6.

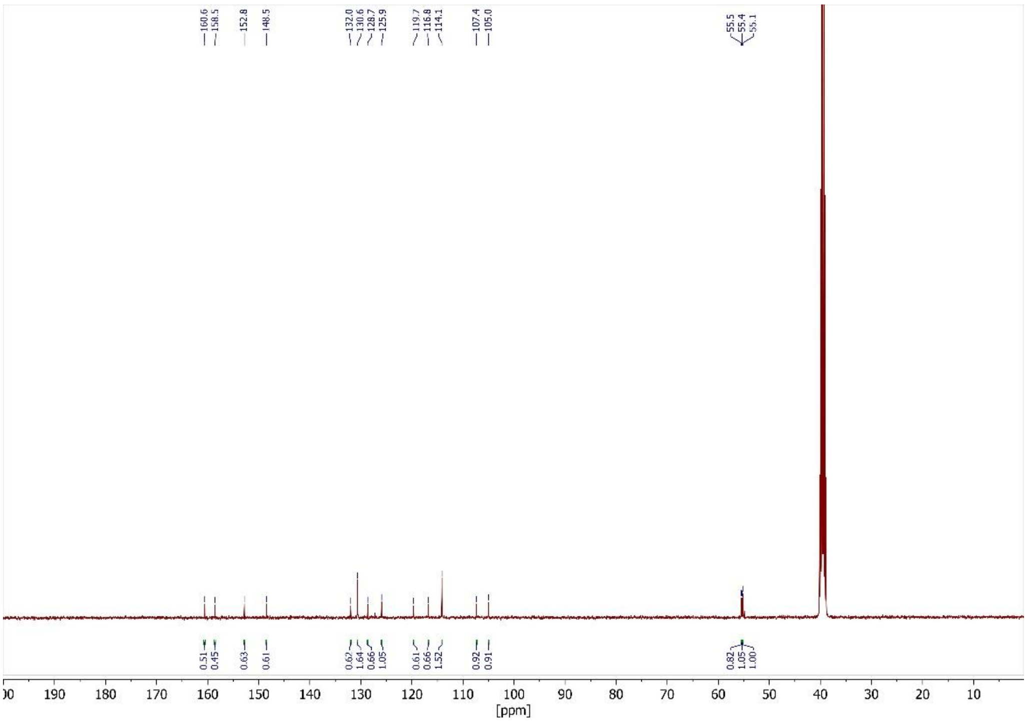

Figure S33. $^{13}C\{^{1}H\}$ NMR spectrum of **7c** in DMSO-d_6.

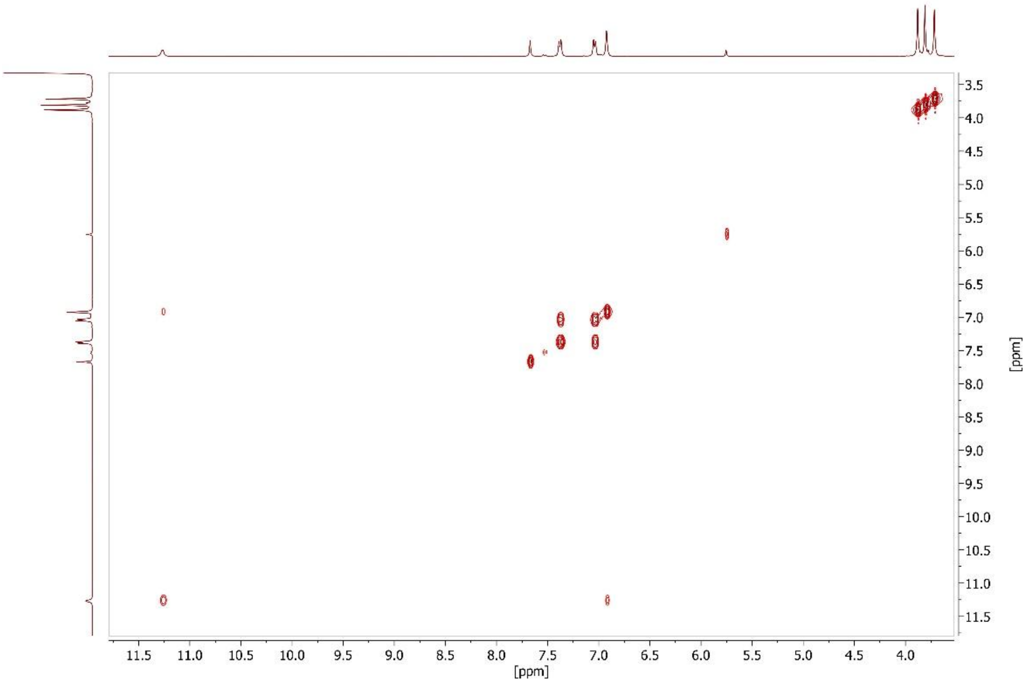

Figure S34. ^{1}H-COSY NMR spectrum of **7c** in DMSO-d_6.

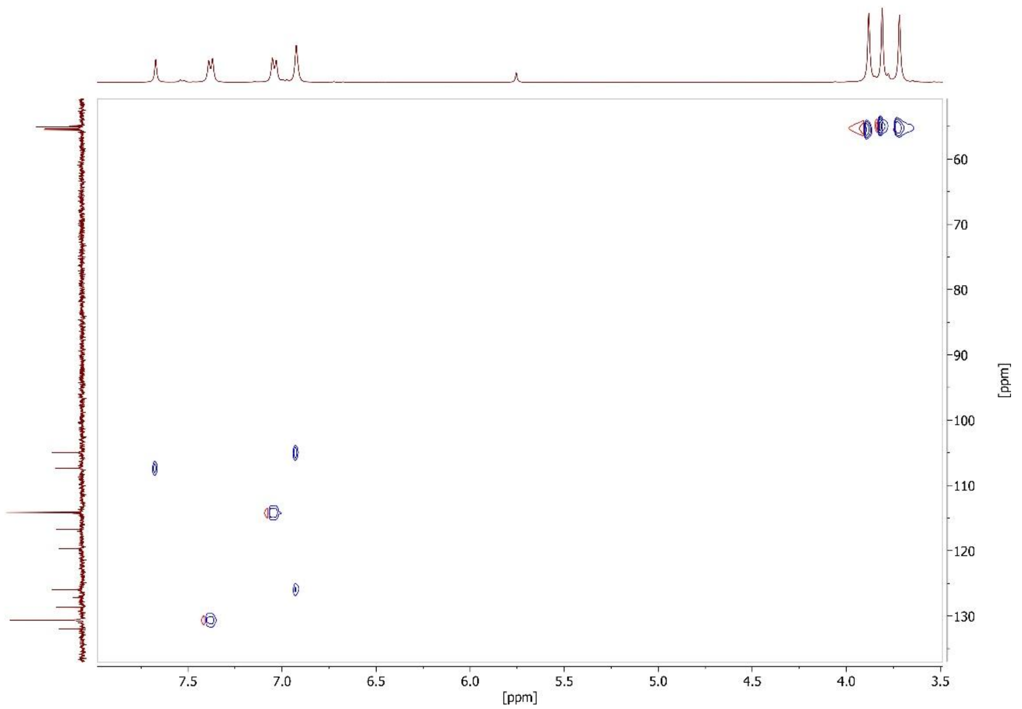

Figure S35. HSQC NMR spectrum of **7c** in DMSO-d_6.

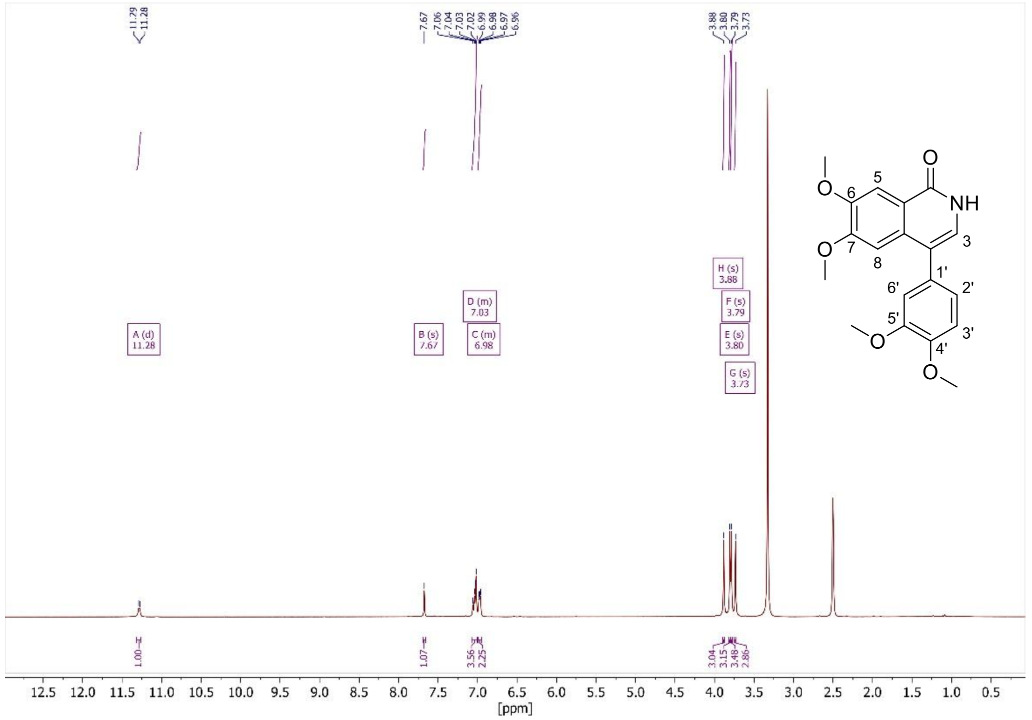

Figure S36. ^{1}H NMR spectrum of **7d** in DMSO-d_6.

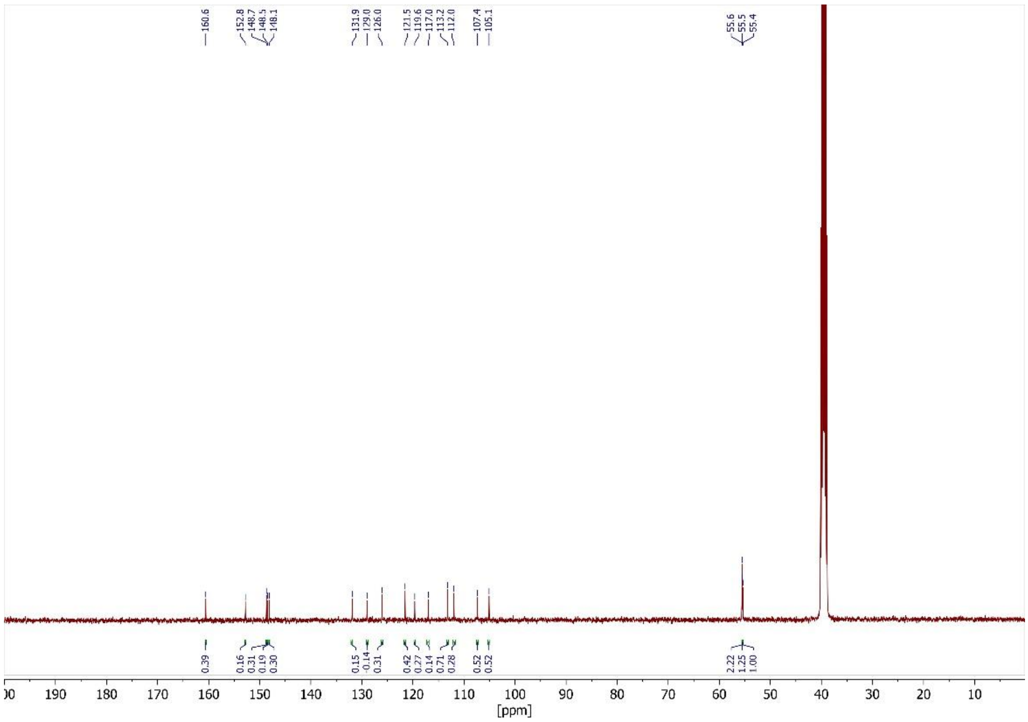

Figure S37. $^{13}C\{^{1}H\}$ NMR spectrum of **7d** in DMSO-*d*$_6$.

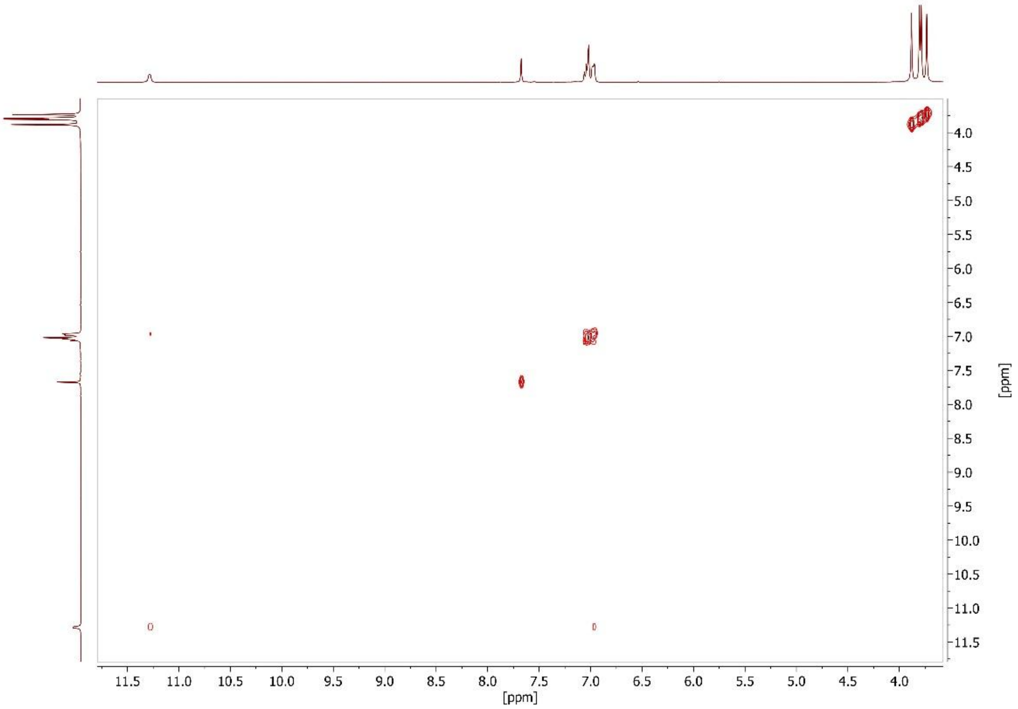

Figure S38. ^{1}H-COSY NMR spectrum of **7d** in DMSO-*d*$_6$.

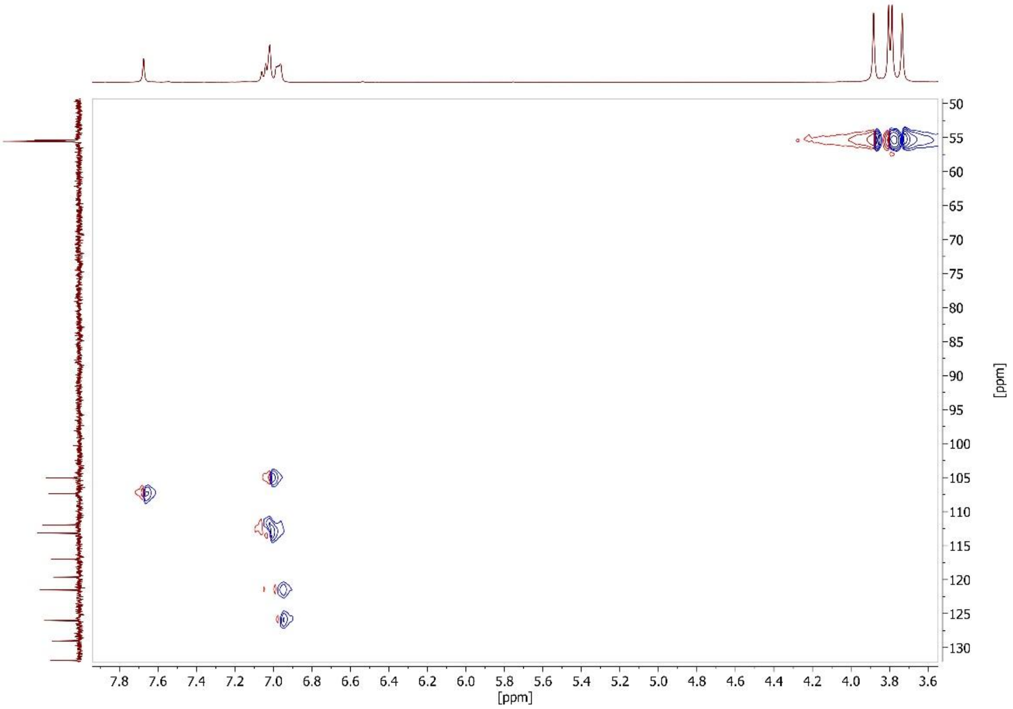

Figure S39. HSQC NMR spectrum of **7d** in DMSO-d_6.

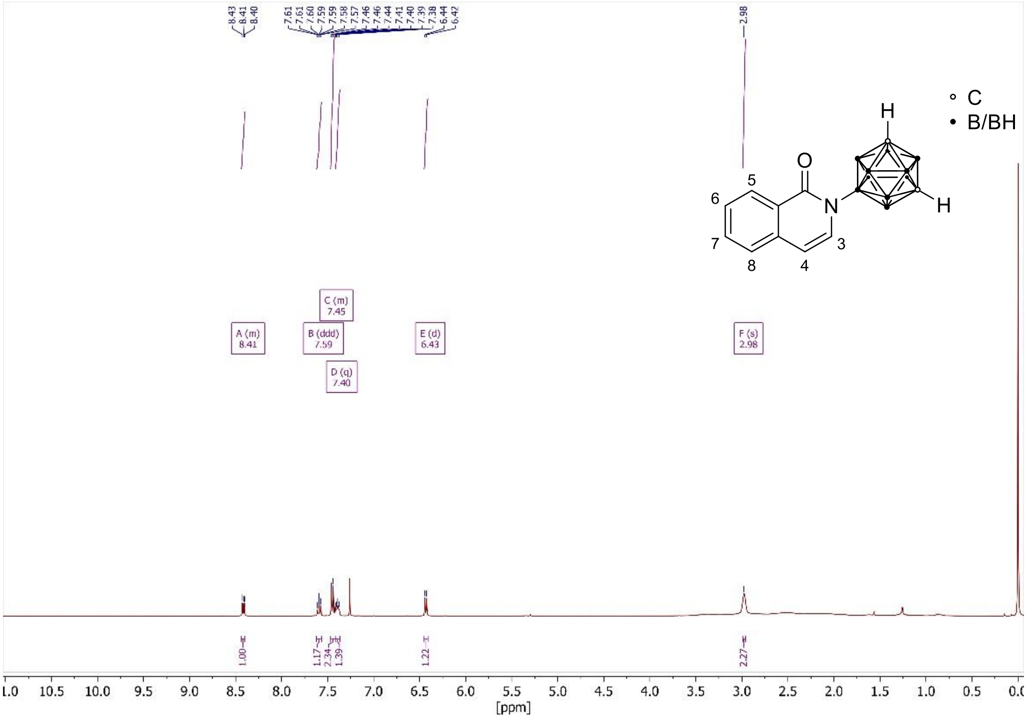

Figure S40. ^{1}H NMR spectrum of **IC-1** in $CDCl_3$.

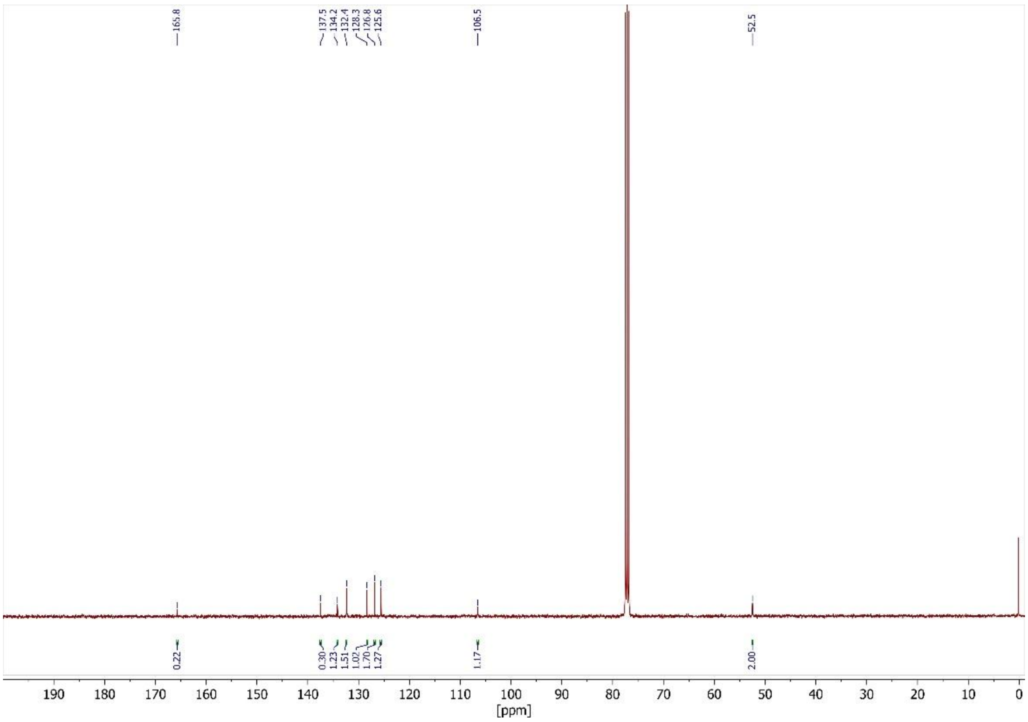

Figure S41. $^{13}C\{^{1}H\}$ NMR spectrum of **IC-1** in $CDCl_3$.

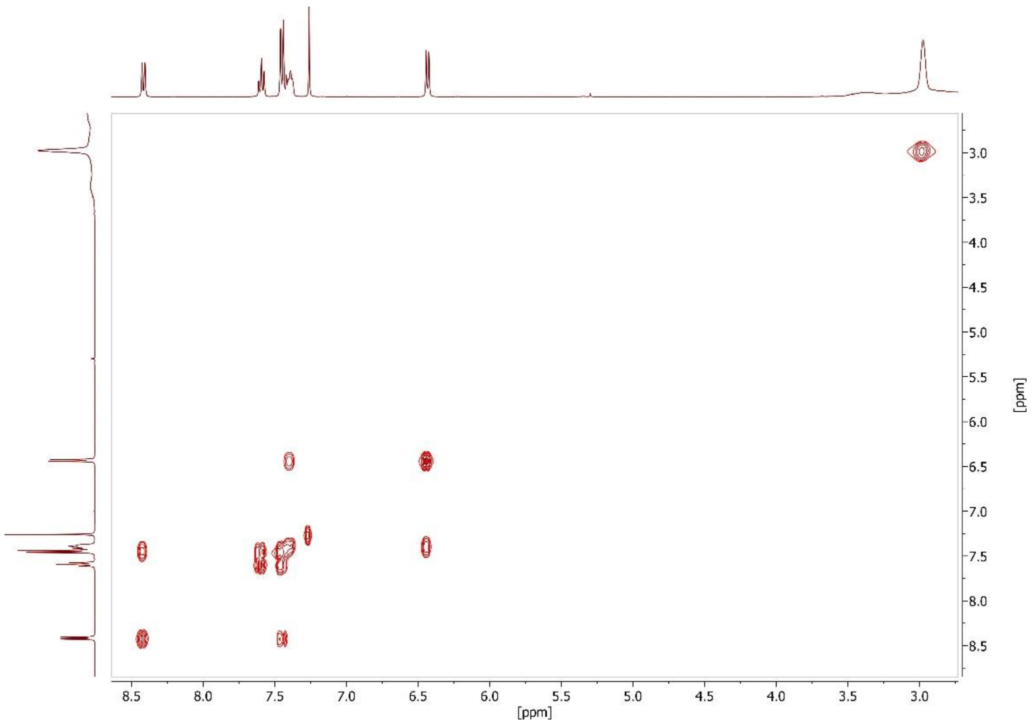

Figure S42. ^{1}H-COSY NMR spectrum of **IC-1** in $CDCl_3$.

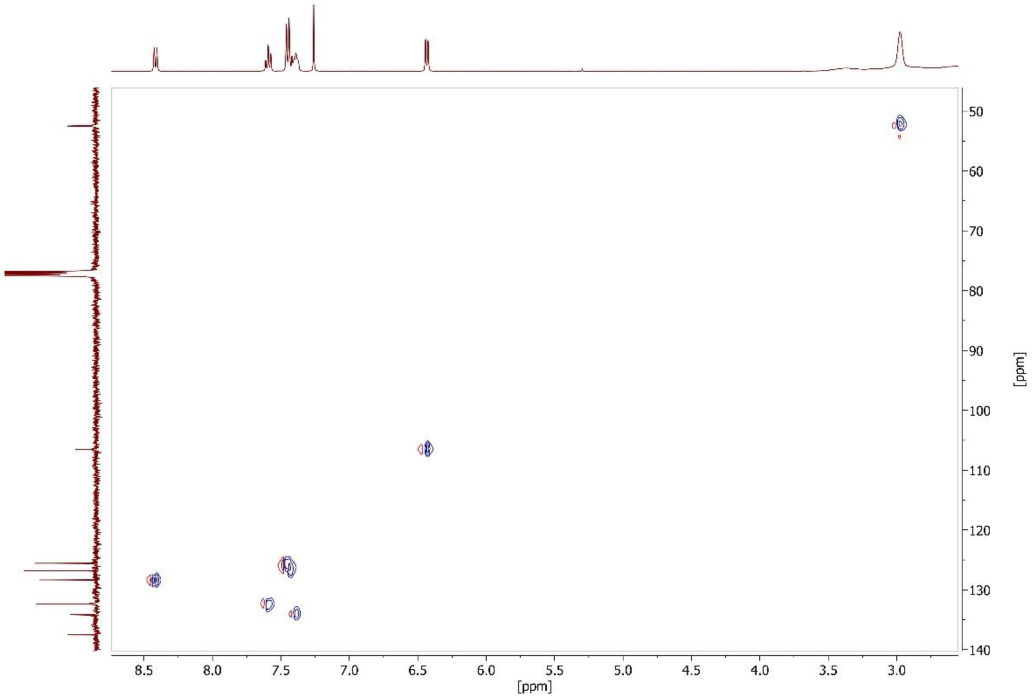

Figure S43. HSQC NMR spectrum of **IC-1** in $CDCl_3$.

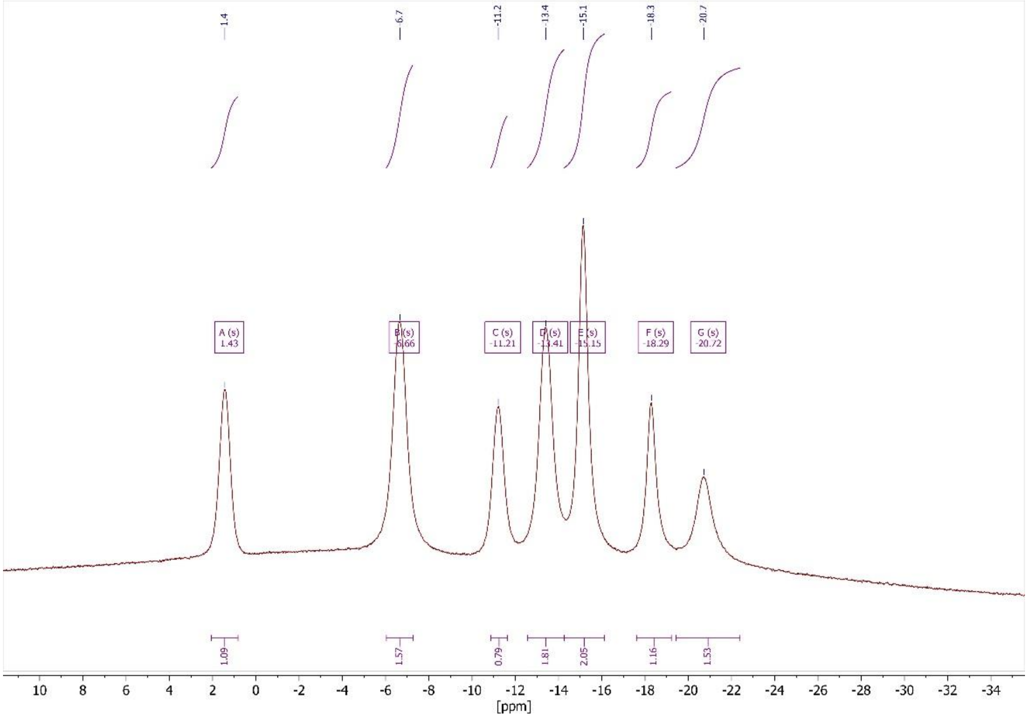

Figure S44. $^{11}B\{^{1}H\}$ NMR spectrum of **IC-1** in $CDCl_3$.

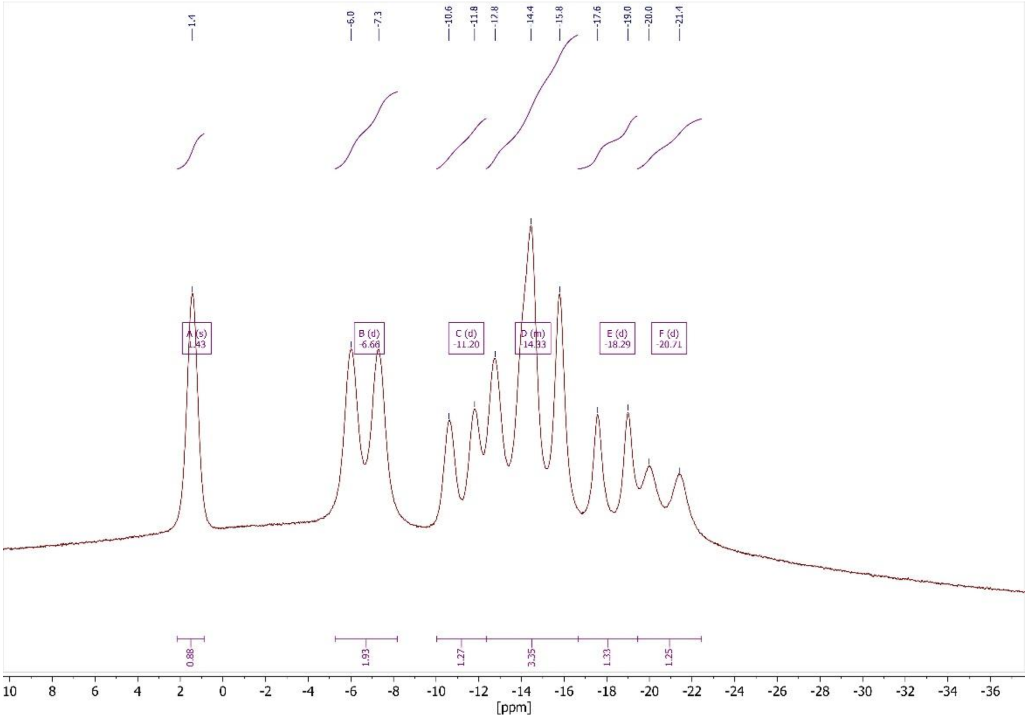

Figure S45. ^{11}B NMR spectrum of **IC-1** in $CDCl_3$.

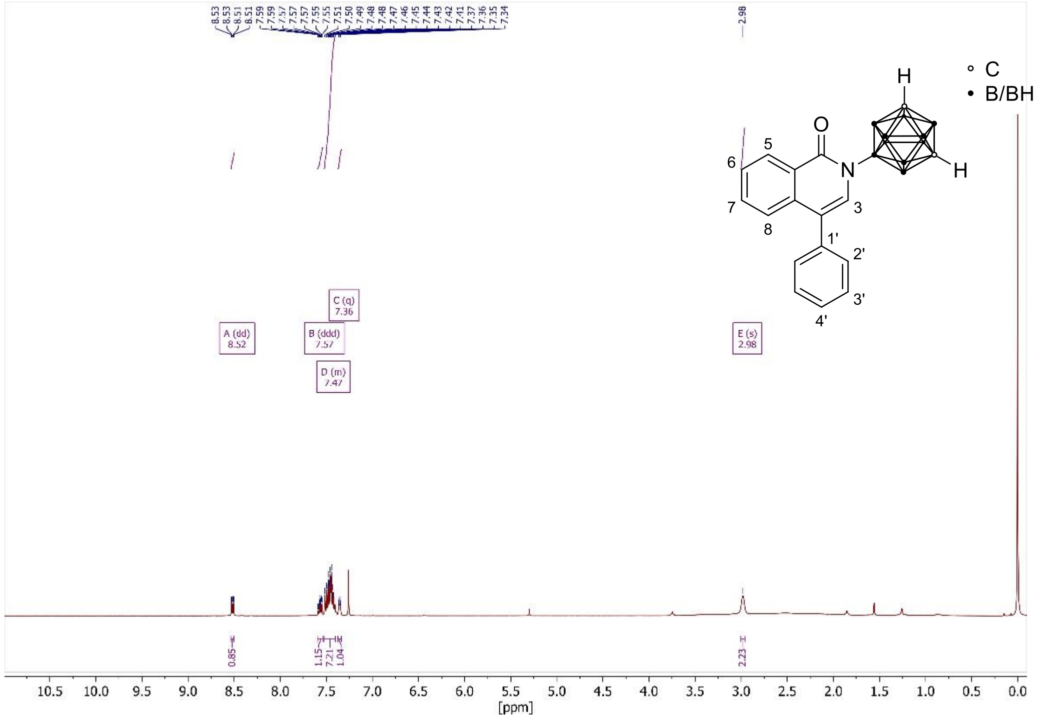

Figure S46. ^{1}H NMR spectrum of **IC-2** in $CDCl_3$.

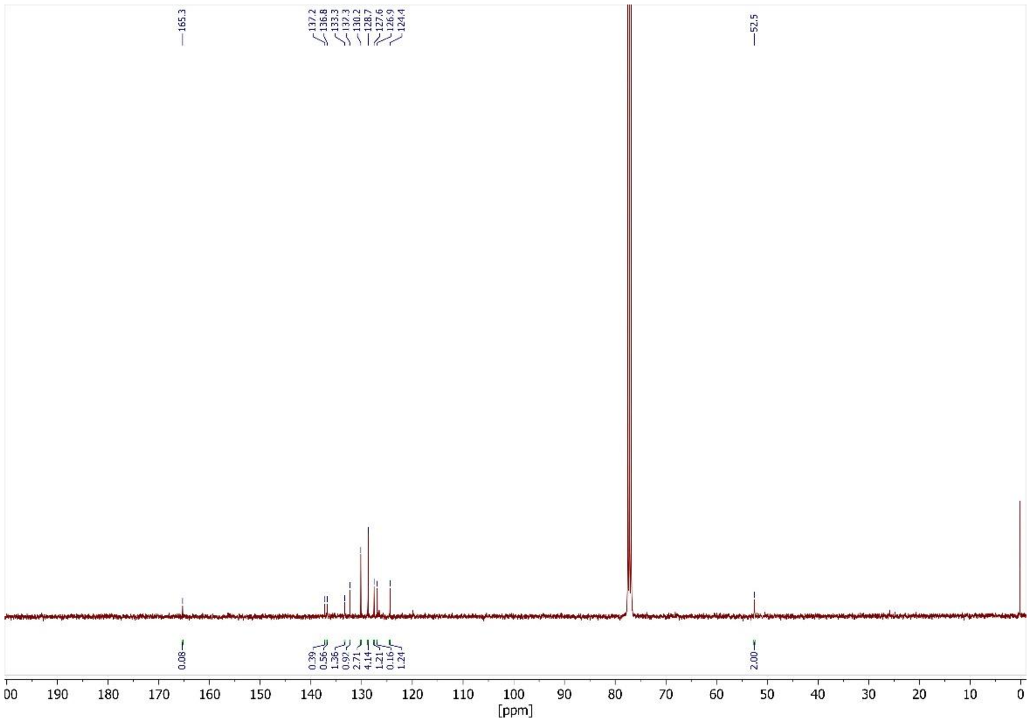

Figure S47. $^{13}C\{^{1}H\}$ NMR spectrum of **IC-2** in $CDCl_3$.

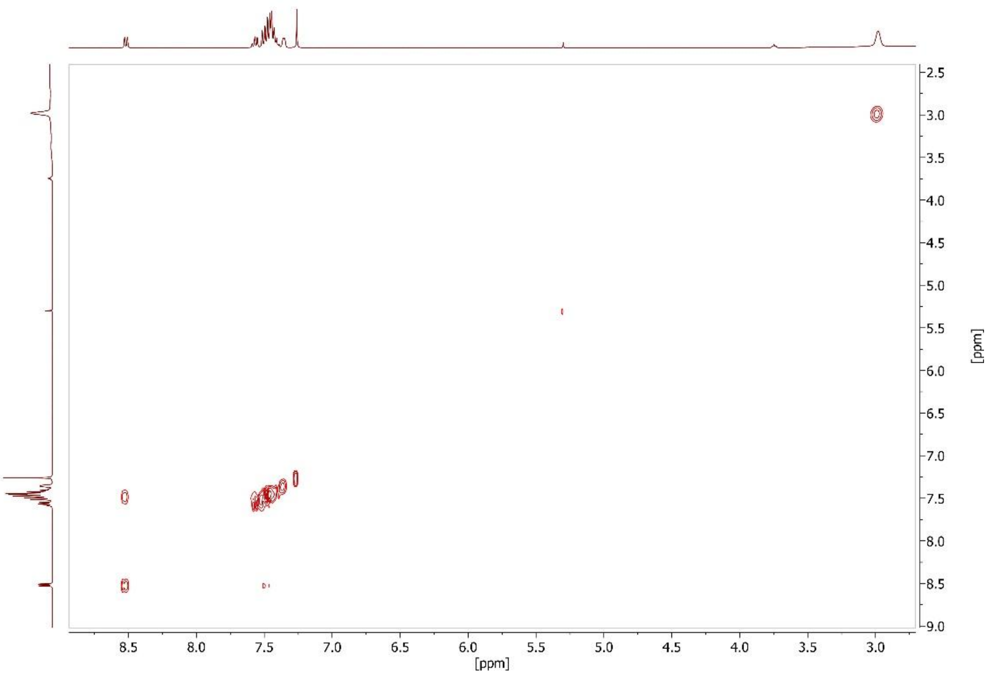

Figure S48. ^{1}H-COSY NMR spectrum of **IC-2** in $CDCl_3$.

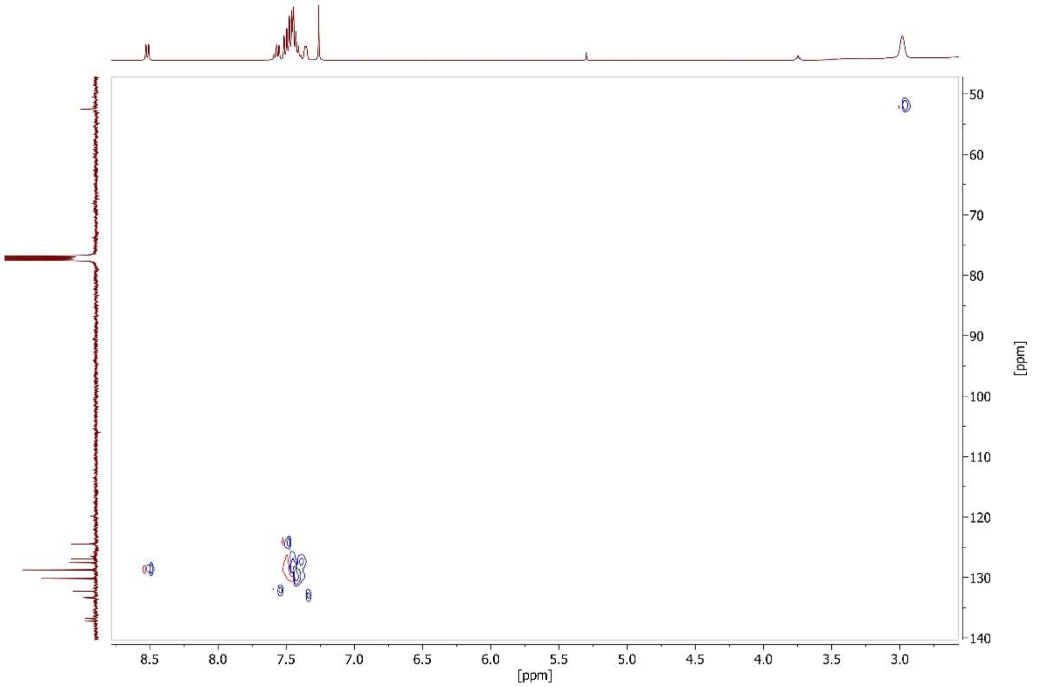

Figure S49. HSQC NMR spectrum of **IC-2** in $CDCl_3$.

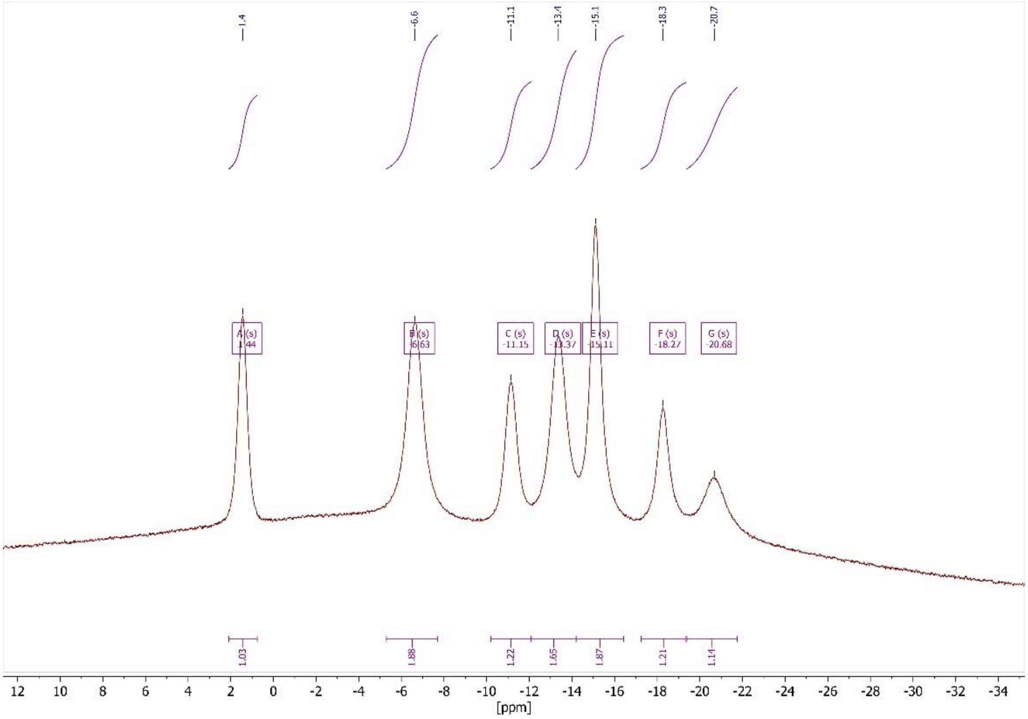

Figure S50. $^{11}B\{^{1}H\}$ NMR spectrum of **IC-2** in $CDCl_3$.

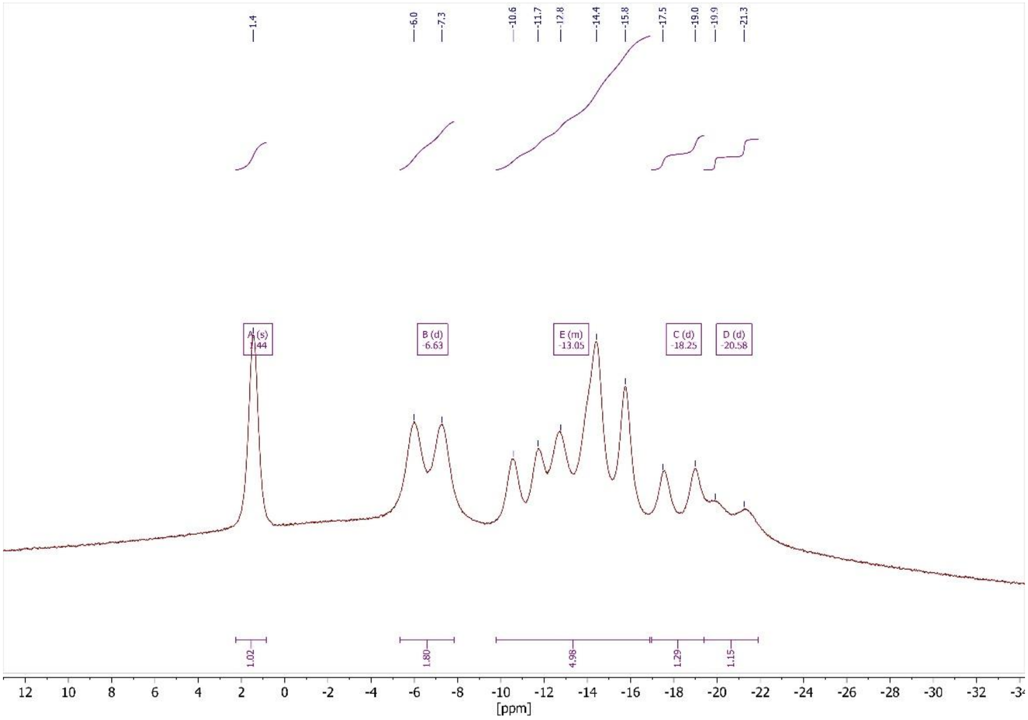

Figure S51. ^{11}B NMR spectrum of **IC-2** in $CDCl_3$.

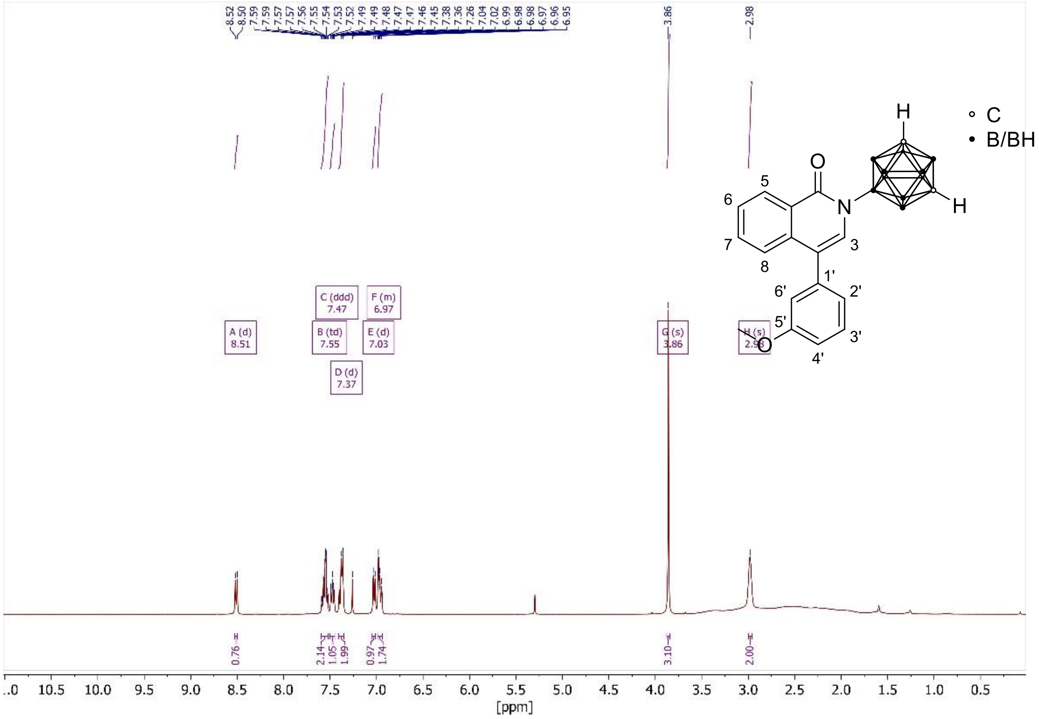

Figure S52. ^{1}H NMR spectrum of **IC-3** in $CDCl_3$.

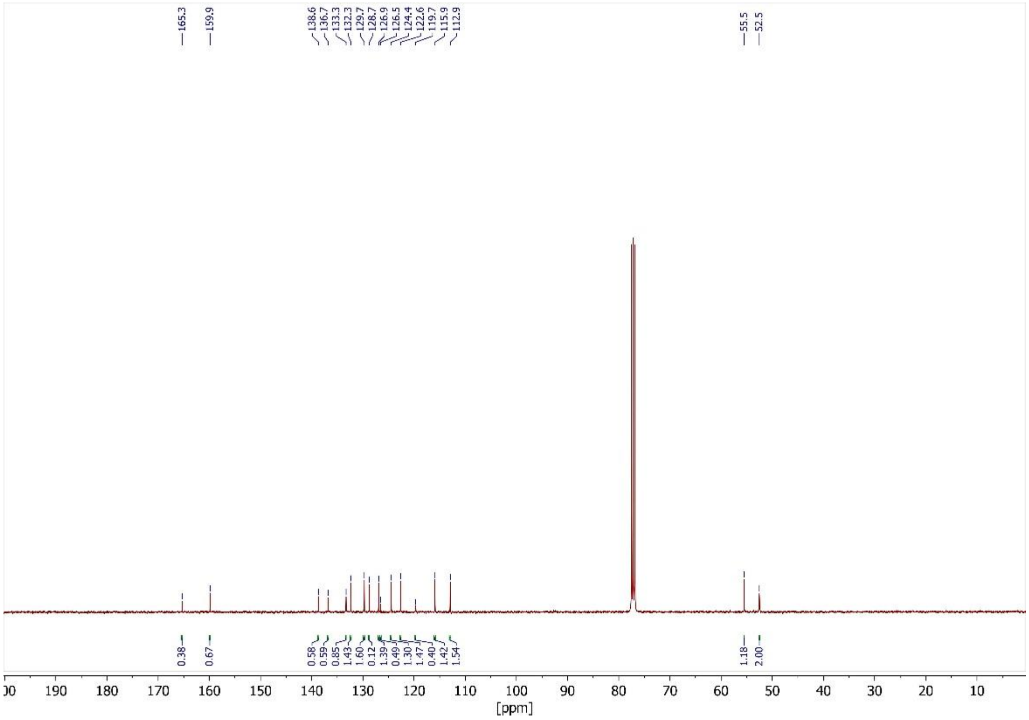

Figure S53. $^{13}C\{^{1}H\}$ NMR spectrum of **IC-3** in $CDCl_3$.

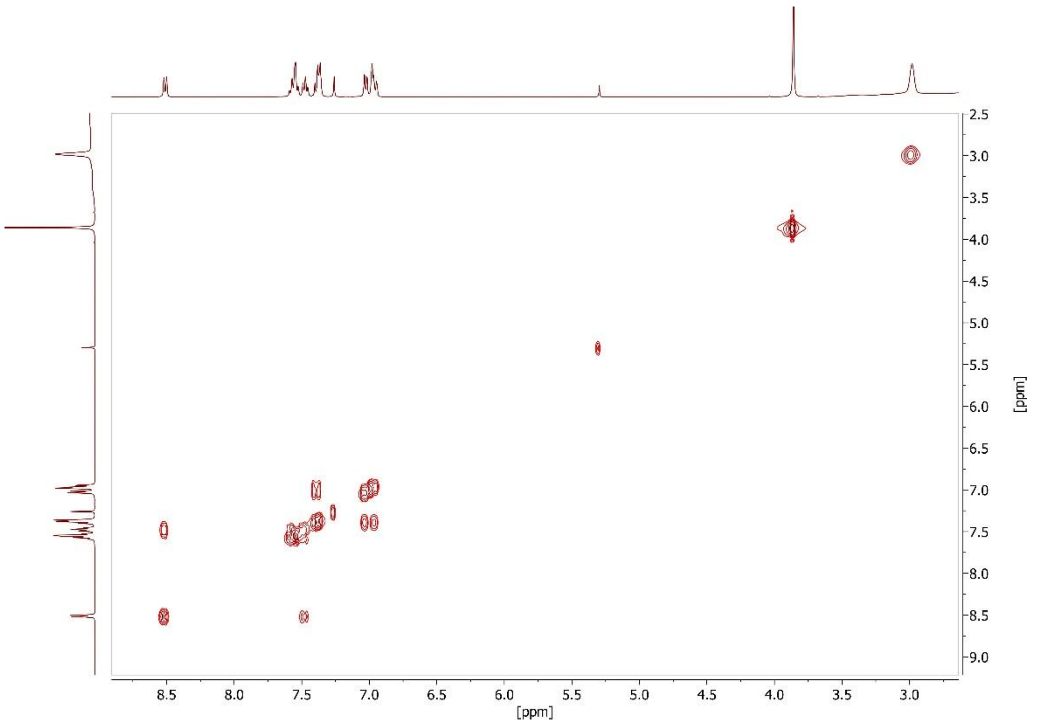

Figure S54. ^{1}H-COSY NMR spectrum of **IC-3** in $CDCl_3$.

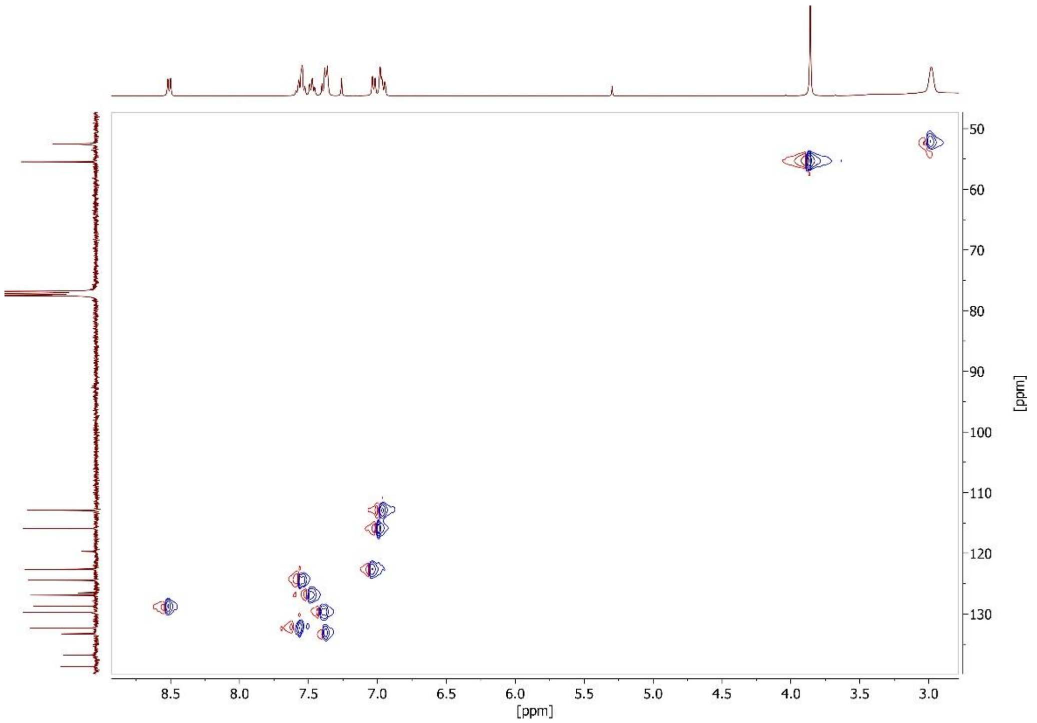

Figure S55. HSQC NMR spectrum of **IC-3** in $CDCl_3$.

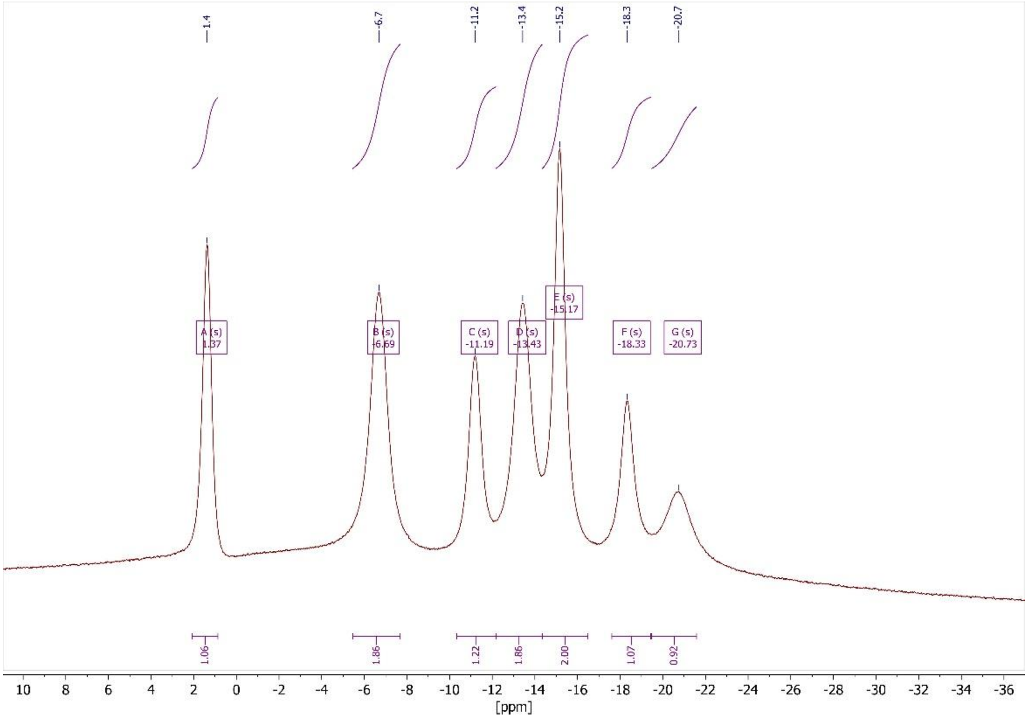

Figure S56. $^{11}B\{^{1}H\}$ NMR spectrum of **IC-3** in $CDCl_3$.

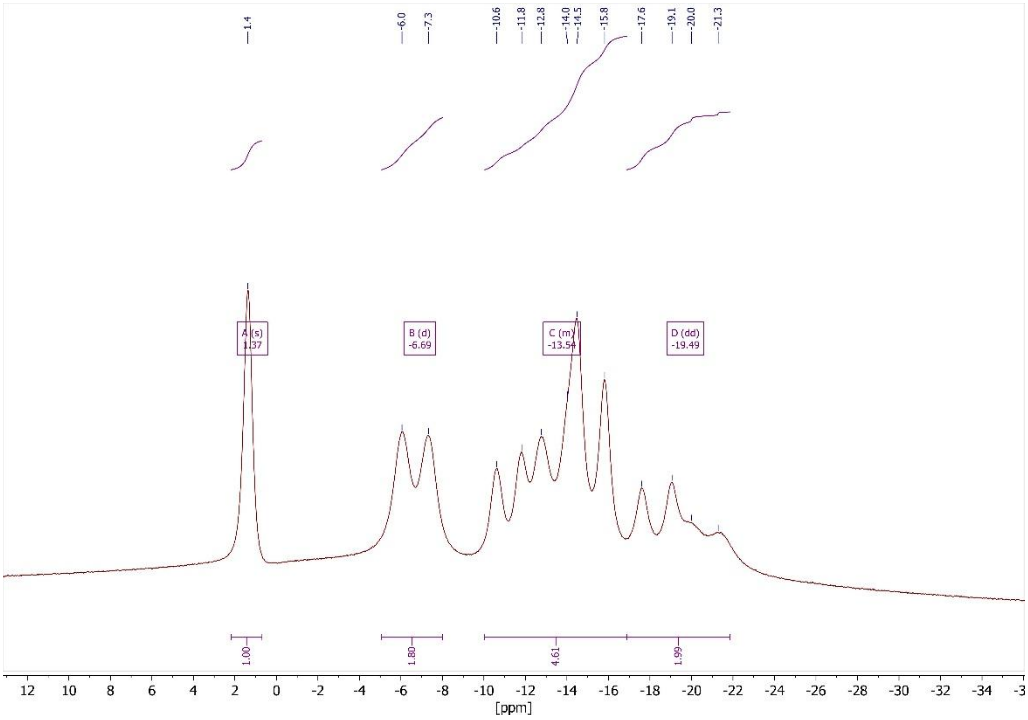

Figure S57. ^{11}B NMR spectrum of **IC-3** in $CDCl_3$.

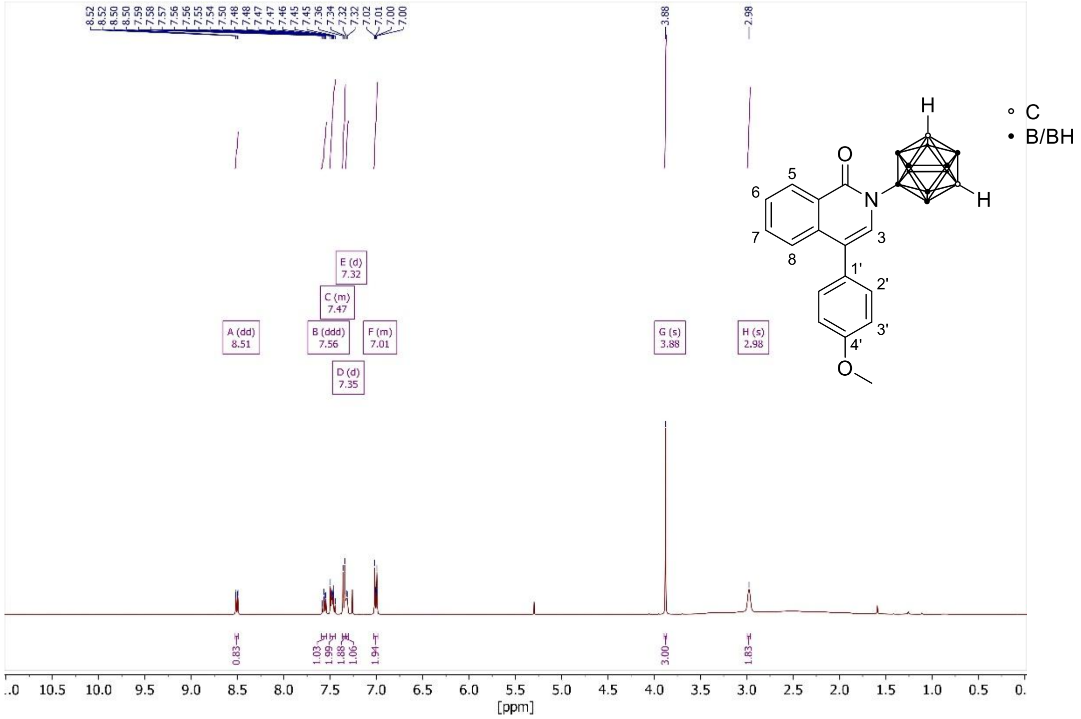

Figure S58. ^{1}H NMR spectrum of **IC-4** in $CDCl_3$.

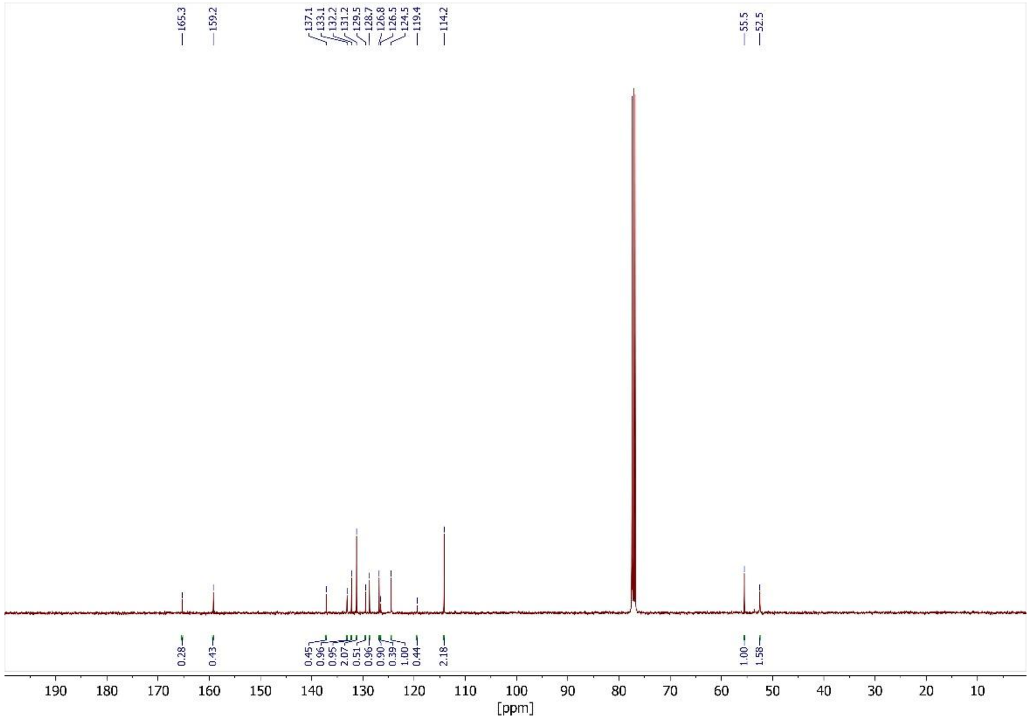

Figure S59. $^{13}C\{^{1}H\}$ NMR spectrum of **IC-4** in $CDCl_3$.

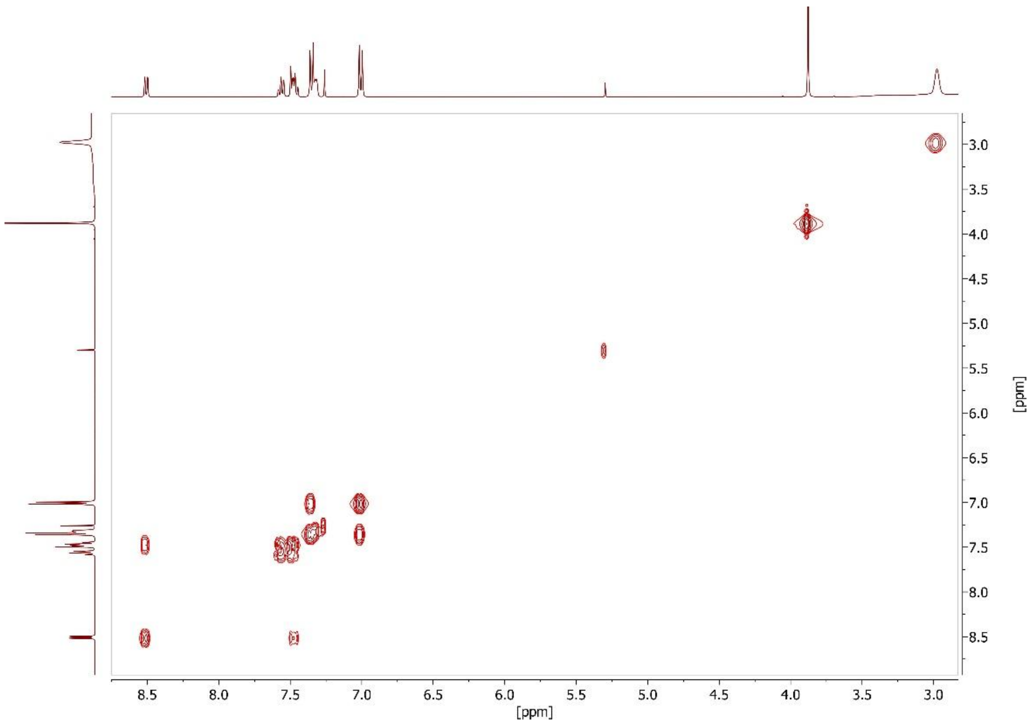

Figure S60. ^{1}H-COSY NMR spectrum of **IC-4** in $CDCl_3$.

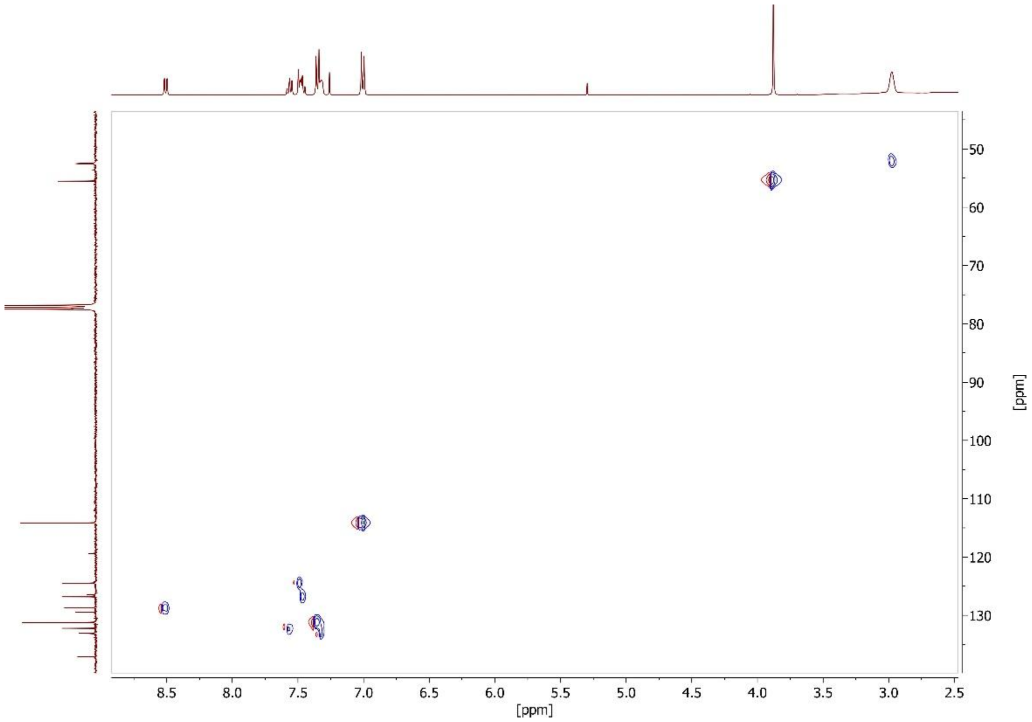

Figure S61. HSQC NMR spectrum of **IC-4** in $CDCl_3$.

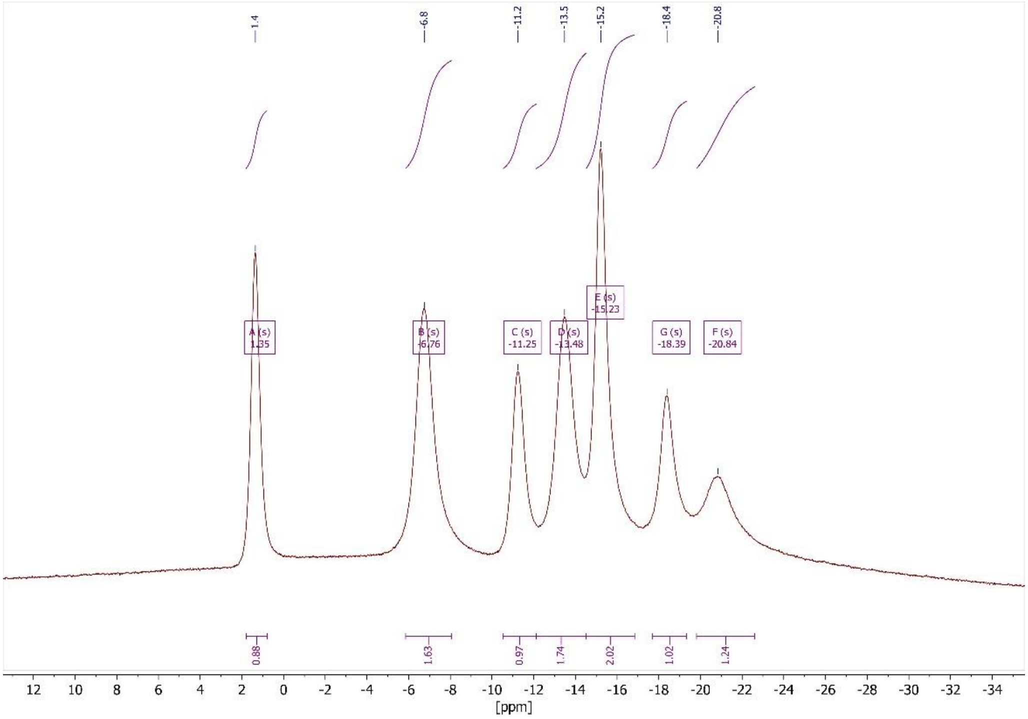

Figure S62. $^{11}B\{^{1}H\}$ NMR spectrum of **IC-4** in $CDCl_3$.

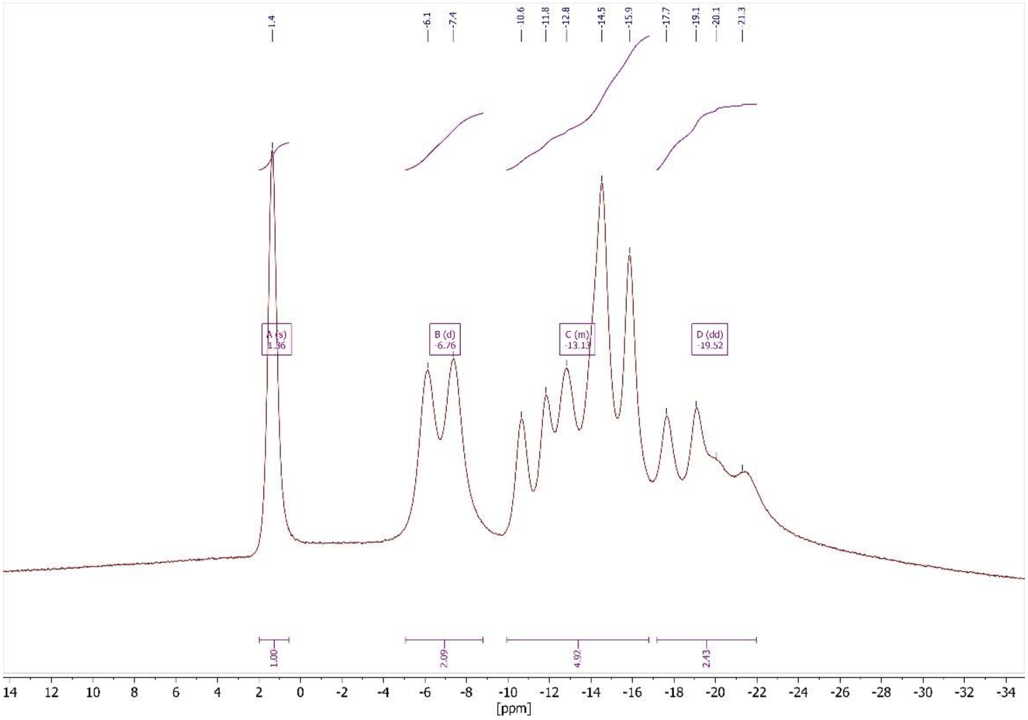

Figure S63. ^{11}B NMR spectrum of **IC-4** in $CDCl_3$.

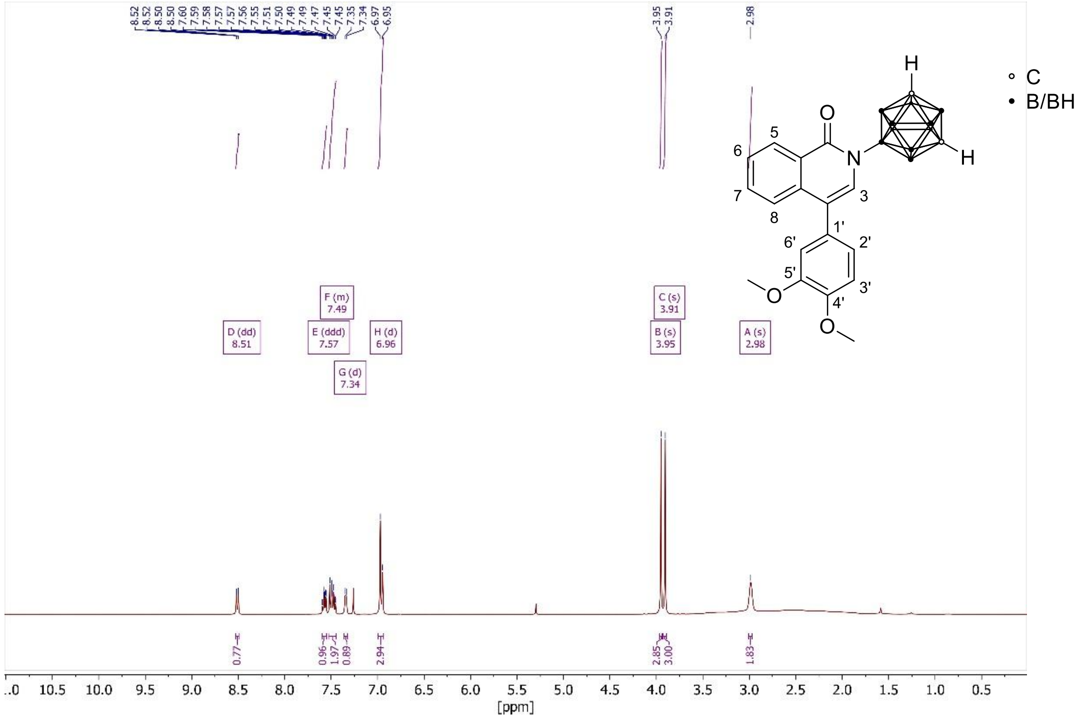

Figure S64. ^{1}H NMR spectrum of **IC-5** in $CDCl_3$.

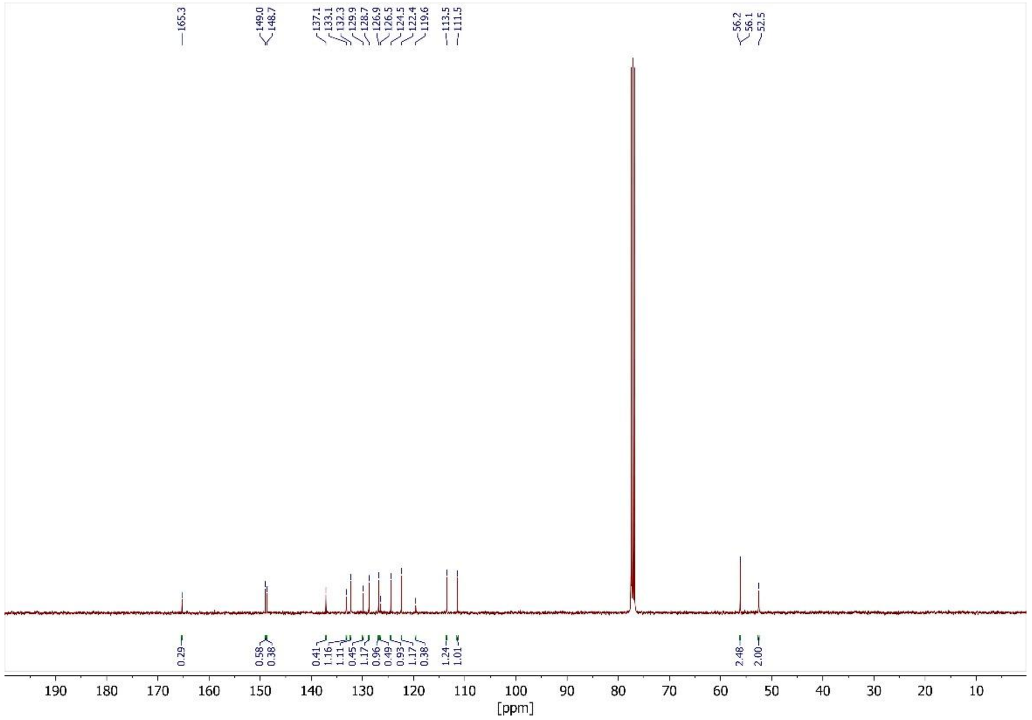

Figure S65. $^{13}C\{^{1}H\}$ NMR spectrum of **IC-5** in $CDCl_3$.

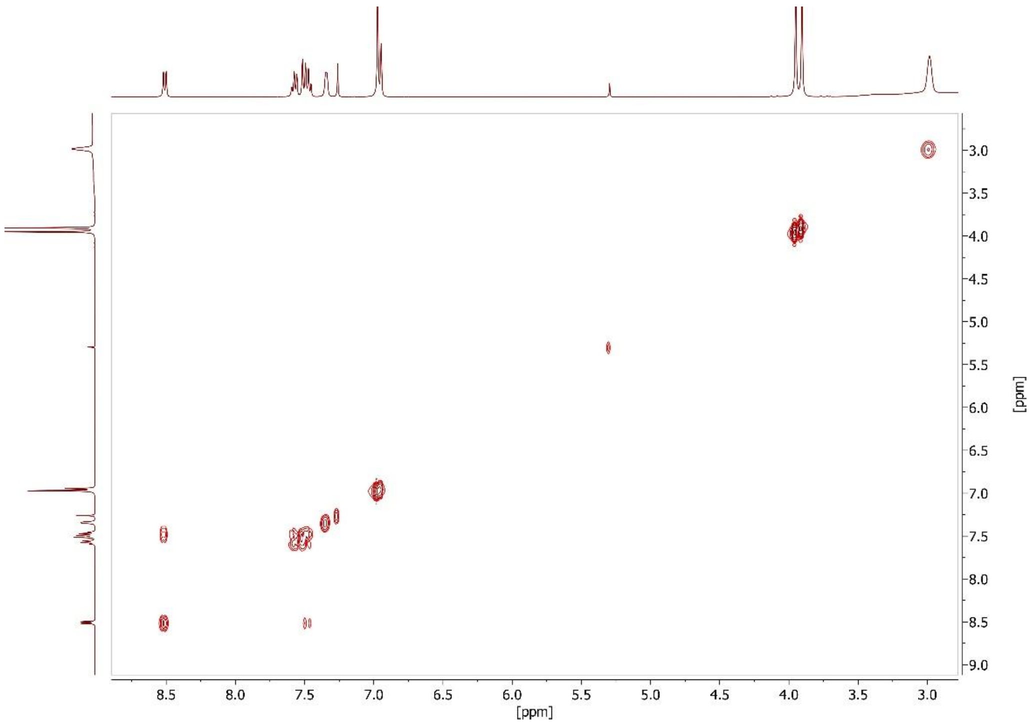

Figure S66. ^{1}H-COSY NMR spectrum of **IC-5** in $CDCl_3$.

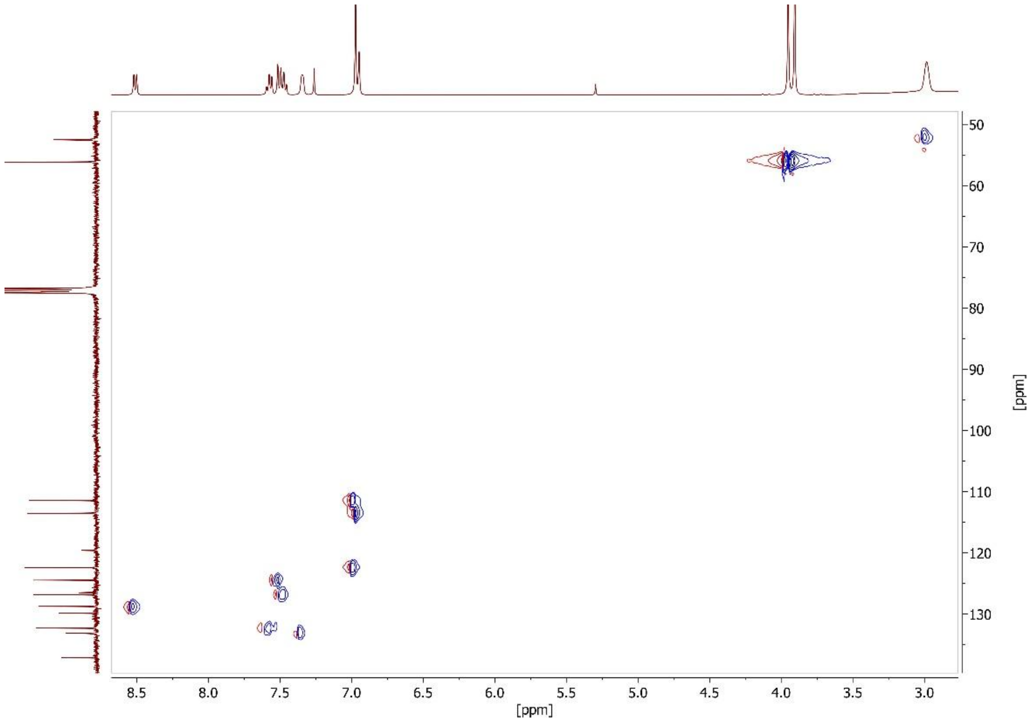

Figure S67. HSQC NMR spectrum of **IC-5** in $CDCl_3$.

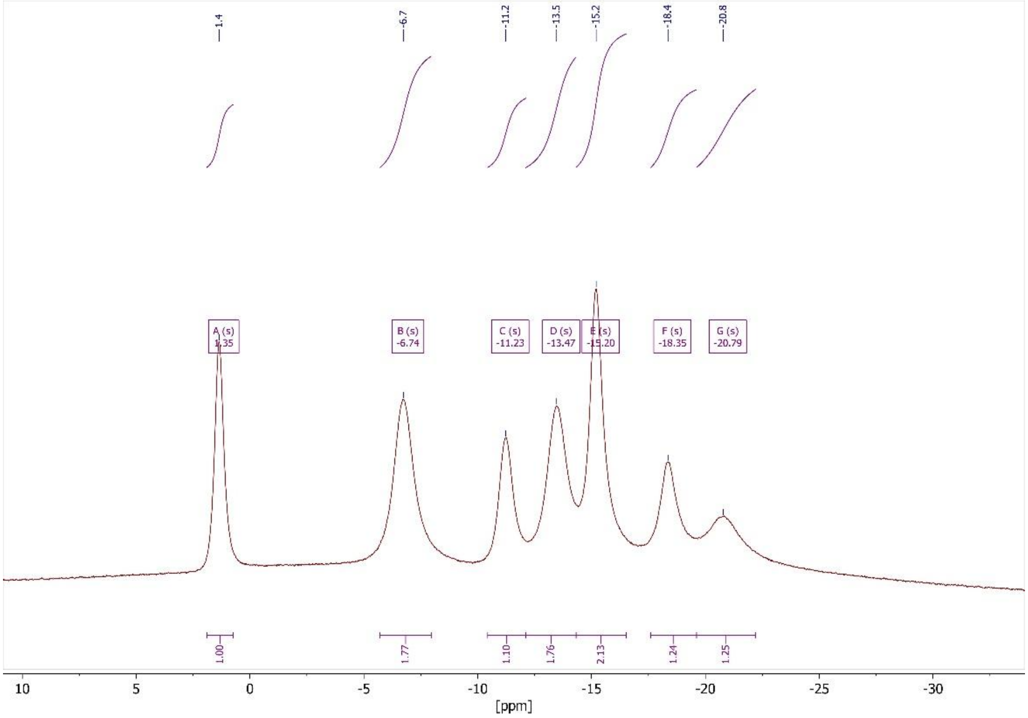

Figure S68. $^{11}B\{^{1}H\}$ NMR spectrum of **IC-5** in $CDCl_3$.

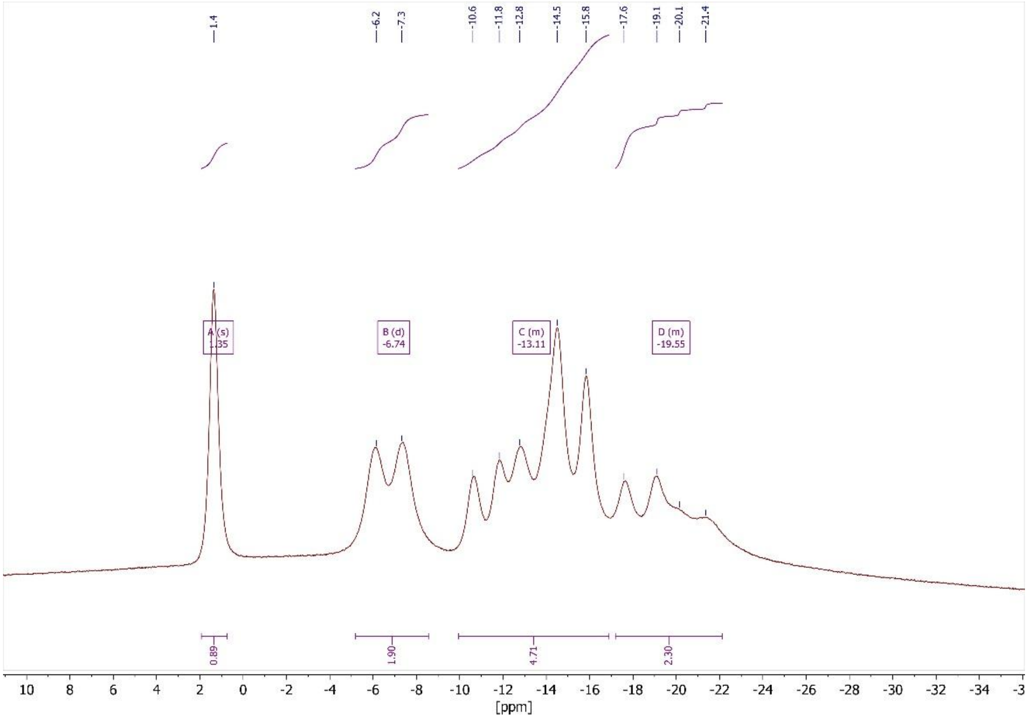

Figure S69. ^{11}B NMR spectrum of **IC-5** in $CDCl_3$.

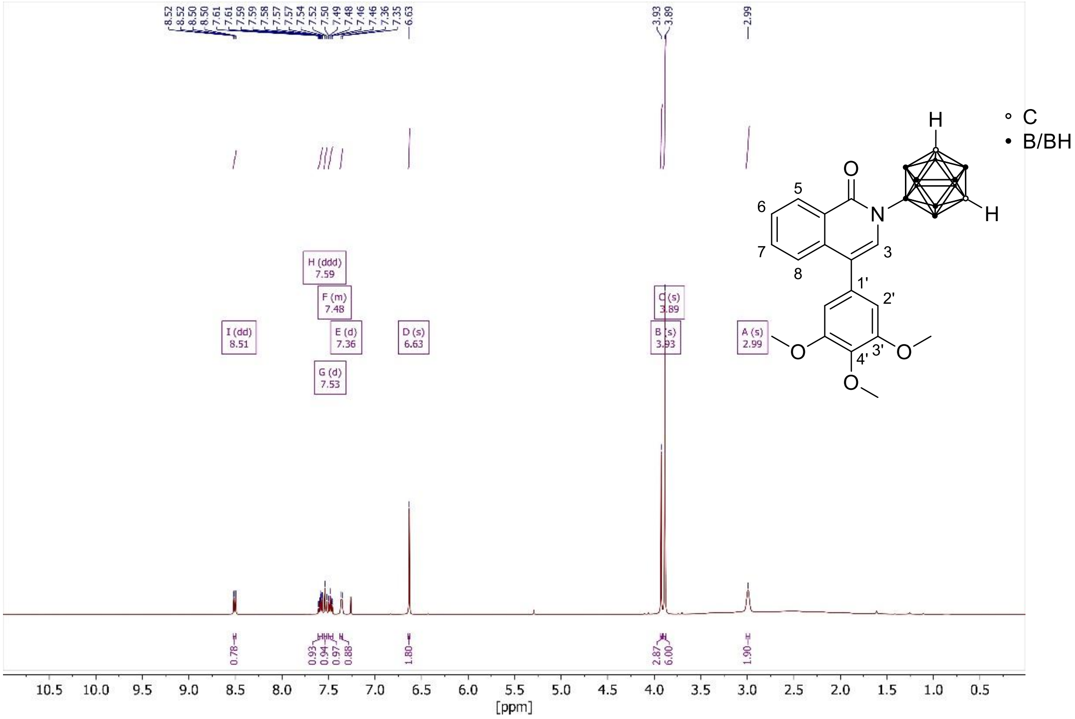

Figure S70. ^{1}H NMR spectrum of **IC-6** in $CDCl_3$.

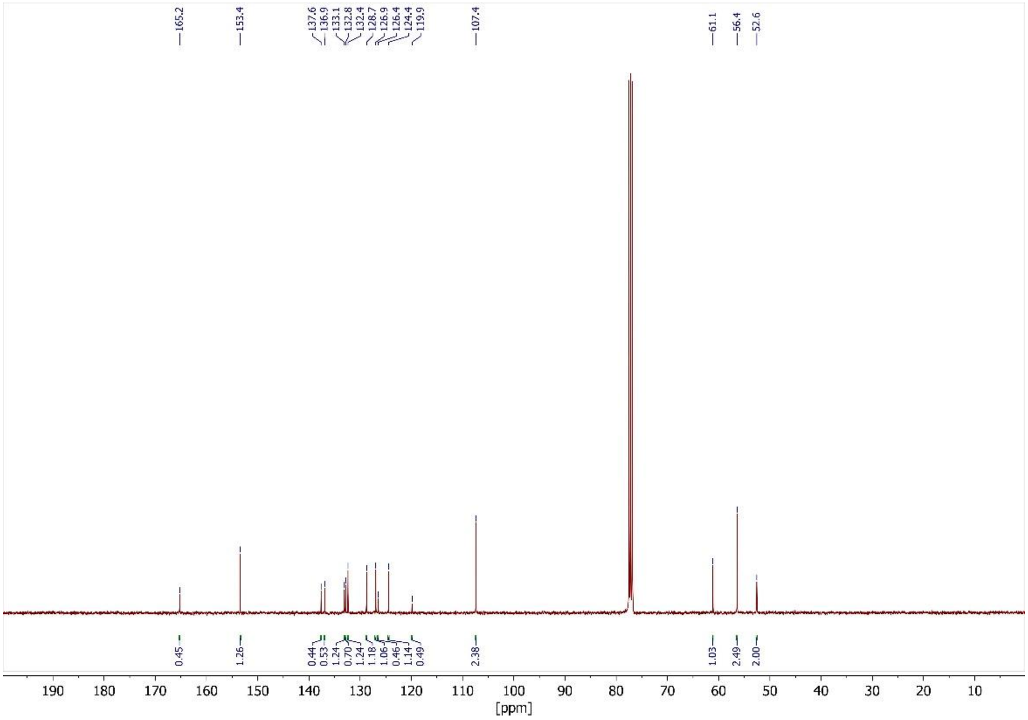

Figure S71. $^{13}C\{^{1}H\}$ NMR spectrum of **IC-6** in $CDCl_3$.

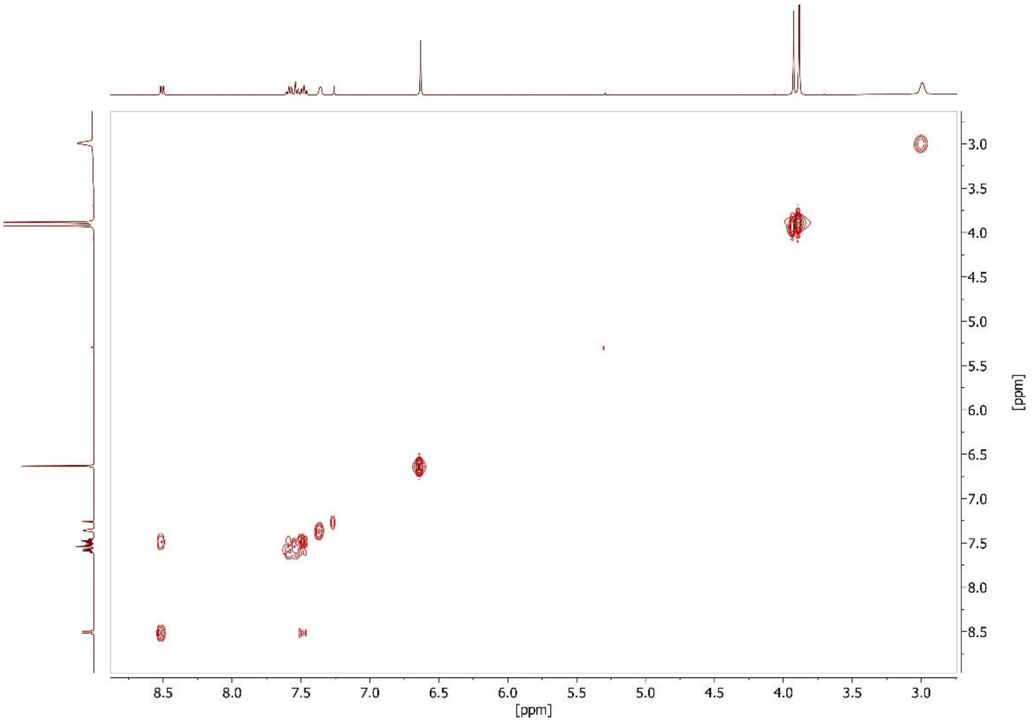

Figure S72. ^{1}H-COSY NMR spectrum of **IC-6** in $CDCl_3$.

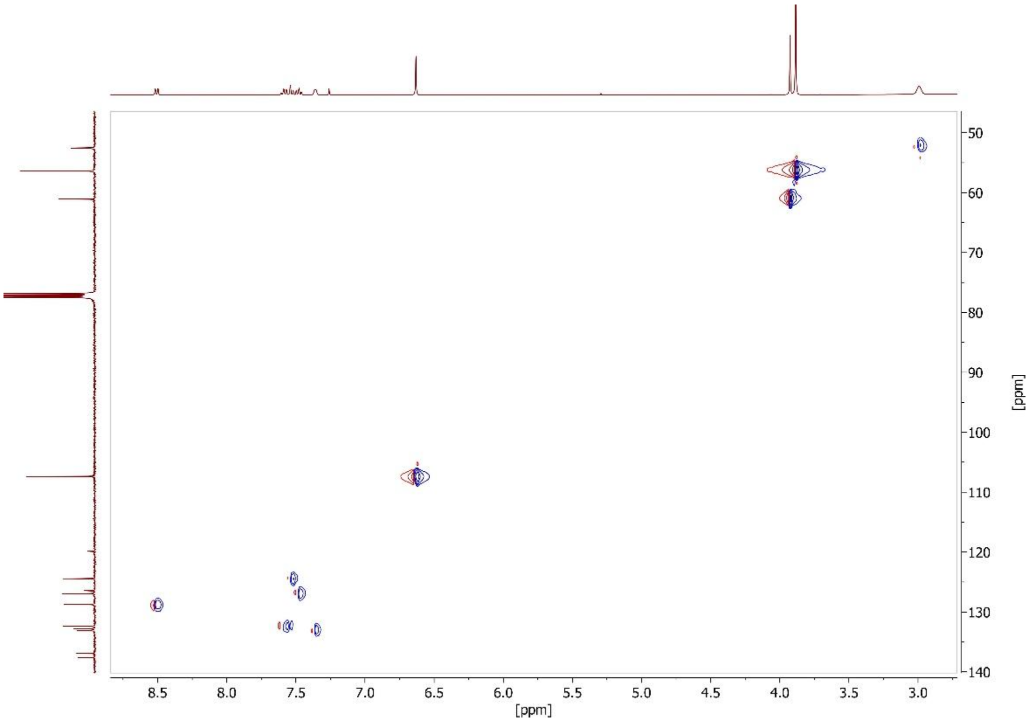

Figure S73. HSQC NMR spectrum of **IC-6** in $CDCl_3$.

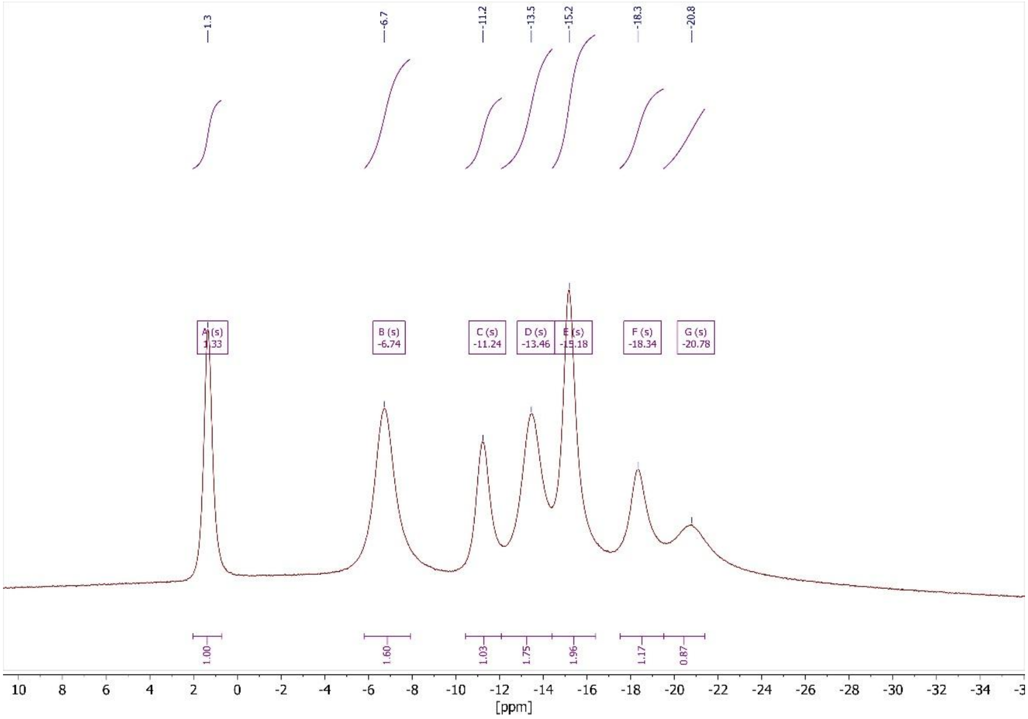

Figure S74. $^{11}B\{^{1}H\}$ NMR spectrum of **IC-6** in $CDCl_3$.

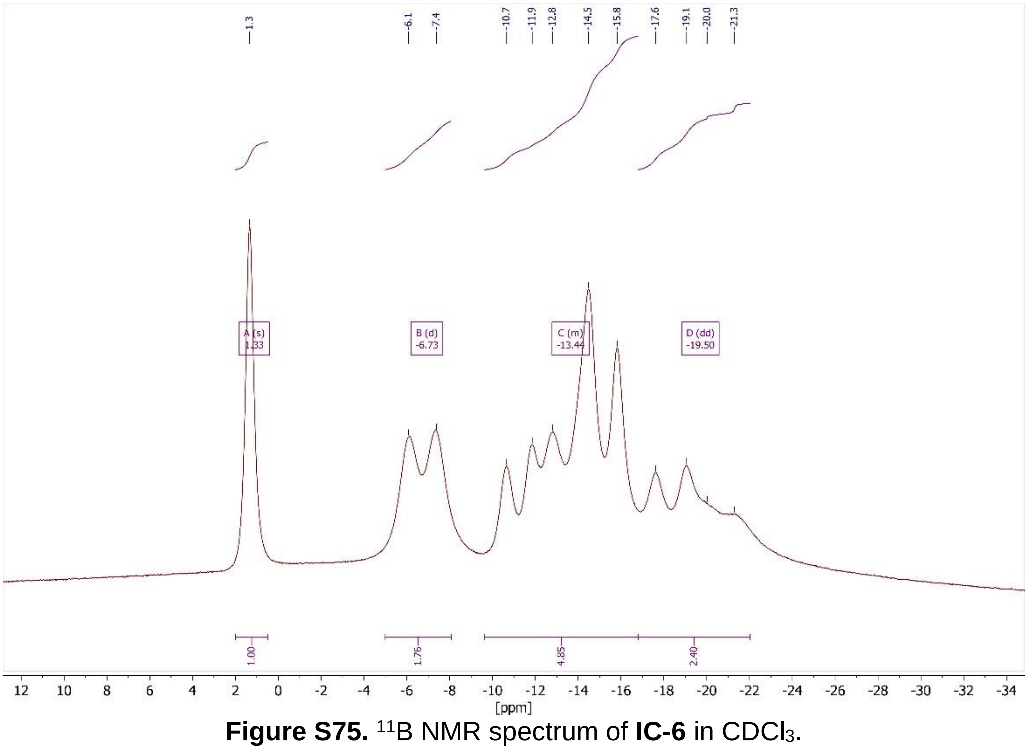

Figure S75. ^{11}B NMR spectrum of **IC-6** in $CDCl_3$.

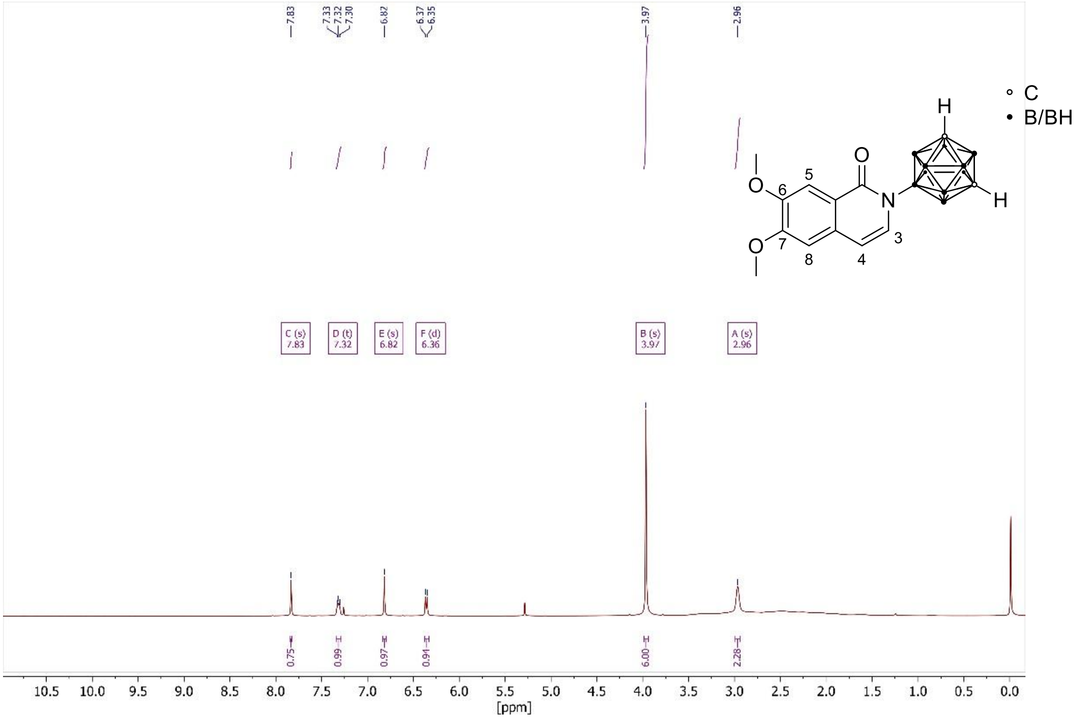

Figure S76. ^{1}H NMR spectrum of **IC-7** in $CDCl_3$.

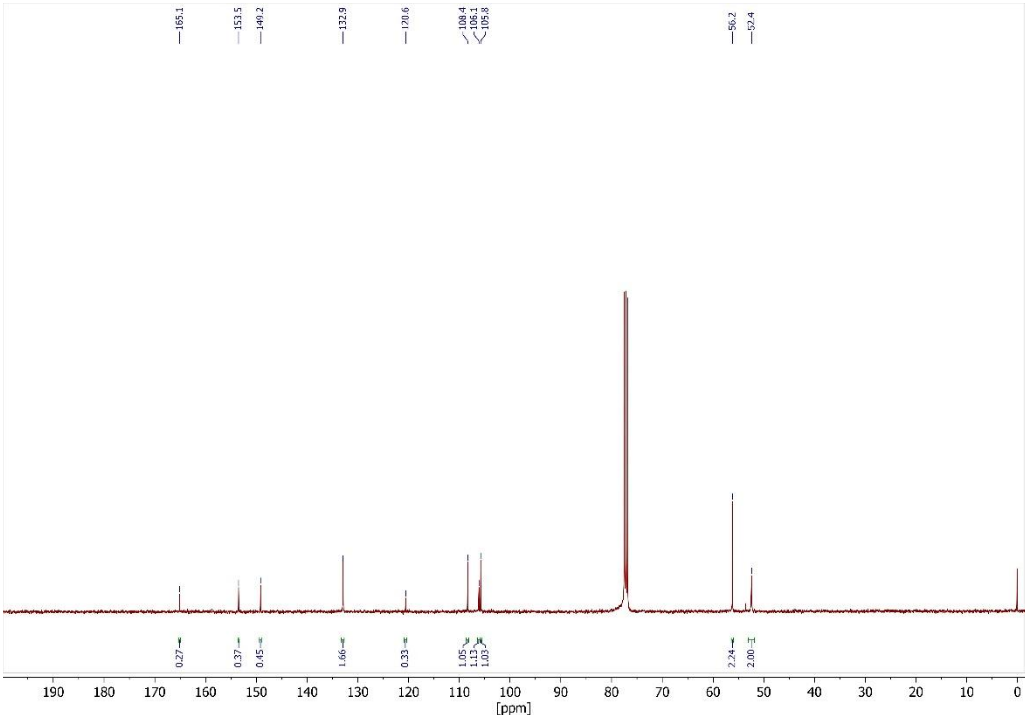

Figure S77. $^{13}C\{^{1}H\}$ NMR spectrum of **IC-7** in $CDCl_3$.

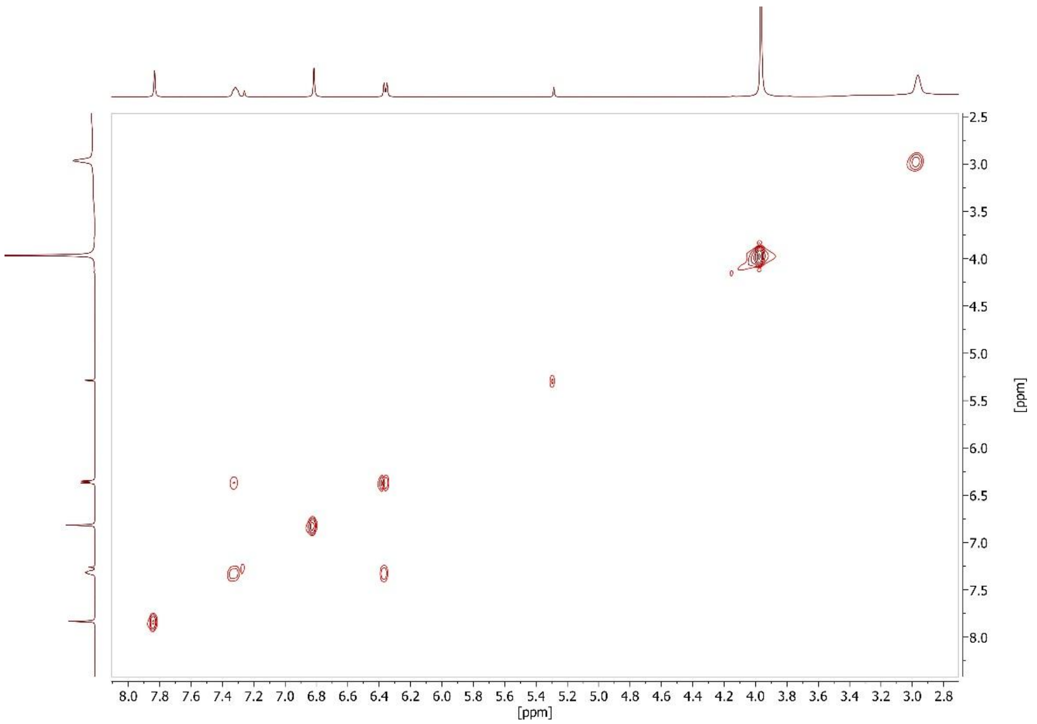

Figure S78. ^{1}H-COSY NMR spectrum of **IC-7** in $CDCl_3$.

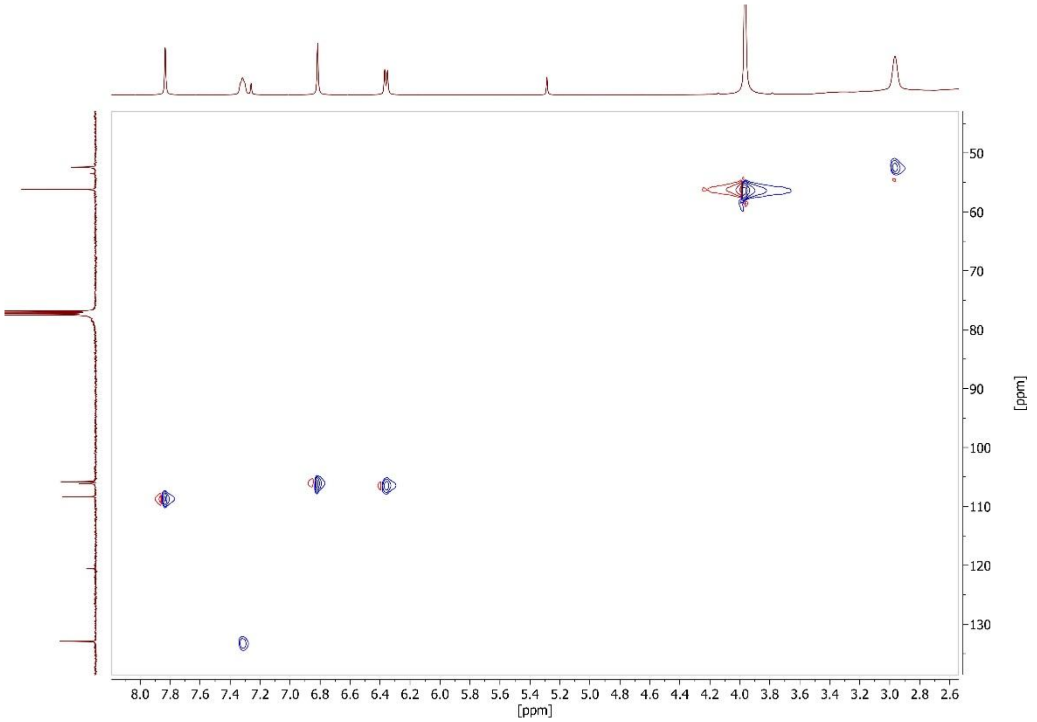

Figure S79. HSQC NMR spectrum of **IC-7** in $CDCl_3$.

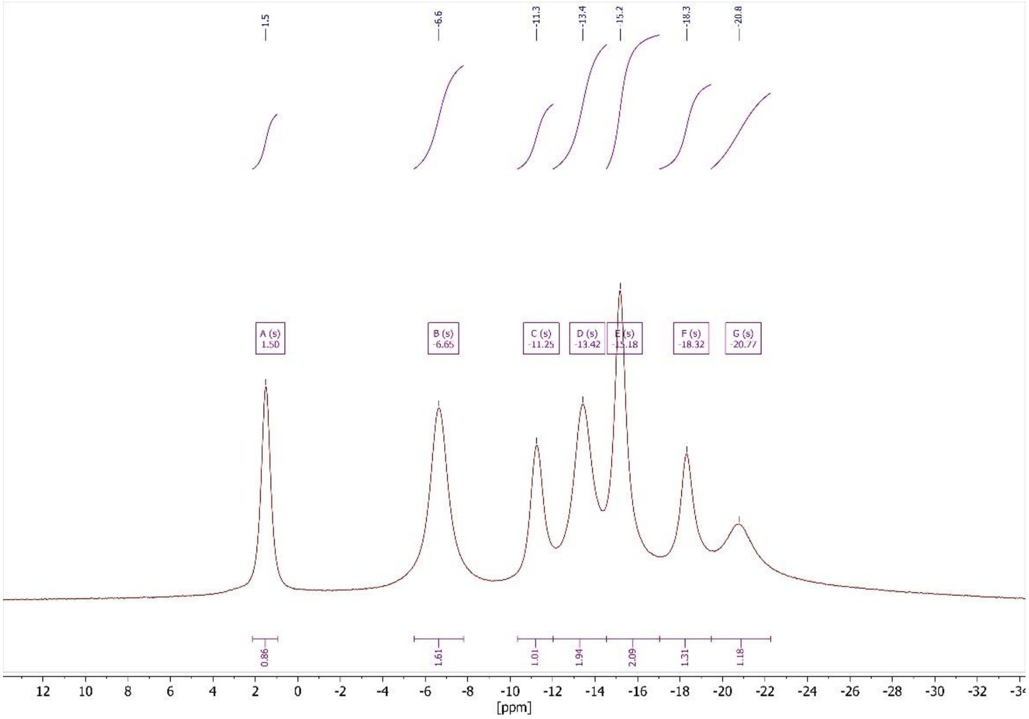

Figure S80. $^{11}B\{^{1}H\}$ NMR spectrum of **IC-7** in $CDCl_3$.

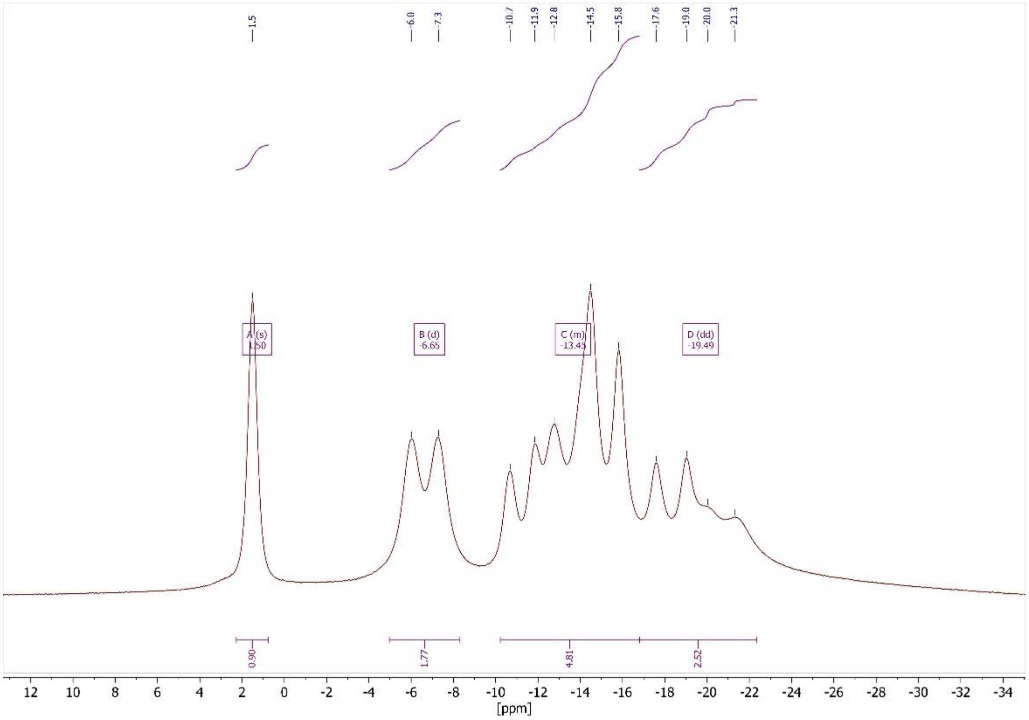

Figure S81. ^{11}B NMR spectrum of **IC-7** in $CDCl_3$.

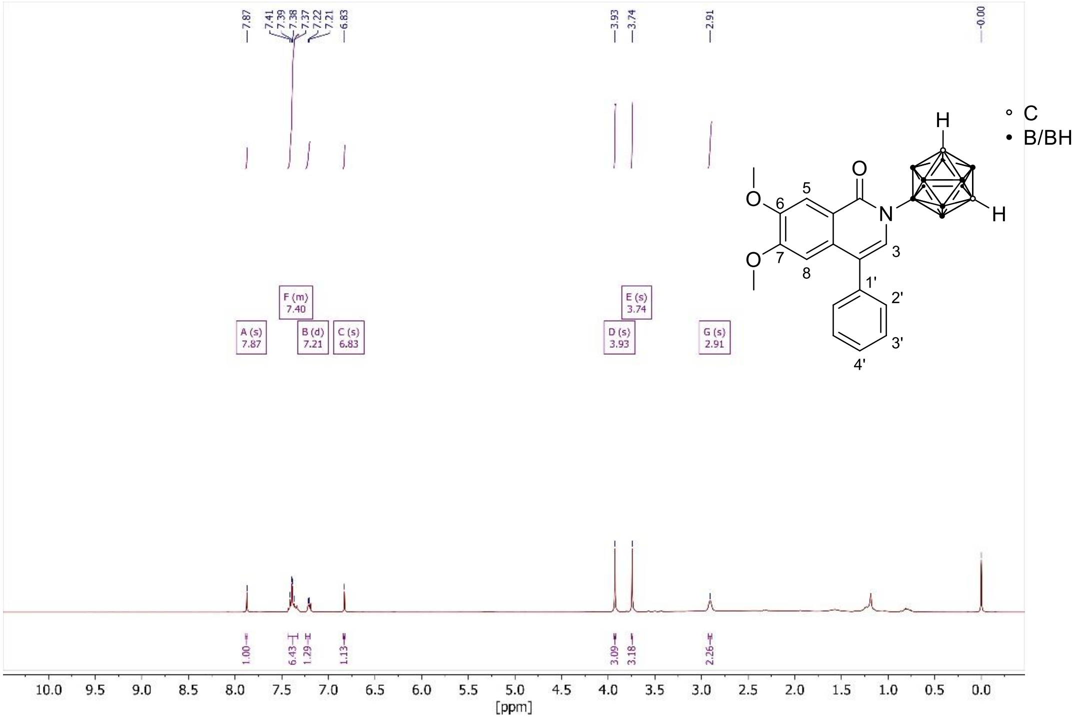

Figure S82. ^{1}H NMR spectrum of **IC-8** in $CDCl_3$.

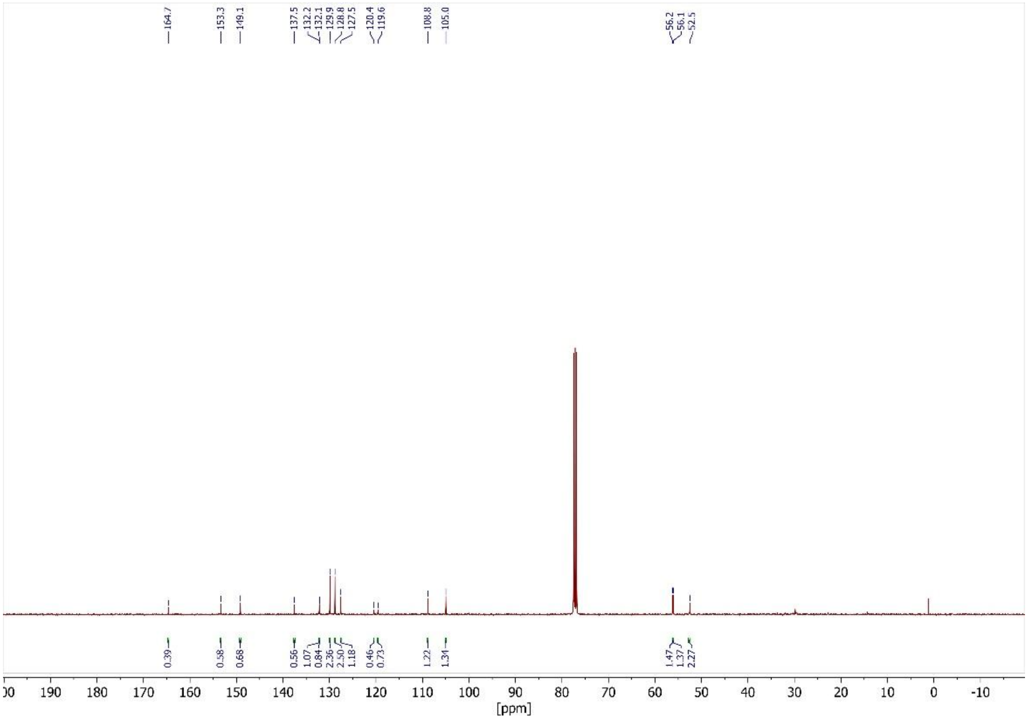

Figure S83. $^{13}C\{^{1}H\}$ NMR spectrum of **IC-8** in $CDCl_3$.

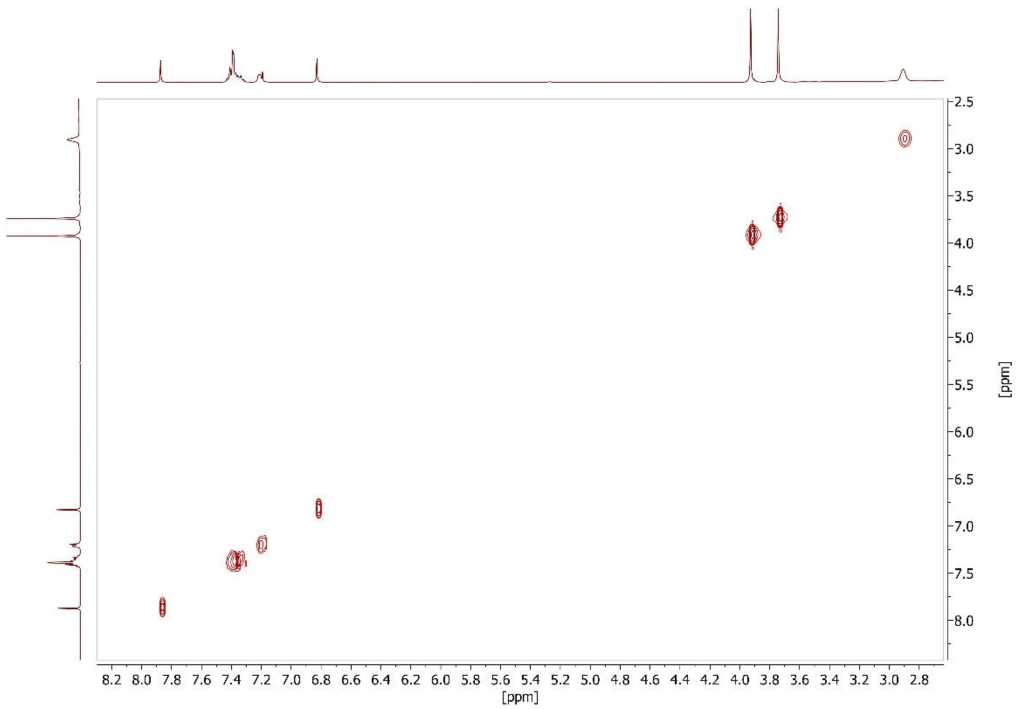

Figure S84. ^{1}H-COSY NMR spectrum of **IC-8** in $CDCl_3$.

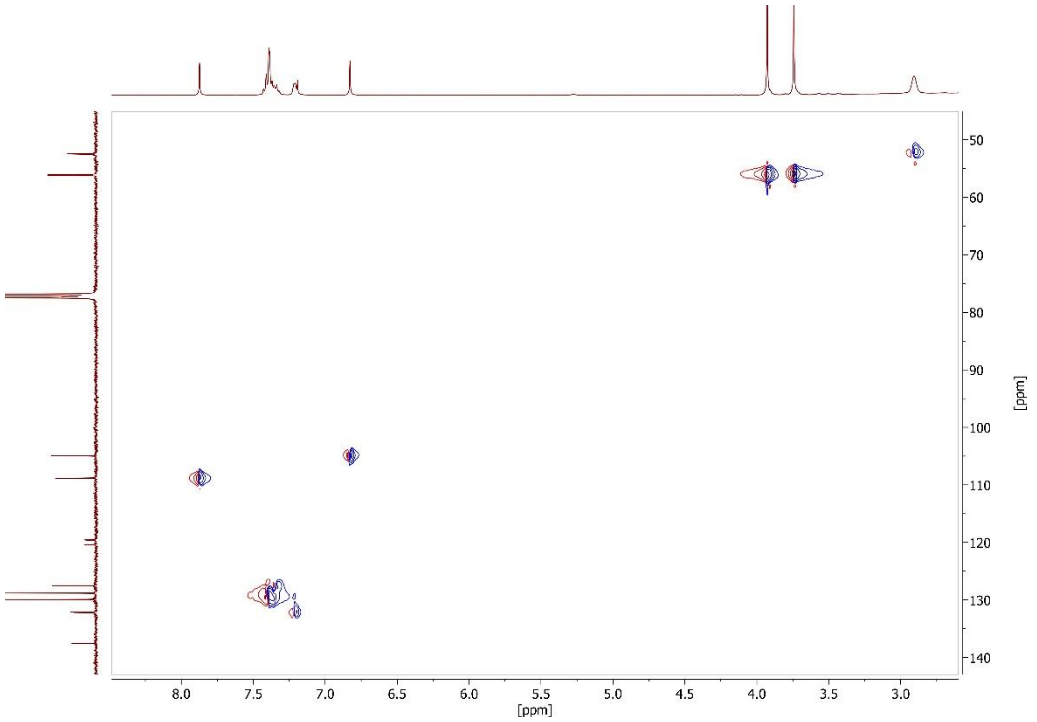

Figure S85. HSQC NMR spectrum of **IC-8** in $CDCl_3$.

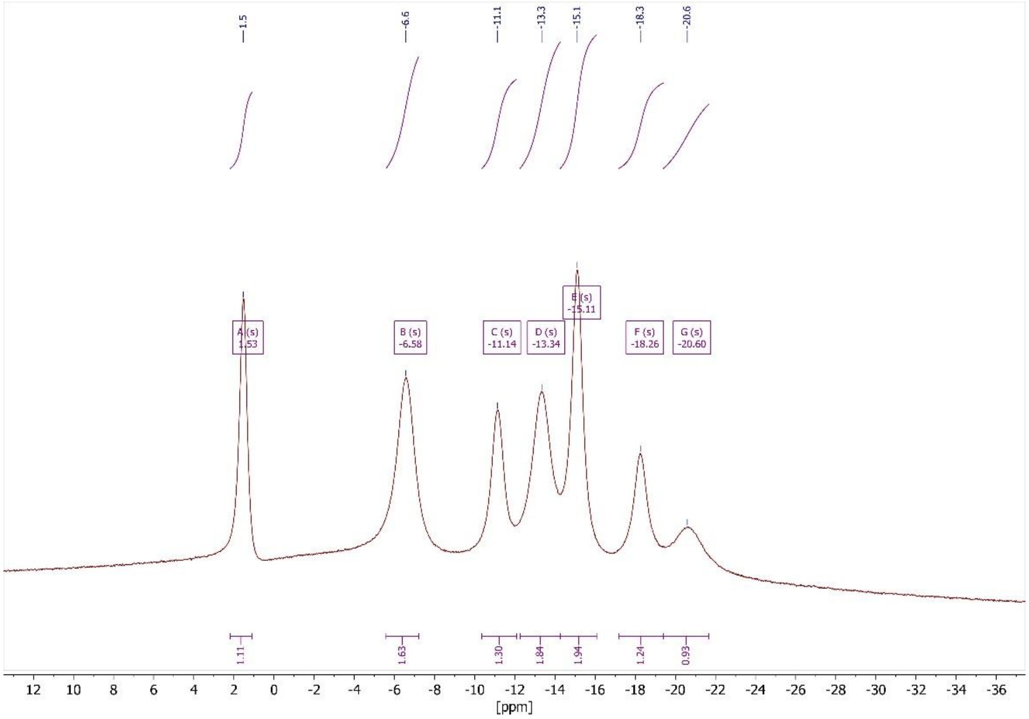

Figure S86. $^{11}B\{^1H\}$ NMR spectrum of **IC-8** in $CDCl_3$.

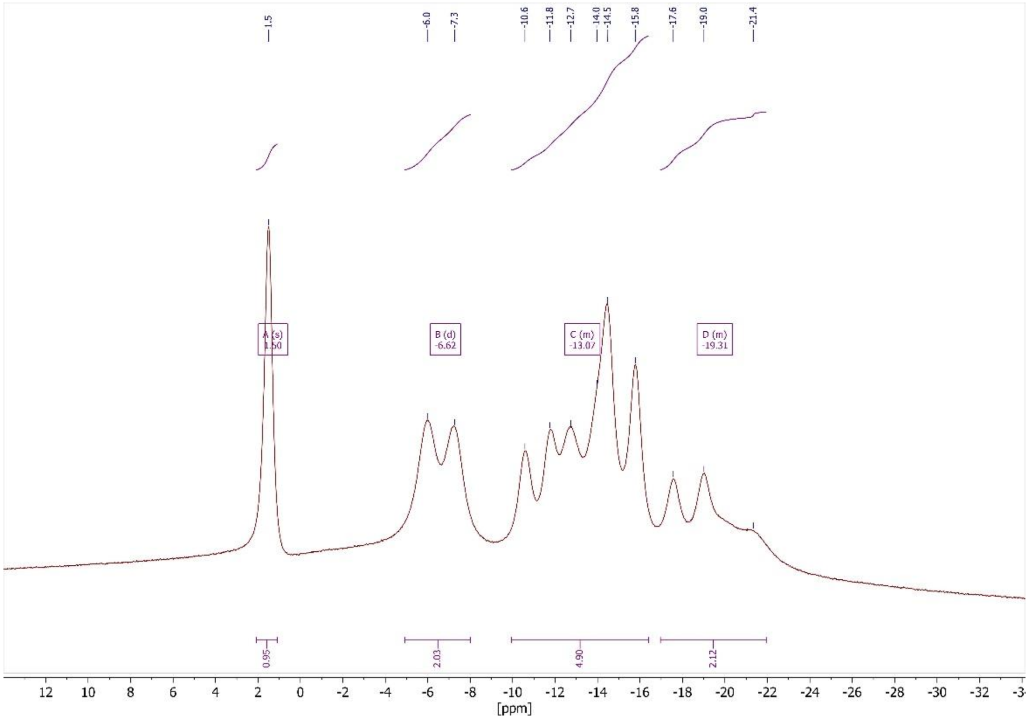

Figure S87. ^{11}B NMR spectrum of **IC-8** in $CDCl_3$.

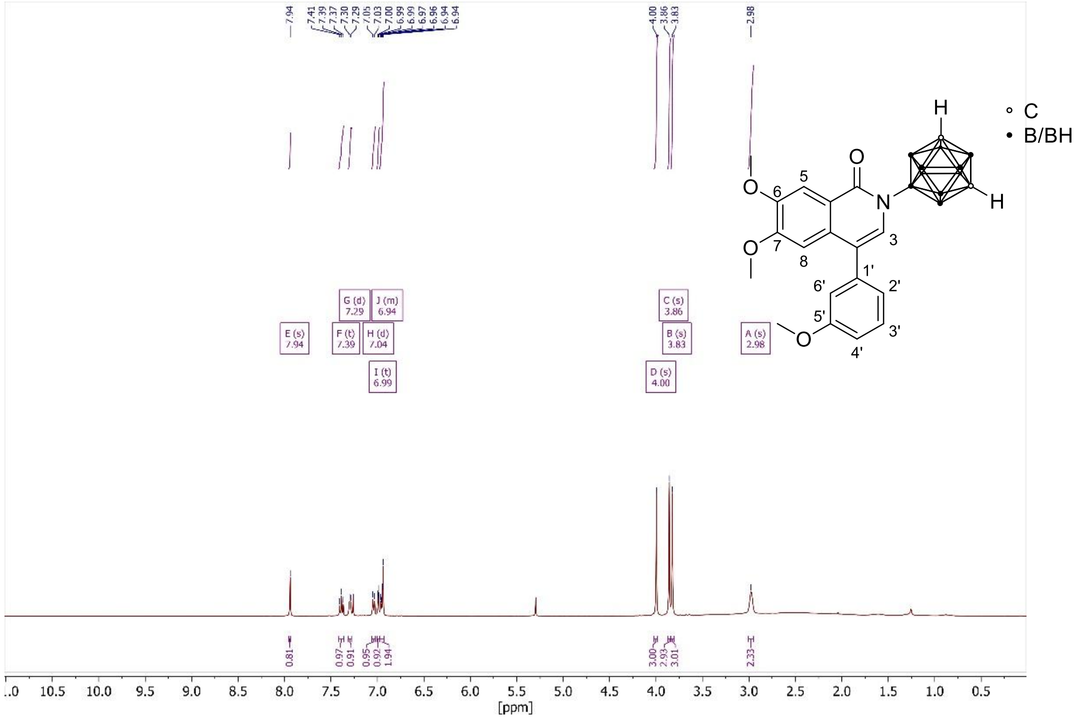

Figure S88. ^{1}H NMR spectrum of **IC-9** in $CDCl_3$.

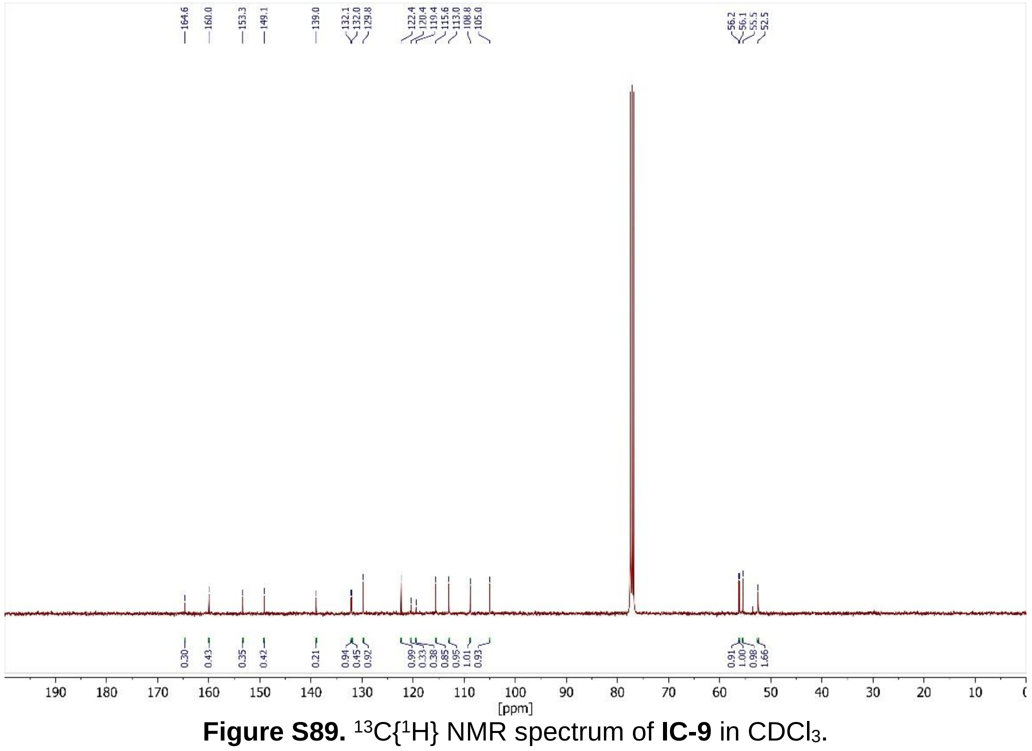

Figure S89. $^{13}C\{^{1}H\}$ NMR spectrum of **IC-9** in $CDCl_3$.

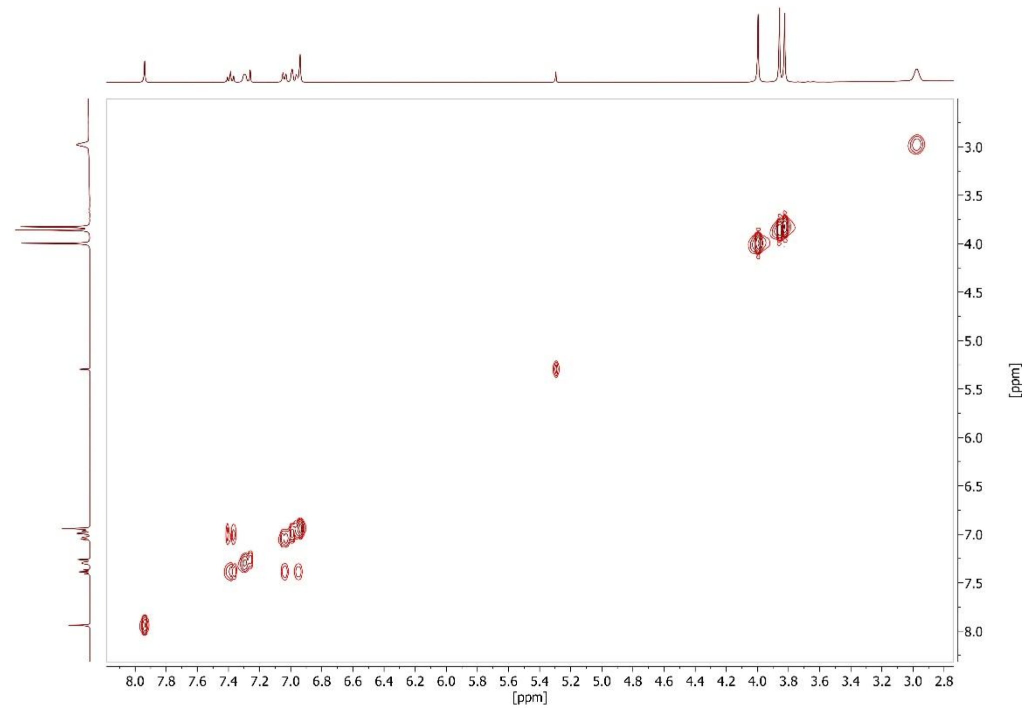

Figure S90. ¹H-COSY NMR spectrum of **IC-9** in $CDCl_3$.

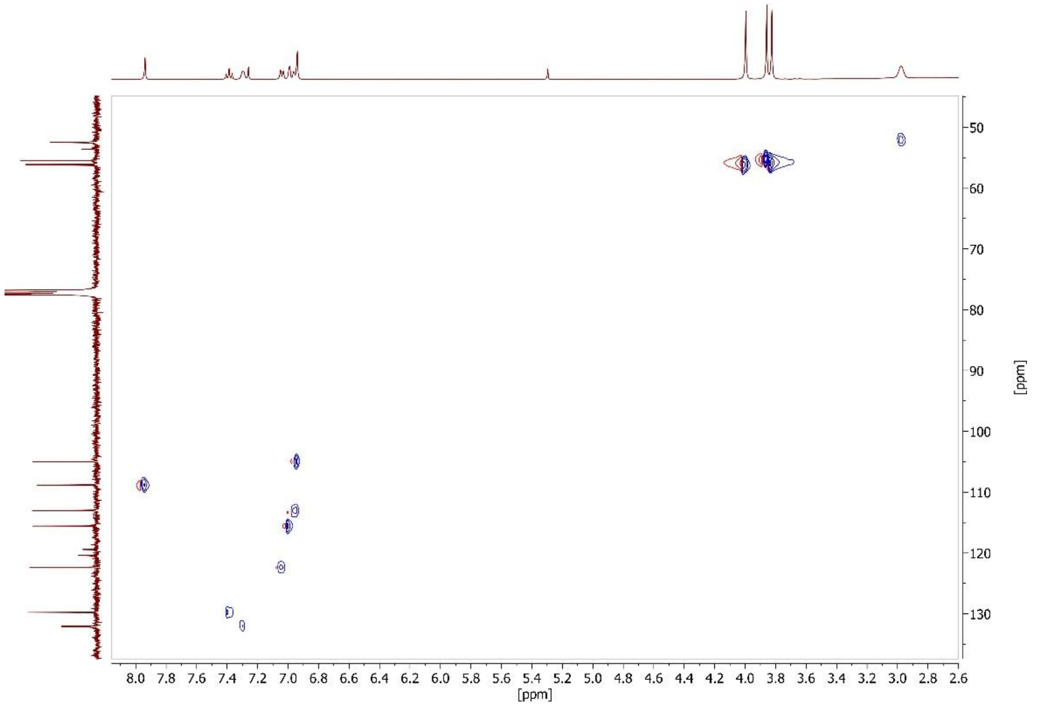

Figure S91. HSQC NMR spectrum of **IC-9** in $CDCl_3$.

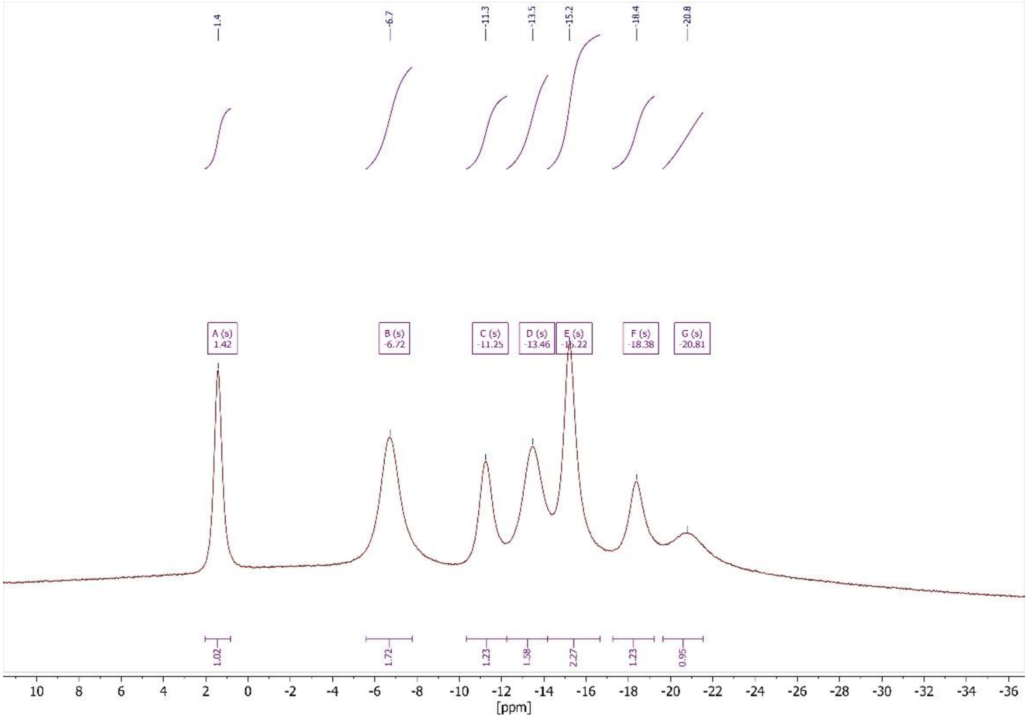

Figure S92. $^{11}B\{^{1}H\}$ NMR spectrum of **IC-9** in $CDCl_3$.

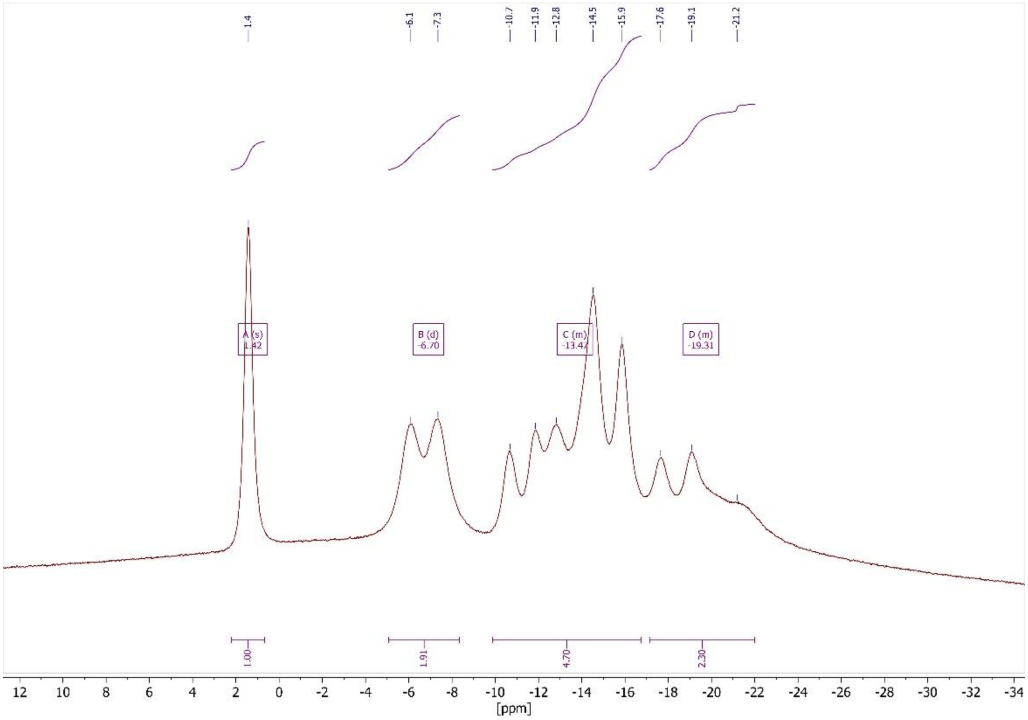

Figure S93. ^{11}B NMR spectrum of **IC-9** in $CDCl_3$.

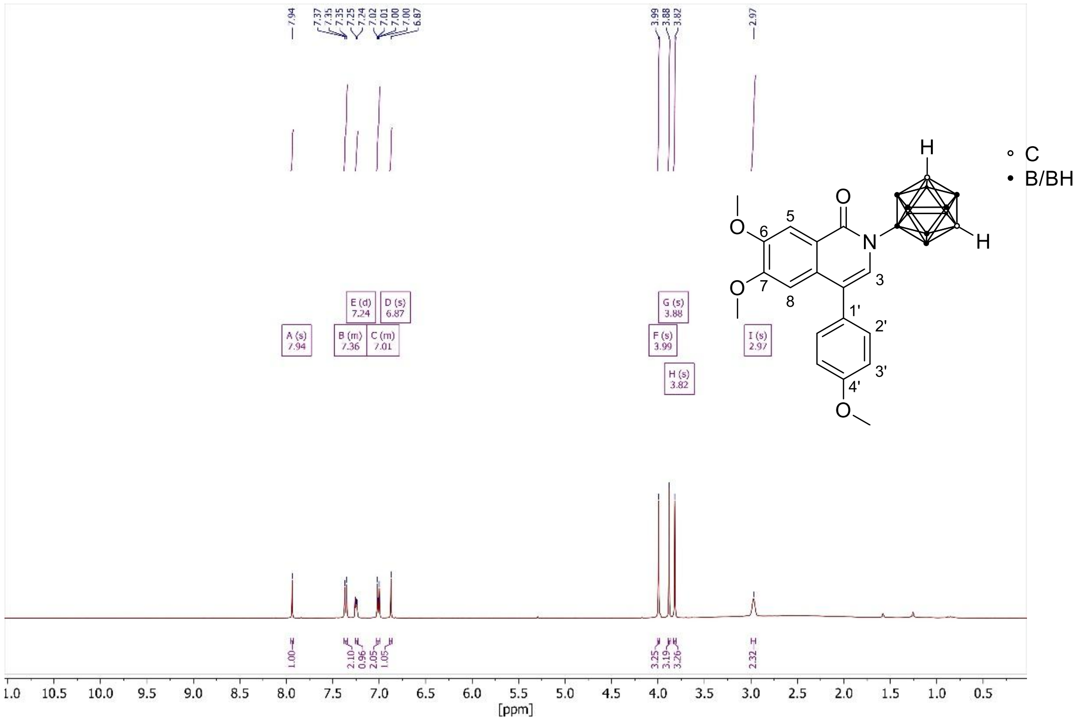

Figure S94. ^{1}H NMR spectrum of **IC-10** in $CDCl_3$.

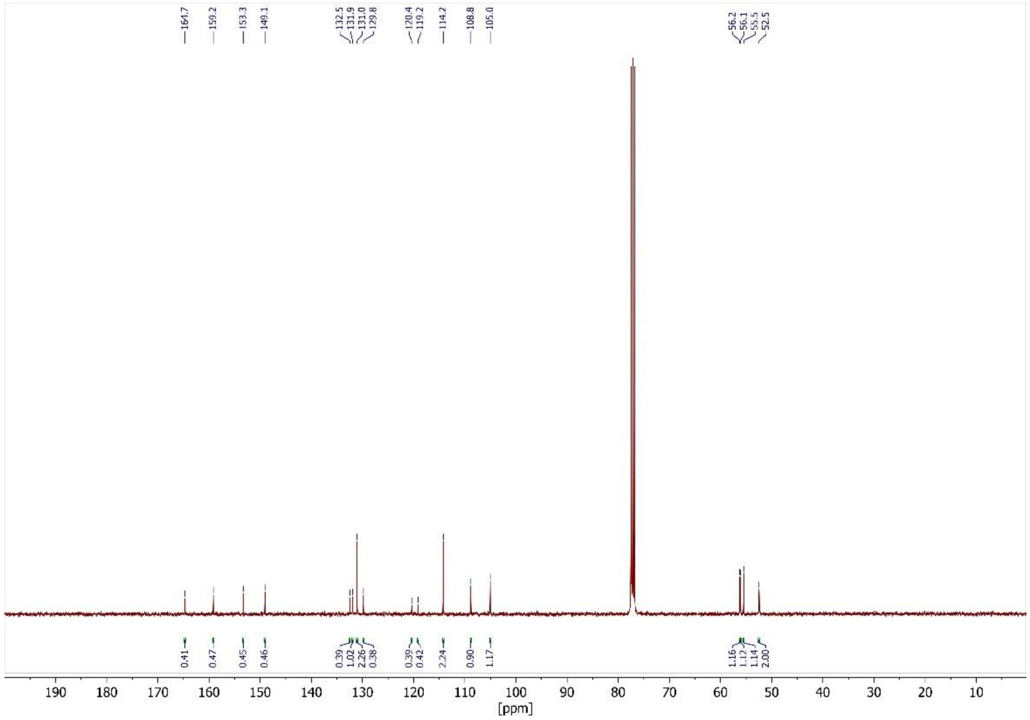

Figure S95. $^{13}C\{^{1}H\}$ NMR spectrum of **IC-10** in $CDCl_3$.

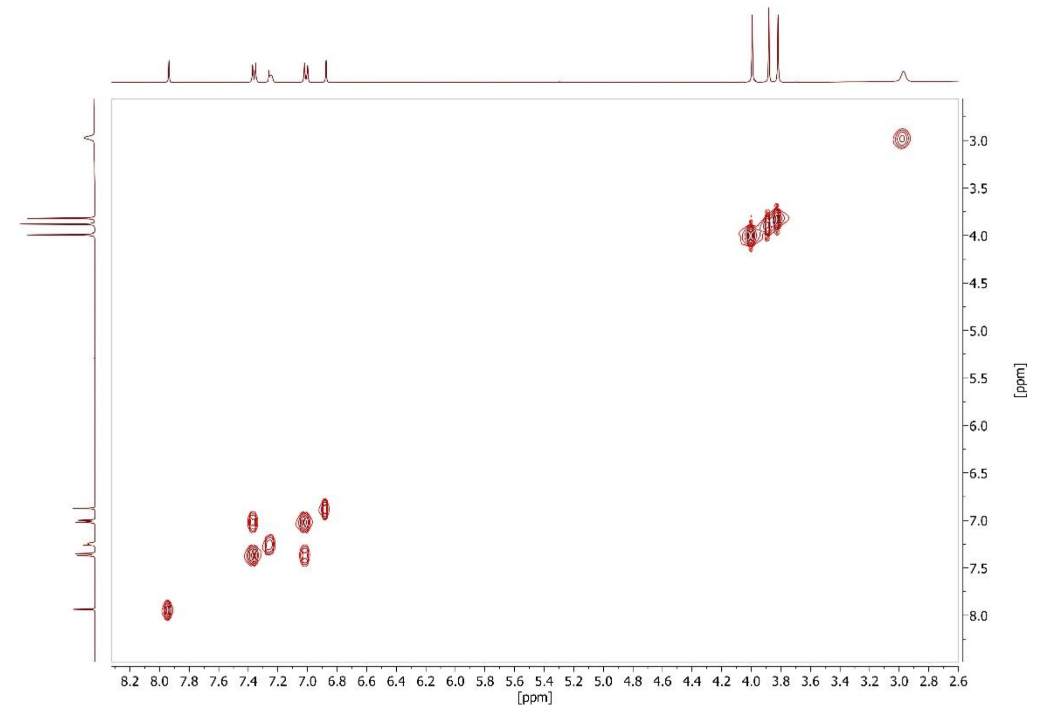

Figure S96. ^{1}H-COSY NMR spectrum of **IC-10** in $CDCl_3$.

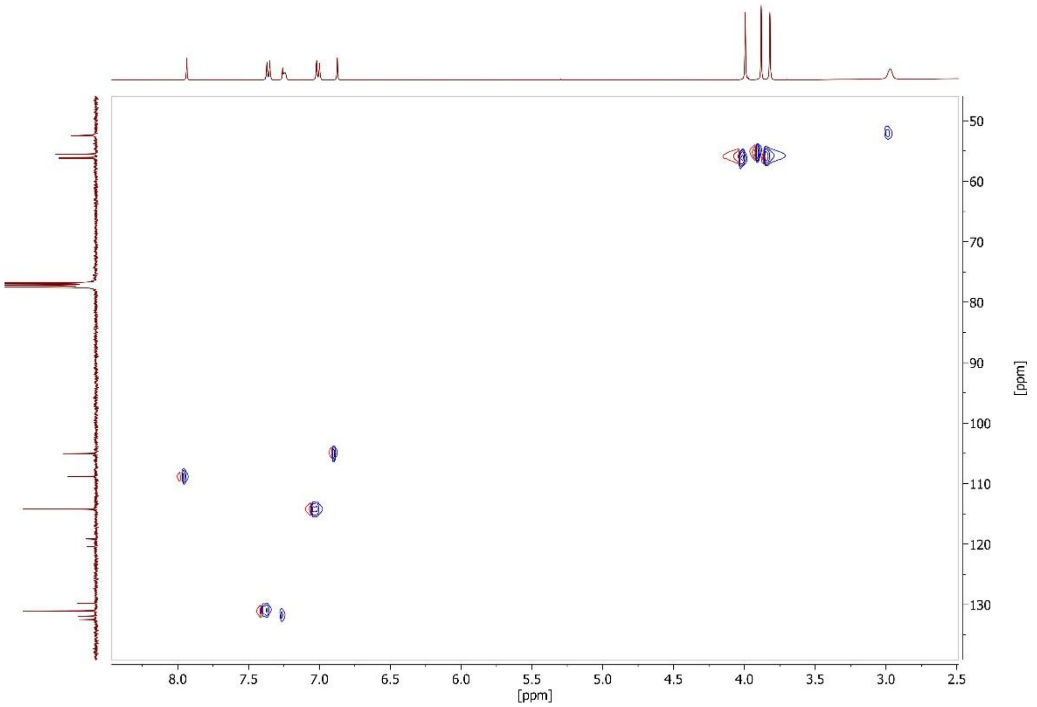

Figure S97. HSQC NMR spectrum of **IC-10** in $CDCl_3$.

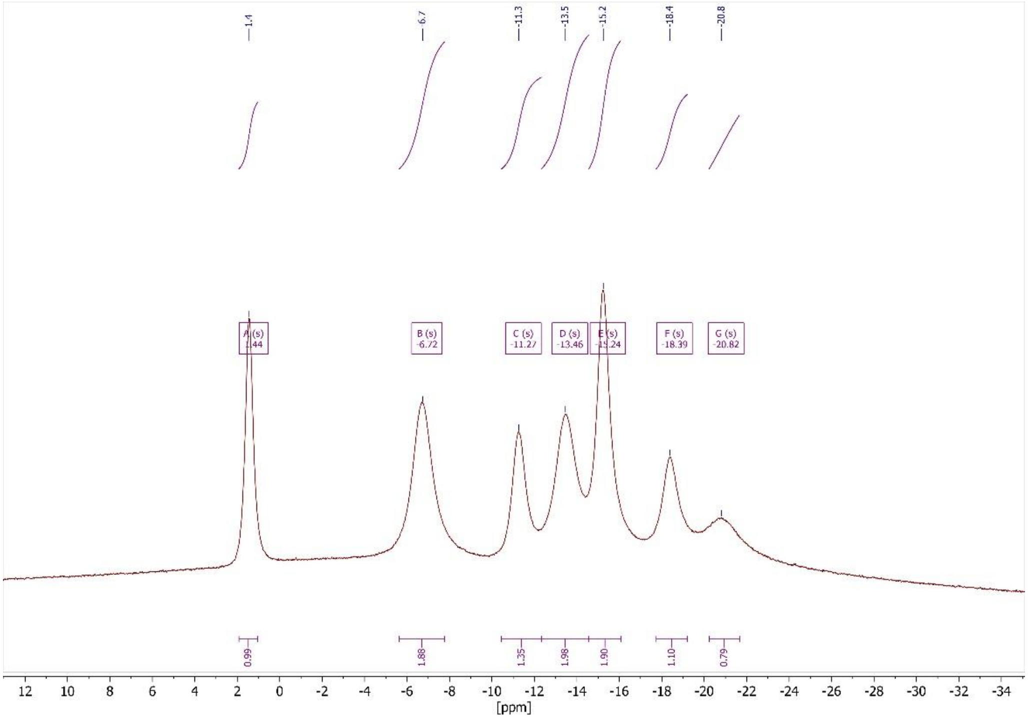

Figure S98. $^{11}B\{^{1}H\}$ NMR spectrum of **IC-10** in $CDCl_3$.

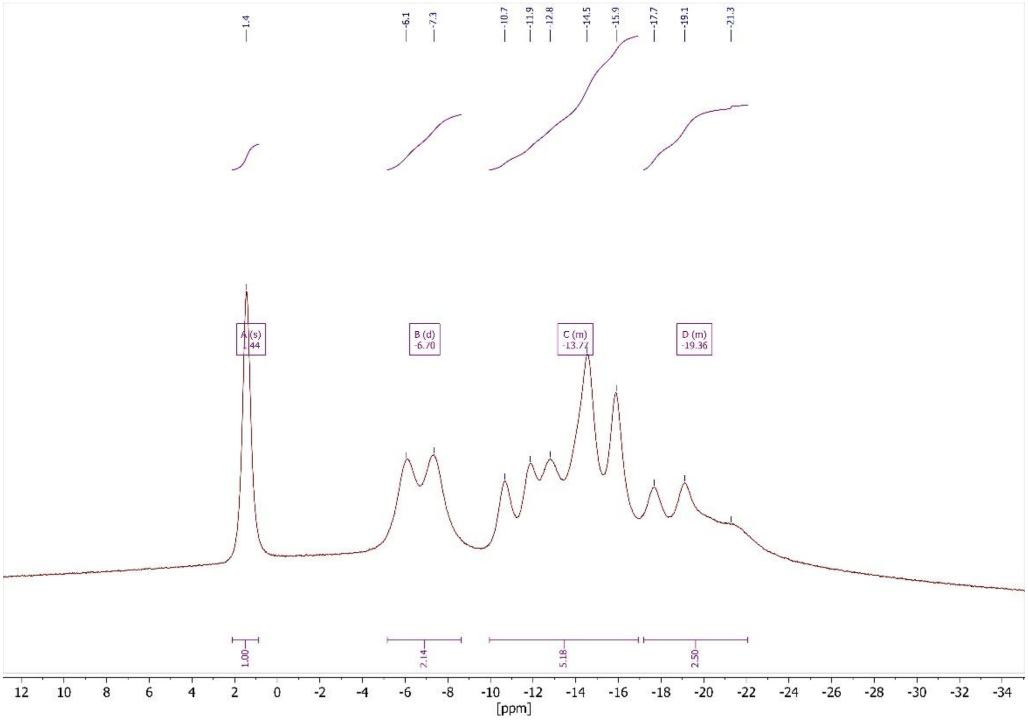

Figure S99. ^{11}B NMR spectrum of **IC-10** in $CDCl_3$.

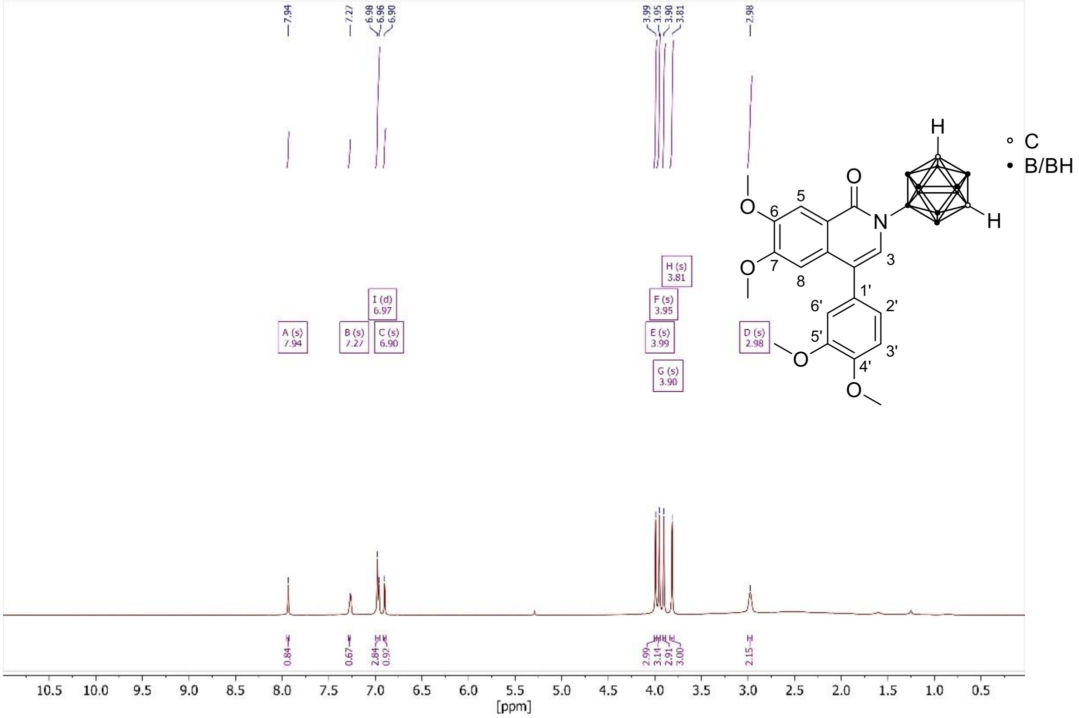

Figure S100. ^{1}H NMR spectrum of **IC-11** in $CDCl_3$.

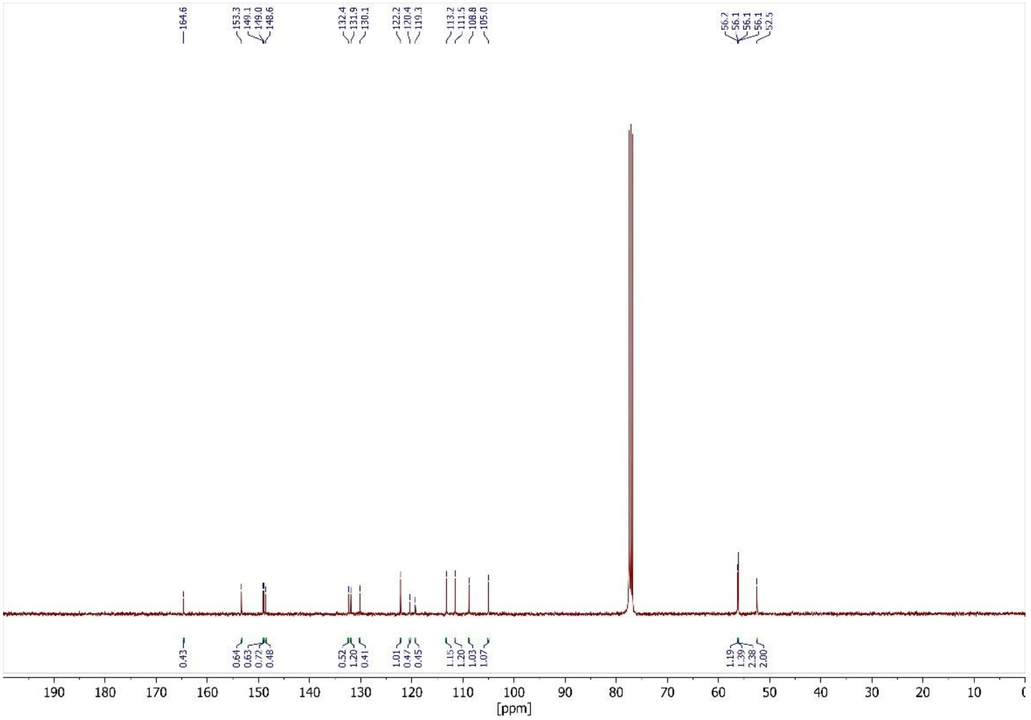

Figure S101. $^{13}C\{^{1}H\}$ NMR spectrum of **IC-11** in $CDCl_3$.

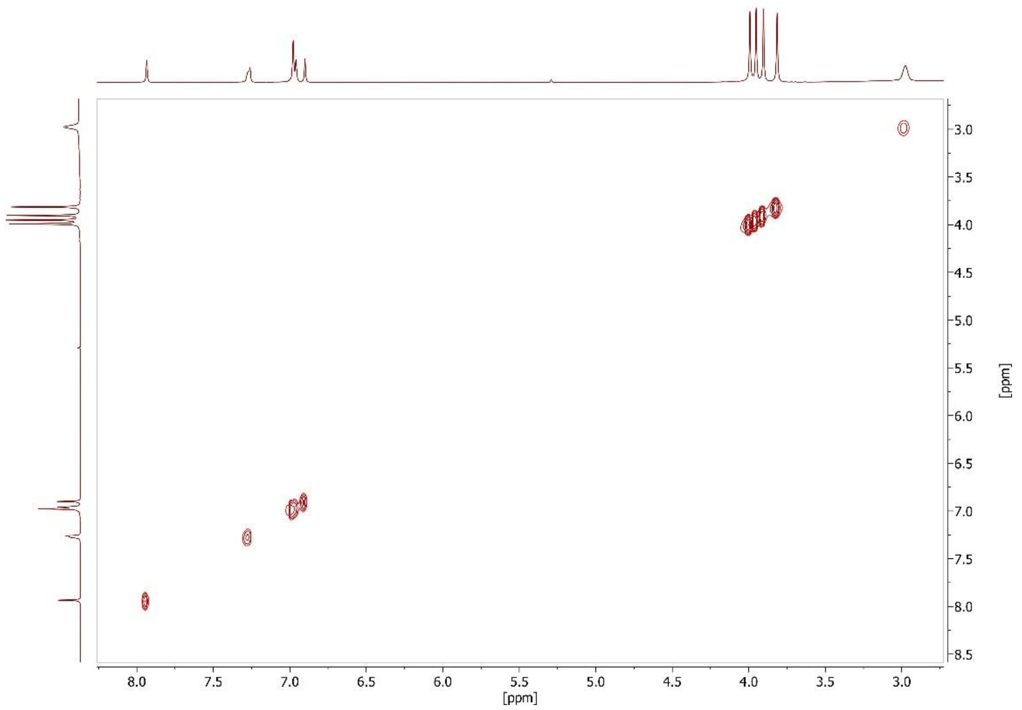

Figure S102. ^{1}H-COSY NMR spectrum of **IC-11** in $CDCl_3$.

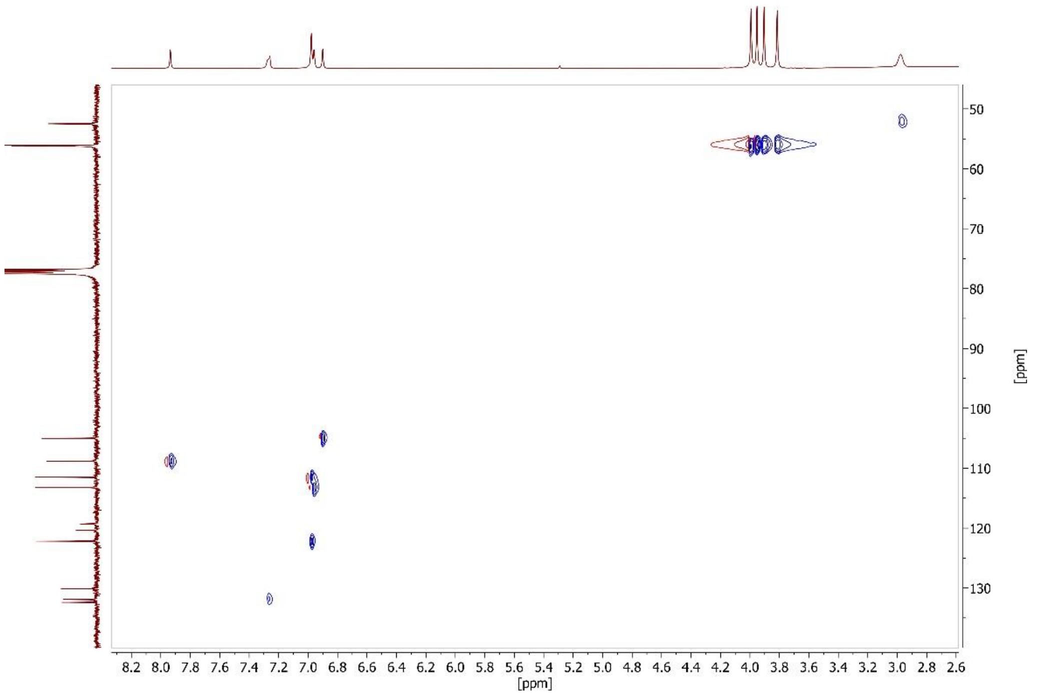

Figure S103. HSQC NMR spectrum of **IC-11** in $CDCl_3$.

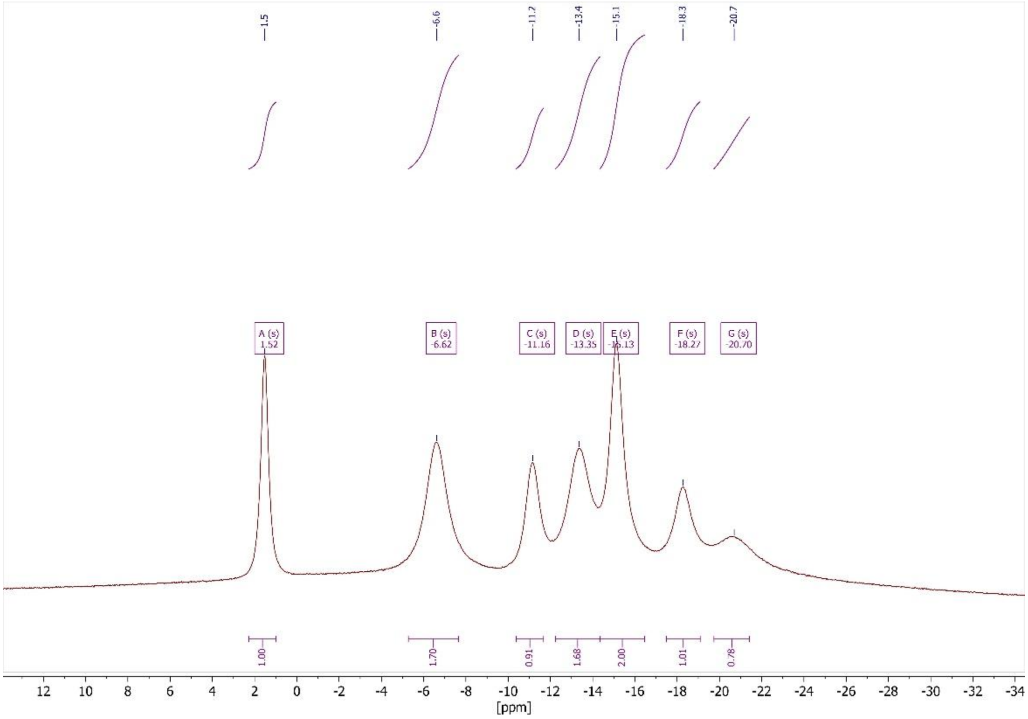

Figure S104. $^{11}B\{^{1}H\}$ NMR spectrum of **IC-11** in $CDCl_3$.

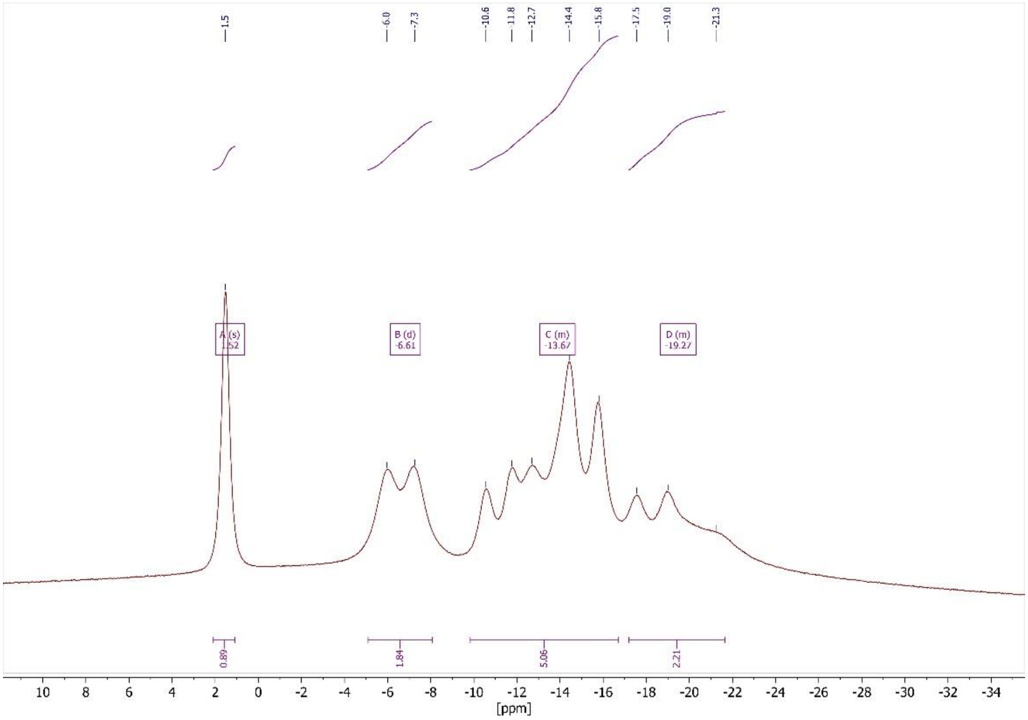

Figure S105. ^{11}B NMR spectrum of **IC-11** in $CDCl_3$.

2. Mass spectra

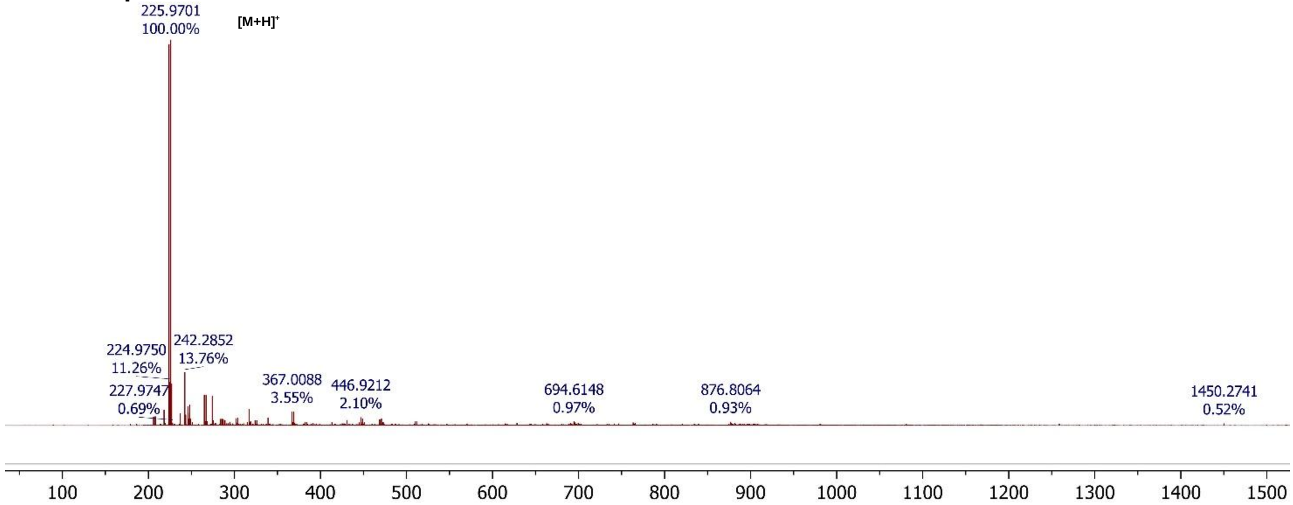

Figure S106. Mass (HR-ESI+) spectrum of **4** in CH_3CN.

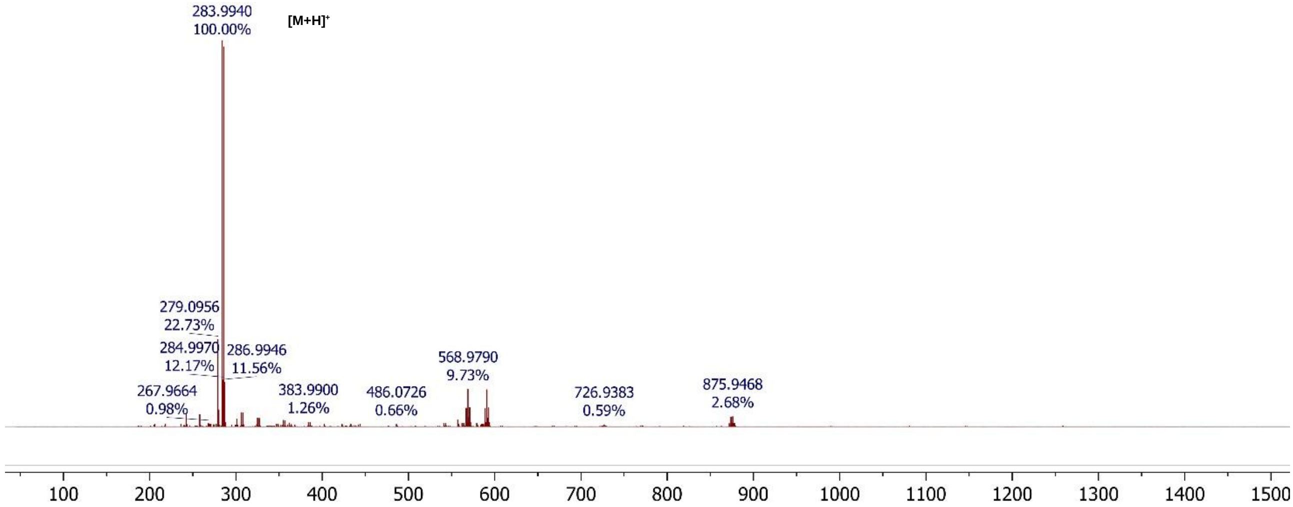

Figure S107. Mass (HR-ESI+) spectrum of **5** in CH_3CN.

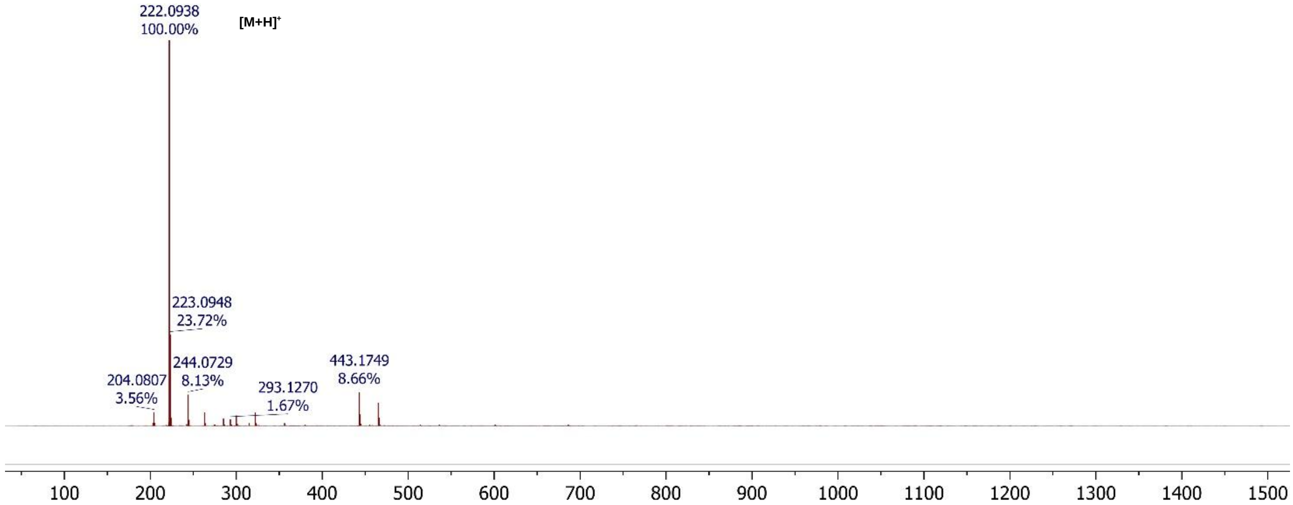

Figure S108. Mass (HR-ESI+) spectrum of **6a** in CH_3CN.

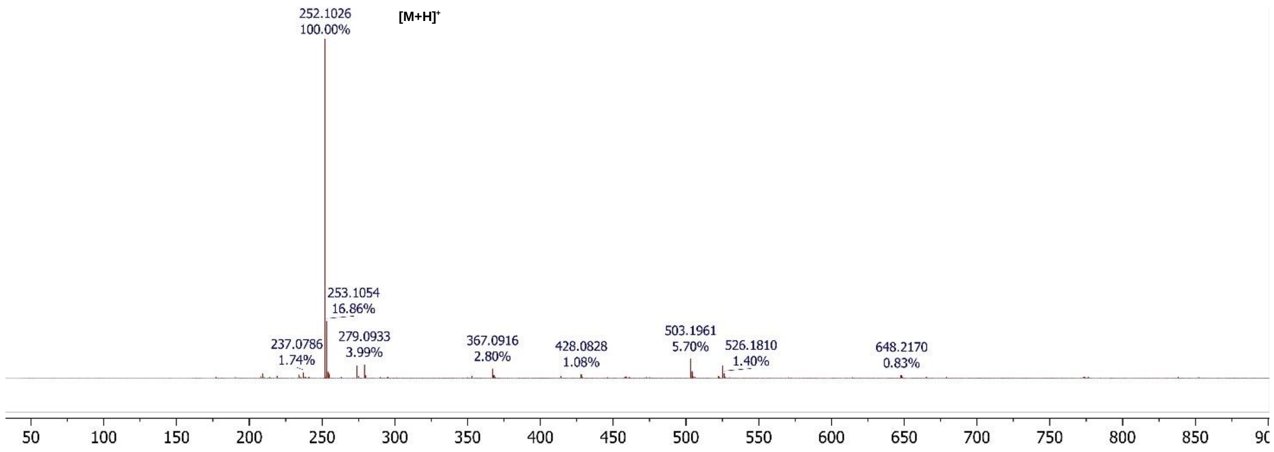

Figure S109. Mass (HR-ESI+) spectrum of **6b** in CH_3CN.

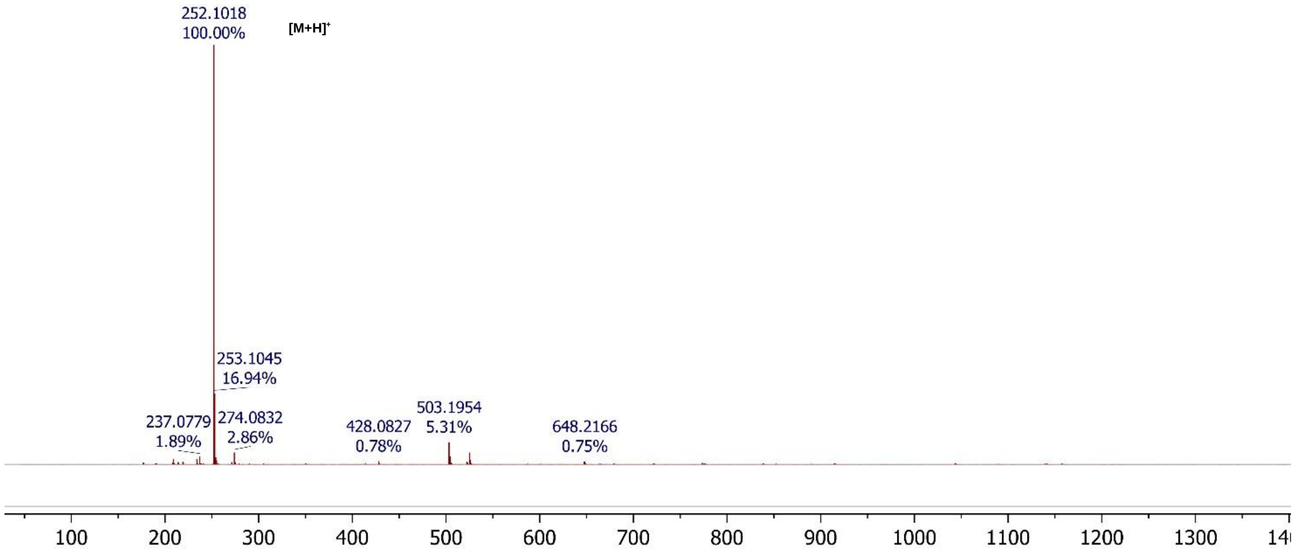

Figure S110. Mass (HR-ESI+) spectrum of **6c** in CH_3CN.

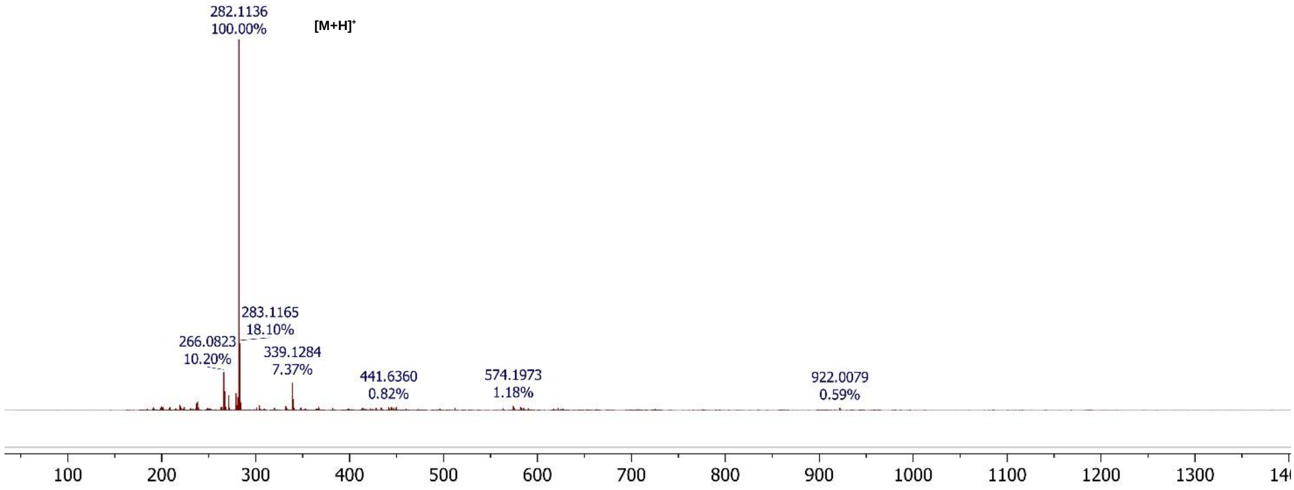

Figure S111. Mass (HR-ESI+) spectrum of **6d** in CH_3CN.

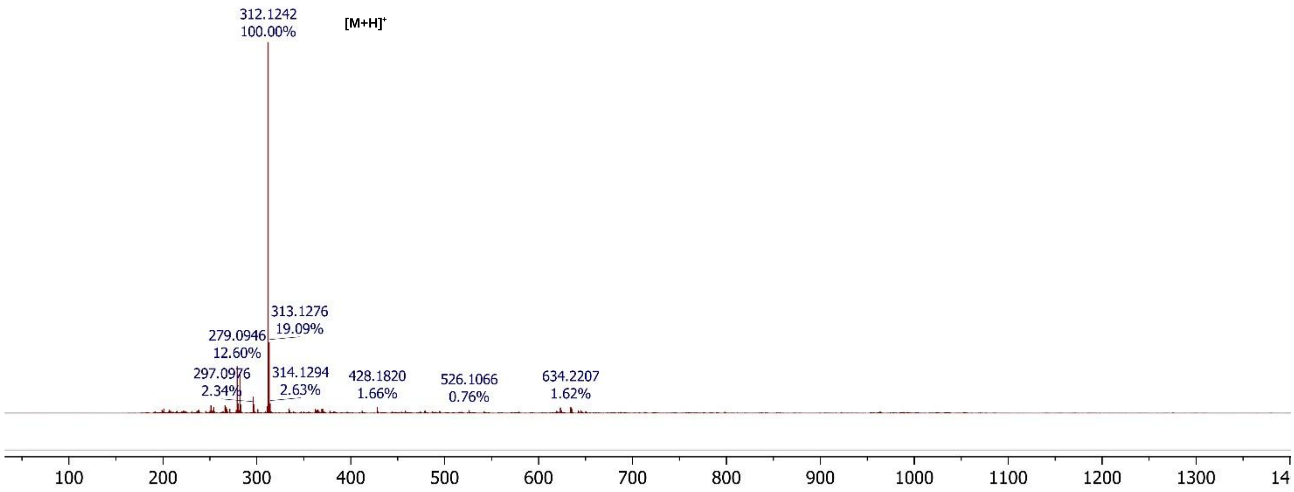

Figure S112. Mass (HR-ESI+) spectrum of **6e** in CH_3CN.

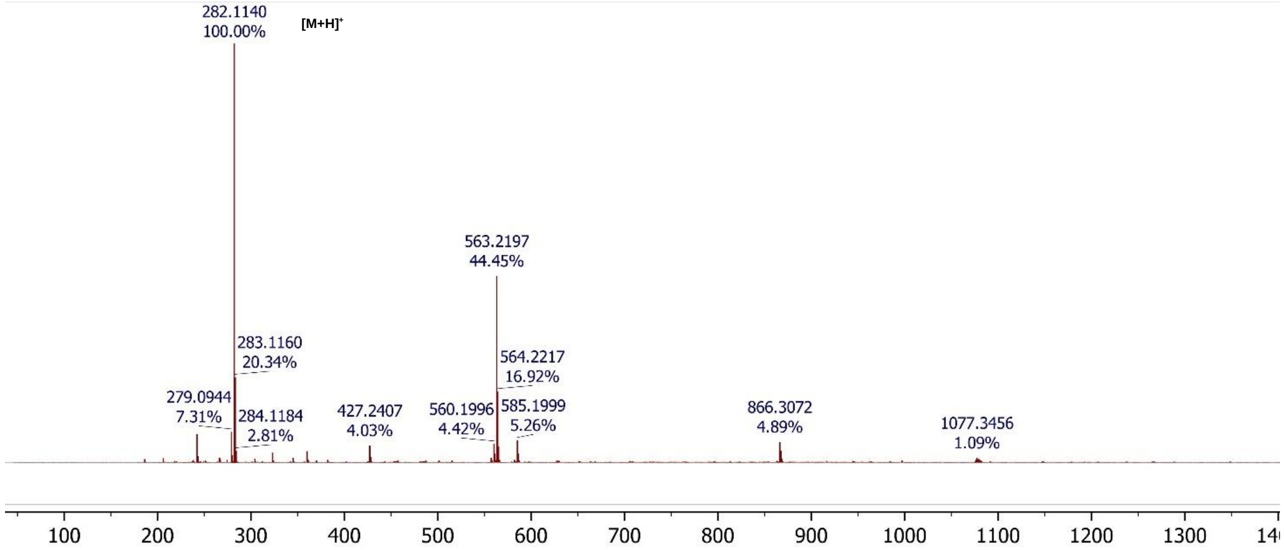

Figure S113. Mass (HR-ESI+) spectrum of **7a** in CH_3CN.

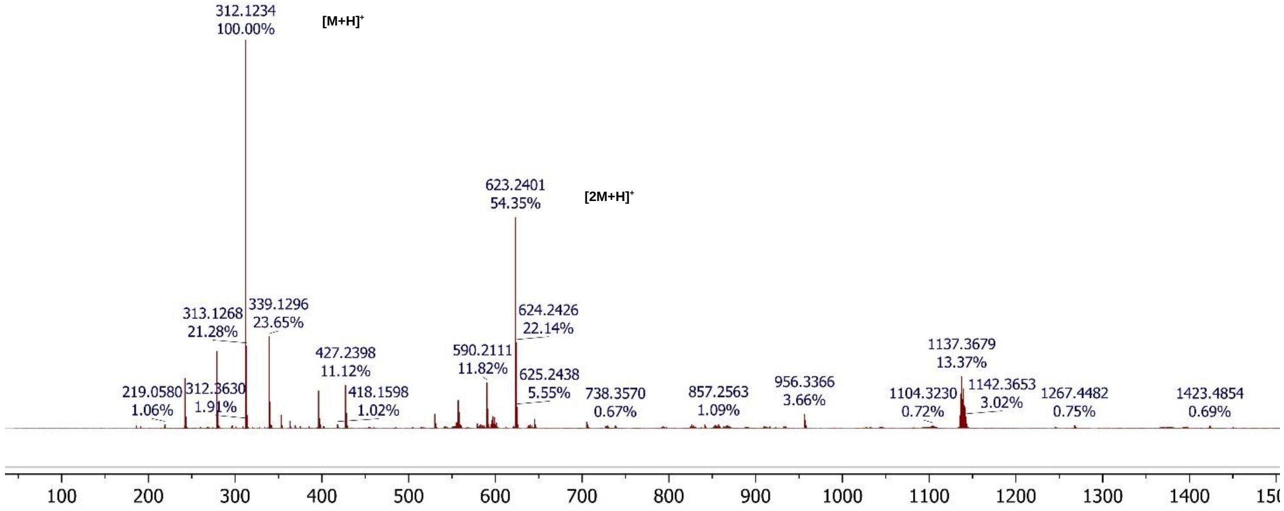

Figure S114. Mass (HR-ESI+) spectrum of **7b** in CH_3CN.

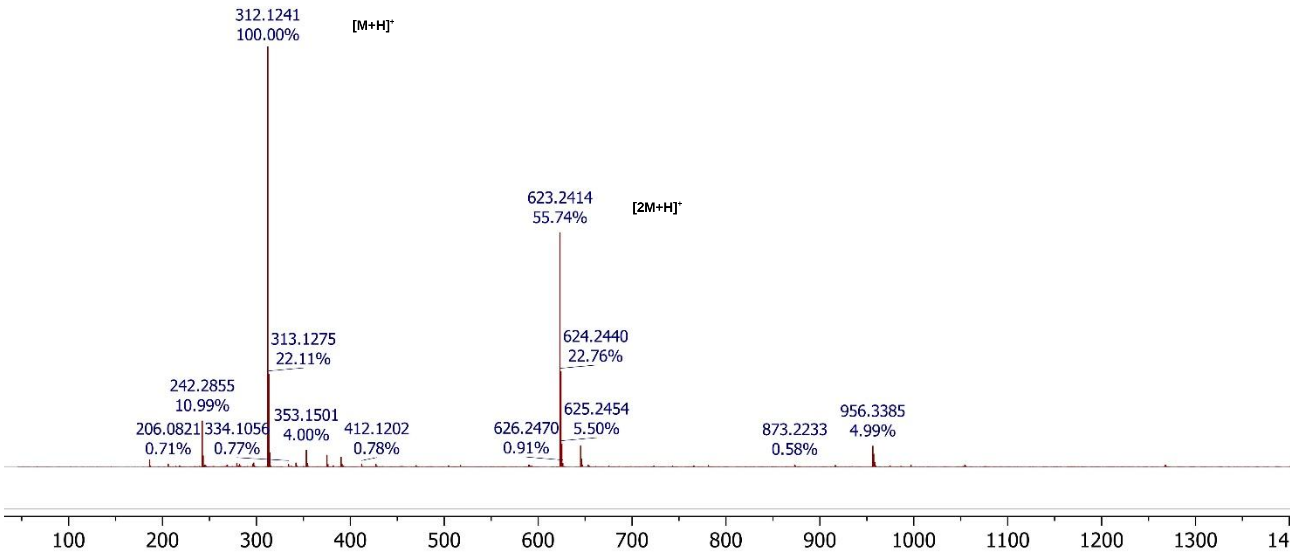

Figure S115. Mass (HR-ESI+) spectrum of **7c** in CH_3CN.

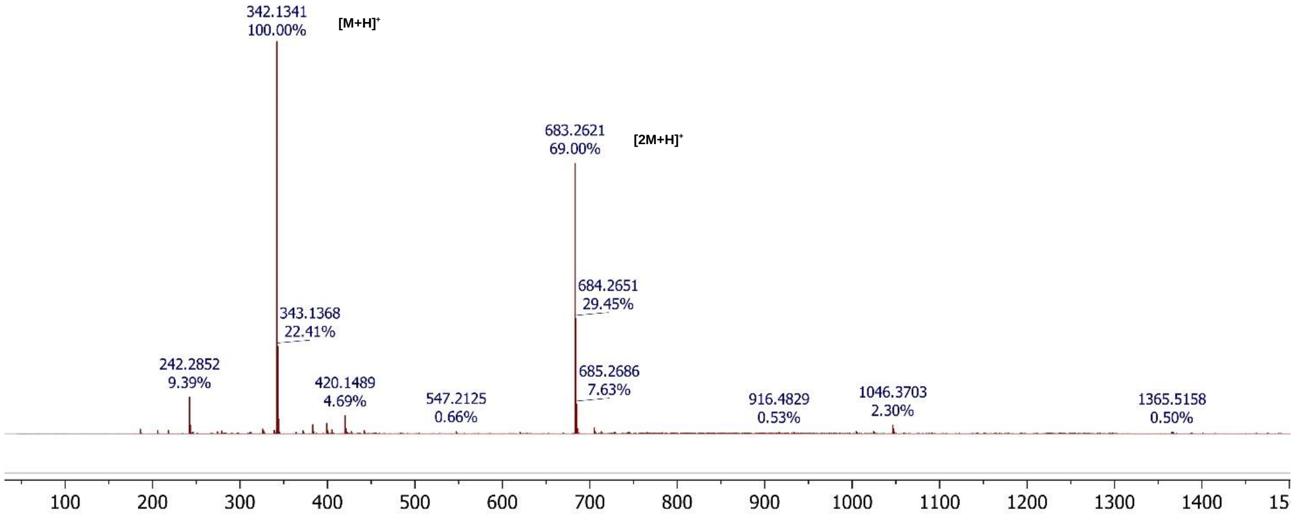

Figure S116. Mass (HR-ESI+) spectrum of **7d** in CH_3CN.

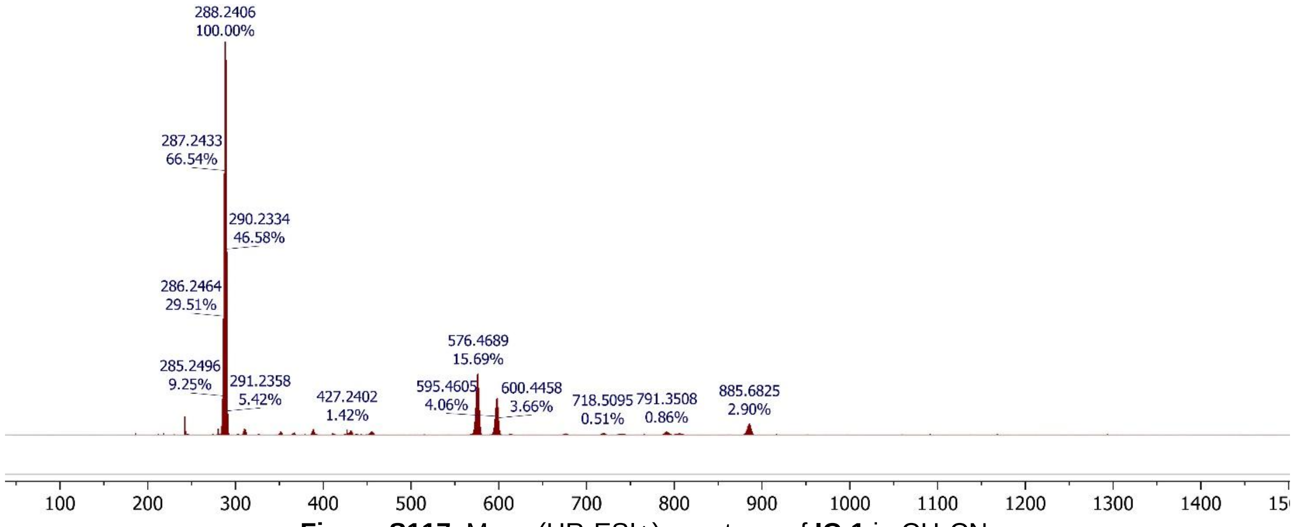

Figure S117. Mass (HR-ESI+) spectrum of **IC-1** in CH_3CN.

$[M+H]^+$

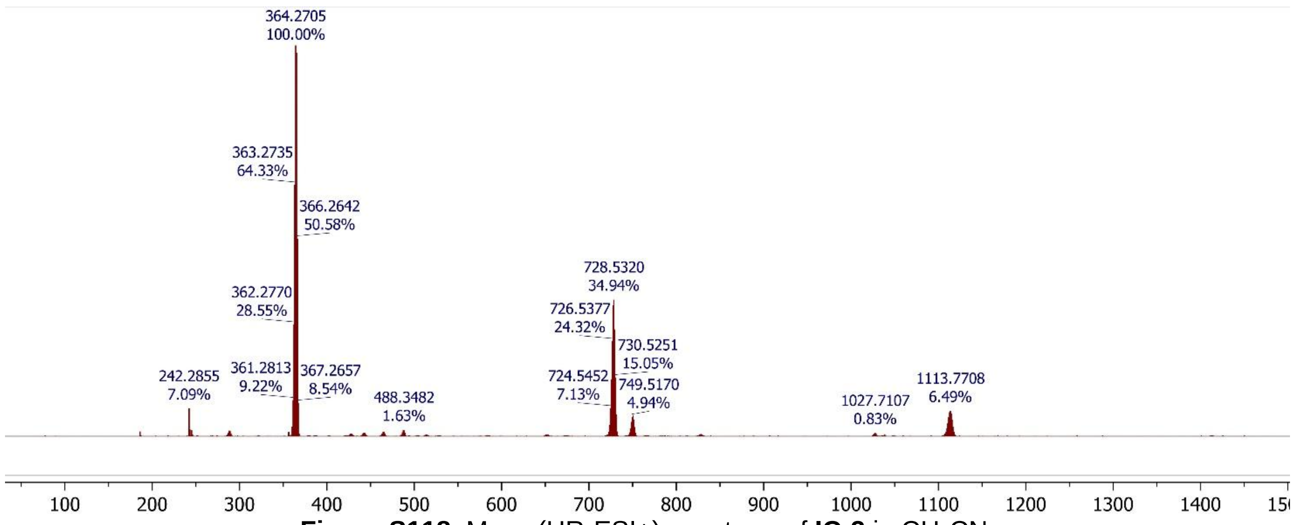

Figure S118. Mass (HR-ESI+) spectrum of **IC-2** in CH_3CN.

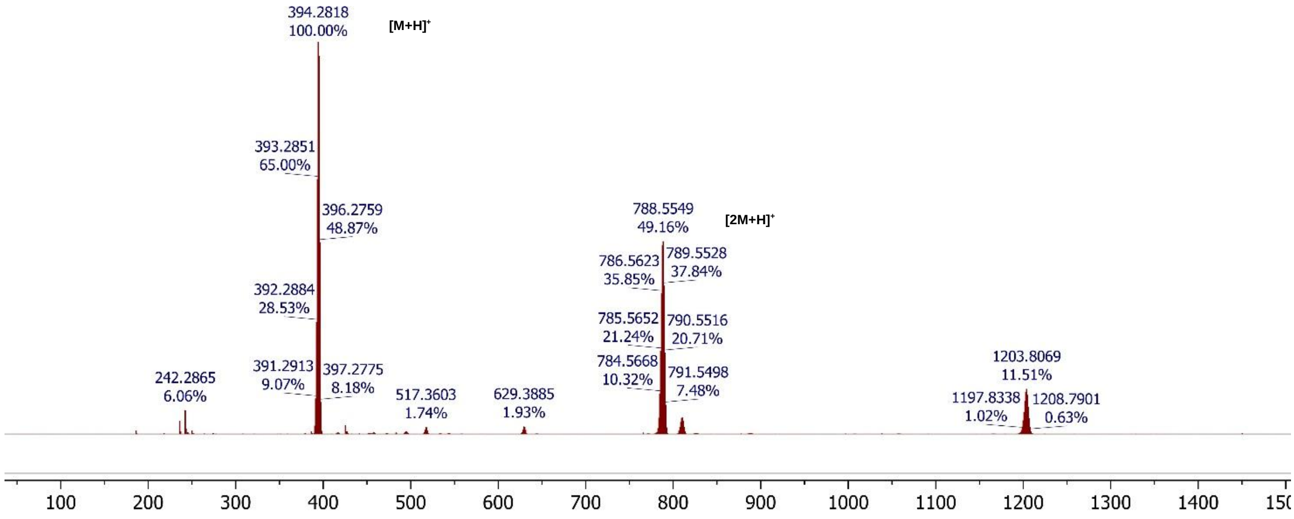

Figure S119. Mass (HR-ESI+) spectrum of **IC-3** in CH_3CN.

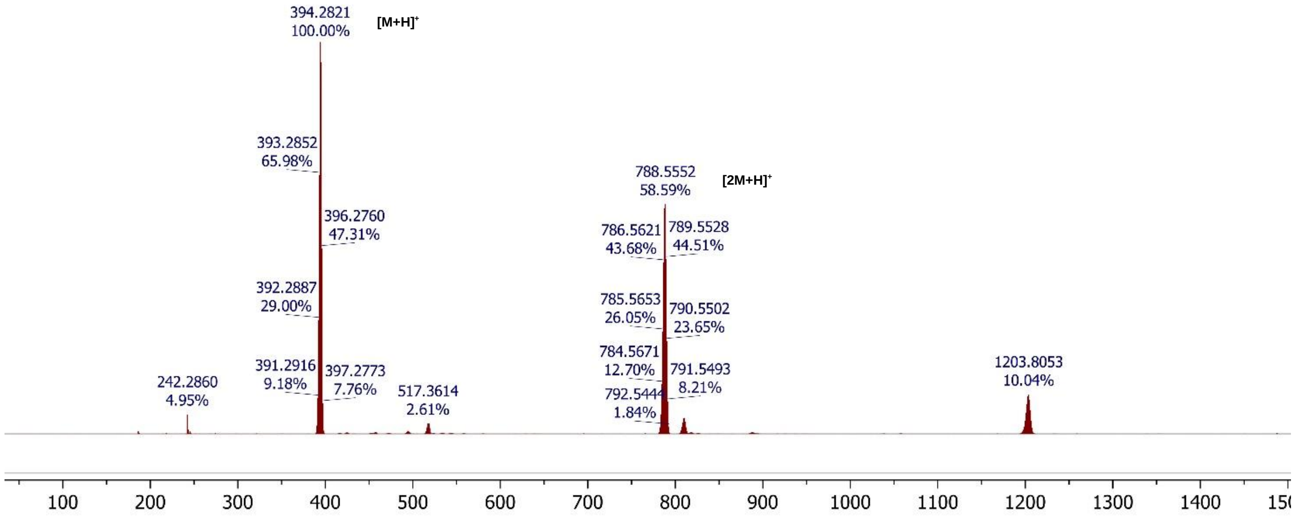

Figure S120. Mass (HR-ESI+) spectrum of **IC-4** in CH_3CN.

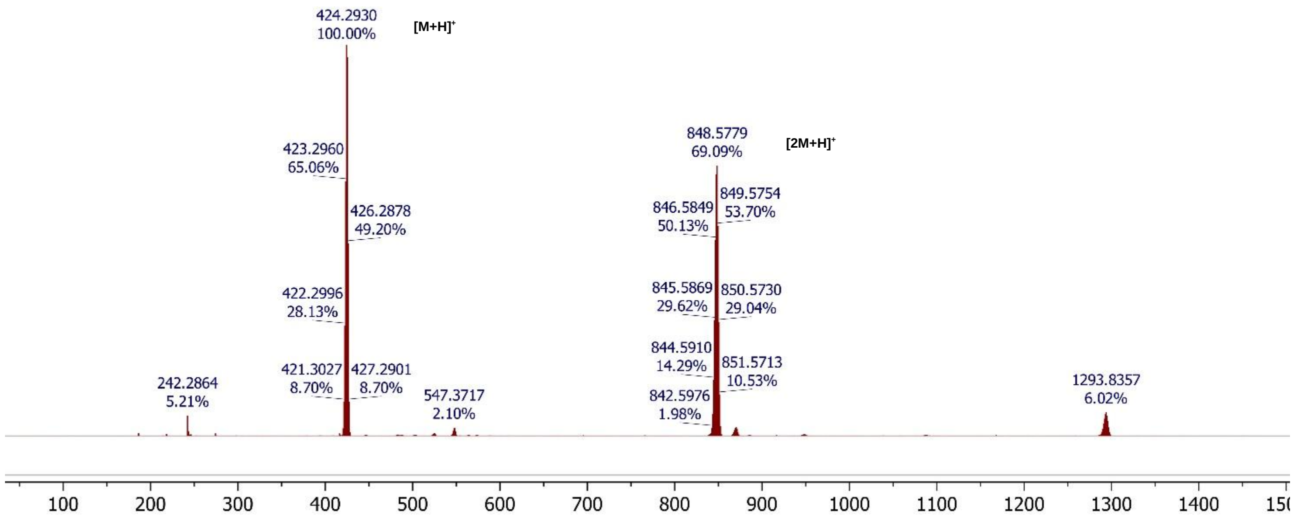

Figure S121. Mass (HR-ESI+) spectrum of **IC-5** in CH_3CN.

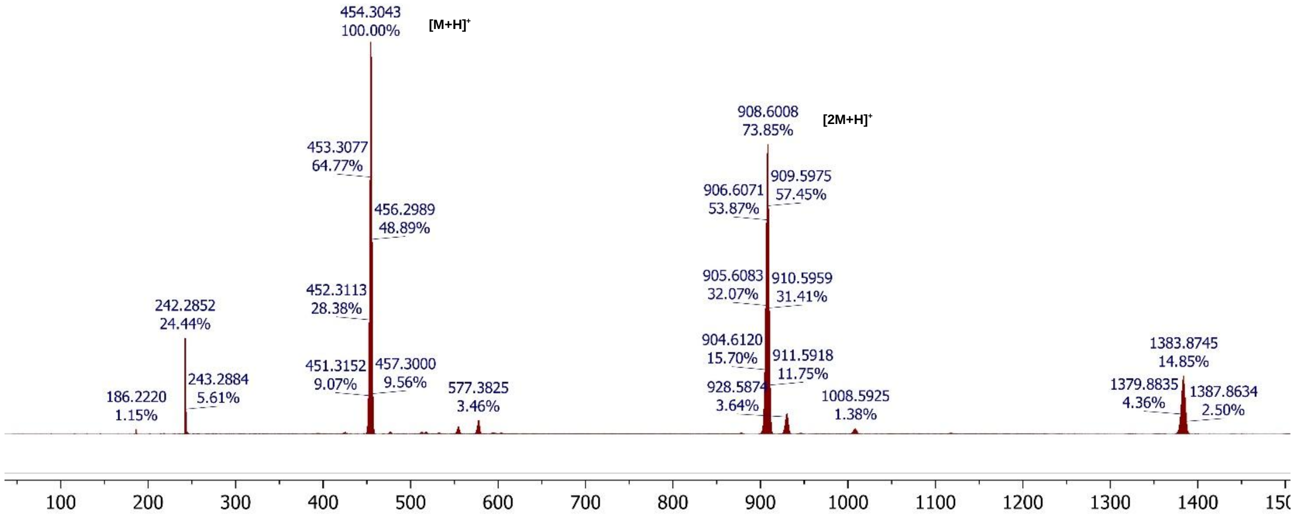

Figure S122. Mass (HR-ESI+) spectrum of **IC-6** in CH_3CN.

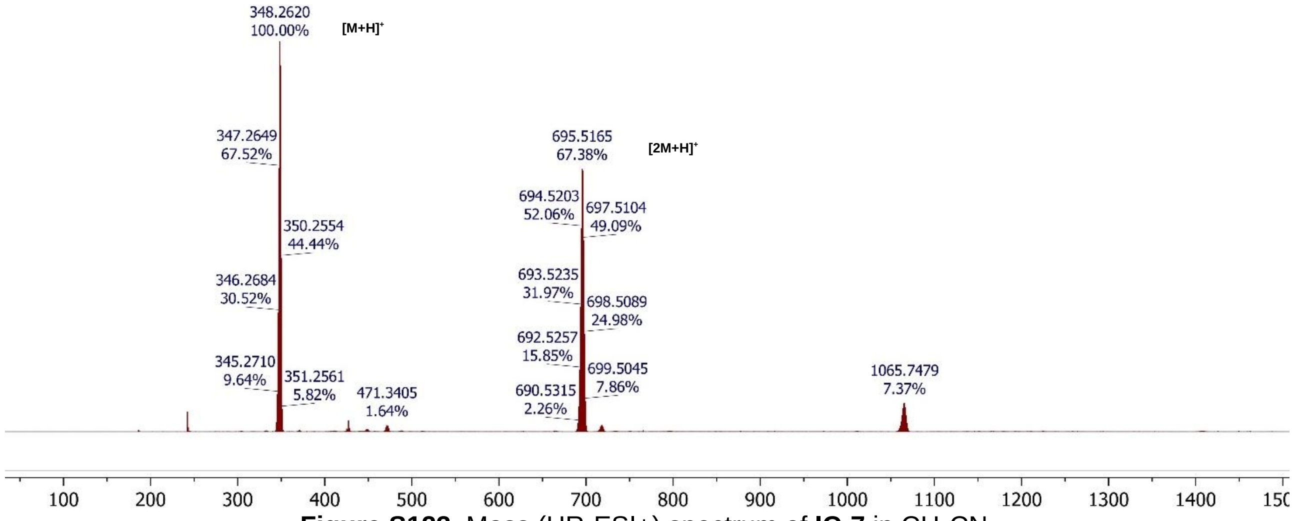

Figure S123. Mass (HR-ESI+) spectrum of **IC-7** in CH_3CN.

$[2M+H]^+$

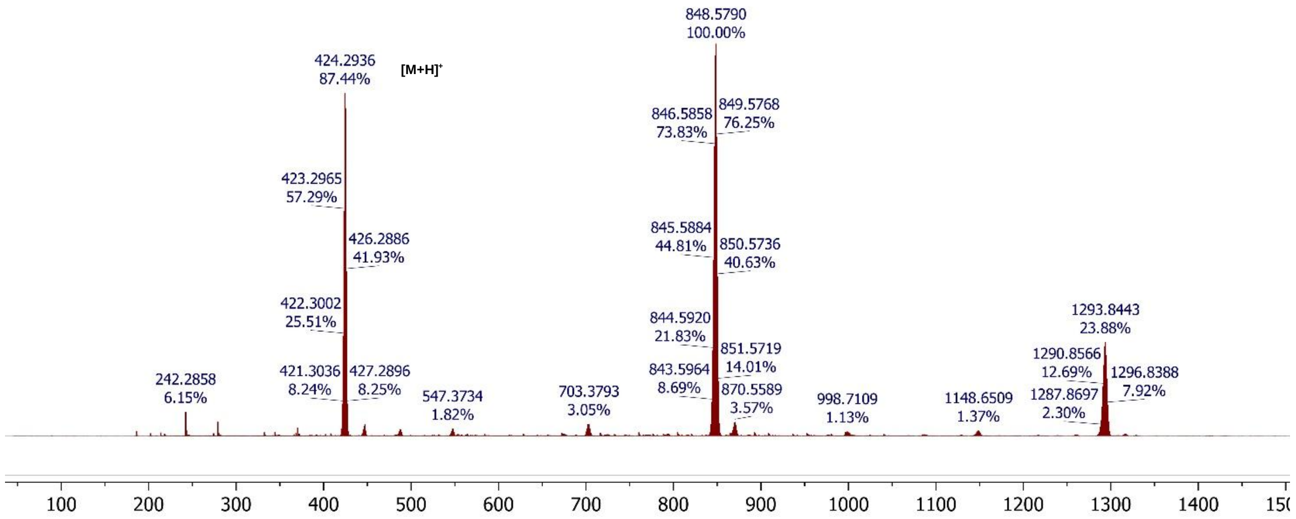

Figure S124. Mass (HR-ESI+) spectrum of **IC-8** in CH_3CN.

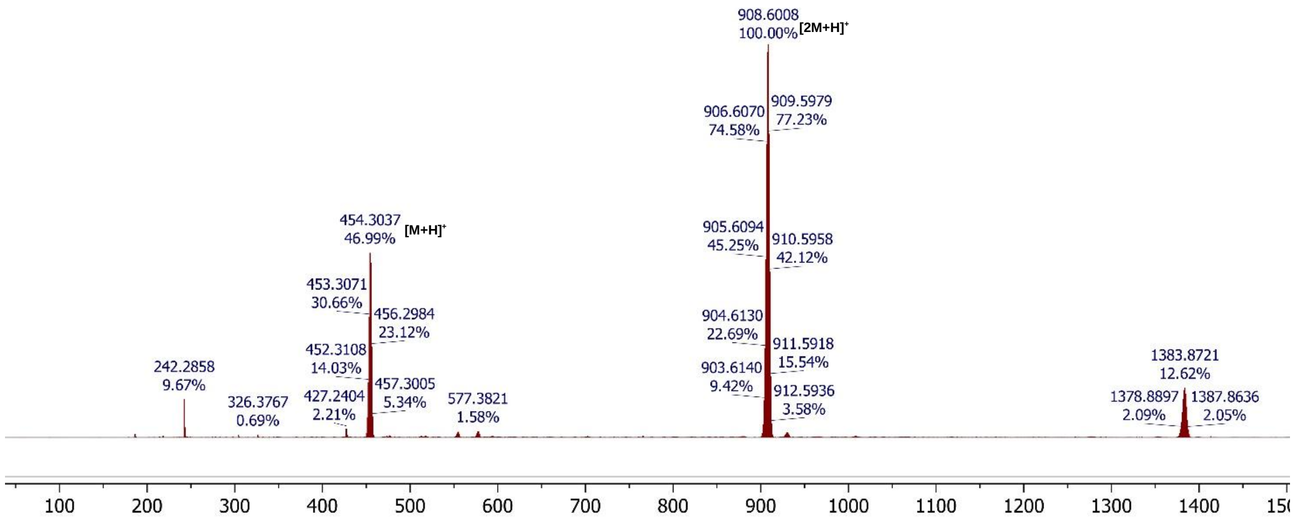

Figure S125. Mass (HR-ESI+) spectrum of **IC-9** in CH_3CN.

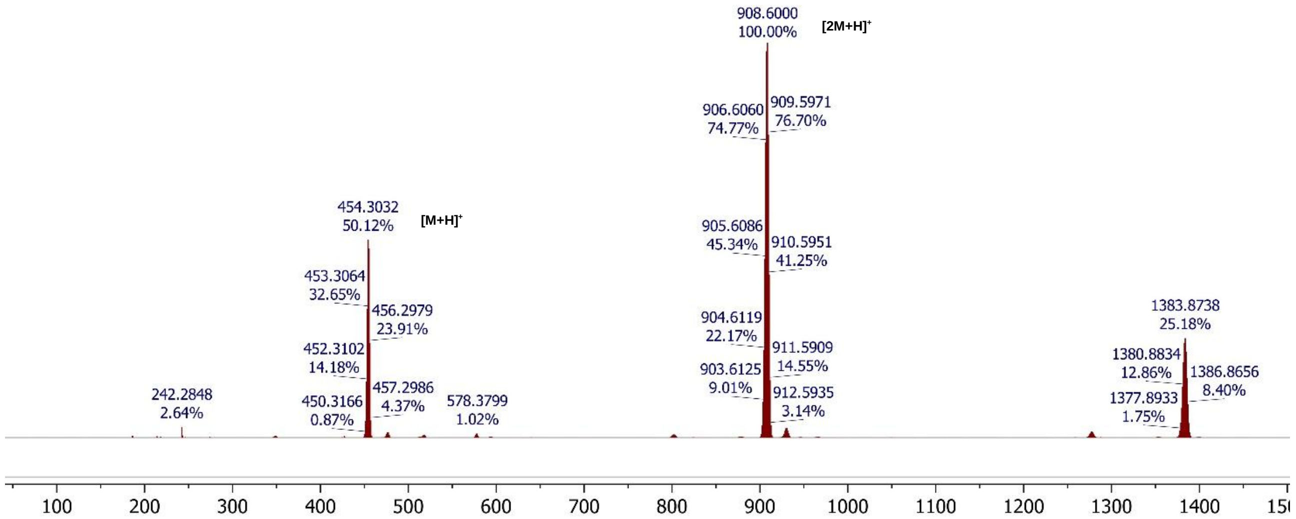

Figure S126. Mass (HR-ESI+) spectrum of **IC-10** in CH_3CN.

$[M+H]^+$

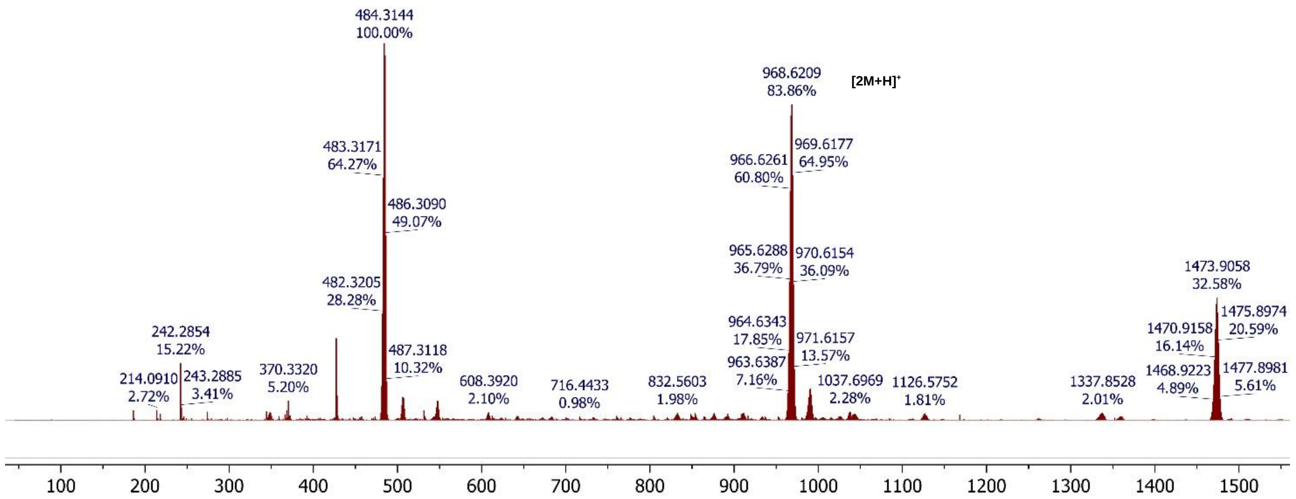

Figure S127. Mass (HR-ESI+) spectrum of **IC-11** in CH_3CN.

3. X-ray Crystallography: Molecular structures of IC-1, IC-2, IC-4, IC-8 and IC-10

Table S1: Fundamental structure parameters

Compound	**IC-1**	**IC-2**	**IC-4**
Empirical formula	$C_{11}H_{17}B_{10}NO$	$C_{17}H_{21}B_{10}NO$	$C_{18}H_{23}B_{10}NO_2$
Formula weight	287.35	363.45	393.47
Temperature [K]	130(2)	130(2)	130(2)
Wavelength [pm]	71.073	71.073	71.073
Crystal system	Monoclinic	Triclinic	Monoclinic
Space group	$P\ 2_1/n$	$P\ \bar{1}$	$P\ 2_1/c$
Unit cell dimensions			
a [pm]	746.07(3)	823.26(3)	689.82(1)
b [pm]	1059.03(4)	1079.66(6)	1108.85(2)
c [pm]	1954.29(7)	1158.32(5)	2714.24(5)
α [deg]	90	74.172(4)	90
β [deg]	93.742(3)	71.916(4)	96.802(2)
γ [deg]	90	81.671(4)	90
Volume [nm^3]	1.5408(1)	0.93956(8)	2.06153(6)
Z	4	2	4
$\rho_{(calculated)}$ [Mg/m^3]	1.239	1.285	1.268
μ [mm^{-1}]	0.066	0.070	0.072
F(000)	592	376	816
Crystal size [mm^3]	0.245 · 0.214 · 0.048	0.480 · 0.378 · 0.347	0.393 · 0.374 · 0.203
Θ_{Min} / Θ_{Max} [deg]	2.089 / 28.323	1.906 / 32.518	2.379 / 30.131
Index ranges	-9 ≤ h ≤ 9<br>-12 ≤ k ≤ 14<br>-23 ≤ l ≤ 25	-11 ≤ h ≤ 9<br>-14 ≤ k ≤ 15<br>-17 ≤ l ≤ 16	-9 ≤ h ≤ 9<br>-15 ≤ k ≤ 14<br>-36 ≤ l ≤ 34
Reflections collected	14365	10078	22630
Indp. reflections (R_{int})	3384 (0.0584)	6034 (0.0216)	5647 (0.0300)
Completeness (Θ_{Max})	100.0 % (25.35)	99.7 % (30.51)	100.0 % (28.29)
T_{Max} / T_{Min}	1.00000 / 0.97671	1.00000 / 0.98948	1.00000 / 0.99404
Restraints / parameters	0 / 276	0 / 346	0 / 372
Gof on F^2	1.013	1.020	1.019
R1 / wR2 (I>2σ(I))	0.0537 / 0.1074	0.0551 / 0.1354	0.0440 / 0.1100
R1 / wR2 (all data)	0.1020 / 0.1293	0.0788 / 0.1521	0.0585 / 0.1214
Residual electron density [e·$Å^{-3}$]	0.208 / -0.212	0.415 / -0.222	0.350 / -0.209
CCDC No[1]	2468442	2468443	2468444

Table S1: continued

Compound	**IC-8**	**IC-10**
Empirical formula	$C_{19}H_{25}B_{10}NO_3$	$C_{20}H_{27}B_{10}NO_4$
Formula weight	423.50	453.52
Temperature [K]	130(2)	130(2)
Wavelength [pm]	71.073	71.073
Crystal system	Monoclinic	Monoclinic
Space group	$P\ 2_1/n$	$P\ 2_1/n$
Unit cell dimensions		
a [pm]	1427.50(5)	951.01(2)
b [pm]	859.17(2)	996.17(2)
c [pm]	1913.36(6)	2490.26(5)
α [deg]	90	90
β [deg]	105.415(3)	96.676(2)
γ [deg]	90	90
Volume [nm^3]	2.2623(1)	2.34320(8)
Z	4	4
$\rho_{(calculated)}$ [Mg/m^3]	1.243	1.286
μ [mm^{-1}]	0.074	0.079
F(000)	880	944
Crystal size [mm^3]	0.305 · 0.160 · 0.094	0.416 · 0.247 · 0.157
Θ_{Min} / Θ_{Max} [deg]	2.068 / 28.212	2.204 / 30.443
Index ranges	$-18 \le h \le 14$<br>$-11 \le k \le 11$<br>$-23 \le l \le 25$	$-13 \le h \le 12$<br>$-14 \le k \le 13$<br>$-34 \le l \le 35$
Reflections collected	16534	33614
Indp. reflections (R_{int})	4976 (0.0553)	6569 (0.0332)
Completeness (Θ_{Max})	100.0 % (25.35)	100.0 % (28.29)
T_{Max} / T_{Min}	1.00000 / 0.92963	1.00000 / 0.99415
Restraints / parameters	0 / 398	0 / 424
Gof on F^2	1.005	1.049
R1 / wR2 (I>2σ(I))	0.0620 / 0.1229	0.0449 / 0.1060
R1 / wR2 (all data)	0.1176 / 0.1502	0.0601 / 0.1145
Residual electron density [e·$Å^{-3}$]	0.233 / -0.254	0.291 / -0.231
CCDC No	2468445	2468446

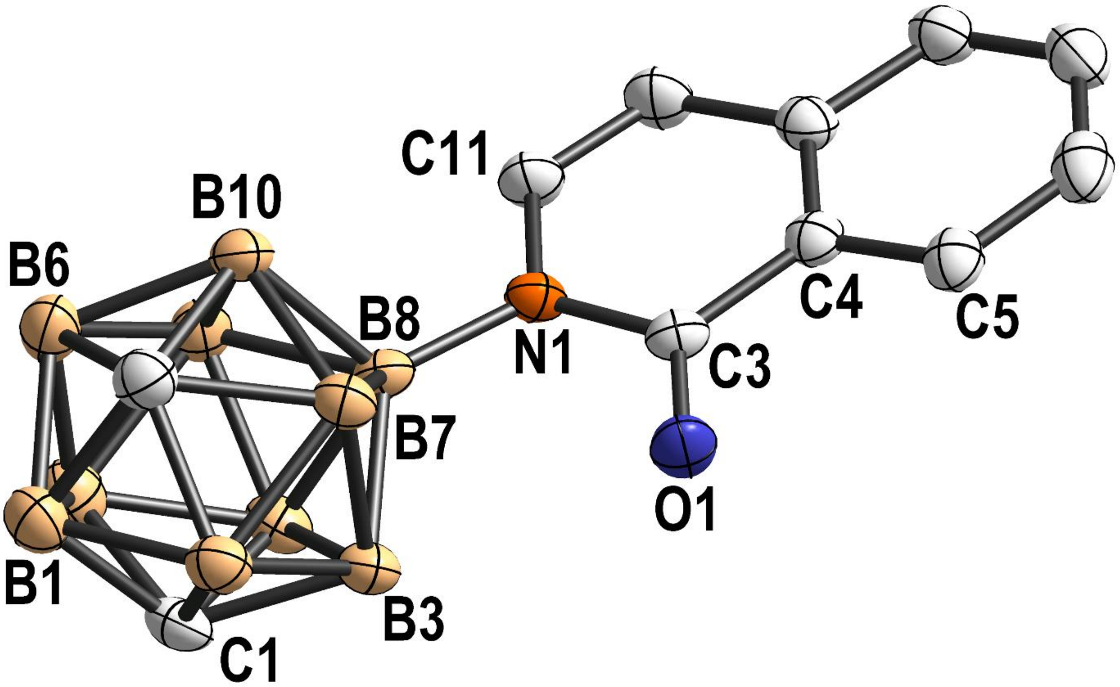

Figure S128. Molecular structure and labeling scheme of **IC-1**. Hydrogen atoms were omitted for clarity. Displacement ellipsoids are drawn at the 50% probability level.

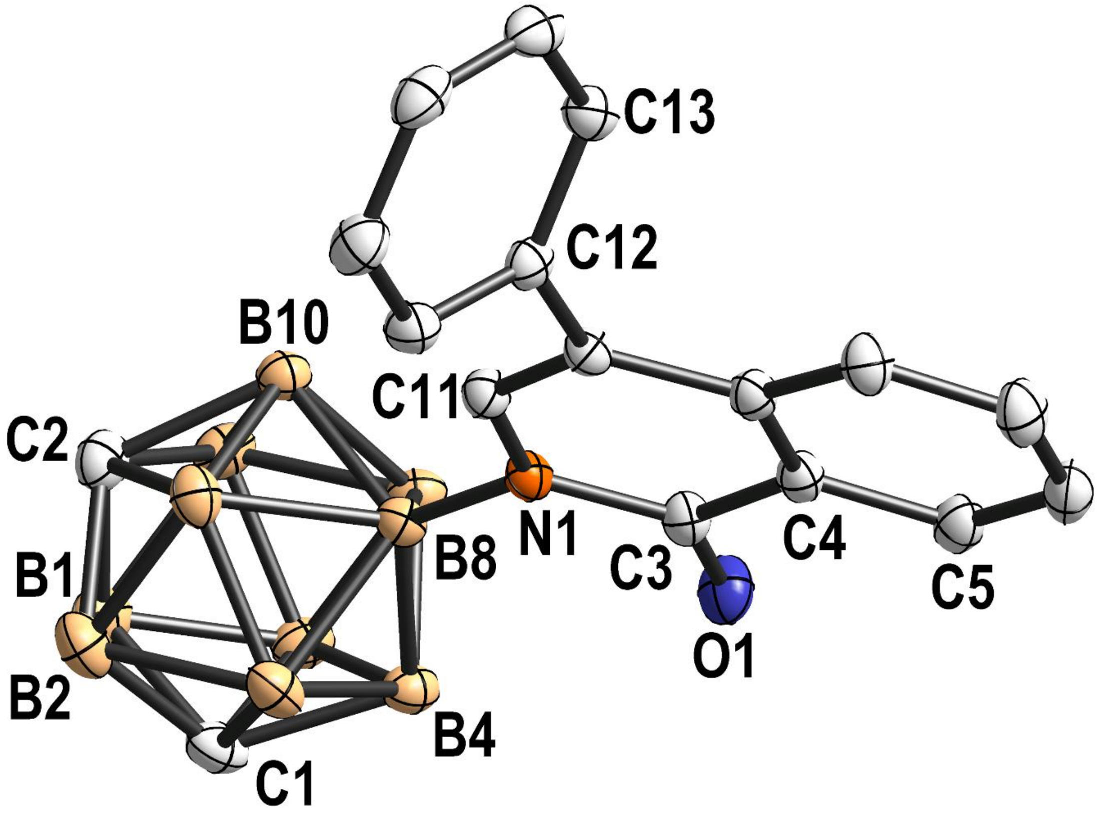

Figure S129. Molecular structure and labeling scheme of **IC-2**. Hydrogen atoms were omitted for clarity. Displacement ellipsoids are drawn at the 50% probability level.

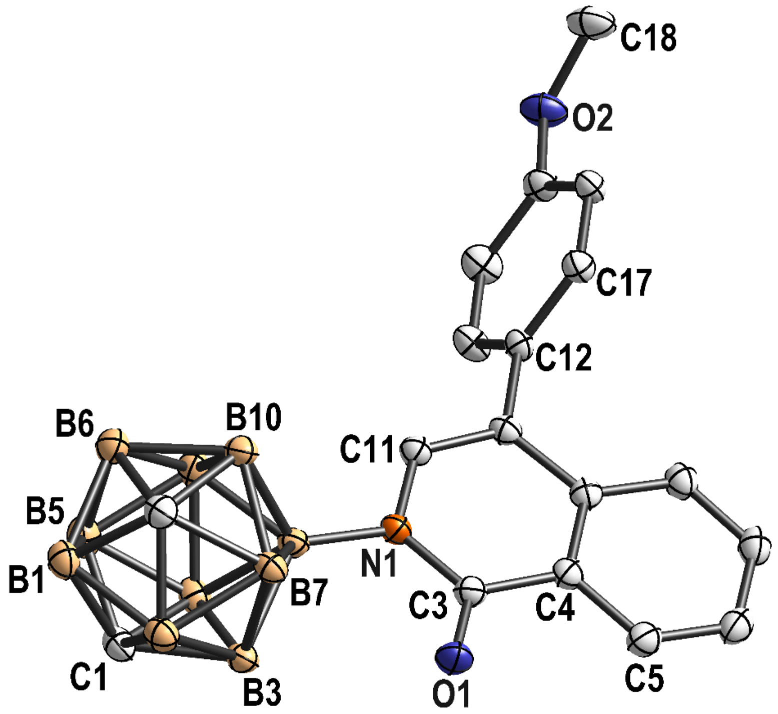

Figure S130. Molecular structure and labeling scheme of **IC-4**. Hydrogen atoms were omitted for clarity. Displacement ellipsoids are drawn at the 50% probability level.

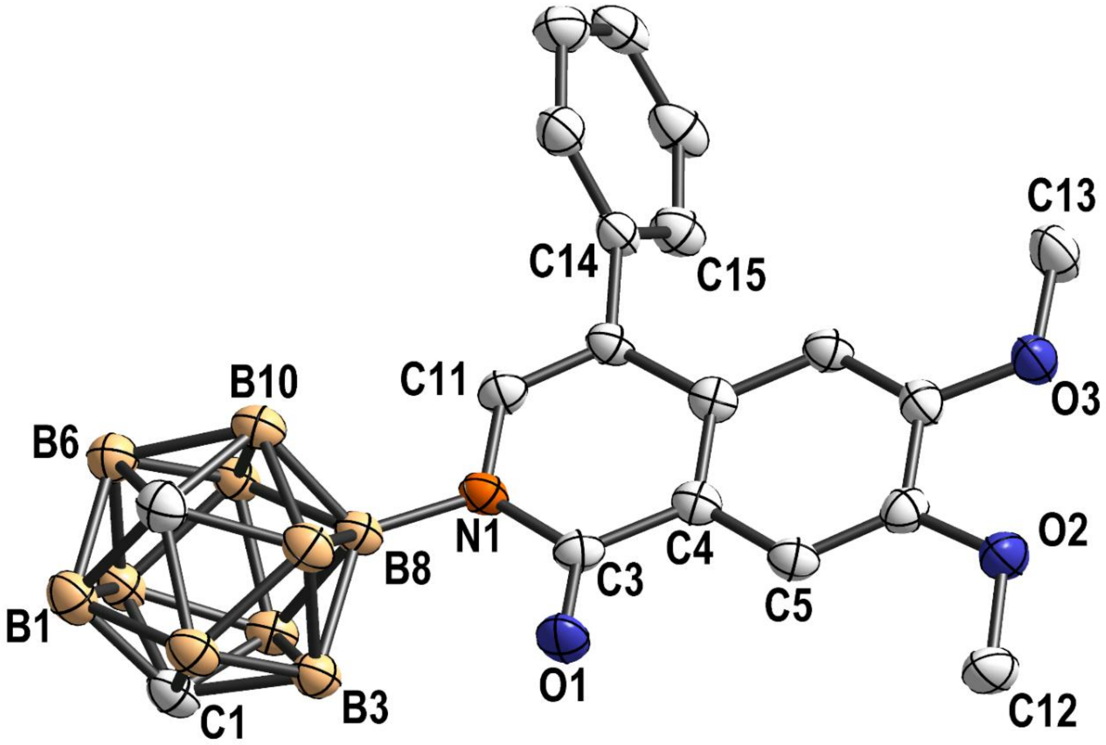

Figure S131. Molecular structure and labeling scheme of **IC-8**. Hydrogen atoms were omitted for clarity. Displacement ellipsoids are drawn at the 50% probability level.

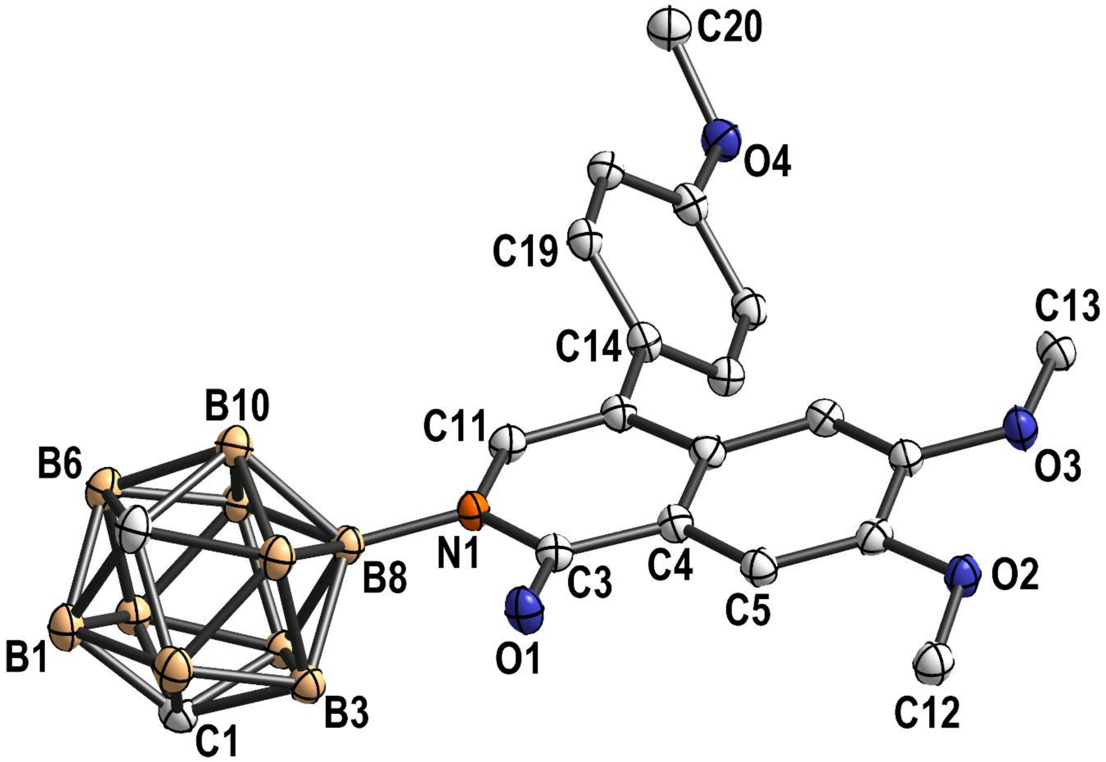

Figure S132. Molecular structure and labeling scheme of **IC-10**. Hydrogen atoms were omitted for clarity. Displacement ellipsoids are drawn at the 50% probability level.

4. Biological data

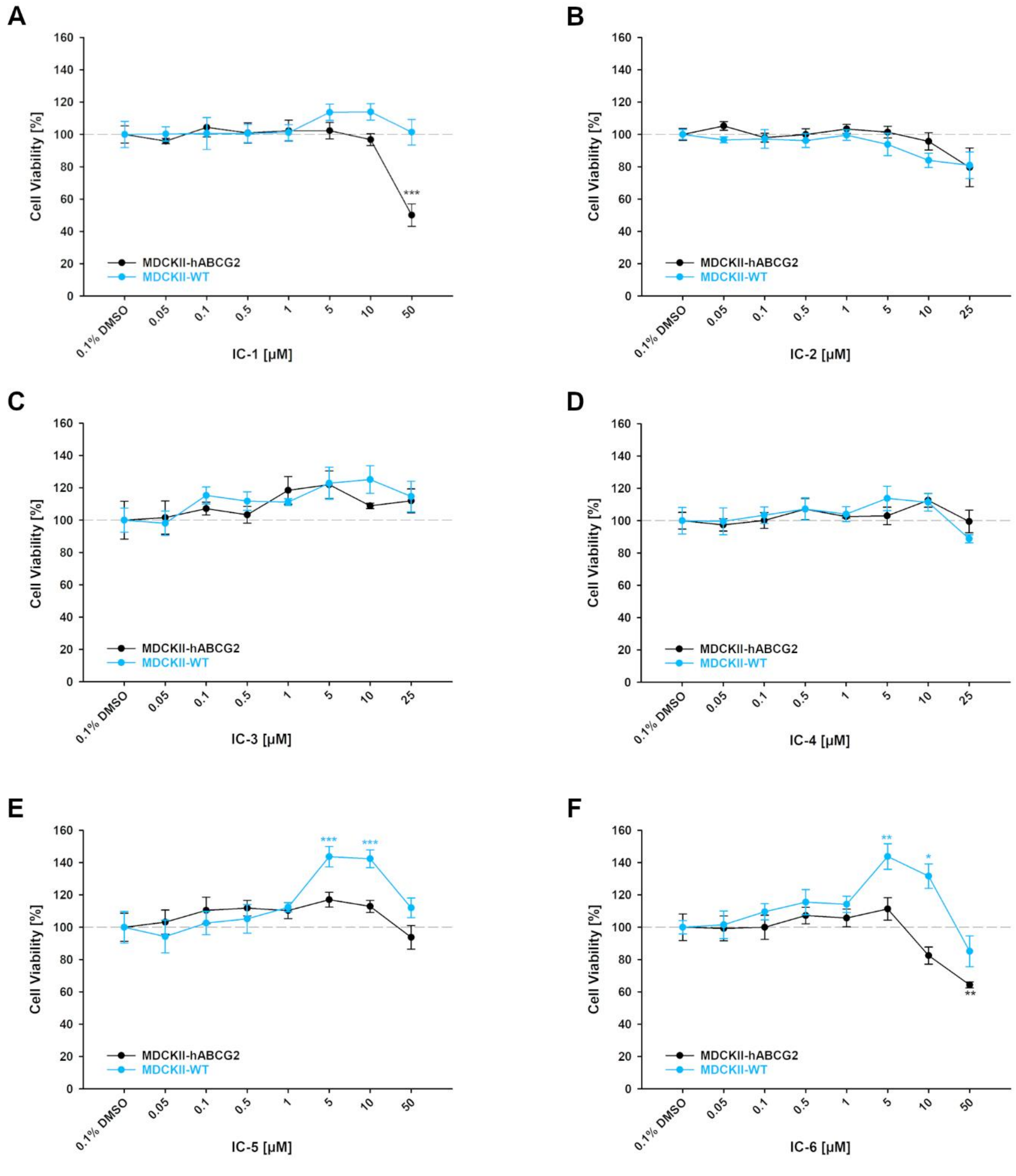

Figure S133. Cytotoxicity of non-substituted *N*-carboranyl isoquinolinones derivatives (**A**) **IC-1**, (**B**) **IC-2**, (**C**) **IC-3**, (**D**) **IC-4**, (**E**) **IC-5** and (**F**) **IC-6** toward MDCKII-hABCG2 (black) and MDCKII-WT cells (blue). MDCKII cells were treated with increasing concentrations of each examined compound in the mentioned concentrations for 48 h. Afterwards, cell viability was assessed by water-soluble tetrazolium-1 (WST-1) assay. Data were normalized to solvent control (0.1% DMSO), which was set as 100% (mean ± SEM, N = 3, one-way ANOVA with Holm-Šidák post-hoc test, * indicating significant difference in comparison to the solvent control, *** $p \le 0.001$, ** $p \le 0.01$, * $p \le 0.05$).

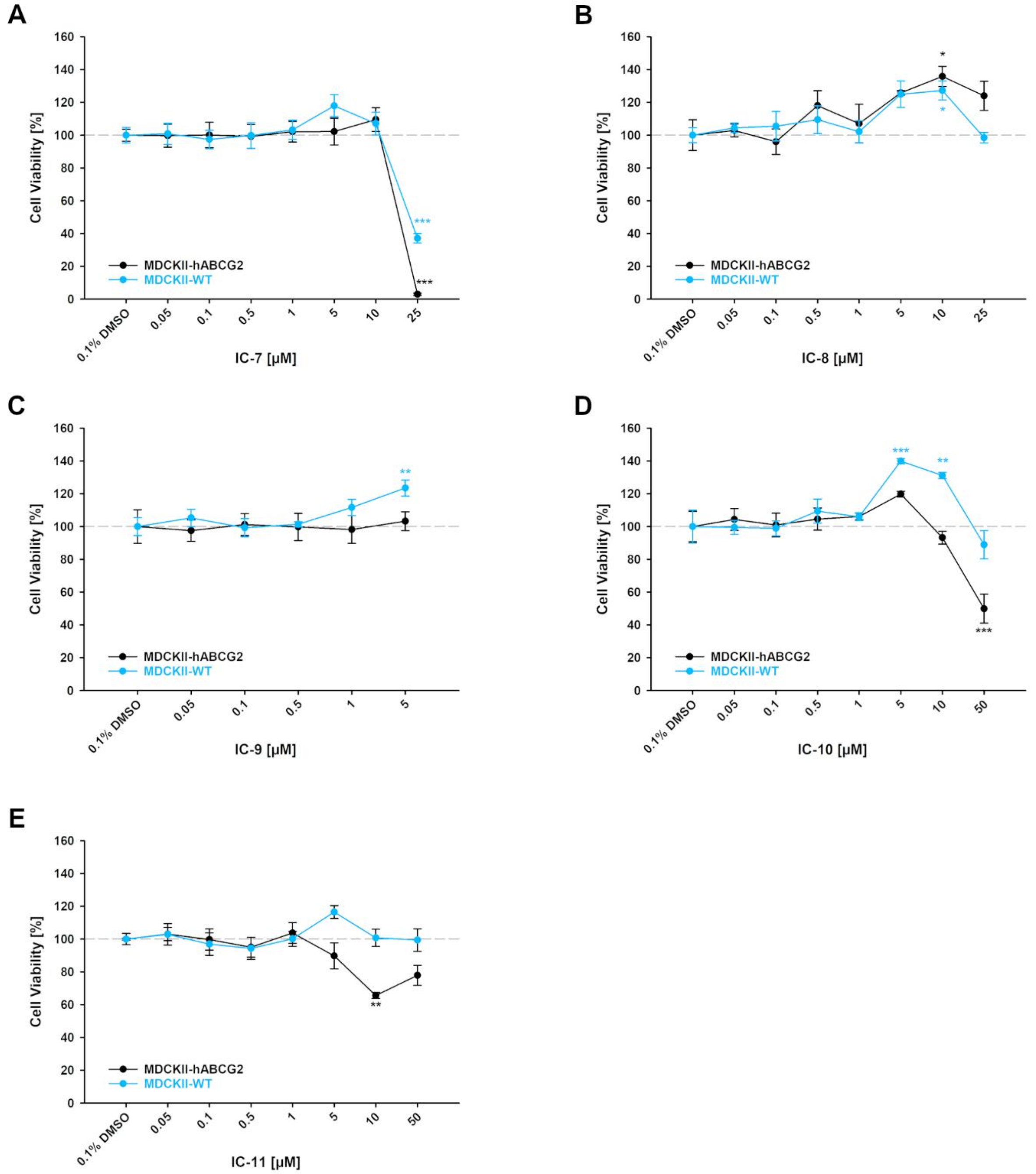

Figure S134. Cytotoxicity of *N*-carboranyl 6,7-dimethoxyisoquinolinones derivatives (**A**) **IC-7**, (**B**) **IC-8**, (**C**) **IC-9**, (**D**) **IC-10**, and (**E**) **IC-11** toward MDCKII-hABCG2 (black) and MDCKII-WT cells (blue). MDCKII cells were treated with increasing concentrations of each investigational compound in the mentioned concentrations for 48 h. Afterwards, cell viability was assessed by water-soluble tetrazolium-1 (WST-1) assay. Data were normalized to solvent control (0.1% DMSO), which was set as 100% (mean ± SEM, N = 3, one-way ANOVA with Holm-Šidák post-hoc test, * indicating significant difference in comparison to the solvent control, *** $p \le 0.001$, ** $p \le 0.01$, * $p \le 0.05$).

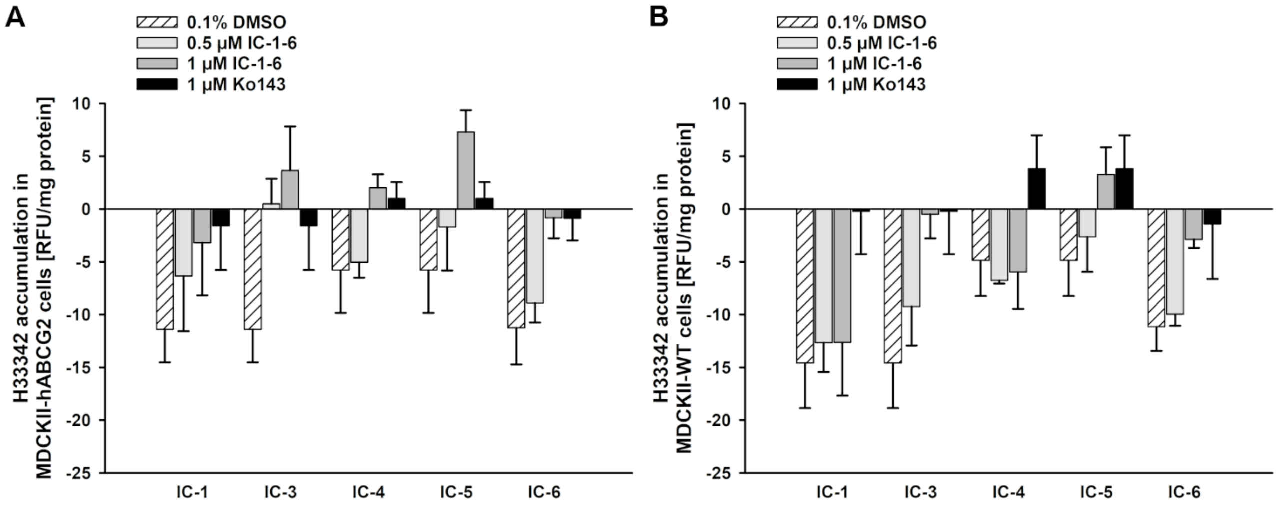

Figure S135. Autofluorescence of unsubstituted *N*-carboranyl isoquinolinones **IC-1 – IC-6** in (**A**) MDCKII-hABCG2 and (**B**) MDCKII-WT cells. Cells were incubated with 0.5 µM and 1.0 µM of the **IC-1 – IC-6** for 4 h. Thereafter, cells were lysed and intracellular fluorescence was detected as described. Data are given as mean ± SEM (N = 3, one-way ANOVA with Holm-Šidák post-hoc test, * significant difference in comparison to the solvent control: p ≤ 0.05).

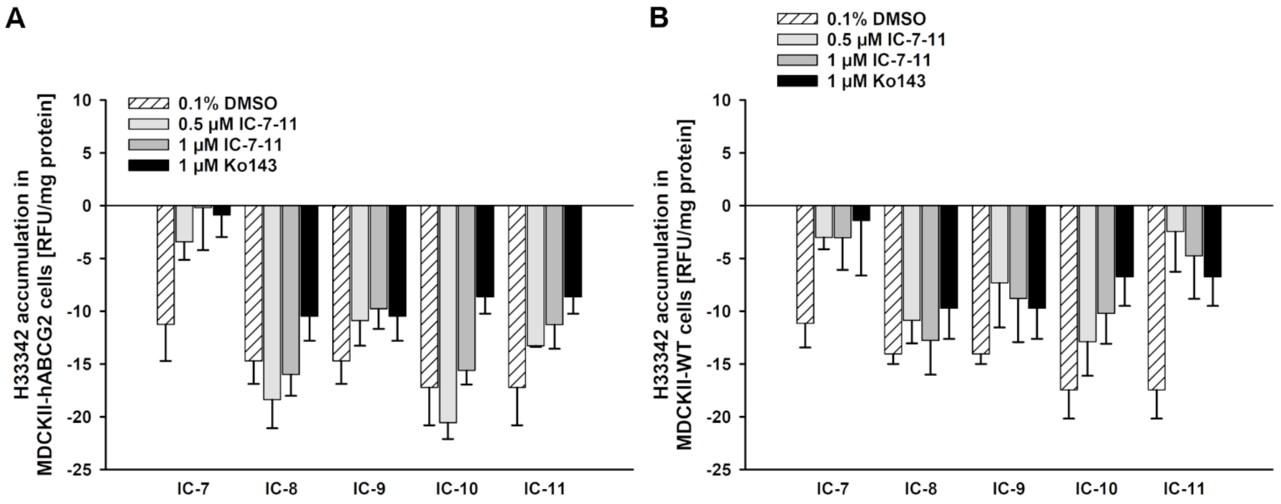

Figure S136. Autofluorescence of *N*-carboranyl 6,7-dimethoxy isoquinolinones **IC-7 – IC-11** in (**A**) MDCKII-hABCG2 and (**B**) MDCKII-WT cells. Cells were incubated with 0.5 µM and 1.0 µM of the **IC-7 – IC-11** for 4 h. Thereafter, cells were lysed and intracellular fluorescence was detected as described. Data are given as mean ± SEM (N = 3, one-way ANOVA with Holm-Šidák post-hoc test, * significant difference in comparison to the solvent control: p ≤ 0.05).

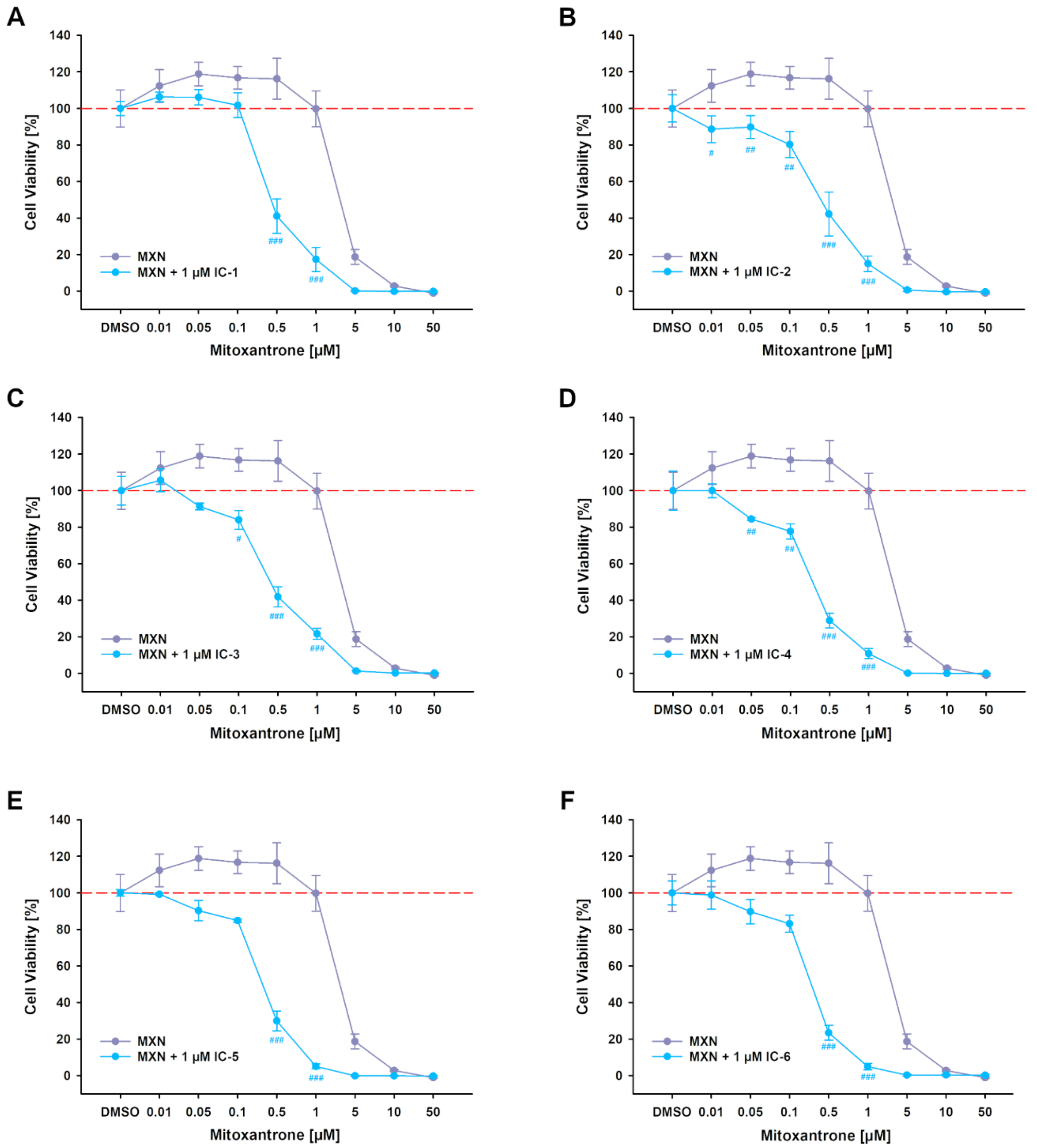

Figure S137. Reversal of ABCG2-mediated mitoxantrone resistance of unsubstituted *N*-carboranyl isoquinolinones **IC-1 – IC-6** in MDCKII-hABCG2 cells. Cells were treated with increasing concentration of MXN alone or in combination with 1.0 µM of (**A**) **IC-1**, (**B**) **IC-2**, (**C**) **IC-3**, (**D**) **IC-4**, (**E**) **IC-5** and (**F**) **IC-6** for 48 h. Afterwards, cell viability was determined by WST-1 assay. Data were normalized to solvent control (0.1% DMSO) and were shown as mean ± SEM (N = 4, one-way ANOVA with Holm-Šidák post-hoc test, # significant different to MXN alone, ### $p \leq 0.001$, ## $p \leq 0.01$, # $p \leq 0.05$).

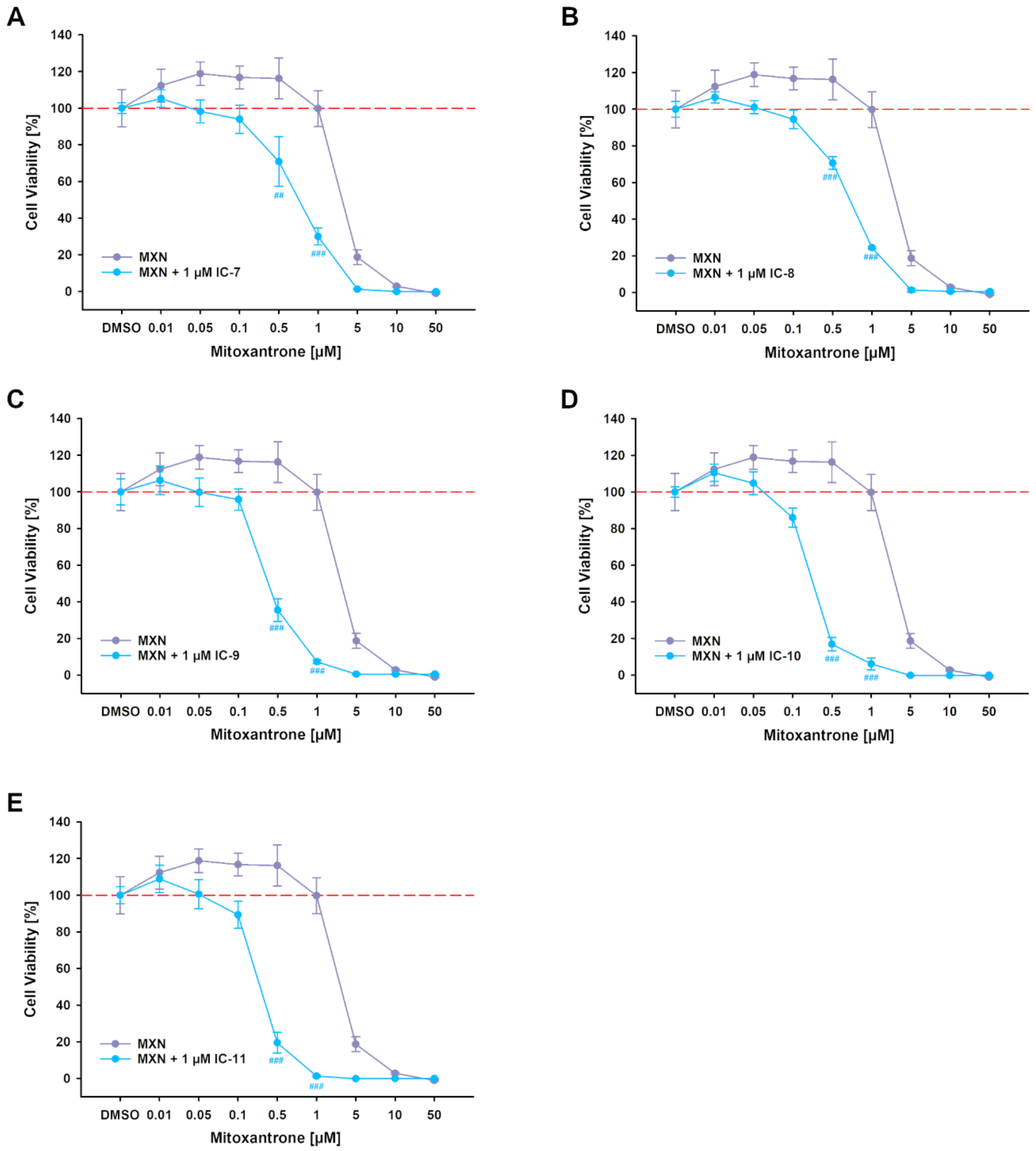

Figure S138. Reversal of ABCG2-mediated mitoxantrone resistance of *N*-carboranyl 6,7-dimethoxy isoquinolinones **IC-7 – IC-11** in MDCKII-hABCG2 cells. Cells were treated with increasing concentration of MXN alone or in combination with 1.0 µM of (**A**) **IC-7**, (**B**) **IC-8**, (**C**) **IC-9**, (**D**) **IC-10** and (**E**) **IC-11** for 48 h. Afterwards, cell viability was determined by WST-1 assay. Data were normalized to solvent control (0.1% DMSO) and were shown as mean ± SEM (N = 4, one-way ANOVA with Holm-Šidák post-hoc test, # significant different to MXN alone, ### p ≤ 0.001, ## p ≤ 0.01).

5. Computational Data

Supplementary Text S1 Description of Docking results

The predicted binding modalities of the examined carboranyl isoquinolinone derivatives revealed, except for compound **IC-3**, strong affinity within the inner, central S1 binding pocket. Compound **IC-3**, surprisingly, showed interactions with the ABCG2 protein in a lateral binding pocket S2,[2,3] despite the merely marginal change in its structure (Figure S151A).
However, despite the similarities in structure and orientation, deviations in the binding poses of the studied derivatives were found. Compound **IC-1** and its dimethoxy derivative **IC-7** demonstrated different predicted simulations. While **IC-1** has two molecules located symmetrically to each other in the inner cavity, only one molecule **IC-7** non-specifically blocks the binding pocket.
In Figure S151D shows the top-ranked poses of the 4-methoxy-, 3,4-dimethoxy- and 3,4,5-trimethoxyphenyl derivatives (**IC-4, IC-5** and **IC-6**) and their 6,7-dimethoxyisoquinolinones derivatives (**IC-10** and **IC-11**) within the binding pocket S1. The top scored poses of compounds **IC-4** and **IC-6** virtually overlap, whereas the simulated structure of compound **IC-5** exhibits a distinct rotation of the molecule due to unfavorable interactions of the 3-methoxy group, and thus different interactions with the protein side chains ((Figure S151B).

In general, the examined structures were mainly found in the slit-like gap (binding pocket S1) between the BCRP monomers. As reported before for comparable annelated ring system-based structures,[4] the flat isoquinolinone scaffold or phenyl substituent revealed stacking-like π-π interactions between the opposing Phe-439 residues of the monomers. With strong interactions of the amino acids Val-401, Leu-405, Thr-542, Ile-543, and Val-546, the carborane moiety of the strongest reversers, **IC-4**, **IC-6** and **IC-10**, is located in a hydrophobic pocket. Furthermore, the 4-methoxyphenyl substituent of **IC-4** and **IC-10** appeared to be beneficial for the orientation toward the amino acid side chains of Thr-435 and Asn-436. A 2D interaction diagram of **IC-10** and the protein side chains is presented in Figure S151C. However, the putative binding poses and deviations in orientation of the examined structures do not yield sufficient evidence to explain the marginal differences of found *in vitro* chemoresistance reversal results.

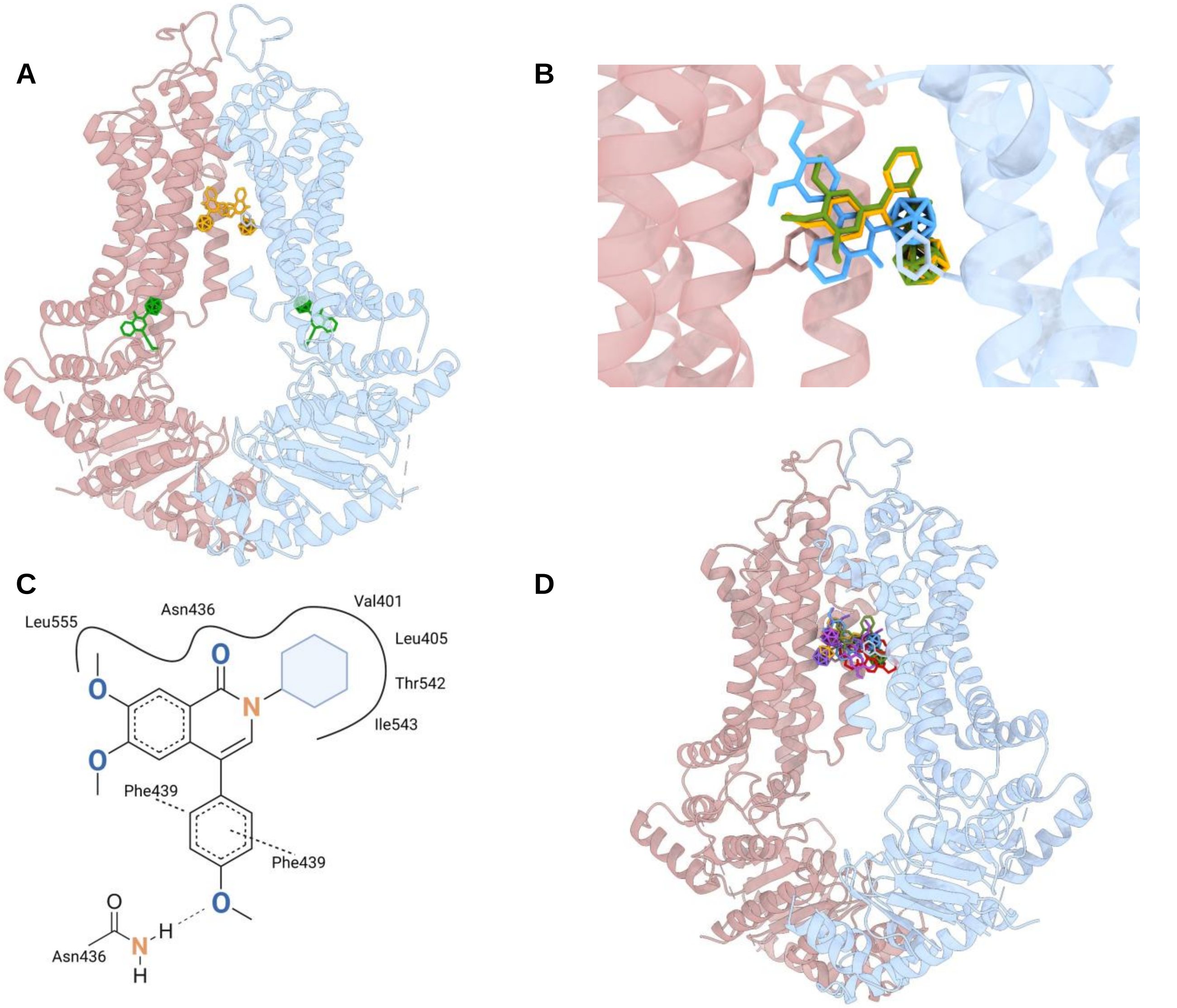

Figure S139. (**A**) Cartoon representation of ABCG2, with monomers depicted in rose and blue. Top-ranked binding poses of **IC-3** (green) and **IC-4** (yellow) within the lateral cavity S2 and the inner cavity S1, respectively. (**B**) Comparison of the highest scoring simulated poses of **IC-4** (yellow), **IC-5** (blue) and **IC-6** (olive); structures shown as stick model; hydrogen atoms omitted for clarity. (**C**) 2D interaction diagram of the top-score poses of **IC-10**; blue hexagon represents the carboranyl moiety. (**D**) Docking of **IC-4** (yellow), **IC-5** (blue), **IC-6** (olive), **IC-10** and **IC-11** (violet) into the crystal structure of ABCG2 (5NJ3) in comparison with mitoxantrone (red).

Table S2: Free binding energies of compounds **IC-1** to **IC-11** towards human ABCG2 transporter in rigid ABCG2 protein (PDB code 5NJ3).

Compound	Free binding energy [kcal/mol] 5NJ3
**IC-1**	−6.6
**IC-2**	−7.2
**IC-3**	−3.2
**IC-4**	−7.9
**IC-5**	−9.3
**IC-6**	−9.6
**IC-7**	−7.0
**IC-8**	−8.0
**IC-9**	−6.7
**IC-10**	−8.6
**IC-11**	−9.2

6. References

[1] Deposition numbers CCDC 2468442 (IC-1), 2468443 (IC-2), 2468444 (IC-4), 2468445 (IC-8), and 2468446 (IC-10) contain the supplementary crystallographic data for this paper. These data are provided free of charge by the joint Cambridge Crystallographic Data Centre and Fachinformationszentrum Karlsruhe Access Structures service., can be found under https://summary.ccdc.cam.ac.uk/structure-summary-form.

[2] K. Silbermann, J. Li, V. Namasivayam, S. M. Stefan, M. Wiese, “Rational drug design of 6-substituted 4-anilino-2-phenylpyrimidines for exploration of novel ABCG2 binding site” *Eur. J. Med. Chem.* **2021**, *212*, 113045, DOI 10.1016/j.ejmech.2020.113045.

[3] K. Silbermann, J. Li, V. Namasivayam, F. Baltes, G. Bendas, S. M. Stefan, M. Wiese, “Superior Pyrimidine Derivatives as Selective ABCG2 Inhibitors and Broad-Spectrum ABCB1, ABCC1, and ABCG2 Antagonists” *J. Med. Chem.* **2020**, *63*, 10412, DOI 10.1021/acs.jmedchem.0c00961.

[4] M. Moinul, S. A. Amin, T. Jha, S. Gayen, “Updated chemical scaffolds of ABCG2 inhibitors and their structure-inhibition relationships for future development” *Eur. J. Med. Chem.* **2022**, *241*, 114628, DOI 10.1016/j.ejmech.2022.114628.
